# Clinical implications of fracture severity risk with pioglitazone: a systematic review and meta-analysis of clinical randomized trials

**DOI:** 10.3389/fphar.2025.1357309

**Published:** 2025-03-06

**Authors:** Hala F. Azhari, Jesse Dawson

**Affiliations:** ^1^ College of Medicine and Pharmacy, Umm Al-Qura University, Makkah, Saudi Arabia; ^2^ School of Cardiovascular and Metabolic Health, University of Glasgow, Glasgow, United Kingdom

**Keywords:** bone fracture, pioglitazone, rosiglitazone, thiazolidinediones, antihyperglycemic, randomized clinical trials

## Abstract

**Introduction:**

Pioglitazone, a thiazolidinedione, effectively reduces stroke and cardiovascular events in individuals with type 2 diabetes, insulin resistance, and/or stroke. However, its potential to increase fracture risk, particularly among women and those with pre-existing skeletal conditions, has not yet been completely understood. This meta-analysis aims to clarify fracture risk associated with pioglitazone, thereby focusing on individuals with a history of stroke.

**Methods:**

A systematic review was performed for clinical trials conducted up to March 2024, focusing on trials comparing pioglitazone to placebo or other antihyperglycemic drugs that reported fracture outcomes.

**Results:**

From 860 trials identified, 78 satisfied the inclusion criteria: 34 with a high risk of bias, 8 with unclear risk, and 36 with low risk. The meta-analysis revealed an association between pioglitazone and a significant increase in fracture risk (risk ratio [RR] 1.21; 95% CI 1.01–1.45; *P* = 0.04), including non-serious (RR 1.25; 95% CI 1.03–1.51; *P* = 0.02) and serious fractures (RR 1.48; 95% CI 1.10–1.98; *P* = 0.01). Notably, the risk was exacerbated for low-energy fractures, particularly resulting from falls (RR 1.49; 95% CI 1.20–1.87; *P* = 0.0004), in insulin resistance individuals (RR 0.87; 95% CI 0.43–1.76; *P* = 0.69), and stroke survivors (RR 1.41; 95% CI 1.09–1.83; *P* = 0.008). Fractures were most frequently observed in lower extremities (RR 1.85; 95% CI 1.33–2.56; *P* = 0.0002), with women at a greater risk (RR 1.56; 95% CI 1.20–2.02; *P* = 0.0008). When compared with other antihyperglycemic drugs, no significant difference in fracture risk was noted (RR 1.08; 95% CI 0.73–1.59; *P* = 0.70), except rosiglitazone, which showed higher fracture risk (RR 1.42; 95% CI 1.23–1.64; *P* < 0.00001). Fracture risk was significant in the fixed-effect model but not in the random-effects model.

**Discussion:**

Though pioglitazone offers several cardiovascular benefits, its association with increased fracture risk, especially among women and non-diabetic individuals post-stroke, warrants careful consideration. Individualized treatment interventions balancing cardiovascular and skeletal outcomes are essential, and further research is needed to optimize therapeutic strategies in this population.

**Systematic Review Registration:**

https://www.crd.york.ac.uk/PROSPERO/display_record.php?ID=CRD42016038242, identifier CRD42016038242.

## 1 Introduction

Stroke survivors face a significantly elevated risk of falls and the associated risk of fractures (Batchelor et al., 2012), with the likelihood of fracture escalating two-to four-fold following a stroke-related fall (Kapral et al., 2017). During hospitalization, between 14% and 65% of people who had strokes experience at least one fall (Teasell et al., 2002), while 37%–73% of falls occur within the first 6 months after hospital discharge (Kerse et al., 2008). This heightened risk of fractures post-stroke, particularly in individuals with pre-existing frailty, may be underestimated as fractures are frequently attributed to falls rather than being viewed as an independent risk. In individuals with osteoporosis, each standard deviation decrease in bone mineral density (BMD) corresponds to an approximately 1.5-fold increase in mortality risk (Kado et al., 2000). Although comorbidities significantly contribute to this association, fracture events themselves may also play a role, either directly or indirectly. Stroke-induced impairments such as functional disabilities (including diminished muscle strength, balance issues, and decreased BMD) (Yavuzer et al., 2002), vitamin D deficiency, prolonged immobility, and structural changes (such as hemineglect and perceptual or visual problems) (Teasell et al., 2002), further exacerbate the risk of occurrence of falls and fractures in post-stroke patients (Batchelor et al., 2012). Therefore, addressing both stroke and fracture risk is critical to mitigating post-stroke morbidity and mortality.

Thiazolidinediones (TZDs), such as pioglitazone and rosiglitazone, are primarily used to improve insulin sensitivity through the activation of peroxisome proliferator-activated receptor-gamma (PPAR-γ) (Kim et al., 2012). These medications enhance glucose and lipid metabolism by enhancing glucose uptake and lipid storage in adipose tissues, making such medications valuable for glycemic control in type 2 diabetes. Beyond their metabolic benefits, pioglitazone has shown significant cardiovascular advantages. The Insulin Resistance Intervention after Stroke (IRIS) (Kernan et al., 2016) trial demonstrated that pioglitazone substantially reduces the risk of recurrent stroke and myocardial infarction in patients with insulin resistance who have experienced a prior stroke. Additionally, pioglitazone improves lipid profiles and exerts anti-inflammatory effects, thereby further enhancing its cardiovascular utility. However, the use of pioglitazone causes some adverse effects. Among its notable adverse effects are weight gain, peripheral edema, and an increased risk of fractures (Kernan et al., 2016). These complications can mitigate its overall benefits, particularly the fracture risk, which necessitates careful patient selection and monitoring of the patients.

A further area of concern is the potential association of pioglitazone with bladder cancer (Nien and Tseng, 2014), which eludes consensus. Epidemiological evidence (Tang et al., 2018) suggests a modestly elevated risk of bladder cancer associated with pioglitazone use, with the highest risks observed in patients with prolonged use (exceeding 2 years) or high cumulative doses. Pathophysiological studies provide several plausible mechanisms for this association. In animal models (Bojková et al., 2014), chronic exposure to pioglitazone has been shown to induce urothelial hyperplasia and carcinoma, likely due to oxidative stress, activation of PPAR-γ, and disruption of normal cell proliferation and apoptosis. In humans (Guan et al., 1999), pioglitazone metabolites may accumulate in the bladder, leading to DNA damage or inflammatory responses that promote tumorigenesis. Despite these findings, the evidence remains inconclusive (Tang et al., 2018), with human studies often yielding conflicting results due to methodological limitations, confounding factors, and potential biases, including conflicts of interest in industry-sponsored research. Consequently, the association between pioglitazone and bladder cancer has not been definitively established, but the concern has led to some regulatory actions, such as the drug’s removal from the market in some countries and heightened scrutiny in other countries.

Considering these conflicting outcomes, the use of pioglitazone requires a personalized and cautious approach. Clinicians must weigh its demonstrated cardiovascular benefits and improvements in insulin sensitivity against the potential risks of adverse events, including fractures. For patients at higher risk of fracture complications, alternative therapeutic options should be considered. Ultimately, ongoing research and vigilant post-market surveillance remain crucial to refine the risk-benefit profile of pioglitazone and ensure its safe and effective use in clinical practice.

To evaluate the net clinical benefit of pioglitazone in post-stroke patients, especially concerning fracture risk, further research is essential. This may include a meta-analysis of randomized controlled trials (RCTs) to determine the fracture risk associated with pioglitazone use in adults, with a specific focus on stroke survivors. It is hypothesized that pioglitazone and TZDs may increase fracture risk in patients with or without diabetes, compared to placebo or other anti-hyperglycemic (AHG) drugs. This research is critical for guiding therapeutic decisions and optimizing patient outcomes while maintaining patient safety.

## 2 Methods

The meta-analysis adhered to the Preferred Reporting Items for Systematic Reviews and Meta-Analyses guidelines (Stewart et al., 2015). The study protocol was formally registered at the University of York under registration number PROSPERO-CRD 42016038242 (Azhari et al., 2021).

### 2.1 Data sources

A comprehensive search was conducted across multiple databases, including the Cochrane Central Register of Controlled Trials, Cochrane Database of Systematic Reviews, EMBASE, MEDLINE, Web of Science, and ClinicalTrials.gov, (NIH U.S National Library of Medicine, 2022), to identify relevant RCTs published since inception to March 2024. The search strategy utilized a broad spectrum of medical terminology related to fractures, TZDs, and AHGs, as detailed in Supplementary Appendix 1.

### 2.2 Study selection

RCTs involving participants aged 18 years or older, which reported fracture outcomes in individuals treated with pioglitazone (TZD), compared to placebo or other AHGs, were eligible for inclusion. Trials were excluded if they did not meet these criteria, specifically if they were not RCTs, did not report fractured outcomes, or included participants younger than 18 years. Additionally, non-peer-reviewed sources or trials lacking robust fracture data were omitted to maintain the quality and relevance of the meta-analysis. Duplicate publications were rigorously screened and removed, with only the most comprehensive and complete version retained for final analysis to avoid any data duplication and redundancy.

### 2.3 Data extraction

The extracted data encompassed a comprehensive range of variables, including the baseline demographic and clinical characteristics of the study population, the specific design and methodology of each trial, the total number of participants enrolled, and detailed information on the backgrounds of both AHG users and non-users. Additionally, data regarding the duration of follow-up for each study were collected, which offered key insight into the long-term outcomes and potential cumulative effects of pioglitazone and other AHG drugs on fracture risk.

### 2.4 Quality assessment

Each RCT was assessed for risk of bias, adhering to the Cochrane Quality Assessment Tool (Higgins et al., 2003). This tool evaluates six key domains of bias: selection, detection, performance, attrition, reporting, and other potential biases. Each key domain was rated as “low risk,” “high risk,” or “unclear,”. The unclear rating indicates insufficient information to determine bias. Trials that scored “high risk” in any of the first three domains were classified as providing low-quality evidence.

Additionally, the overall certainty of the evidence was appraised using the Grading of Recommendations Assessment, Development, and Evaluation (GRADE) approach (Guyatt et al., 2008). Evidence quality was considered ‘high’ if multiple trials exhibited a low risk of bias across different domains. However, ratings were downgraded in the presence of concerns related to trial design, inconsistency in findings, indirectness of evidence, imprecision, or potential publication bias. The principal investigator conducted a thorough quality assessment of the included trials, while the other investigators independently addressed any discrepancies to ensure the precision and robustness of the conclusions.

### 2.5 Outcomes measured

The primary outcome of interest was the incidence of fractures, which were analyzed as safety endpoints. These outcomes were stratified considering the severity of events. The fractures were categorized as either non-serious or serious (those resulting in hospitalization, surgery, or physiotherapy). Another criterion to classify fractures was based on their underlying mechanisms, such as stress-related (e.g., repetitive stress or minimal trauma) versus non-stress-related. Fractures were also categorized by energy level: high-energy fractures resulted from significant trauma (e.g., falls from substantial heights or motor vehicle accidents), while low-energy fractures arose from minimal trauma (e.g., falls from standing height or lower). Additionally, pathological fractures, associated with underlying conditions such as osteoporosis, were distinguished from non-pathological ones.

The timing of fractures relative to TZD exposure (before or after treatment initiation) was analyzed alongside fracture location, including key anatomical sites such as the spine, hip, pelvis, femur, and upper and lower extremities (including wrist and ankle). BMD data were also collected for a more comprehensive assessment of bone health and fracture susceptibility. This detailed framework allowed for the examination of pioglitazone’s impact on fracture risk across different subgroups, such as male and female individuals, thereby providing valuable insights into the gender-specific risks of TZD therapy. All fracture events were classified according to the Medical Dictionary for Regulatory Activities (MedDRA, 2022a).

### 2.6 Data synthesis

In trials that simultaneously reported fracture risk by subtype (serious or non-serious adverse events), one of these fractured outcomes data was excluded from the intervention of the interest group, re-reported the data of fracture by subtypes separately, and then compared the intervention of interest to placebo or other AHGs.

The comparator AHGs, either as monotherapy or in combination, were categorized into the following drug classes: metformin, sulfonylureas, TZDs, dipeptidyl peptidase-4 inhibitors, glucagon-like peptide-1 agonists, sodium-glucose co-transporter-2 inhibitors, α-glucosidase inhibitors, and insulin. To avoid unit-of-analysis errors, trials involving multiple doses of TZDs were aggregated to develop a unified pairwise comparator.

In trials with two or more intervention groups, where all participants, including those in control and comparator groups, were exposed to TZDs, a high risk of bias was identified. Although these trials were included in the study to provide a comprehensive evaluation, their fracture outcome data were excluded from the meta-analysis to maintain accuracy.

### 2.7 Statistical analyses

A meta-analysis was conducted to aggregate pooled data for quantitative evaluation. For determining dichotomous outcomes, relative risks (RRs), 95% confidence intervals (CIs), and weighted mean differences were computed. Continuous variables were assessed by calculating the mean values of BMD levels, sample sizes, and standardized mean differences (SMD). Fracture outcomes were combined using the Mantel–Haenszel test, incorporating a continuity correction of 0.5 for trials reporting zero events in any treatment arm. Trials with zero events in both arms were excluded to prevent data distortion.

The meta-analysis addressed fracture risk, including mechanisms and severity, across various skeletal sites, participant sex, and BMD levels. Several fracture events were analyzed to evaluate the overall safety profile of pioglitazone compared to placebo or other AHGs, considering participants with insulin resistance or type 2 diabetes mellitus (T2DM), and those with or without cardiovascular disease (CVD) risk, including stroke. These analyses accounted for different baseline characteristics across studies.

Both fixed and random-effects models were applied in the analysis. The fixed-effect model assumes a common effect size across studies, applicable when heterogeneity within the meta-analysis is minimal. In contrast, the random-effects model accommodates unexplained heterogeneity by assuming varied effect sizes across studies, with effects related to the intervention. This model, based on inverse variance, adjusted the study weights according to the degree of heterogeneity or variation among different intervention effects (Riley et al., 2011).

Inter-study heterogeneity was evaluated using the *Q* statistic and quantified by the *I* ^*2*^ statistic, where an *I* ^*2*^ value ≥75% indicated substantial heterogeneity not attributable to chance (Higgins et al., 2003). Potential reporting bias was examined using Egger’s linear regression (Egger et al., 1997) and Begg’s rank correlation tests (Begg and Mazumdar, 1994), with results visually inspected using funnel plots. These plots depicted the total trial inverse sample size (standard error) against the natural logarithm of the treatment-effect magnitude of RRs. A symmetrical inverted funnel suggested no reporting bias.

All statistical tests were two-sided, with a significant threshold set at a *P* value of <0.05. The absolute risk, absolute risk difference, and risk rates per 100 patient years were determined. Statistical analyses and graphical outputs were produced using Review Manager version 5.4.1 (MedDRA, 2022b) and GRADEpro GDT software (GRADEpro GDT Cochrane Community, 2022).

## 3 Results

**FIGURE 1:**
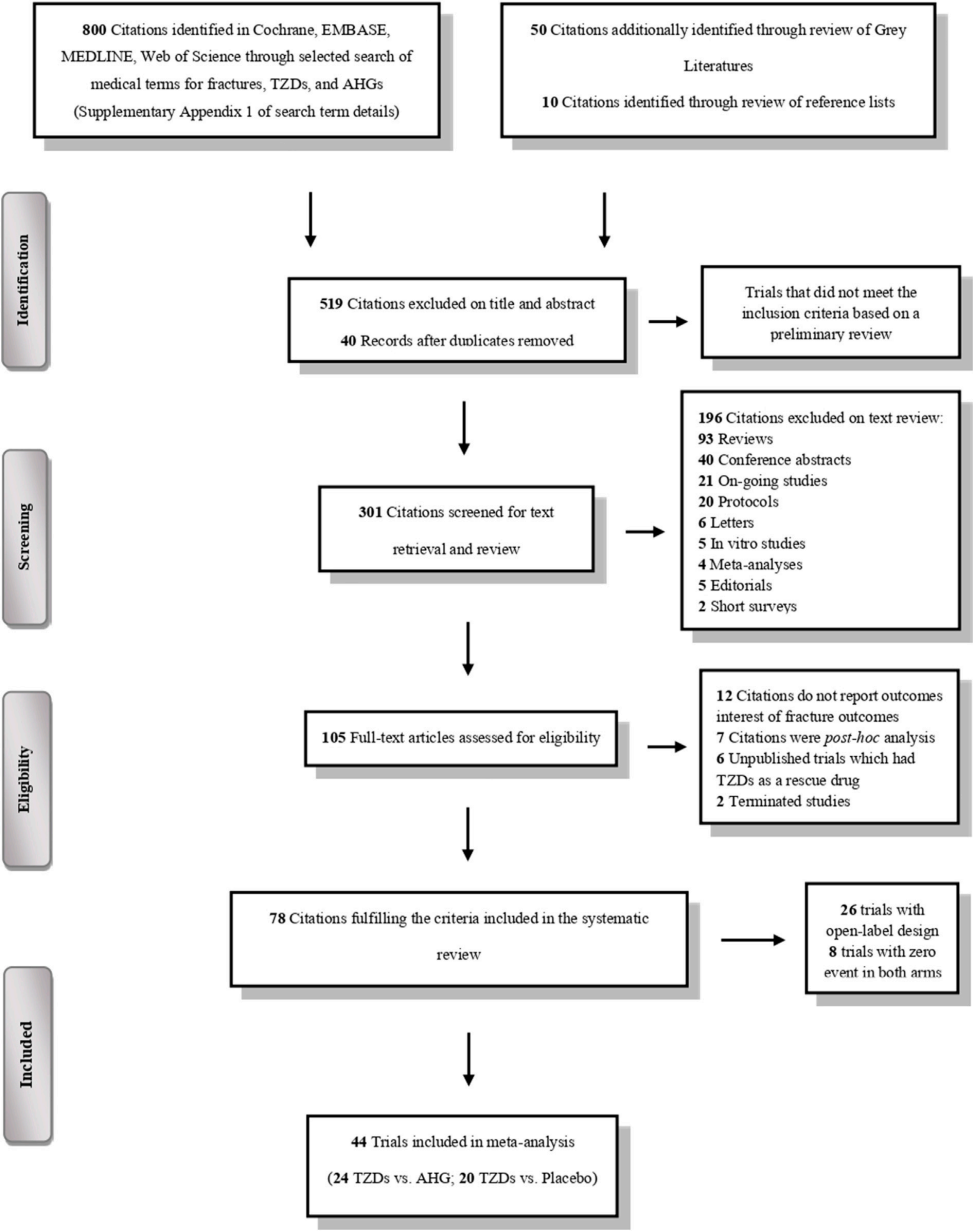
PRISMA flowchart depicting the selection process of the clinical trials included in the systematic review and meta-analysis.

The literature search yielded a total of 860 trials, of which 78 met the predefined eligibility criteria (Figure 1). The key characteristics of these included trials are detailed in Supplementary Table S1.

### 3.1 Study population

All participants included in the meta-analysis were diagnosed with either insulin resistance or T2DM, with 10% being nondiabetic and 90% diabetic. The average age of participants was 72 years, and 58% were male individuals. The mean HbA1c level across the studies was approximately 8.5%. Participants who were administered pioglitazone (or TZDs) had a history of stroke ranging from 2.3% to 18.8%, prior fractures between 3.8% and 15%, and bone disease or low BMD in less than 1% of cases.

### 3.2 Study interventions and exposures

Most trials utilize various comparative dosing regimens, either oral or injectable. For TZD, the administered doses ranged from 15 to 45 mg per day. Additional AHG drugs were used in accordance with their approved dosages as per medical guidelines. In the pioglitazone trials, the median follow-up duration varied from 12 to 261 weeks, with three RCTs having follow-up periods of less than 24 weeks, nine RCTs ranging from 24 to 52 weeks, eight RCTs from 52 to 104 weeks, and seven RCTs extending beyond 104 weeks.

### 3.3 Risk of bias assessment

**FIGURE 2:**
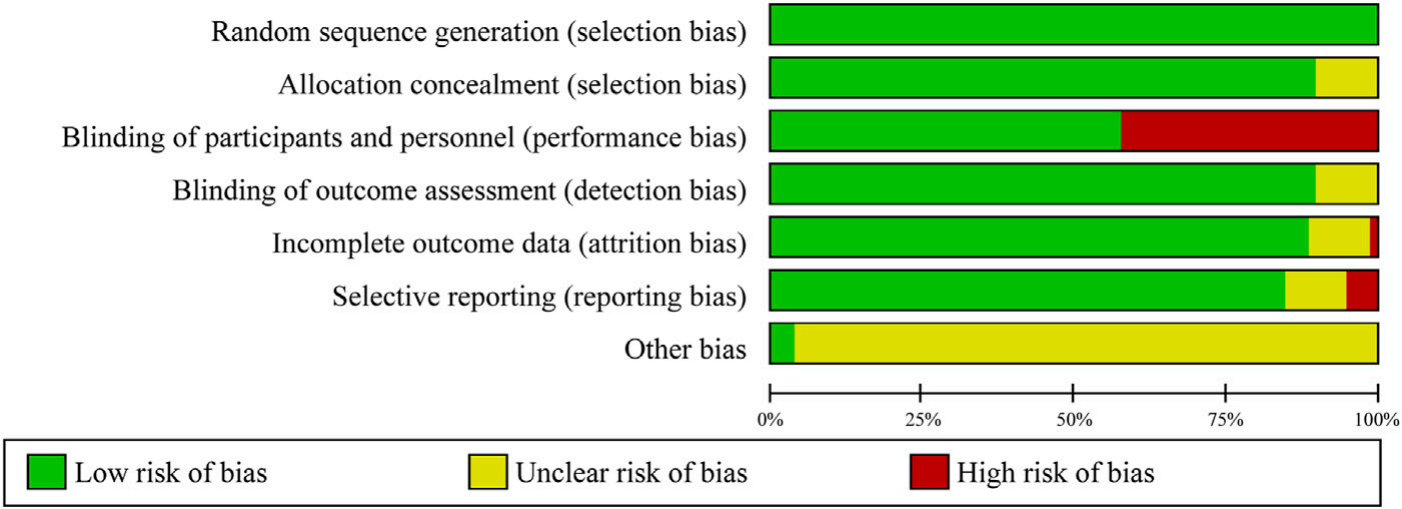
Risk of bias graph: review of authors’ judgments regarding each risk of bias item presented as percentages across all included clinical trials.

The risk of bias assessment in the meta-analysis revealed varying levels of quality across the trials. Out of the trials evaluated, 36 were classified as having a low risk of bias, eight as having an unclear risk, and 34 as having a high risk. Although sufficient descriptive data were available for random sequence generation, 26 trials (26%) used an open-label design with two or more intervention groups, by comparing non-TZD pioglitazone trials with other AHG drugs in both arms. In these trials, participants in both arms were exposed to pioglitazone and other TZD drugs, enabling a direct comparison of pioglitazone’s efficacy and safety against other AHG treatments. However, this design introduced a high risk of performance bias, leading to the exclusion of these trials from the meta-analysis. Additionally, 8% of the trials demonstrated unclear risks of detection and selection bias (Figure 2; Supplementary Figure S1).

### 3.4 Outcome measures

Among the 78 trials assessed, 70 reported fracture events, whereas eight trials recorded no fractures in either study arm. The latter were excluded from our meta-analysis. Most trials primarily focus on the change in HbA1c levels from baseline. However, the PROactive (Dormandy et al., 2009) trial was specifically designed to assess major adverse cardiovascular events (MACE), including stroke, and the IRIS (Kernan et al., 2016) trial targeted fatal or non-fatal stroke as its primary outcomes. Despite this, fracture outcomes were consistently recorded across all 78 trials as part of the safety assessment process, categorized as either serious or non-serious adverse events.

### 3.5 Outcome results

Detailed results of trial outcomes are provided in Supplemental Tables: fracture risk categorized by severity and mechanism (Supplementary Figures S2, S3), anatomical site (Supplementary Figures S4–S10), sex differences (Supplementary Figures S11, S12), and BMD levels (Supplementary Figure S13). Out of the 70 trials assessed, a meta-analysis was performed on 44 trials that reported at least one fracture event, after excluding 26 trials identified as having a high risk of performance bias, Table 1. This analysis included comparisons between TZDs and non-TZDs, comprising 12 trials on rosiglitazone versus non-rosiglitazone, and 27 trials on pioglitazone versus non-pioglitazone. Of the pioglitazone trials, 14 compared it to placebo, while 13 compared it to other AHG drugs.

**TABLE 1:** The overall risk of fracture by severity and mechanism with TZDs drugs and comparators of AHGs class.

Primary and secondary outcomes	Trials	Participants	Mantel-Haenszel fixed effect (RR [95% CI])	Heterogeneity
TZDs and fracture risk: severity and mechanism of fractures				
• Follow-up: range 12–261 weeks	44	86,659	RR 1.35 [1.24, 1.48]^*^	*P* < 0.00001^*^; *I* ^*2*^ = 10%
TZDs – rosiglitazone drug				
Rosiglitazone vs. non-rosiglitazone	12	13,470	RR 1.42 [1.23, 1.64]^*^	*P* < 0.00001^*^; *I* ^*2*^ = 34%
TZDs – pioglitazone drug and fracture risk: comparators				
Pioglitazone vs. non-pioglitazone	27	24,718	RR 1.19 [1.01, 1.40]^*^	*P* = 0.04^*^; *I* ^*2*^ = 23%
• Pioglitazone vs. placebo	14	13,451	RR 1.21 [1.01, 1.45]^*^	*P* = 0.04^*^; *I* ^*2*^ = 32%
• Pioglitazone vs. AHGs	13	11,267	RR 1.08 [0.73, 1.59]	*P* = 0.70; *I* ^*2*^ = 15%
Pioglitazone and fracture risk: severity and mechanism of fractures				
• Non-serious fractures	7	10,794	RR 1.25 [1.03, 1.51]^*^	*P* = 0.02^*^; *I* ^*2*^ = 53%
• Serious fractures	7	6,194	RR 1.48 [1.10, 1.98]^*^	*P* = 0.010^*^; *I* ^*2*^ = 24%
• Low-energy fractures	1	3,876	RR 1.49 [1.20, 1.87]^*^	*P* = 0.0004^*^
• High-energy fractures	4	5,066	RR 1.43 [0.93, 2.20]	*P* = 0.10; *I* ^*2*^ = 0%
• Stress fractures	1	3,876	RR 1.25 [0.49, 3.16]	*P* = 0.64
• Pathological fractures	3	9,270	RR 0.67 [0.24, 1.87]	*P* = 0.44; *I* ^*2*^ = 0%
Pioglitazone and fracture risk: baseline characteristics in clinical trials				
• Non-diabetic–insulin resistance	3	1,165	RR 0.87 [0.43, 1.76]	*P* = 0.69; *I* ^*2*^ = 0%
• Non-diabetic with stroke	1	3,876	RR 1.41 [1.09, 1.83]^*^	*P* = 0.008^*^
• T2DM	17	17,768	RR 1.02 [0.80, 1.30]	*P* = 0.88; *I* ^*2*^ = 34%
• T2DM with CVD	2	1,130	RR 1.44 [0.81, 2.57]	*P* = 0.22; *I* ^*2*^ = 76%
Pioglitazone and fracture risk: skeletal location				
• Spine	3	9,777	RR 2.13 [1.28, 3.55]^*^	*P* = 0.004^*^; *I* ^*2*^ = 66%
• Hip	3	4,697	RR 1.38 [0.84, 2.28]	*P* = 0.20; *I* ^*2*^ = 0%
• Femur	1	3,876	RR 8.99 [0.48, 116.88]	*P* = 0.14
• Lower extremities	3	9,264	RR 1.85 [1.33, 2.56]^*^	*P* = 0.0002^*^; *I* ^*2*^ = 0%
• Upper extremities	2	9,114	RR 1.37 [0.98, 1.93]	*P* = 0.07; *I* ^*2*^ = 61%
• Wrist	1	602	RR 6.91 [0.36, 133.16]	*P* = 0.20
• Ankle	5	1,717	RR 0.63 [0.21, 1.90]	*P* = 0.41; *I* ^*2*^ = 0%
Pioglitazone and fracture risk: sex differences				
• Females	8	5,315	RR 1.56 [1.20, 2.02]^*^	*P* = 0.0008^*^; *I* ^*2*^ = 0%
• Males	6	8,149	RR 1.10 [0.84, 1.43]	*P* = 0.49; *I* ^*2*^ = 14%
Pioglitazone and fracture risk: follow-up duration				
• ≤ 24 weeks	3	1,231	RR 1.06 [0.21, 5.44]	*P* = 0.94; *I* ^*2*^ = 0%
• 24–52 weeks	9	3,598	RR 0.81 [0.35, 1.88]	*P* = 0.63; *I* ^*2*^ = 0%
• > 52–104 weeks	8	6,419	RR 1.00 [0.55, 1.80]	*P* = 1.00; *I* ^*2*^ = 50%
• > 104 weeks	7	13,470	RR 1.23 [1.03, 1.46]^*^	*P* = 0.02^*^; *I* ^*2*^ = 48%
Pioglitazone and fracture risk: treatment dose				
• 30 mg/day	5	3,502	RR 0.68 [0.32, 1.42]	*P* = 0.30; *I* ^*2*^ = 0%
• 45 mg/day	2	1,057	RR 0.33 [0.06, 1.78]	*P* = 0.20; *I* ^*2*^ = 41%
• 15–30 mg/day—fixed dose	3	1,675	RR 4.26 [0.96, 18.96]	*P* = 0.06; *I* ^*2*^ = 36%
• 30–45 mg/day—fixed dose	7	2,547	RR 0.62 [0.34, 1.12]	*P* = 0.11; *I* ^*2*^ = 42%
• 15–45 mg/day—fixed dose	10	7,638	RR 1.29 [1.08, 1.54]^*^	*P* = 0.005^*^; *I* ^*2*^ = 0%
Pioglitazone and fracture risk: risk of bias in clinical trials				
• Low risk of bias clinical trials	20	19,411	RR 1.23 [1.02, 1.48]^*^	*P* = 0.03^*^; *I* ^*2*^ = 40%
• Unclear risk of bias clinical trials	2	1,826	RR 1.19 [0.22, 6.55]	*P* = 0.84^*^; *I* ^*2*^ = 0%
• High risk of bias clinical trials	5	3,481	RR 1.05 [0.57, 1.96]	*P* = 0.87; *I* ^*2*^ = 0%
Pioglitazone and fracture risk: stratification by individual subclasses of AHG comparators				
• Metformin	3	1,756	RR 0.32 [0.06, 1.77]	*P* = 0.19; *I* ^*2*^ = 0%
• Sulfonylurea	5	3,975	RR 1.27 [0.81, 2.01]	*P* = 0.30; *I* ^*2*^ = 20%
• Metformin + sulfonylurea—fixed dose	1	420	RR 0.71 [0.03, 17.33]	*P* = 0.84

*Statistical analysis tests were two-sided using Review Manager software, demonstrating a significant relative risk difference between groups, with a significant threshold set at *P* < 0.05.

In the analysis of 44 trials involving TZDs (86,659 participants; 1,915 fractures), the relative risk of fractures was found to be 35% higher with TZD use compared to non-TZDs. Specifically, TZD use was found associated with an increased fracture incidence (RR 1.35; 95% CI 1.24–1.48; *P* < 0.00001), as revealed by both fixed- and random-effects models (Supplementary Figures S3, S4).

**FIGURE 3:**
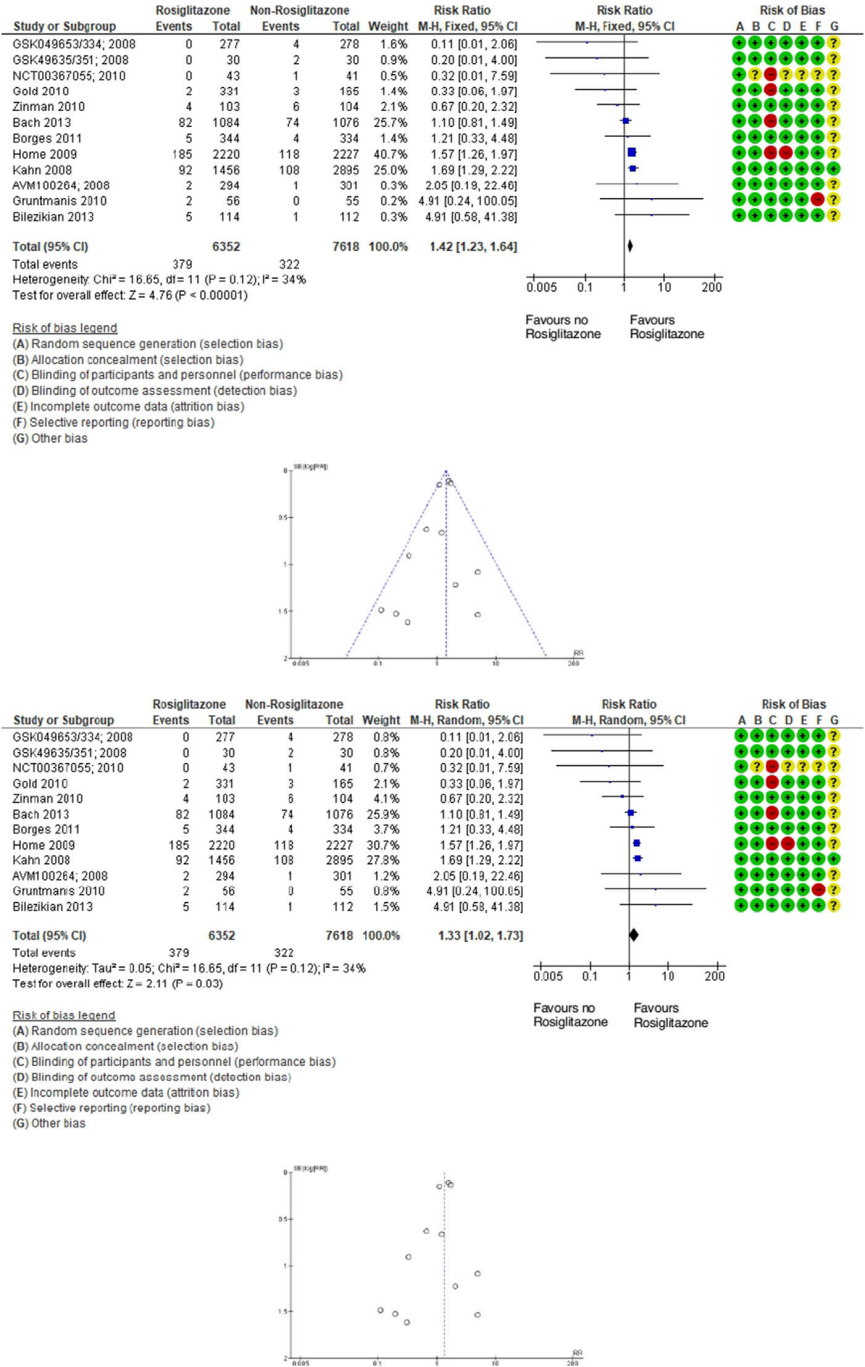
Forest and funnel plot of rosiglitazone and fracture versus placebo, fixed-, and random-effect models.

Similarly, in the 12 trials evaluating rosiglitazone (13,470 participants; 701 fractures), the relative risk of fractures was a 42% increase with rosiglitazone compared to non-rosiglitazone comparators. Rosiglitazone (TZD) use was associated with a significantly increased incidence of fractures (RR 1.42; 95% CI 1.23–1.64; *P* < 0.00001), as assessed using fixed- and random-effects models (Figure 3).

### 3.6 Pioglitazone

**FIGURE 4:**
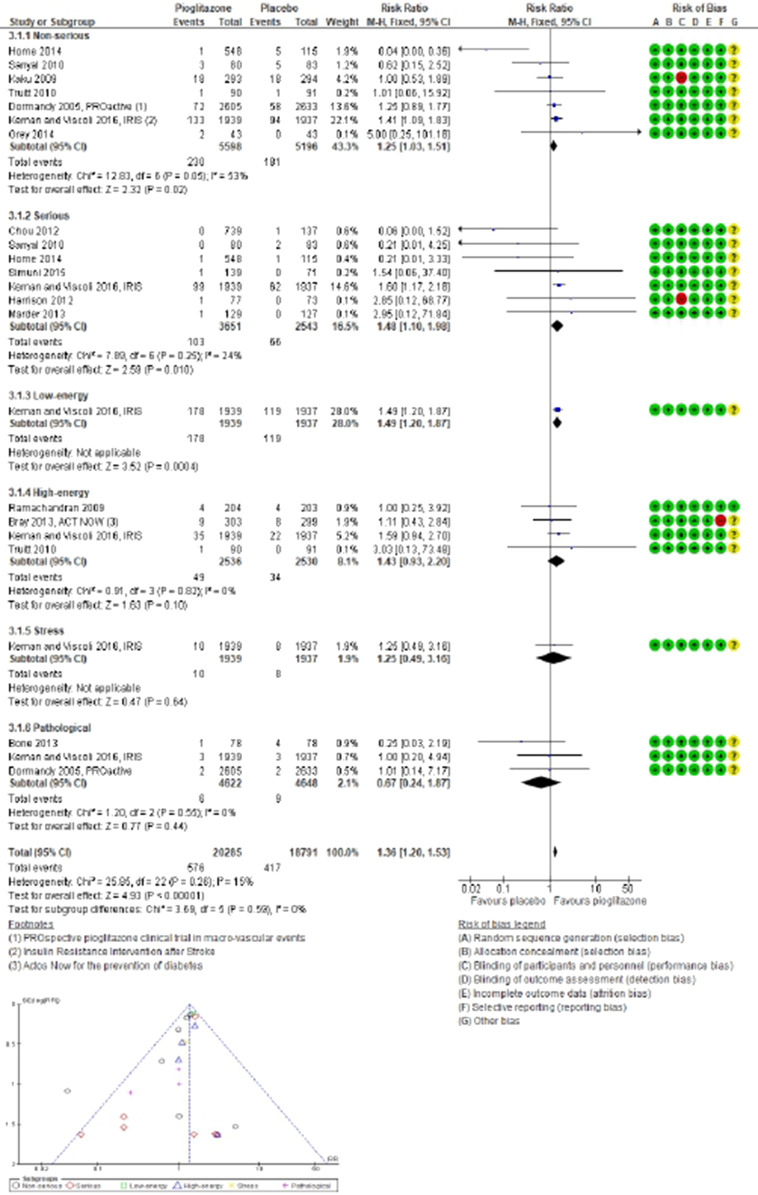
Forest and funnel plot of pioglitazone and fracture versus no-pioglitazone, fixed-effect model.

In the analysis of 27 trials involving TZD pioglitazone (24,718 participants; 548 fractures), the incidence risk of fractures increased by 19% with pioglitazone compared to non-pioglitazone (RR 1.19; 95% CI 1.01–1.40; *P* = 0.04), as assessed using both fixed-effect and random-effects models (Figure 4; Supplementary Figures S5–S8).

**FIGURE 5:**
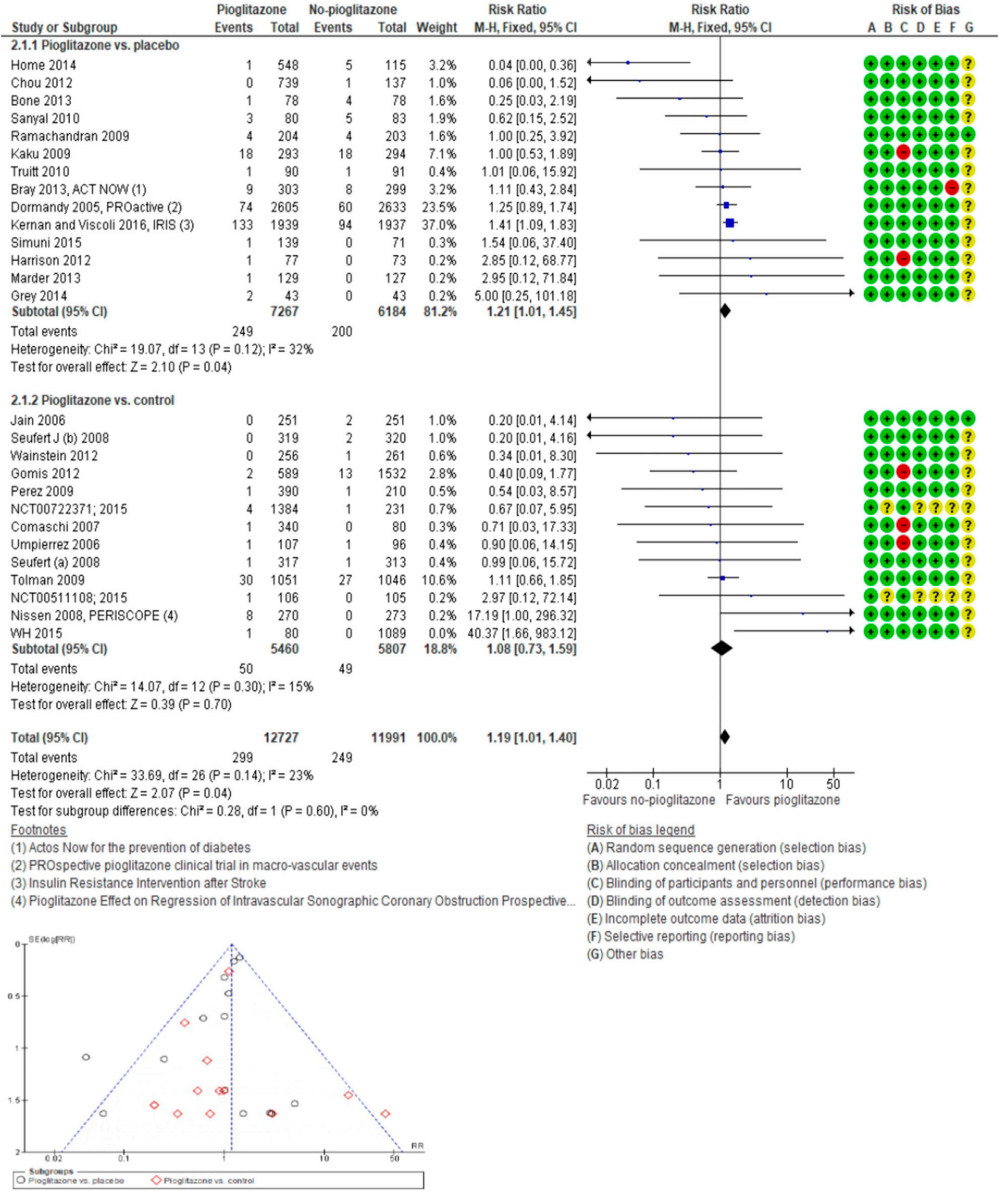
Forest and funnel plot of pioglitazone and fracture subtype by severity and mechanism versus placebo, fixed-effect model.

In 14 trials comparing pioglitazone with placebo (13,451 participants; 449 fractures), pioglitazone use was found associated with a significant increase in fracture incidence (RR 1.21; 95% CI 1.01–1.45; *P* = 0.04). This increase was observed for both non-serious (RR 1.25; 95% CI 1.03–1.51; *P* = 0.02) and serious fractures (RR 1.48; 95% CI 1.10–1.98; *P* = 0.01), particularly low-energy fractures from falls (RR 1.49; 95% CI 1.20–1.87; *P* = 0.0004). However, no significant increase in the risk of high-energy (RR 1.43; 95% CI 0.93–2.20; *P* = 0.10), stress-related (RR 1.25; 95% CI 0.49–3.16; *P* = 0.64), or pathological fractures (RR 0.67; 95% CI 0.24–1.87; *P* = 0.44) was observed with pioglitazone compared to placebo, Figure 5.

Pioglitazone use was associated with a higher incidence risk of fractures in nondiabetic individuals (RR 0.87; 95% CI 0.43–1.76; *P* = 0.69), especially those with a history of stroke (RR 1.41; 95% CI 1.09–1.83; *P* = 0.008). Conversely, pioglitazone did not significantly increase fracture risk in patients with T2DM (RR 1.02; 95% CI 0.08–1.30; *P* = 0.88) or in those at risk or not at risk for CVD (RR 1.44; 95% CI 0.81–2.57; *P* = 0.22) compared to placebo.

The study results also elucidated that pioglitazone use led to a significant increase in the risk of fractures in the lower extremities (RR 1.85; 95% CI 1.33–2.56; *P* = 0.0002) and spine (RR 2.13; 95% CI 1.28–3.55; *P* = 0.004), with a more pronounced risk observed in female individuals (RR 1.56; 95% CI 1.20–2.02; *P* = 0.0008) compared to male individuals (RR 1.10; 95% CI 0.84–1.43; *P* = 0.49). In three trials, use of pioglitazone was found associated with decreased SMD in BMD, with the most significant reductions observed in the lumbar spine (SMD -0.18; 95% CI -0.34 to −0.03; *P* = 0.02) and hip (SMD -0.53; 95% CI -0.96 to −0.10; *P* = 0.02), but not in the femoral neck (SMD -0.25; 95% CI -0.61 to 0.11; *P* = 0.17) compared to placebo.

In 13 trials comparing pioglitazone to other AHGs (11,267 participants; 99 fractures), pioglitazone use could not significantly increase the incidence risk of fractures (RR 1.08; 95% CI 0.73–1.59; *P* = 0.70). There was no statistically significant difference between the risk of non-serious (RR 1.28; 95% CI 0.81–2.01; *P* = 0.29) and serious fractures (RR 0.71; 95% CI 0.32–1.55; *P* = 0.38) or high-energy fractures (RR 0.18; 95% CI 0.01–4.40; *P* = 0.29) and the use of pioglitazone and other AHGs. The fracture risk did not significantly differ between female (RR 1.55; 95% CI 0.84–2.86; *P* = 0.16) or male individuals (RR 0.95; 95% CI 0.49–1.82; *P* = 0.87) when comparing pioglitazone to other AHGs.

Detailed meta-analysis results on fracture risk by follow-up duration (Supplementary Figures S9, S10), clinical trial baseline characteristics (Supplementary Figures S11–S14), skeletal location (Supplementary Figures S15, S16), sex differences (Supplementary Figures S17–S20), severity and mechanism of fractures (Supplementary Figures S21–S24), BMD (Supplementary Figures S25, S26), stratification by individual subclasses of AHGs comparators (Supplementary Figures S27, S28), clinical trial risk of bias (Supplementary Figures S29, S30), and treatment dose of pioglitazone (Supplementary Figures S31, S32) are comprehensively detailed in the Supplementary Appendix 1.

## 4 Discussion

The increased fracture risk associated with TZD use, particularly pioglitazone, remains a key concern; this sometimes makes clinicians hesitant to prescribe TZDs after an individual suffers a stroke. This meta-analysis aims to elucidate the fracture risk associated with pioglitazone and present a clearer perspective on its risk-benefit profile for clinical decision-making and future research. Pioglitazone was selected for this analysis because other TZD drugs stand withdrawn from the market due to adverse effects (Wright et al., 2014). The findings reveal an elevated risk of both non-serious and serious fractures, especially low-energy fractures from falls, in non-diabetic stroke survivors treated with pioglitazone compared to placebo. However, the increased fracture risk might be influenced by unmonitored events or confounding factors, including diabetes severity, pre-existing fracture risks, falls, sex, or concomitant AHG drugs.

Diabetes itself is a notable risk factor for stroke and contributes to macrovascular and microvascular complications (Vinik and Ziegler, 2007), such as orthostatic hypotension, peripheral neuropathy, and limb deformities (Tilling et al., 2006). Hyperglycemia exacerbates fracture susceptibility (McNair et al., 2005) by impairing bone turnover, thereby altering collagen glycosylation, and inducing osteodystrophy (Hofbauer et al., 2007). Pioglitazone has been linked to higher fracture risk, particularly in insulin-resistant post-stroke individuals, though pioglitazone’s impact on fracture risk in patients with T2DM appears minimal, regardless of cardiovascular risk (Spence et al., 2019). This suggests that fracture risk may be more closely associated with insulin resistance and post-stroke conditions rather than T2DM alone. Given pioglitazone’s therapeutic benefits, such as reducing stroke recurrence and improving lipid profiles, it is crucial to carefully weigh these advantages against fracture risks in specific subgroups.

This analysis highlights that fractures associated with pioglitazone use predominantly involve the spine and lower limbs. These fractures are typically low-energy and non-serious, especially in insulin-resistant stroke survivors (Viscoli et al., 2017) and T2DM patients with high cardiovascular risk (Dormandy et al., 2009). The increased fracture risk in these populations may be influenced by some stroke-related biomechanical vulnerabilities (Seref-Ferlengez et al., 2015), such as skeletal impairments, peripheral extremity weakness, and diminished ability to protect against falls, which collectively heighten vulnerability to spinal and lower limb fractures. Another contributing factor may be the enhanced glycemic control achieved with pioglitazone, especially in combination with metformin. Although it is a therapeutic goal, improved glycemic management may inadvertently elevate the risk of hypoglycemia—a key cause that may lead to falls (Monami et al., 2014). Frequent hypoglycemic episodes, often associated with stricter glycemic targets, suggest that fracture risk may not solely stem from pioglitazone’s direct effects on bone health but rather from the interplay between improved glycemic control and its adverse consequences. These findings underscore the need for a comprehensive and balanced evaluation of pioglitazone’s clinical utility. Although its cardiovascular and stroke prevention benefits are proven, the potential fracture risks in at-risk populations, particularly stroke survivors and insulin-resistant individuals, necessitate careful consideration in individuals on pioglitazone.

Gender differences further compound the fracture risk profile. Stroke incidence is higher in male subjects, female subjects, esp. postmenopausal, face greater fracture risks related to severity (Appelros et al., 2009) due to bone density loss associated with reduced estrogen levels (Yu et al., 2020). This meta-analysis reported that pioglitazone-related fractures were more frequent in women, and corroborated the findings of the IRIS trial (Viscoli et al., 2017) that associated these fractures with falls. Non-serious adverse events were also found more common in women, whereas men showed higher incidences of serious adverse events. These differences highlight the need for sex-specific evaluations of pioglitazone’s safety and efficacy. It is suggested that future research should focus on long-term, real-life studies to better understand gender-specific responses and inform individualized treatment approaches that balance cardiovascular and skeletal health.

The mechanisms underlying pioglitazone-induced fracture risk remain poorly understood. Results of the experimental models suggest that TZDs promote bone resorption and reduce bone formation, thereby accelerating bone loss (Lecka-Czernik et al., 2002). In this meta-analysis, minimal decreases in BMD were documented at the lumbar spine and hip with pioglitazone use, though not at the femoral neck. Though these reductions are less than the average annual BMD loss in postmenopausal women, (Hannan et al., 2000), cumulative effects over extended use could become clinically significant. As only a few trials have examined BMD and fracture risk in diabetic (Grey et al., 2013) and insulin-resistant patients, (Bone et al., 2013; Bray et al., 2013), evaluating the long-term skeletal impact of pioglitazone remains a critical safety concern post-stroke.

Despite the fracture risks associated with pioglitazone, its cardiovascular benefits, including the prevention of stroke and myocardial infarction, remain clinically significant. Comparisons with other AHG drugs, such as metformin and sulfonylureas, highlight distinct risk profiles. For example, metformin demonstrates protective effects on bone by inhibiting PPAR-γ, thereby promoting bone formation and reducing resorption (Feng et al., 2021), the use of sulfonylureas is associated with an increased risk of falls and fractures due to hypoglycemia (Zhang et al., 2020). Notably, this meta-analysis showed no statistically significant differences in fracture rates between users of pioglitazone and the other AHG drugs. To optimize therapeutic outcomes, combining pioglitazone with bone-protective therapies or metformin may effectively mitigate fracture risks while preserving its substantial cardiovascular benefits.

The current research employed both fixed- and random-effects models. More significant increases in fracture risk were observed under the fixed-effect model for certain subgroups, such as women, low-energy fractures, and fractures in lower limbs, compared to those under the random-effects model, thereby suggesting variability in subgroup-specific risks. Moderate to high-quality evidence, as assessed by GRADE scores, supports an increased fracture risk with pioglitazone in women, though findings are not completely validated when stratified by skeletal site or BMD values. Future studies should address these limitations, including variability in fracture reporting, to refine fracture risk assessments.

Stroke’s debilitating consequences are often considered worse than death (Pratt et al., 2019), underscoring the importance of preventing stroke recurrence. Although the observed fracture risks with pioglitazone are modest, they remain clinically relevant. Current data suggest that treating 100 patients with pioglitazone for 5 years may result in one additional non-serious fracture and up to three serious fractures. These risks must be carefully weighed against the prevention of recurrent strokes or myocardial infarctions in post-stroke patients. A comprehensive, multifaceted approach—incorporating genetics (Meschia et al., 2007) and pharmacological factors—is essential to optimize therapeutic strategies and improve outcomes in both cardiovascular and skeletal health.

The contrasting effects of TZDs, such as rosiglitazone and pioglitazone, further highlight the need for tailored approaches in managing T2DM patients. Both TZDs exert their glucose-lowering effects through PPAR-γ activation, (Kim et al., 2012), which enhances insulin sensitivity. However, the cardiovascular and skeletal impacts of these TZDs differ substantially. The use of Rosiglitazone has been associated with an increased risk of myocardial infarction (Nissen and Wolski, 2007), attributed to its adverse influence on lipid profiles, including a rise in low-density lipoprotein (LDL) cholesterol levels. This lipid imbalance potentially accelerates atherogenesis and elevates cardiovascular risk (Lincoff et al., 2007). In contrast, pioglitazone improves lipid profiles, reducing LDL cholesterol while increasing high-density lipoprotein cholesterol. These favorable effects underpin pioglitazone’s cardioprotective properties, (Lincoff et al., 2007), including reduced stroke incidence and MACEs in high-risk populations. Pioglitazone’s substantial cardiovascular benefits must be balanced against its fracture risks, particularly in vulnerable subgroups such as postmenopausal women (Ma et al., 2012) and stroke survivors. Expanding research to include larger and more diverse cohorts, alongside comparative studies with other AHG drugs, will provide a clearer understanding of pioglitazone’s safety profile in future studies. Personalized treatment strategies for post-stroke diabetes management (Hewitt et al., 2024), addressing both metabolic and skeletal health and exploring adjunctive treatments like bone-protective agents or lifestyle modifications, (Azhari et al., 2024) are essential to ensure the safe and effective use of pioglitazone in clinical practice.

### 4.1 Strength and limitations

Previous meta-analyses examining pioglitazone’s fracture risk in patients with T2DM have yielded inconsistent findings. Some studies (Zhu et al., 2014; Loke et al., 2009) reported an increased fracture risk in women, with significant reductions in BMD at the lumbar spine, femoral neck, and hip, irrespective of age or cumulative exposure. However, one study (Pavlova et al., 2018) reported no significant differences in fracture risk across genders or exposure durations. These discrepancies underscore the need for a more nuanced investigation. This meta-analysis seeks to bridge these gaps by evaluating pioglitazone’s impact on fracture risk compared to other AHG drugs, analyzing mechanisms, severity, and skeletal site-specific risks while accounting for factors like gender, insulin resistance, T2DM, and CVD.

The included trials offered diverse fracture outcomes but lacked uniformity in reporting key variables, such as stroke status, fracture specifics, and risk factors. For instance, while the PROactive (Dormandy et al., 2009) trial involved T2DM patients post-stroke, it did not provide clear fracture data, limiting its applicability to findings from the IRIS (Kernan et al., 2016) trial. Additionally, inconsistencies in fracture definitions—encompassing broad categories like leg/hip, hip/femur, foot/ankle, or hand/wrist fractures and multiple fractures in a single individual—complicated accurate interpretation. Given that fractures were primarily assessed as safety outcomes, crucial details such as fracture-related disability, mortality, fall incidence, follow-up duration, and baseline bone markers were inconsistently reported, further impeding precise risk assessment.

Current evidence indicates an increased fracture risk with pioglitazone use in non-diabetic post-stroke patients; however, the limited number of studies in this population calls for cautious interpretation of evidence. Expanding research to include larger, more diverse cohorts of non-diabetic post-stroke individuals could enhance the reliability of these findings. Furthermore, it remains unclear whether pioglitazone’s fracture risk stems directly from its effects or from neurological disabilities post-stroke, as trials to date have primarily compared pioglitazone to placebo. It may be noted that conducting cross-sectional and comparative studies involving other AHGs, such as metformin, and larger population are critical to understanding relative fracture risks. To guide clinical decision-making, personalized treatment strategies are essential, balancing pioglitazone’s fracture risk against its cardiovascular benefits, particularly in high-risk groups. Future research should prioritize comprehensive safety evaluations, including standardized fracture incidence reporting, BMD changes, and long-term follow-up, to inform clinical guidelines and optimize the safe use of pioglitazone in diverse patient populations.

### 4.2 Conclusion

Although the use of pioglitazone demonstrates substantial cardiovascular benefits, including reduced stroke and MACEs, its associated fracture risk—particularly in postmenopausal female individuals and those with pre-existing skeletal conditions—remains a significant concern. Current evidence suggests that the increased fracture risk is notable primarily in specific subgroups and may be influenced by the interplay between improved glycemic control and hypoglycemia-related falls. Future comparative studies between pioglitazone and other AHG drugs, alongside comprehensive safety evaluations, are essential to refine risk profiles and optimize treatment strategies. Clinicians should adopt personalized approaches that balance pioglitazone’s cardiovascular advantages with its potential adverse effects on bone health, ensuring informed and effective management of patients with T2DM. The results may guide appropriate treatment decisions regarding the use of pioglitazone in selected individuals and inform future post-stroke trial designs.

## Data Availability

The original contributions presented in the study are included in the article/Supplementary Material; further inquiries can be directed to the corresponding author.

## Abbreviations

AHGs, antihyperglycemic active comparators; TZDs, thiazolidinediones; T2DM, type 2 diabetes mellitus; CVD, cardiovascular disease; RR, relative risk; CI, confidence interval.
