# Supplementary material for: Clinical implications of fracture severity risk with pioglitazone: a systematic review and meta-analysis of clinical randomized trials
Source: Front Pharmacol. 2025 Mar 6;16:1357309. doi: 10.3389/fphar.2025.1357309 (PMC11922898; doi:10.3389/fphar.2025.1357309)
Supplement: Supplementary file 1 [file DataSheet1.docx]

Supplementary Material

**Table of contents**

[Supplementary Appendix 1 of search term details 9](#_Toc79439171)

[Cochrane Central Register of Controlled Trials (CENTRAL) 9](#_Toc79439172)

[MEDLINE search strategy (Ovid) 10](#_Toc79439173)

[EMBASE search strategy 12](#_Toc79439174)

[PubMed search strategy 14](#_Toc79439175)

[Web of Science search strategy 15](#_Toc79439176)

[Risk of bias outcomes 16](#_Toc79439177)

[Trial baseline characteristic outcomes 17](#_Toc79439178)

[Fracture outcome results 25](#_Toc79439179)

[Meta-analysis and GRADE outcomes 56](#_Toc79439180)

[TZDs and fracture risk by severity and mechanism 56](#_Toc79439181)

[GRADE evidence 60](#_Toc79439182)

[Pioglitazone and fracture risk by comparator 63](#_Toc79439183)

[GRADE evidence 64](#_Toc79439184)

[Pioglitazone and fracture by severity, mechanism, and comparators 66](#_Toc79439185)

[GRADE evidence 70](#_Toc79439186)

[Pioglitazone and fracture by follow-up duration 74](#_Toc79439187)

[GRADE evidence 77](#_Toc79439188)

[Pioglitazone and fracture by baseline characteristics 80](#_Toc79439189)

[GRADE evidence 85](#_Toc79439190)

[Pioglitazone and fracture by skeletal location and comparators 88](#_Toc79439191)

[GRADE evidence 93](#_Toc79439192)

[Pioglitazone and fracture by six difference and comparators 97](#_Toc79439193)

[GRADE evidence 101](#_Toc79439194)

[Pioglitazone and fracture by six, comparator, severity, and mechanism 103](#_Toc79439195)

[GRADE evidence 109](#_Toc79439196)

[Pioglitazone and fracture by bone mineral density and skeletal location 113](#_Toc79439197)

[GRADE evidence 115](#_Toc79439198)

[Pioglitazone and fracture by AHGs subclass 118](#_Toc79439199)

[GRADE evidence 120](#_Toc79439200)

[Pioglitazone and fracture by risk of bias and comparators 123](#_Toc79439201)

[GRADE evidence 126](#_Toc79439202)

[Pioglitazone and fracture by treatment dose 128](#_Toc79439203)

[GRADE evidence 131](#_Toc79439204)

[References 133](#_Toc79439205)

**Table of Figures**

[Figure S1. Risk of bias outcomes in individuals’ trials included in the systematic review 16](#_Toc81327707)

[Figure S2. Forest and funnel plot of TZDs and fracture by severity and mechanism, Fixed-effect model. 57](#_Toc81327708)

[Figure S3. Forest and funnel plot of TZDs and fracture by severity and mechanism, Random-effect model. 58](#_Toc81327709)

[Figure ‎S4. Forest and funnel plot of pioglitazone and fracture by comparator, Random-effect model. 63](#_Toc81327710)

[Figure S5. Forest and funnel plot of pioglitazone and fracture by severity and mechanism versus placebo, Random-effect model. 66](#_Toc81327711)

[Figure S6. Forest and funnel plot of pioglitazone and fracture by severity and mechanism versus control, Fixed-effect model. 67](#_Toc81327712)

[Figure S7. Forest and funnel plot of pioglitazone and fracture by severity and mechanism versus control, Random-effect model. 68](#_Toc81327713)

[Figure S8. Forest and funnel plot of pioglitazone and fracture by follow-up duration, Fixed-effect model. 75](#_Toc81327714)

[Figure S9. Forest and funnel plot of pioglitazone and fracture by follow-up duration, Random-effect model. 76](#_Toc81327715)

[Figure S10. Forest and funnel plot of pioglitazone and fracture in nondiabetic individuals, Fixed-effect model. 80](#_Toc81327716)

[Figure S11. Forest and funnel plot of pioglitazone and fracture in nondiabetic individuals, Random-effect model. 81](#_Toc81327717)

[Figure S12. Forest and funnel plot of pioglitazone and fracture in patients with T2DM, Fixed-effect model. 82](#_Toc81327718)

[Figure S13. Forest and funnel plot of pioglitazone and fracture in patients with T2DM, Random-effect model. 83](#_Toc81327719)

[Figure S14. Forest and funnel plot of pioglitazone and fracture by skeletal location and comparators, Fixed-effect model. 89](#_Toc81327720)

[Figure S15. Forest and funnel plot of pioglitazone and fracture by skeletal location and comparators, Random-effect model. 90](#_Toc81327721)

[Figure S16. Forest and funnel plot of pioglitazone and fracture in females versus comparator, Fixed-effect model. 98](#_Toc81327722)

[Figure S17. Forest and funnel plot of pioglitazone and fracture in females versus comparator, Random-effect model. 98](#_Toc81327723)

[Figure S18. Forest and funnel plot of pioglitazone and fracture in males versus comparator, Fixed-effect model. 99](#_Toc81327724)

[Figure S19. Forest and funnel plot of pioglitazone and fracture in males versus comparator, Random-effect model. 99](#_Toc81327725)

[Figure S20. Forest and funnel plot of pioglitazone and fracture in females by comparator, severity, and mechanism, Fixed-effect model. 104](#_Toc81327726)

[Figure S21. Forest and funnel plot of pioglitazone and fracture in females by comparator, severity, and mechanism, Random-effect model. 105](#_Toc81327727)

[Figure S22. Forest and funnel plot of pioglitazone and fracture in males by comparator, severity, and mechanism, Fixed-effect model. 106](#_Toc81327728)

[Figure S23. Forest and funnel plot of pioglitazone and fracture in males by comparator, severity, and mechanism, Random-effect model. 107](#_Toc81327729)

[Figure S24. Forest and funnel plot of pioglitazone and fracture by bone mineral density versus comparator, Fixed-effect model. 114](#_Toc81327730)

[Figure S25. Forest and funnel plot of pioglitazone and fracture by bone mineral density versus comparator, Random-effect model. 114](#_Toc81327731)

[Figure S26. Forest and funnel plot of pioglitazone and fracture by AHGs subclass, Fixed-effect model. 119](#_Toc81327732)

[Figure S27. Forest and funnel plot of pioglitazone and fracture by AHGs subclass, Random-effect model. 119](#_Toc81327733)

[Figure S28. Forest and funnel plot of pioglitazone and fracture by risk of bias and comparators, Fixed-effect model. 123](#_Toc81327734)

[Figure S29. Forest and funnel plot of pioglitazone and fracture by risk of bias and comparators, Random-effect model. 124](#_Toc81327735)

[Figure S30. Forest and funnel plot of pioglitazone and fracture by treatment dose, Fixed-effect model. 128](#_Toc81327736)

[Figure S31. Forest and funnel plot of pioglitazone and fracture by treatment dose, Random-effect model. 129](#_Toc81327737)

**Table of tables**

[Table S1. Trial baseline characteristics of TZDs 17](#_Toc81327738)

[Table S2. Trial outcomes of adverse event fractures 25](#_Toc81327739)

[Table S3. Trial outcomes of serious adverse event fractures 30](#_Toc81327740)

[Table S4. Trial outcomes of spine fracture 36](#_Toc81327741)

[Table S5. Trial outcomes of hip fracture 38](#_Toc81327742)

[Table S6. Trial outcomes of femur fracture 41](#_Toc81327743)

[Table S7. Trial outcomes of upper-limb fracture 43](#_Toc81327744)

[Table S8. Trial outcomes of wrist fracture 45](#_Toc81327745)

[Table S9. Trial outcomes of lower-limb fracture 46](#_Toc81327746)

[Table S10. Trial outcomes of ankle fracture 48](#_Toc81327747)

[Table S11. Trial outcomes of adverse event fracture by sex difference 51](#_Toc81327748)

[Table S12. Trial outcomes of serious adverse event fracture by sex difference 54](#_Toc81327749)

[Table S13. Trial outcomes of fracture and bone mineral density 55](#_Toc81327750)

[Table S14. GRADE evidence profile of TZDs and fracture by severity and mechanism 59](#_Toc81327751)

[Table S15. GRADE evidence profile of pioglitazone and fracture by comparator 64](#_Toc81327752)

[Table S16. GRADE evidence profile of pioglitazone and fracture by severity, mechanism, and comparators 69](#_Toc81327753)

[Table S17. GRADE evidence profile of pioglitazone and fracture by follow-up duration 77](#_Toc81327754)

[Table S18. GRADE evidence profile of pioglitazone and fracture by baseline characteristics 84](#_Toc81327755)

[Table S19. GRADE evidence profile of pioglitazone fracture by skeletal location and comparators 91](#_Toc81327756)

[Table S20. GRADE evidence profile of pioglitazone and fracture by six difference and comparators 100](#_Toc81327757)

[Table S21. GRADE evidence profile of pioglitazone and fracture by six, comparator, severity, and mechanism 108](#_Toc81327758)

[Table S22. GRADE evidence profile of pioglitazone and fracture by bone mineral density and skeletal allocation 115](#_Toc81327759)

[Table S23. GRADE evidence profile of pioglitazone and fracture by AHGs subclass 120](#_Toc81327760)

[Table S24. GRADE evidence profile of pioglitazone and fracture by risk of bias and comparators 125](#_Toc81327761)

[Table S25. GRADE evidence profile of pioglitazone and fracture by treatment dose 130](#_Toc81327762)

## **Supplementary Appendix 1 of search terms details**

A computer-assisted literature search was performed using the Cochrane Library, Ovid Medline, Embase, PubMed, and Web of Science databases.

### Cochrane Central Register of Controlled Trials (CENTRAL)

#1 (Bone fracture* or bone* or fracture* or fracture risk*):ti,ab

#2 (fracture*):ti,ab

#3 (Fracture* or bone*):ti,ab

#4 (#1 or #2 or #3)

#5 [mh ˆ“PPAR gamma”/AG,DE,ME,PD,TU]

#6 [mh ˆ“Peroxisome Proliferator-Activated Receptors”/] or [mh ˆ“Transcription Factors”/AG,DE,ME,PD,TU]

#7 [mh ˆThiazolidinediones]

#8 ((“peroxisome proliferator-activated receptor gamma” or “PPAR gamma” or “PPAR gamma” or “PPARgamma” or PPARG or NR1C3) near/5 (agonist* or modulator* or stimulat* or stimulant* or activat*)):ti,ab

#9 (thiazolidinedione* or glitazone* or pioglitazone or rosiglitazone or troglitazone or netoglitazone or rivoglitazone or ciglitazone or balaglitazone or darglitazone or edaglitazone or englitazone or lobeglitazone):ti,ab

#10 (#5 or #6 or #7 or #8 or #9)

#11 (#4 and #10)

**CENTRAL search syntax**

The ‘near’ operator defaults to within 6 words.

‘*’ indicates truncation.

### MEDLINE search strategy (Ovid)

1. Bone fracture/ or exp bone/ or exp fracture/ or exp fracture risk/

2. fracture$.ti,ab.

3. exp Fractures, bone/

4. or/1-4

5. PPAR gamma/ag, de, me, pd, tu

6. Peroxisome Proliferator-Activated Receptors/ag, de, me, pd, tu or Transcription Factors/ag, de, me, pd, tu

7. Thiazolidinediones/

8. ((peroxisome proliferator-activated receptor gamma or PPAR gamma or PPAR-gamma or PPARgamma or PPARG or NR1C3) adj5 (agonist$ or modulator$ or stimulat$ or stimulant$ or activat$)).tw.

9. (thiazolidinedione$ or glitazone$ or pioglitazone or rosiglitazone or troglitazone or netoglitazone or rivoglitazone or ciglitazone or balaglitazone or darglitazone or edaglitazone or englitazone or lobeglitazone).tw,nm.

10. 5 or 6 or 7 or 8 or 9

11. Randomized Controlled Trials as Topic/

12. random allocation/

13. Controlled Clinical Trials as Topic/

14. control groups/

15. clinical trials as topic/ or clinical trials, phase i as topic/ or clinical trials, phase ii as topic/ or clinical trials, phase iii as topic/ or clinical trials, phase iv as topic/

16. double-blind method/

17. single-blind method/

18. Placebo/

19. placebo effect/

20. Drug Evaluation/

21. Research Design/

22. randomized controlled trial.pt.

23. controlled clinical trial.pt.

24. (clinical trial or clinical trial phase i or clinical trial phase ii or clinical trial phase iii or clinical trial phase iv).pt.

25. random$.tw.

26. (controlled adj5 (trial$ or stud$)).tw.

27. (clinical$ adj5 trial$).tw.

28. ((control or treatment or experiment$ or intervention) adj5 (group$ or subject$ or patient$)).tw.

29. (quasi-random$ or quasi random$ or pseudo-random$ or pseudo random$).tw.

30. ((singl$ or doubl$ or tripl$ or trebl$) adj5 (blind$ or mask$)).tw.

31. placebo$.tw.

32. controls.tw.

33. (RCT or RCTs).tw. or trial.ti.

34. or/11-33

35. 4 and 10 and 34

36. exp animals/ not humans.sh.

37. 35 not 36

**Ovid search syntax**

.pt. denotes a Publication Type term;

.ab. denotes a word in the abstract;

.fs. denotes a ‘floating’ subheading;

.sh. denotes a Medical Subject Heading (MeSH) term;

.ti. denotes a word in the title;

The ‘adj6’ operator indicates within 6 words;

.mp. indicates a search of title, original title, abstract, name of substance word and subject heading word;

‘$’ the dollar sign ($) stands for any character(s) indicates truncation;

tw. text word.

### EMBASE search strategy

1. Bone fracture$.tw. or bone$.tw. or fracture$.tw. or fracture risk$.tw.

2. fracture$.tw.

3. Fractures$.tw., bone$.tw.

4. 1 or 2 or 3

5. PPAR gamma/ag, de, me, pd, tu

6. Peroxisome Proliferator-Activated Receptors/ag, de, me, pd, tu or Transcription Factors/ag, de, me, pd, tu

7. Thiazolidinediones/

8. ((peroxisome proliferator-activated receptor gamma or PPAR gamma or PPAR-gamma or PPARgamma or PPARG or NR1C3) adj5 (agonist$ or modulator$ or stimulat$ or stimulant$ or activat$)).tw.

9. (thiazolidinedione$ or glitazone$ or pioglitazone or rosiglitazone or troglitazone or netoglitazone or rivoglitazone or ciglitazone or balaglitazone or darglitazone or edaglitazone or e nglitazone or lobeglitazone).tw,nm.

10. 5 or 6 or 7 or 8 or 9

11. Randomized Controlled Trials as Topic/

12. random allocation/

13. Controlled Clinical Trials as Topic/

14. control groups/

15. clinical trials as topic/ or clinical trials, phase i as topic/ or clinical trials, phase ii as topic/ or clinical trials, phase iii as topic/ or clinical trials, phase iv as topic/

16. double-blind method/

17. single-blind method/

18. Placebo/

19. placebo effect/

20. Drug Evaluation/

21. Research Design/

22. randomized controlled trial.pt.

23. controlled clinical trial.pt.

24. (clinical trial or clinical trial phase i or clinical trial phase ii or clinical trial phase iii or clinical trial phase iv).pt.

25. random$.tw.

26. (controlled adj5 (trial$ or stud$)).tw.

27. (clinical$ adj5 trial$).tw.

28. ((control or treatment or experiment$ or intervention) adj5 (group$ or subject$ or patient$)).tw.

29. (quasi-random$ or quasi random$ or pseudo-random$ or pseudo random$).tw.

30. ((singl$ or doubl$ or tripl$ or trebl$) adj5 (blind$ or mask$)).tw.

31. placebo$.tw.

32. controls.tw.

33. (RCT or RCTs).tw. or trial.ti.

34. 11 or 12 or 13 or 14 or 15 or 16 or 17 or 18 or 19 or 20 or 21 or 22 or 23 or 24 or 25 or 26 or 27 or 28 or 29 or 30 or 31 or 32 or 33

35. 4 and 10 and 34

36. exp animals/ not humans.sh.

37. 35 not 36

### PubMed search strategy

#1 (Bone fracture* or bone* or fracture* or fracture risk*):[tiab]

#2 (fracture*):[tiab]

#3 (Fracture* or bone*):[tiab]

#4 (#1 or #2 or #3)

#5 [mh ˆ“PPAR gamma”/AG,DE,ME,PD,TU]

#6 [mh ˆ“Peroxisome Proliferator-Activated Receptors”/AG,DE,ME,PD,TU] or [mh ˆ“Transcription Factors”/AG,DE,ME,PD,TU]

#7 [mh ˆThiazolidinediones]

#8 ((“peroxisome proliferator-activated receptor gamma” or “PPAR gamma” or “PPAR gamma” or “PPARgamma” or PPARG or NR1C3) near/5 (agonist* or modulator* or stimulat* or stimulant* or activat*)):[tiab]

#9 (thiazolidinedione* or glitazone* or pioglitazone or rosiglitazone or troglitazone or netoglitazone or rivoglitazone or ciglitazone or balaglitazone or darglitazone or edaglitazone or englitazone or lobeglitazone): [tiab]

#10 (#5 or #6 or #7 or #8 or #9)

#11 randomized controlled trial [pt]

#12 controlled clinical trial [pt]

#13 randomized [tiab]

#14 placebo [tiab]

#15 clinical trials as topic [mesh: noexp]

#16 randomly [tiab]

#17 drug therapy [sh]

#18 randomly [tiab]

#19 trial [tiab]

#20 groups [tiab]

#21 #12 OR #13 OR #14 OR #15 OR #16 OR #17 OR #18 OR #19 OR #20

#22 animals [mh] NOT humans [mh]

#23 #21 NOT #22

#24 #4 AND #10 AND #23

**PubMed search syntax**

[pt] denotes a Publication Type term;

[tiab] denotes a word in the title or abstract;

[sh] denotes a subheading;

[mh] denotes a Medical Subject Heading (MeSH) term (‘exploded’);

[mesh: noexp] denotes a Medical Subject Heading (MeSH) term (not ‘exploded’);

[ti] denotes a word in the title.

### Web of Science search strategy

Thiazolidinediones AND bone fracture.

Articles identified through Cochrane Library **25**, MEDLINE **132**, EMBASE **291**, PubMed **284**, Web of Science **68** and **50** publications through Grey Literature.

## Risk of bias outcomes

Figure S1. Risk of bias outcomes in individuals’ trials included in the systematic review


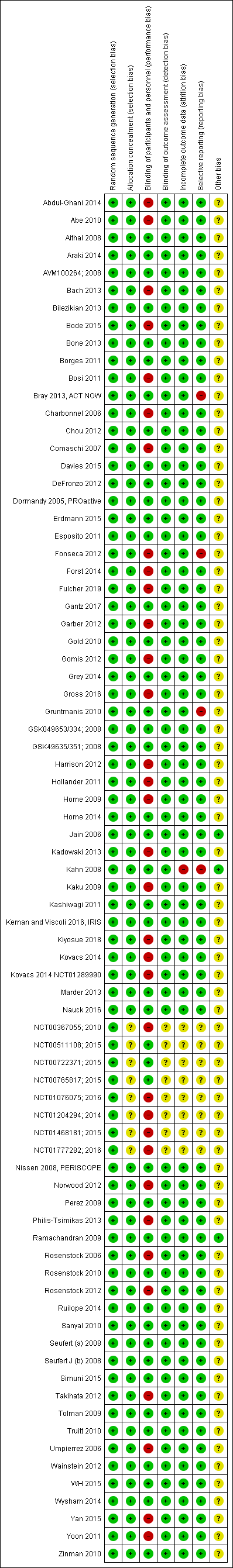


## Trial baseline characteristic outcomes

Table S1. Trial baseline characteristics of TZDs

| Study  Trial registry | Population  Sample size | T2DM† duration (years) | HbA_1C_† levels % | Prior stroke/TIA (%) | Prior fracture/ bone disease % | Mean age (years)^‡^  Males (%) | Study design and country | Parallel arms | Intervention vs. placebo or control | Background AHGs therapy | Intervention of interest dosage^‡^ mg/day  (TZDs therapy) | Duration (weeks) |
| --- | --- | --- | --- | --- | --- | --- | --- | --- | --- | --- | --- | --- |
|  |  | **Intervention of interest (TZDs therapy) vs. placebo or control** | | | | |  |  |  |  |  |  |
| Kernan and Viscoli 2016^1^  NCT00091949 | IR with stroke/TIA (*n*=3876) | IR | IR | 100% | NR | 63.5/63.5  66.7/64.3 | Double-blind, RCT in 179 centers in 7 countries, IRIS trial | 2 | Pioglitazone vs. placebo | None | 15, 30, 45 | 251^**^ |
| Simuni 2015^2^  NCT01280123 | Parkinson’s disease (*n*=210) | IR | IR | NR | NR | 61.3/59.0  72.0/68.0 | Phase II, double-blind RCT in 35 centers in USA, NINDS Exploratory trial | 3 | Pioglitazone vs. placebo | None | 15, 30, 45 | 44 |
| Grey 2014^3^ | T2DM or IGT (*n*=86) | NR | 7.4±3.5/7.5±3.2 | NR | Fracture 5.0/5.0 | 64.0/63.0  49.0/53.0 | Double-blind RCT in Australia | 2 | Pioglitazone vs. placebo | AHG + insulin | 15-30 | 52 |
| Bray 2013^4^  NCT00220961 | IGT (*n*=602) | IGT | 5.0/5.0 | NR | NR | 50.7/48.1  58.0/57.8 | Prospective, double-blind RCT in 8 centers in USA, ACT NOW trial | 2 | Pioglitazone vs. placebo | None | 30-45 | 126 |
| Bone 2013^5^  NCT00708175 | Postmenopausal IGT (*n*=156) | IGT | IGT | NR | Fracture 6.4/3.8 | 59.0/60.2 | Prospective, double-blind RCT in 25 centers in USA | 2 | Pioglitazone vs. placebo | None | 30-45 | 78 |
| Marder 2013^6^  NCT00554853 | Rheumatoid arthritis (*n*=256) | IR | IR | NR | NR | 54.9^**/^54.9^**^  24.0/24.0 | Double-blind, crossover RCT in USA | 2 | Pioglitazone vs. placebo | None | 30-45 | 32 |
| Sanyal 2010^7^  NCT00063622 | Non-alcoholic steatohepatitis (*n*=163) | IR | IR | NR | NR | 47.0/45.4  41.0/42.0 | Phase III, double-blind RCT in USA, PIVENS trial | 2 | Pioglitazone vs. placebo | None | 30 | 96 |
| Ramachandran 2009^8^  NCT00276497 | IGT (*n*=407) | IGT | 5.8±0.4/5.8±0.4 | NR | NR | 45.1/45.5 | Prospective, double-blind RCT in Asia and India, IDPP-2 trial | 2 | Pioglitazone vs. placebo | None | 30 | 157 |
| Aithal 2008^9^  N0192119052 | Non-alcoholic steatohepatitis (*n*=74) | IR | 5.8±0.6/5.9±1.0 | NR | NR | 52.0/55.0  70.0/51.0 | Double-blind RCT in UK | 2 | Pioglitazone vs. placebo | None | 30 | 52 |
| Dormandy 2005^10^  NCT00174993 | T2DM with macrovascular disease (*n*=5238) | 8.0/8.0 | NR | Stroke *n*=985 (18.8% vs. 18.8%)  TIA *n*=299 (5.7% vs. 5.7%) | NR | 61.9/61.6  67.0/66.0 | Prospective, double-blind RCT in 19 European countries, PROactive trial | 2 | Pioglitazone vs. placebo | Metformin ± sulfonylurea ± insulin | 15, 30, 45 | 150 |
| Home 2014^11^ NCT00839527 | T2DM (*n*=663) | 9.2±6.1/8.5±6.3/9.3±6.1 | 8.3±0.8/8.2±0.9/8.3±0.9 | NR | NR | 55.1/55.7  52.0/61.0 | Phase III, double-blind RCT in 234 centers in 9 countries HARMONY 5 | 3 | Pioglitazone + albiglutide vs. placebo | Metformin + glimepiride | 30-45 | 156 |
| Chou 2012^12^  NCT00484198 | T2DM (*n*=1886) | 4.4±4.9/4.6±4.8/4.9±6.1 | 7.7±0.5/7.7±0.5/7.7±0.5 | NR | NR | 55.0/55.1/55.4  53.0/50.0/49.0 | Phase III, double-blind RCT in 254 centers in 4 countries | 4 | Pioglitazone + rivoglitazone vs. placebo | Metformin ± sulfonylurea ± α-glucosidase Is ± meglitinide | Pioglitazone 45  Rivoglitazone 1-1.5 | 26 |
| Truitt 2010^13^  NCT00143520 | T2DM (*n*=438) | 6.6±7.5/6±6.4/6.7±5.6 | 7.9±0.8/8.1±0.9/8.2±0.9 | NR | NR | 56.6/56.4/55.3  58.2/53.9/51.1 | Double-blind RCT in USA | 5 | Pioglitazone + rivoglitazone vs. placebo | Metformin ± sulfonylurea | Pioglitazone 45  Rivoglitazone 1-3 | 26 |
| NCT00722371; 2015^14^ | T2DM (*n*=1615) | NR | NR | NR | NR | 58.9/58.9  56.0/59.0 | Phase III double-blind RCT, factorial study in USA 0431-102-AM2 trial | 7 | Pioglitazone + sitagliptin^¶^ vs. sitagliptin + placebo | Metformin + sulfonylurea | 15, 30, 45 | 54 |
| NCT00511108; 2015^15^ | T2DM (*n*=211) | NR | 7.9±0.9/7.7±0.8/8.0±1.1 | NR | NR | 53.3/54.6/53.3  54.0/54.0/60.0 | Phase I, double-blind RCT in USA, 0431-061 trial | 4 | Pioglitazone + sitagliptin^¶^ vs. sitagliptin + placebo | None | 30 | 24 |
| Kaku 2009^16^  UMIN000001363 | T2DM with CVD (*n*=587) | ≥5.0/≥5.0 | 7.6/7.5 | Stroke  5.3% vs. 5.3% | NR | 58.1/57.9  63.0/62.0 | Prospective, open-label (PROBE) in Japan | 2 | Pioglitazone vs. control | Sulfonylurea ± biguanide ± α-glucosidase Is ± insulin | 15, 30, 45 | 192 |
| Abe 2010^17^ | T2DM with CKD on dialysis (*n*=63) | 16.6±5.5/16.3±5.5 | NR | NR | NR | 65.2/67.2  68.0/68.0 | Prospective, open-label RCT in Japan | 2 | Pioglitazone/conventional AHGs vs. control (AHGs excluding TZDs) | α-glycoside Is  + mitiglinide | 15-30 | 104 |
| Yan 2015^18^  NCT00633282 | T2DM/IGT with non-alcoholic liver disease (*n*=100) | NR | 6.4±0.6/6.1±0.6 | NR | NR | 53.5/50.6 | Open-label RCT in China | 2 | Pioglitazone vs. no-TZDs (lifestyle intervention + berberine) | None | 15 | 16 |
| Harrison 2012^19^ | IR with HCV (*n*=150) | IR | IR | NR | NR | 52.0/51.0^**^  50.0/61.0 | Open-label RCT in USA,  SENSITIZE trial | 2 | Pioglitazone vs. no-TZDs (standard care of peginterferon α-2a + ribavirin) | None | 30-45 | 88 |
| Gold 2010^20^  NCT00428090 | Alzheimer’s disease  (*n*=496) | IR | IR | NR | NR | 71.7/72.5  36.0/40.0 | Phase III, double-blind RCT, in 134 centers in 19 countries | 3 | Rosiglitazone vs. placebo | None | 2-8 | 24 |
| Gruntmanis 2010^21^ | T2DM with CVD (*n*=111) | NR | 7.6±1.8/7.6±1.7 | NR | NR | 56.7/55.8  58.9/58.9 | Prospective, double-blind RCT in USA | 2 | Rosiglitazone vs. placebo | NR  AHG agents | 4-8 | 24 |
| GSK49653/334; 2008^22^  NCT00306644 | T2DM and/or IR (*n*=555) | NR | NR | NR | NR | 67.7/67.3  45.0/48.0 | Phase IV, double-blind RCT in Sweden | 2 | Rosiglitazone vs. placebo | Metformin + sulfonylurea + nateglinide | 4-8 | 52 |
| GSK49635/351; 2008^23^  NCT00231387 | T2DM with CVD or hypertension (*n*=60) | NR | NR | NR | NR | 62.2/65.6  21.0/23.0 | Phase III b, double-blind RCT in UK | 2 | Rosiglitazone vs. placebo | None ± metformin ± sulfonylurea | 4-8 | 52 |
| Zinman 2010^24^  NCT00116932 | IGT (*n*=207) | IGT | IGT | NR | NR | 50.0/55.0^**^  35.0/31.7 | Double-blind RCT in Canada, CANOE trial | 2 | Rosiglitazone/metformin^¶^ vs. placebo | None | 2/500-4/100 | 204 |
| Bach 2013^25^  NCT00006305 | T2DM with CAD (*n*=1360) | 10.8±8.1/10.0±9.1 | 7.8±1.6/7.5±1.6 | 10% vs. 10% | NR | 62.0/62.7  70.0/72.0 | Randomized 2ҳ2 factorial design study in 6 countries, BARI 2D trial | 4 | Rosiglitazone vs. no-TZDs | Metformin + sulfonylurea + insulin + meglitinide | NR | 235 |
| Erdmann 2015^26^  NCT01042769 | T2DM or IR with CVD (*n*=1996) | NR | 8.5/>10 | NR | NR | Pre-DM 66.7/66.6  T2DM 65.8/65.8 | Phase III, double-blind RCT in 812 centers in USA, ALEPREVENT trial | 2 | Aleglitazar vs. placebo | Sulfonylurea ± DPP4-Is ± GLP1-RAs ± biguanide | 0.15 | 235 |
| Esposito 2011^27^ | T2DM (*n*=110) | NR | 8.0±1.0/8.1±1.0 | NR | NR | 54.2/54.2 | Prospective, double-blind RCT in Italy | 2 | Pioglitazone vs. metformin | None | 15, 30, 45 | 24 |
| Seufert (b) 2008^28,29^ | T2DM (*n*=639) | 7±5.6/7.1±5.6 | 8.8±0.9/8.8±0.7 | NR | NR | 60.0/60.0 | Double-blind, RCT in 3 countries | 2 | Pioglitazone vs. metformin | Sulfonylurea | 15, 30, 45 | 52 |
| Perez 2009^30^  NCT00727857 | T2DM (*n*=600) | NR | 8.9±0.1/8.6±0.1 | NR | NR | 54.7/53.7  41.0/47.0 | Phase III b double-blind RCT in 6 countries | 3 | Pioglitazone/  metformin^¶^ vs. metformin | None | 30 | 24 |
| Wainstein 2012^31^  NCT00532935 | T2DM (*n*=517) | 3.3±3.5/3.2±4.0 | 8.9±1.3/ 9.0±1.3 | NR | NR | 52.2/52.4  52.0/55.0 | Phase III, double-blind RCT in USA, 0431A-066 | 2 | Pioglitazone vs. metformin/sitagliptin^¶^ | Insulin | 30-45 | 32 |
| Bilezikian 2013^32^  NCT00679939 | T2DM  Postmenopausal (*n*=226) | 3.9/3.3 | 6.8±0.7/6.8±0.7 | NR | No osteoporosis | 63.6/76.9 | Double-blind RCT in Canada and USA | 2 | Rosiglitazone vs. metformin | Metformin + sulfonylurea | 8 | 76 |
| Borges 2011^33^  NCT00386100 | T2DM (*n*=678) | 2.3±3.1/2.6±3.3 | 8.6±0.9/8.6±0.9 | NR | Fractures 15.0/15.0  Bone disease <1/<1 | 51.5/50.7  53.0/53.0 | Phase IV, double-blind RCT in 98 centers in 9 countries | 2 | Rosiglitazone/metformin^¶^ vs. metformin | None | 4-8 | 80 |
| WH 2015^34^  NCT01217073 | T2DM (*n*=1169) | 5.3±4.4/5.8±4.6 | 8.1±0.9//8.1±0.9 | NR | NR | 55.9/55.9  56.0/57.0 | Phase IIb, double-blind RCT in USA, MK-3102-006 trial | 8 | Pioglitazone/metformin vs. omarigliptin + placebo | Metformin | 15-30 | 78 |
| Tolman 2009^35^  NCT00494312 | T2DM (*n*=2097) | 5.8/5.6 | 9.5±2/9.5±2 | NR | NR | 54.0/55.0^**^  57.2/55.5 | Double-blind RCT in 171 centers in USA | 2 | Pioglitazone vs. glyburide | Metformin ± sulfonylurea | 15, 30, 45 | 144 |
| Jain 2006^36^ | T2DM (*n*=502) | 9.6±13.7/9.4±15.1 | 9.2±1.2/9.2±1.2 | NR | NR | 52.1/52.1  53.0/56.0 | Prospective, double-blind RCT in 65 centers in Puerto Rico or USA | 2 | Pioglitazone vs. glyburide | None | 15, 30, 45 | 56 |
| Nissen 2008^37^  NCT00225277 | T2DM with CHD (*n*=543) | 5.8/5.9 | NR | NR | NR | 60.0/59.7  68.9/65.9 | Prospective, double-blind RCT in 97 centers in USA, PERISCOPE trial | 2 | Pioglitazone vs. glimepiride | None + metformin + sulfonylurea | 15, 30, 45 | 78 |
| Umpierrez 2006^38^ | T2DM (*n*=203) | 5.9±6.1/3.8±4.9 | 8.3±0.7/8.4±0.7 | NR | NR | 55.7/51.6  56.0/53.0 | Open-label RCT in USA | 2 | Pioglitazone vs. glimepiride | Metformin | 30-45 | 28 |
| Seufert (a) 2008^28,39^ | T2DM (*n*=630) | 5.8±5.1/5.5±5.1 | 8.7±1/8.5±0.8 | NR | NR | 56.0/57.0  50.0/49.0 | Double-blind, RCT in 75 centres in 3 countries | 2 | Pioglitazone vs. gliclazide | Metformin | 15, 30, 45 | 52 |
| Comaschi 2007^40^ | T2DM (*n*=420) | NR | 8.5± 0.8/8.6± 0.9 | NR | NR | 59.1/59.1 | Open-label RCT in Italy | 2 | Pioglitazone + sulfonylurea vs. metformin/glibenclamide**^¶^** | Metformin | 15-30 | 24 |
| Home 2009^41^  NCT00379769 | T2DM (*n*=4447) | 6.1/6.3/7.9/7.9 | 7.8/7.8/8/8 | Stroke  2.3/1.8 vs. 2.6/2.9  TIA 2.4/2.3 vs. 2.2/2 | NR | 57/57.2 vs. 59.8/59.7  53.8/52.9 vs. 49/50.6 | Prospective, open-label RCT in 327 centres in Europe and Australasia, RECORD trial | 4 | Rosiglitazone vs. metformin or sulfonylurea | Metformin + sulfonylurea | 4-8 | 261 |
| Kahn 2008^42^  NCT00279045 | T2DM (*n*=4351) | 3.2/3.9/3.6 | 7.3/7.3/7.3 | NR | NR | 56.3/56.8/56.5  55.7/59.4/58 | Double-blind RCT in 490 centres in 3 countries, ADOPT trial | 3 | Rosiglitazone vs. metformin + glyburide | None | 4-8 | 209 |
| NCT00367055; 2010^43^ | T2DM (*n*=84) | NR | 7.5±0.5/7.3±0.5 | NR | NR | 58.3/58.1  75.0/61.0 | Phase IV, open-label RCT in France | 2 | Rosiglitazone + metformin^¶^ vs. gliclazide + metformin^¶^ | Metformin | 4-8 | 144 |
| AVM100264; 2008^44^  NCT00359112 | T2DM or IR (*n*=595) | NR | 8.0±0.8/7.9±0.9 | NR | NR | 58.5/59.3  53.0/53.0 | Phase IV, double-blind RCT in 18 centres in 11 countries | 2 | Rosiglitazone + metformin^¶^ vs. metformin + sulfonylurea | Metformin | 4-8 | 52 |
| DeFronzo 2012^45^  NCT00328627 | T2DM (*n*=1554) | 7.6±7.1/5.9±10.5/6.0±5.0 | 8.5±0.7/8.6±0.7/8.5±0.6 | NR | NR | 54.4/53.2/55.2  45.0/46.0/47.0 | Double-blind RCT in 327 centres in 20 countries | 5 | Pioglitazone + alogliptin^¶^ vs. alogliptin + placebo | Metformin | 15, 30, 45 | 26 |
| Rosenstock 2010^46^  NCT00395512 | T2DM (*n*=654) | 3.2±3.5/3.2±3.7 | 8.7±0.9/8.8±0.9 | NR | NR | 53.0/53.0 | Double-blind RCT in USA | 4 | Pioglitazone + alogliptin^¶^ vs. alogliptin | None | 30 | 26 |
| Gomis 2012^47^  NCT00736099 | T2DM (*n*=2121) | ≤1 and >5/ ≤1 and >5 | 7.8±1.0/7.3±0.9 | NR | NR | 57.8/57.8  53.0/52.0 | Phase III, open-label trial in 232 centres in 31 countries | 2 | Pioglitazone/linagliptine^¶^ vs. linagliptine | Metformin + sulfonylurea | 30 | 78 |
| NCT01204294; 2014^48^ | T2DM (*n*=574) | NR | NR | NR | NR | 60.4/60.8  79.0/69.0 | Phase III, open-label RCT in 43 centres in Japan | 7 | TZDs + linagliptin vs. linagliptin | Metformin + sulfonylurea + biguanide + glinide + α-glycoside Is | NR | 52 |
| Takihata 2013^49^  UMIN000004716 | T2DM (*n*=115) | NR | 7.4±0.6/7.4±0.6 | NR | NR | 60.7/60.3  56.0/62.0 | Open-label RCT in 9 centres in Japan COMPASS trial | 2 | Pioglitazone vs. sitagliptin | Metformin + sulfonylurea | 15-30 | 24 |
| NCT01777282; 2016^50^ | T2DM (*n*=374) | NR | NR | NR | NR | 59.0/57.0  82.0/68.0 | Phase III, open-label RCT in 49 centres in Japan | 5 | TZDs + albiglutide vs. albiglutide | Sulfonylurea + biguanide + glinide + α-glycoside Is | NR | 52 |
| NCT01468181; 2015^51^ | T2DM (*n*=394) | NR | NR | NR | NR | 56.4/57.1  79.0/75.0 | Phase III, open-label trial in Japan | 5 | TZDs + dulaglutide vs. dulaglutide | Sulfonylurea + biguanide + glinide + α-glycoside Is | NR | 52 |
| Araki 2015^52^ NCT01368081 | T2DM (*n*=1160) | 40±29/40±29 | 7.9±4.2/7.9±4.2 | NR | NR | 60.4/60.0  79.0/70.0 | Phase III, double-blind RCT in 86 centres in Japan | 13 | TZDs + empagliflozin vs. empagliflozin | Metformin + sulfonylurea + biguanide + glinide + α-glycoside Is + DPP4-Is | NR | 52 |
| Abdul-Ghani 2014^53^  NCT01107717 | T2DM (*n*=170) | 5.1±0.6/5.1±0.7 | 8.6±0.2/8.6±0.2 | NR | NR | 46.0/47.0  62.0/55.0 | Open-label RCT in San Antonio USA, EDICT trial | 2 | Pioglitazone/metformin/  exenatide vs. metformin/  sulfonylurea/insulin | None | 15, 30, 45 | 104 |
| Ruilope 2014^54^  NCT01043029^†^ | T2DM with CKD-III (*n*=301) | 10.2/9.6 | 7.6±0.9/7.5±0.9 | NR | NR | 68.2/66.8  47.0/50.0 | Phase IIb, double-blind RCT in 4 countries, AleNephro trial | 2 | Pioglitazone + aleglitazar vs. non-pioglitazone | None ± metformin ± sulfonylurea | Pioglitazone 45  Aleglitazar 0.15 | 52 |
| Bosi 2011^55^  NCT00432276^†^ | T2DM (*n*=803) | 7.5/6.9 | 8.3/8.1 | NR | NR | 54.3/55.9  52.0/51.1 | Double-blind RCT in 22 countries | 2 | Pioglitazone + alogliptin vs. non-pioglitazone | Metformin + pioglitazone | 30-45 | 52 |
| Yoon 2011^56^ NCT00397631^†^ | T2DM (*n*=520) | 2.4±4.1/2.4±4.1 | 9.5±1.2/9.5±1.2 | NR | NR | 50.9/50.9  54.0/54.0 | Phase III double-blind RCT in USA, 0431-064 trial | 2 | Pioglitazone + sitagliptin^¶^ vs. non-pioglitazone | None | 30 | 24 |
| Kashiwagi 2011^57^ NCT00372060^†^ | T2DM (*n*=134) | NR | 7.7±0.9/7.6±0.8 | NR | NR | 57.8/59.0  58.0/72.0 | Phase III, double-blind RCT in Japan 0431-055 | 2 | Pioglitazone + sitagliptin + placebo vs. non-pioglitazone | Pioglitazone | 15 | 52 |
| NCT01076075^†^; 2016^58^ | T2DM (*n*=422) | NR | 8.4±0.9/8.4±0.8 | NR | NR | 54.9/54.4  46.0/45.0 | Phase III, double-blind RCT in 3 countries, MK-0431-229 trial | 2 | Pioglitazone + sitagliptin + placebo vs. non-pioglitazone | Metformin + sulfonylurea + pioglitazone | 30 | 54 |
| Rosenstock 2006^59^ NCT00086502^†^ | T2DM (*n*=353) | 6.1±5.4/6.1±5.7 | 8.1±0.8/8.0±0.8 | NR | NR | 55.6/56.9  53.0/58.0 | Phase III, double-blind RCT in USA | 2 | Pioglitazone + sitagliptin + placebo vs. non-pioglitazone | Pioglitazone | 15, 30, 45 | 24 |
| Fonseca 2013^60^ NCT00885352^†^ | T2DM (*n*=313) | 9.8±5.9/9.8±5.9 | 8.7±1.0/8.7±1.0 | NR | NR | 56.1/56.1  62.0/62.0 | Phase III, double-blind RCT in 58 centres in 12 countries, MK-0431-128 trial | 2 | Pioglitazone + sitagliptin + placebo vs. non- pioglitazone | Metformin + pioglitazone ± sulfonylurea | 30-45 | 26 |
| Charbonnel 2006^61^ NCT00086515^†^ | T2DM (*n*=701) | NR | 8.0±0.8/8.0±0.8 | NR | NR | 54.5/54.5  57.0/57.0 | Phase III, double-blind RCT in USA, 0431-020 trial | 2 | Pioglitazone + sitagliptin + glipizide + placebo vs. non-pioglitazone | Metformin + pioglitazone | 15 | 24 |
| Forst 2014^62^  NCT01106690^†^ | T2DM (*n*=684) | 10.5±7.0/10.5±7.0 | 7.9±1.0/7.9±1.0 | NR | NR | 57.4/57.4  63.0/63.0 | Phase III, double-blind RCT in 74 centres in 11 countries, CANTATA-MP trial | 3 | Pioglitazone + sitagliptine + canagliflozin + placebo vs. non-pioglitazone | Metformin +  pioglitazone | 30-45 | 26 |
| Kovacs 2014^63^ NCT01289990^†^ | T2DM (*n*=2705) | 31±18.8/27±16.1/26±15.8 | 8.1±0.9/8.1±0.9/8.2±0.9 | NR | NR | 54.7/55.9/55.1/55.9  48/57/63/53 | Phase III, double-blind trial in 69 centres in 8 countries | 13 | Pioglitazone + sitagliptin + empagliflozin + placebo vs. non-pioglitazone | Metformin + pioglitazone + sulfonylurea | 30-45 | 76 |
| Philis-Tsimikas 2013^64^ NCT01046110^†^ | T2DM (*n*=454) | 7.8±6.1/7.8±6.1 | 8.9±1.0/8.9±1.0 | NR | NR | 55.7/55.7  59.0/59.0 | Phase III, open-label RCT in 78 centres in 7 countries, BEGIN^TM^ trial | 2 | Pioglitazone + sitagliptin + insulin degludec vs. non-pioglitazone | Metformin+ pioglitazone + sulfonylureas + glinides | NR | 26 |
| Nauck 2016^65^ NCT01183013 | T2DM (*n*=936) | ≤1 and >10/≤1 and >10 | 8.1±0.9/8.1±0.9 | NR | NR | 57.1/57.1  54.0/54.0 | Phase III, double-blind RCT in 132 centres in 6 countries | 7 | Pioglitazone + linagliptin^¶^ vs. non-pioglitazone | Pioglitazone | 15, 30, 45 | 30 |
| Gantz 2017^66^ NCT01697592^†^ | T2DM (*n*=580) | 9.18±5.4 | 7.9±0.6 | NR | NR | 61.0/60.5  72.0/72.0 | Phase III, double-blind RCT in Japan MK-3102-015 trial | 10 | Pioglitazone/omarigliptin + placebo vs. non-pioglitazone | Pioglitazone + sulfonylurea + biguanide + glinide + α-glycoside Is | NR | 52 |
| Hollander 2011^67^  NCT00295633^†^ | T2DM (*n*=565) | 5.2±10.2/5.1±5.4 | 8.3±1.1/8.2±1.1 | NR | NR | 54.9/54.0  51.0/46.0 | Phase III, double-blind RCT in USA | 3 | TZDs + saxagliptin + placebo vs. no-TZDs | TZDs | Pioglitazone 30-45 Rosiglitazone 4-8 | 76 |
| Kadowaki 2013^68^ NCT01026194^†^ | T2DM (*n*=204) | 7.3±5.3/8.1±5.8 | 8.1±0.8/7.9±0.8 | NR | NR | 60.4/60.4  71.0/71.0 | Phase III, double-blind RCT in Japan | 2 | Pioglitazone/teneligliptin^¶^ + placebo vs. non-pioglitazone | Pioglitazone | NR | 52 |
| Davies 2015^69^  NCT01272232^†^ | T2DM with metabolism and nutrition disorder (*n*=844) | 7.3±5.4/7.3±5.4 | 7.9±0.8/7.9±0.8 | NR | NR | 54.9/54.9  50.0/50.0 | Phase III, double-blind RCT in 126 centres in 9 countries SCALE™ trial | 3 | TZDs + liraglutide + placebo vs. no-TZDs | Metformin + TZDs + sulfonylurea | NR | 68 |
| Kiyosue 2018^70^ NCT01512108^†^ | T2DM (*n*=360) | 8.1±6.1 | 8.1±0.8/8.1±0.8 | NR | NR | 59.5/59.5  73.0/73.0 | Phase III, open-label RCT in 36 centres in Japan | 2 | TZDs + liraglutide + additional AHG vs. no-TZDs | Metformin + TZDs + glinide + α-glycoside Is | NR | 52 |
| Norwood 2012^71^ NCT00753896^†^ | T2DM (*n*=134) | 6.0 | 7.2±0.9/7.2±0.9 | NR | NR | 55.0/55.0  55.0/55.0 | Phase III, crossover open-label, randomized trial in 6 countries | 2 | TZDs + metformin + exenatide vs. no-TZDs | TZDs | Pioglitazone 30-45 Rosiglitazone ≥4 | 52 |
| Wysham 2014^72^ NCT01064687^†^ | T2DM (*n*=978) | 8.7±5.6/8.7±5.6 | 8.1±1.3/8.1±1.3 | NR | NR | 55.7/55.7  58.0/58.0 | Phase III, double-blind RCT in 89 centres in 4 countries, AWARD-1 trial | 4 | Pioglitazone+ exenatide + dulaglutide + placebo vs. non-pioglitazone | Metformin + pioglitazone | 30 | 52 |
| NCT00765817^†^; 2015^73^ | T2DM (*n*=259) | NR | NR | NR | NR | 59.0/59.0  57.0/57.0 | Phase III, double-blind RCT in 59 centres in 4 countries | 2 | Pioglitazone + exenatide + insulin glargine + placebo vs. non-pioglitazone | Metformin + pioglitazone | NR | 30 |
| Bode 2015^74^  NCT01106651^†^ | T2DM (*n*=714) | 11.7±7.5/11.7±7.5 | 7.7±0.8/7.7±0.8 | NR | NR | 63.6/63.6  56.0/56.0 | Phase III, double-blind RCT in 90 centres in 17 countries | 3 | TZDs + canagliflozin + placebo vs. no-TZDs | Metformin + sulfonylurea + TZDs + α-glycoside Is + DPP4-Is + insulin | NR | 104 |
| Rosenstock 2012^75^  NCT00683878^†^ | T2DM (*n*=420) | 5.6±5.9/5.0±5.0 | 8.3±0.9/8.3±1 | NR | NR | 53.8/53.5  48.7/51.0 | Double-blind RCT in 105 centres in 8 countries | 3 | Pioglitazone + dapagliflozin + placebo vs. non-pioglitazone | Pioglitazone | 30-45 | 24 |
| Kovacs 2014^63^  NCT01210001^†^ | T2DM (*n*=498) | >10.0/>10.0 | 8.1±0.8/8.2±0.9 | NR | NR | 54.5/54.6  50.5/44.2 | Phase III, double-blind RCT in 69 centres in 8 countries, EMPA-REG PIO^TM^ trial | 3 | Pioglitazone + empagliflozin + placebo vs. non-pioglitazone | Pioglitazone ± metformin | 30-45 | 24 |
| Garber 2012^76^ NCT00972283^†^ | T2DM (*n*=1004) | 13.5 | 8.4±0.9/8.3±0.8 | NR | NR | 58.1/95.2  54.0/54.0 | Phase III, open-label RCT in 136 centres in 4 countries, BEGIN^TM^ trial | 2 | ± Pioglitazone + insulin degludec + insulin glargine vs. non-pioglitazone | Metformin + pioglitazone | NR | 78 |
| Fulcher 2019^77^  NCT01009580^†^ | T2DM (*n*=446) | 18.3±8.6 | 8.4±0.8/8.4±0.8 | NR | NR | 58.7/58.7  56.0/56.0 | Phase III, open-label RCT in 53 centres in 3 countries, BOOST^TM^ trial | 2 | ± Pioglitazone + insulin degludec/insulin aspart + biphasic insulin aspart vs. non-pioglitazone | Metformin + pioglitazone + DPP4-Is | NR | 27 |
| Gross 2016^78^ NCT01175824 | T2DM (*n*=476) | 11.5±7.1 | 8.6±0.7 | NR | NR | 57.5/57.5  45.0/45.0 | Phase IV, open-label RCT in 8 countries | 2 | ± Pioglitazone + insulin lispro + insulin glargine vs. non-pioglitazone | Metformin + pioglitazone ± insulin | NR | 24 |

***Abbreviations:*** *AH,* anti-hyperglycaemic drugs; *BMD*, bone mineral density; *CVD,* cardiovascular disease; *CKD,* chronic kidney disease; *CAD,* coronary artery disease; *CHD,* coronary heart disease, *DPP4-Is*, dipeptidyl peptidase-4 inhibitors; *GLP1-RAs,* glucagon-like peptide-1 receptor agonists; *HbA_1c_*, glycosylated form of hemoglobin; *HCV,* hepatitis C virus; *IGT*, impaired glucose tolerance; *IR*, insulin resistance; *mg/day,* milligram pear day; NR*,* not report; *RCT*, randomized controlled trial; *T2DM,* type 2 diabetes mellitus; *TZD,* thiazolidinedione; *TIA,* transient ischemic attack.

***Note:* ^‡^** Mean age and dosages present for active TZDs drugs (pioglitazone or rosiglitazone or rivoglitazone or aleglitazar); ^**^ Median age or follow-up; **^¶^** Novel treatment of fixed-dose combination of 2 different classes of AHG; **^†^**these trials report fractures in both groups however all their participants had been exposed to TZDs.

## Fracture outcome results

Table S2. Trial outcomes of adverse event fractures

| Study | Treatment groups | Fracture outcomes | TZDs | | Controls | | Fracture per comparator |
| --- | --- | --- | --- | --- | --- | --- | --- |
|  |  |  | **Events Total** | | **Events Total** | |  |
| Kernan and Viscoli 2016^1^ | Pioglitazone vs. placebo | AEs  Non-stress  Stress  Low energy  High-energy  Pathological  Non-pathologic  Low-energy, non-pathologic | 133  F: 64  M: 64  204  10  F: 88  M: 90  F: 6  M: 29  3  F: 96  M: 119  F: 88  M: 88 | 1939  F: 646  M: 1293  F: 646  M: 1293  F: 646  M: 1293  F: 646  M: 1293  F: 646  M: 1293 | 94  F: 48  M: 44  137  8  F: 73  M: 46  F: 4  M: 18  3  F: 79  M: 62  F: 73  M: 4 | 1937  F: 692  M: 1245  F: 692  M: 1245  F: 692  M: 1245  F: 692  M: 1245  F: 692  M: 1245 | Pioglitazone F: 59 upper-limb (16 humerus, 20 radius, 7 ulna, 4 carpal, 12 metacarpal/phalange), 14 proximal-lower-limb (11 hip, 8 pelvis, 3 femur, 24 spine (7 lumbar, 12 thoracic, 5 cervical/sacrum)), 6 rib, 62 distal-lower-limb (1 patella, 23 fibula, 17 tibia, 4 tarsal, 17 metatarsal/phalange), 13 other (4 skull/face, 1 scapula, 0 clavicle)  Pioglitazone M: 34 upper-limb (9 humerus, 10 radius, 1 ulna, 3 carpal, 11 metacarpal/phalange), 15 proximal-lower-limb (14 hip, 4 pelvis, 1 femur, 30 spine (17 lumbar, 10 thoracic, 3 cervical/sacrum)), 43 rib, 55 distal-lower-limb (2 patella, 14 fibula, 17 tibia, 5 tarsal, 17 metatarsal/phalange), 21 other (13 skull/face, 1 scapula, 3 clavicle)  Placebo F: 34 upper-limb (7 humerus, 12 radius, 7 ulna, 2 carpal, 6 metacarpal/phalange), 14 proximal-lower-limb (10 hip, 7 pelvis, 4 femur, 24 spine (12 lumbar, 7 thoracic, 5 cervical/sacrum)), 12 rib, 33 distal-lower-limb (2 patella, 9 fibula, 7 tibia, 1 tarsal, 14 metatarsal/phalange), 11 other (2 skull/face, scapula, 2 clavicle)  Placebo M: 34 upper-limb (10 humerus, 7 radius, 6 ulna, 2 carpal, 9 metacarpal/phalange), 7 proximal-lower-limb (7 hip, 3 pelvis, femur, 4 spine (2 lumbar, 2 thoracic, 0 cervical/sacrum)), 17 rib, 20 distal-lower-limb (patella, 6 fibula, 4 tibia, tarsal, 10 metatarsal/phalange), 15 other (9 skull/face, 0 scapula, 3 clavicle)  Overall multiple fractures^§^ 190 (94 F; 96 M) (128 (64F/64M pioglitazone) and (92 (48F/44M placebo) |
| Grey 2014^3^ | Pioglitazone vs. placebo | AEs | 2 | 43 | 0 | 43 | Pioglitazone: 2 ankle, 1 toe |
| Bray 2013^4^ | Pioglitazone vs. placebo | AEs | 9 | 303 | 8 | 299 | Pioglitazone: 9 traumatic fractures (0 hand, 1 right collarbone, 3 wrists, 1 tibia, 1 fibula, 2-foot, 0 ankle, 1 toe)  Placebo: 8 traumatic fractures (1 hand, 0 right collarbone, 0 wrist, 0 tibia, 1 fibula, 2-foot, 3 ankle, 1 toe) |
| Bone 2013^5^ | Pioglitazone vs. placebo | AEs | F:1 | F:78 | F:3 | F:78 | Pioglitazone postmenopausal F (pathological fracture): radius, metacarpus  Placebo postmenopausal F (pathological fracture): ankle, lateral malleolus, metatarsal |
| Sanyal 2010^7^ | Pioglitazone vs. placebo | AEs | 3 | 80 | 5 | 83 | Pioglitazone 30 mg: 3  Placebo: 5 |
| Ramachandran 2009^8^ | Pioglitazone vs. placebo | AEs | 4 | 204 | 4 | 203 | Pioglitazone 30 mg: traumatic fracture (RTA)  Placebo: traumatic fracture (RTA) |
| Aithal 2008^9^ | Pioglitazone vs. placebo | AEs | 0 | 37 | 0 | 37 | No fracture events |
| Dormandy 2005^10^ | Pioglitazone vs. placebo | AEs | 74  F:44  M:30 | 2605  F:870  M:1735 | 60  F:23  M:37 | 2633  F:905  M:1728 | Pioglitazone F: 6 spine, 2 hip, 7 proximal-limbs, 22 distal-limbs, 7 undefined limb, 2 osteoporotic/pathological  Placebo F: 0 spine, 0 hip, 1 proximal-limb, 11 distal-limbs, 4 undefined limb, 2 osteoporotic/pathological  M NR |
| Home 2014^11^ NCT00839527 | Pioglitazone + albiglutide vs. placebo | AEs | 1 | 548 | 5 | 115 | Pioglitazone 30-45 mg + metformin ≥1500 mg + glimepiride 4 mg: 1/277 (AEs: rib)  Albiglutide 30 mg + metformin ≥1500 mg + glimepiride 4 mg: 2/271 (AEs: rib)  Metformin + glimepiride + placebo AEs: 3/115 (rib) |
| Truitt 2010^13^ | Pioglitazone + rivoglitazone vs. placebo | AEs | 3 | 347 | 1 | 91 | Pioglitazone 45 mg: 1/90 (traumatic multiple fracture (RTA)  Rivoglitazone 1 mg: 1/86 (foot)  Rivoglitazone 2 mg: 1/85 (foot)  Rivoglitazone 3 mg: 0/86  Placebo: 1/91 (foot) |
| Kaku 2009^16^ | Pioglitazone vs. control | AEs | 18 | 293 | 18 | 294 | NR |
| Abe 2010^17^ | Pioglitazone/conventional AHG vs. control | AEs | 0 | 31 | 0 | 32 | No fracture events |
| Gold 2010^20^ | Rosiglitazone vs. placebo | AEs | 2  F:2  M:0 | 331  129  71 | 3  F:3  M:0 | 165  60  40 | Rosiglitazone 2 mg F AEs: 2/166 (hip, hand)  Rosiglitazone 2 mg AEs: 0/165  Placebo F AEs: hip, upper arm, wrist. |
| Gruntmanis 2010^21^ | Rosiglitazone vs. placebo | AEs | 2  F:1  M:1 | 56  F:23  M:33 | 0  F:0  M:0 | 55  F:22  M:33 | Rosiglitazone  F: left humerus (fall)  M: rib (fall) |
| GSK49653/334; 2008^22^ | Rosiglitazone vs. placebo | AEs | 0 | 277 | 1 | 278 | Placebo T2DM AEs: facial fracture |
| Zinman 2010^24^ | Rosiglitazone/metformin**^¶^** vs. placebo | AEs | 4 | 103 | 6 | 104 | NR |
| Bach 2013^25^ | Rosiglitazone vs. no-TZDs | Bone fracture | 82  50  F:29  M:21 | 1084  680  F:210  M:470 | 74  45  F:18  M:27 | 1076  680  F:210  M:470 | NR |
| Erdmann 2015^26^ | Aleglitazar vs. placebo | AEs | 2 | 999 | 5 | 997 | Aleglitazar 0.15 mg: 1 fracture in T2DM and other on pre-diabetic |
| Esposito 2011^27^ | Pioglitazone vs. metformin | AEs | 0 | 55 | 0 | 55 | No fracture events |
| Seufert J (b) 2008^28,29^ | Pioglitazone vs. metformin | AEs | 0  F:0  M:0 | 319  F:148  M:171 | 2  F:1  M:1 | 320  F:145  M:175 | NR |
| Perez 2009^30^ | Pioglitazone/metformin**^¶^** vs. metformin | AEs | 1  F:1  M:0 | 390  F:234  M:156 | 1  F:0  M:1 | 210  F:112  M:98 | Pioglitazone/metformin F: 1/200 wrist  Pioglitazone: 0/190  Metformin M: 1/210 traumatic (RTA) wrist |
| Borges 2011^33^ | Rosiglitazone/metformin**^¶^** vs. metformin | AEs | 5  F:5  M:0 | 344  F:160  M:184 | 4  F:3  M:1 | 334  F:158  M:176 | Rosiglitazone/metformin AEs: 5  Metformin AEs: 4 |
| Tolman 2009^35^ | Pioglitazone vs. glibenclamide (glyburide) | AEs | 30  F:16  M:14 | 1051  F:450  M:601 | 27  F:13  M:14 | 1046  F:465  M:581 | NR |
| Nissen 2008^37^ | Pioglitazone vs. glimepiride | AEs | 8  F:6  M:2 | 270  F:84  M:186 | 0  F:0  M:0 | 273  F:93  M:180 | Pioglitazone: upper-limb, 2 lower-limb, hand, facial, 2 humerus, wrist, metacarpal, rib, malleolar, 2 foot  NR by gender |
| Seufert J (a) 2008^28,39^ | Pioglitazone vs. gliclazide | AEs | 1  F:1  M:0 | 317  F:156  M:161 | 1  F:1  M:0 | 313  F:159  M:154 | NR |
| Comaschi 2007^40^ | Pioglitazone + sulfonylurea vs. metformin/glibenclamide**^¶^** | AEs | 1 | 340 | 0 | 80 | Pioglitazone: 1/170 hip arthroplasty  Pioglitazone + metformin: 0/103  Pioglitazone + sulfonylurea: 0/67  Metformin + glibenclamide: 0/80 |
| Home 2009^41^ | Rosiglitazone vs. metformin + sulfonylurea (active control) | AEs | 185  F:124  M:61 | 2220  F:1078  M:1142 | 118  F:68  M:50 | 2227  F:1075  M:1152 | Rosiglitazone F: 8 spine, 7 femur/hip, 0 pelvis, 63 upper-limb, 47 distal-lower-limb, 11 others  Rosiglitazone M: 6 spine, 3 femur/hip, 0 pelvis, 23 upper-limb, 23 distal-lower-limb, 14 others  Active control F: 4 spine, 7 femur/hip, 1 pelvis, 36 upper-limb, 16 distal-lower-limb, 1 other  Active control M: 5 spine, 1 femur/hip, 3 pelvis, 19 upper-limb, 11 distal-lower-limb, 15 others |
| Kahn 2008^42^ | Rosiglitazone vs. metformin + glyburide | AEs  High energy or low energy  Pathological/osteoporotic | 92  F:60  M:32  F: 7  M: 0  F: 10  M: 0 | 1456  F:645  M:811  F:645  M:811  F:645  M:811 | 108  F:51  M:57  F: 10  M: 0  F: 7  M: 0 | 2895  F:1195  M:1700  F:1195  M:1700 F:1195  M:1700 | Rosiglitazone F: 1spine, 22 upper-limb (3 upper-limb, 0 forearm, 5 humerus, 1 radius, 8 hand, 5 wrist, 1 clavicle), 36 lower-limb (2 hip, 0 pelvic, 2 femur, 1 lower-limb, 1 tibia, 3 fibula, 5 ankle, 1 patella, 22 foot), 4 thoracic cage, 1 skull and facial, 0 not specific (60/645 total AEs)  Rosiglitazone M: 3 spine, 10 upper-limb (0 upper-limb, 2 humerus, 1 radius, 6 hand, 2 wrist, 0 clavicle), 10 lower-limb (1 hip, 0 femur, 1 lower-limb, 2 tibia, 2 fibula, 2 ankle, 0 patella, 3 foot), 9 thoracic cage, 0 skull and facial, 2 not specific (32/811 total AEs)  Metformin F: 1 spine, 10 upper-limb (2 upper-limb, 0 forearm, 0 humerus, 1 radius, 4 hand, 3 wrist, 0 clavicle), 18 lower-limb (2 hip, 2 pelvic, 0 femur, 2 lower-limb, 0 tibia, 2 fibula, 6 ankle, 1 patella, 7 foot), 2 thoracic cage, 0 skull and facial, 0 not specific (30/590 total AEs)  Metformin M: 0 spine, 7 upper-limb (1 upper-limb, 0 humerus, 2 radius, 3 hand, 2 wrist, 1 clavicle), 13 lower-limb (0 hip, 2 femur, 0 lower-limb, 0 tibia, 1 fibula, 3 ankle, 0 patella, 8 foot), 8 thoracic cage, 1 skull and facial, 1 not specific (29/864 total AEs)  Glyburide F: 1 spine, 9 upper-limb (1 upper-limb, 1 forearm, 0 humerus, 2 radius, 1 hand, 4 wrist, 0 clavicle), 8 lower-limb (0 hip, 0 pelvic, 0 femur, 0 lower-limb, 1 tibia, 0 fibula, 3 ankle, 0 patella, 4 foot), 1 thoracic cage, 1 skull and facial, 2 not specific (21/605 total AEs)  Glyburide M: 1spine, 10 upper-limb (1 upper-limb, 3 humerus, 3 radius, 0 hand, 2 wrist, 2 clavicle), 16 lower-limb (1 hip, 0 femur, 0 lower-limb, 0 tibia, 0 fibula, 3 ankle, 1 patella, 11 foot), 2 thoracic cage, 1 skull and facial,  1 not specific (28/836 total AEs) |
| Rosenstock 2010^46^ | Pioglitazone + alogliptin**^¶^** vs. alogliptin + placebo | AEs | 0 | 1168 | 0 | 386 | No fracture events  Pioglitazone: 0/388  Pioglitazone + alogliptin: 0/780  Alogliptin: 0/257  Placebo: 0/129 |
| Rosenstock 2010^46^ | Pioglitazone + alogliptin**^¶^** vs. alogliptin | AEs | 0 | 490 | 0 | 164 | No fracture events  Pioglitazone: 0/163  Pioglitazone + alogliptin: 0/327  Alogliptin: 0/164 |
| NCT01468181; 2015^51^ | TZDs + dulaglutide vs. dulaglutide | AEs | 2 | 66 | 5 | 328 | TZDs + dulaglutide 0.75 mg AEs: 2/66 (fracture, foot)  Dulaglutide 0.75 mg + sulfonylurea AEs: 4/131 (hand, foot, 2 tooth)  Dulaglutide 0.75 mg + biguanide AEs: 0/61  Dulaglutide 0.75 mg + glinide SAEs: 0/71  Dulaglutide 0.75 mg + α-glycoside Is AEs: 1/65 (radius) |
| Abdul-Ghani 2014^53^ | Pioglitazone/metformin/exenatide vs. metformin/sulfonylurea/insulin | AEs | 0 | 79 | 0 | 91 | No fracture events |
| Ruilope 2014^†54^ | Pioglitazone + aleglitazar vs. non-pioglitazone | AEs | 5 | 301 | 0 | 0 | Pioglitazone 45 mg: 2/152 (clavicle, rib and scapula)  Aleglitazar 0.15 mg: 3/149 (humerus, ankle, foot) |
| Bosi 2011^†55^ | Pioglitazone + alogliptin vs. non-pioglitazone | AEs | 10 | 803 | 0 | 0 | Pioglitazone + metformin + alogliptin: 6/404  Pioglitazone + metformin: 4/399 |
| Kovacs 2014^†63^ | Pioglitazone + empagliflozin + placebo vs. non-pioglitazone | AEs | 7 | 498 | 0 | 0 | Empagliflozin 10 mg: 2/165  Empagliflozin 25 mg: 1/168  Placebo: 4/165 |
| Hollander 2011^†67^ | TZDs + saxagliptin + placebo vs. no-TZDs | AEs | 6 | 565 | 0 | 0 | TZDs + saxagliptin 2.5 mg: 4/195  TZDs + saxagliptin 5 mg: 1/186  TZDs + placebo: 1/184 |
| Kadowaki 2013^†68^ | Pioglitazone/teneligliptin**^¶^** + placebo vs. non-pioglitazone | AEs | 2 | 204 | 0 | 0 | Pioglitazone + teneligliptin/teneligliptin: 2/103 thoracic vertebra fracture  Pioglitazone + teneligliptin/placebo: 1/101 radius |
| Rosenstock 2012^†75^ | Pioglitazone + dapagliflozin + placebo vs. non-pioglitazone | AEs | 2  F:1  M:1 | 420  F:212  M:208 | 0  F:0  M:0 | 0  F:0  M:0 | Pioglitazone + dapagliflozin: 2/281 limb fracture  F: foot  M: hand  Pioglitazone + placebo: 0/139 |
| Gross 2016^†78^ | ± Pioglitazone + insulin lispro + insulin glargine vs. non-pioglitazone | AEs | 4 | 476 | 0 | 0 | Insulin lispro: wrist, tooth/236  Insulin lispro + insulin glargine: lower-limb, tooth/240 |

***Abbreviations:*** *AEs,* adverse events; *F,* female; *M,* male; *RTA,* road traffic accident; *TZD,* thiazolidinedione.

***Note:* ^¶^** Novel treatment of fixed-dose combination of 2 different classes of AHG; ^§^Multiple fractures per person; **^†^**these trials report fractures in both groups however all their participants had been exposed to TZDs.

Table S3. Trial outcomes of serious adverse event fractures

| Study | Treatment groups | Fracture outcomes | TZDs | | Controls | | Fracture per comparator |
| --- | --- | --- | --- | --- | --- | --- | --- |
|  |  |  | **Events Total** | | **Events Total** | |  |
| Kernan and Viscoli 2016^1^ | Pioglitazone vs. placebo | Non-stress  Stress  SAEs  Low energy  High-energy  Pathological  Non-pathologic  Low-energy, non-pathologic  Serious, low-energy, non-pathologic | 204  10  F: 41  M: 58  F: 88  M: 90  F: 6  M: 29  3  F: 96  M: 119  F: 88  M: 88  F: 35  M: 41 | F: 646  M: 1293  F: 646  M: 1293  F: 646  M: 1293  F: 646  M: 1293  F: 646  M: 1293  F: 646  M: 1293 | 137  8  F: 38  M: 24  F: 73  M: 46  F: 4  M: 18  3  F: 79  M: 62  F: 73  M: 45  F: 37  M: 15 | F: 692  M: 1245  F: 692  M: 1245  F: 692  M: 1245  F: 692  M: 1245  F: 692  M: 1245  F: 692  M: 1245 | Pioglitazone F: 59 upper-limb (16 humerus, 20 radius, 7 ulna, 4 carpal, 12 metacarpal/phalange), 14 proximal-lower-limb (11 hip, 8 pelvis, 3 femur, 24 spine (7 lumbar, 12 thoracic, 5 cervical/sacrum)), 6 rib, 62 distal-lower-limb (1 patella, 23 fibula, 17 tibia, 4 tarsal, 17 metatarsal/phalange), 13 other (4 skull/face, 1 scapula, 0 clavicle)  Pioglitazone M: 34 upper-limb (9 humerus, 10 radius, 1 ulna, 3 carpal, 11 metacarpal/phalange), 15 proximal-lower-limb (14 hip, 4 pelvis, 1 femur, 30 spine (17 lumbar, 10 thoracic, 3 cervical/sacrum)), 43 rib, 55 distal-lower-limb (2 patella, 14 fibula, 17 tibia, 5 tarsal, 17 metatarsal/phalange), 21 other (13 skull/face, 1 scapula, 3 clavicle)  Placebo F: 34 upper-limb (7 humerus, 12 radius, 7 ulna, 2 carpal, 6 metacarpal/phalange), 14 proximal-lower-limb (10 hip, 7 pelvis, 4 femur, 24 spine (12 lumbar, 7 thoracic, 5 cervical/sacrum)), 12 rib, 33 distal-lower-limb (2 patella, 9 fibula, 7 tibia, 1 tarsal, 14 metatarsal/phalange), 11 other (2 skull/face, scapula, 2 clavicle)  Placebo M: 34 upper-limb (10 humerus, 7 radius, 6 ulna, 2 carpal, 9 metacarpal/phalange), 7 proximal-lower-limb (7 hip, 3 pelvis, femur, 4 spine (2 lumbar, 2 thoracic, 0 cervical/sacrum)), 17 rib, 20 distal-lower-limb (patella, 6 fibula, 4 tibia, tarsal, 10 metatarsal/phalange), 15 other (9 skull/face, 0 scapula, 3 clavicle)  Overall multiple fractures^§^ 178 (77 F; 101 M) (99 (41F/58M pioglitazone) and (99 (38F/24M placebo) |
| Simuni 2015^2^ | Pioglitazone vs. placebo | SAEs | 1 | 139 | 0 | 71 | Pioglitazone 15 mg: 1/72 (ankle)  Pioglitazone 45 mg: 0/67 |
| Bone 2013^5^ | Pioglitazone vs. placebo | SAEs | F:0 | F:78 | F:1 | F:78 | Pioglitazone postmenopausal F: 0  Placebo postmenopausal F (pathological fracture): ankle |
| Marder 2013^6^ | Pioglitazone vs. placebo | SAEs | 1 | 129 | 0 | 127 | Pioglitazone: hip |
| Sanyal 2010^7^ | Pioglitazone vs. placebo | SAEs | 0 | 80 | 2 | 83 | Pioglitazone 30 mg: 0  Placebo SAEs: 2 |
| Home 2014^11^ NCT00839527 | Pioglitazone + albiglutide vs. placebo | SAEs | 1 | 548 | 1 | 115 | Pioglitazone 30-45 mg + metformin ≥1500 mg + glimepiride 4 mg: 1/277 (SAEs: ankle)  Albiglutide 30 mg + metformin ≥1500 mg + glimepiride 4 mg: 1/271 (SAEs: thoracic vertebra fracture)  Metformin + glimepiride + placebo SAEs: 0/115 |
| Chou 2012^12^ | Pioglitazone + rivoglitazone vs. placebo | SAEs | 1 | 1749 | 1 | 137 | Pioglitazone 45 mg: 0/739  Rivoglitazone 1 mg: 0/269  Rivoglitazone 1.5 mg: 1/741 (spinal compression fracture)  Placebo: 1/137 (hand) |
| NCT00722371; 2015^14^ | Pioglitazone + sitagliptin**^¶^** vs. sitagliptin + placebo | SAEs | 3 | 1384 | 1 | 231 | Pioglitazone: 1/693 foot  Pioglitazone/sitagliptin: 2/691 (upper-limb, foot)  Sitagliptin: 1/231 foot |
| NCT00511108; 2015^15^ | Pioglitazone + sitagliptin**^¶^** vs. sitagliptin + placebo | SAEs | 1 | 106 | 0 | 105 | Pioglitazone 30 mg + sitagliptin 100 mg: 1/52 (tibia)  Pioglitazone 30 mg: 0/54  Sitagliptin 100 mg: 0/52  Placebo: 0/53 |
| Yan 2015^18^ | Pioglitazone vs. no-TZDs | SAEs | 0 | 47 | 0 | 53 | No fracture events |
| Harrison 2012^19^ | Pioglitazone vs. no-TZDs | SAEs | 1 | 77 | 0 | 73 | Pioglitazone: lower limb |
| Gold 2010^20^ | Rosiglitazone vs. placebo | SAEs | 0  F:0  M:0 | 331  129  71 | 1  F:1  M:0 | 165  60  40 | Rosiglitazone 2 mg F SAEs: 0/166  Rosiglitazone 2 mg SAEs: 0/165  Placebo F SAEs: hip |
| GSK49653/334; 2008^22^ | Rosiglitazone vs. placebo | SAEs | 0 | 277 | 3 | 278 | Placebo IR SAEs: femoral neck, upper-limb, radius |
| GSK49635/351; 2008^23^ | Rosiglitazone vs. placebo | SAEs | 0 | 30 | 2 | 30 | Placebo: pubic rami, humerus |
| Wainstein 2012^31^ | Pioglitazone vs. metformin/sitagliptin**^¶^** | SAEs | 0 | 256 | 1 | 261 | Pioglitazone: 0/256  Metformin/sitagliptin: 1/261 femur |
| Bilezikian 2013^32^ | Rosiglitazone vs. metformin | SAEs | F:5 | F:114 | F:1 | F:112 | Rosiglitazone 8 mg postmenopausal F (pathological fractures): lumbar spine, wrist (fall), fingers (fall), lower leg (fall), toes (trauma)  Metformin (pathological fractures): wrist (fall) |
| Borges 2011^33^ | Rosiglitazone/metformin**^¶^** vs. metformin | SAEs | 2  F:2  M:0 | 344  F:160  M:184 | 0  F:0  M:0 | 334  F:158  M:176 | Rosiglitazone/metformin: 0  Rosiglitazone/metformin F: 2 ankles  Metformin: 0 |
| WH 2015^34^ | Pioglitazone/metformin/placebo vs. omarigliptin + placebo | SAEs | 1 | 80 | 0 | 1089 | Metformin/placebo: 1/80 (rib)  Omarigliptin 0.25 mg: 0/113  Omarigliptin 1 mg: 0/115  Omarigliptin 3 mg: 0/114  Omarigliptin 10 mg: 0/115  Omarigliptin 25 mg: 0/114  Placebo: 0/113 |
| Jain 2006^36^ | Pioglitazone vs. glyburide | SAEs | 0 | 251 | 2 | 251 | Glyburide: ankle |
| Umpierrez 2006^38^ | Pioglitazone vs. glimepiride | SAEs | 1 | 107 | 1 | 96 | Pioglitazone: Foot  Glimepiride: Foot |
| Home 2009^41^ | Rosiglitazone vs. metformin + sulfonylurea (active control) | SAEs | 49 | 2220 | 36 | 2227 | NR |
| NCT00367055; 2010^43^ | Rosiglitazone + metformin**^¶^** vs. gliclazide + metformin**^¶^** | SAEs | 0 | 43 | 1 | 41 | Gliclazide + metformin: wrist |
| AVM100264; 2008^44^ | Rosiglitazone + metformin**^¶^** vs. metformin + sulfonylurea | SAEs | 2 | 294 | 1 | 301 | Rosiglitazone + metformin: femur, humerus  Metformin + sulfonylurea: femoral neck |
| Gomis 2012^47^ | Pioglitazone/linagliptine**^¶^** vs. linagliptine | SAEs | 2 | 589 | 13 | 1532 | Pioglitazone 30 mg + linagliptine: 2foot  Linagliptine: spinal compression, hip, femur, humerus, radius, ulna, hand, scapula, lower-limb, tibia, fibula, ankle, rib |
| NCT01204294; 2014^48^ | TZDs + linagliptin vs. linagliptin | SAEs | 0 | 74 | 3 | 500 | TZDs + linagliptin: 0/74  Sulfonylurea + metformin: 0/63  Sulfonylurea + linagliptin: 1/143 (ankle)  Biguanide + linagliptin: 1/82 (lumbar vertebra fracture)  Glinide + linagliptin: 1/66 (clavicle)  α-glycoside Is + metformin: 0/61  α-glycoside Is + linagliptin: 0/85 |
| Takihata 2013^49^ | Pioglitazone vs. sitagliptin | SAEs | 0 | 57 | 0 | 58 | No fracture events |
| NCT01777282; 2016^50^ | TZDs + albiglutide vs. albiglutide | SAEs | 1 | 61 | 1 | 313 | TZDs + albiglutide 30 mg: 1/61 (foot)  Albiglutide 30 mg + sulfonylurea: 0/120  Albiglutide 30 mg + biguanide: 0/67  Albiglutide 30 mg + glinide: 0/65  Albiglutide 30 mg + α-glycoside Is: 1/61 (femur) |
| NCT01468181; 2015^51^ | TZDs + dulaglutide vs. dulaglutide | SAEs | 1 | 66 | 1 | 328 | TZDs + dulaglutide 0.75 mg SAEs: 1/66 (pelvic)  Dulaglutide 0.75 mg + sulfonylurea SAEs: 0/131  Dulaglutide 0.75 mg + biguanide SAEs: 0/61  Dulaglutide 0.75 mg + glinide SAEs: 1/71 (ankle)  Dulaglutide 0.75 mg + α-glycoside Is SAEs: 0/65 |
| Araki 2015^52^ | TZDs + empagliflozin vs. empagliflozin | SAEs | 2 | 273 | 4 | 887 | TZDs + empagliflozin 10 mg: 0/137  TZDs + empagliflozin 25 mg: 2/136 (pelvic, ankle)  Metformin + sulfonylurea: 0/63  Empagliflozin 10 mg + sulfonylurea: 1/136 (pelvic)  Empagliflozin 25 mg + sulfonylurea: 1/137 (ankle)  Empagliflozin 10 mg + biguanide: 1/68 (femur)  Empagliflozin 25 mg + biguanide: 0/65  Empagliflozin 10 mg + glinide: 0/70  Empagliflozin 25 mg + glinide: 0/70  Empagliflozin 10 mg + α-glycoside Is: 0/69  Empagliflozin 25 mg + α-glycoside Is: 0/70  Empagliflozin 10 mg + DPP4-Is: 1/68 (femur)  Empagliflozin 25 mg + DPP4-Is: 0/71 |
| Yoon 2011^†56^ | Pioglitazone + sitagliptin**^¶^** vs. non-pioglitazone | SAEs | 1 | 520 | 0 | 0 | Pioglitazone 30 mg + sitagliptin: 1/261 humerus  Pioglitazone: 0/259 |
| Kashiwagi 2011^†57^ | Pioglitazone + sitagliptin + placebo vs. non-pioglitazone | SAEs | 3 | 134 | 0 | 0 | Sitagliptin: 2/66 patella, rib  Sitagliptin/placebo: 1/68 lower-limb |
| NCT01076075^†^; 2016^58^ | Pioglitazone/placebo + sitagliptin vs. non-pioglitazone | SAEs | 1 | 422 | 0 | 0 | Pioglitazone 30 mg/placebo: 1/212 skull  Sitagliptin: 0/210 |
| Rosenstock 2006^†59^ | Pioglitazone + sitagliptin + placebo vs. non-pioglitazone | SAEs | 1 | 353 | 0 | 0 | Sitagliptin: 0/175  Placebo: 1/178 lower-limb |
| Fonseca 2013^†60^ | Pioglitazone + sitagliptin + placebo vs. non- pioglitazone | SAEs | 1 | 313 | 0 | 0 | Sitagliptin: 0/157  Placebo: 1/156 patella |
| Charbonnel 2006^†61^ | Pioglitazone + sitagliptin + glipizide/placebo vs. non-pioglitazone | SAEs | 1 | 701 | 0 | 0 | Glipizide/placebo: 1/237 traumatic fracture  Sitagliptin: 0/464 |
| Forst 2014^†62^ | Pioglitazone + canagliflozin + sitagliptine/placebo vs. non-pioglitazone | SAEs | 3 | 684 | 0 | 0 | Canagliflozin 100 mg: 2/113 (2 periprosthetic fracture)  Canagliflozin 300 mg: 1/114 (tibia)  Sitagliptine/placebo: 0/230 |
| Kovacs 2014^†63^ | Pioglitazone + empagliflozin + sitagliptin + placebo vs. non-pioglitazone | SAEs | 16 | 2705 | 0 | 0 | Pioglitazone + empagliflozin 10 mg: 3/165 (humerus, hand, traumatic fracture)  Pioglitazone + empagliflozin 25 mg: 3/165 (upper-limb, ankle, comminute fracture)  Pioglitazone + placebo: 0/168  Metformin + placebo: 0/214  Metformin + sulfonylurea + placebo: 2/217 (femur, ankle)  Empagliflozin 10 mg: 0/229  Empagliflozin 25 mg: 1/224 (upper limb)  Empagliflozin 10 mg + metformin: 2/206 (hip, tibia)  Empagliflozin 25 mg + metformin: 2/217 (facial, comminute fracture)  Empagliflozin 10 mg + metformin + sulfonylurea: 1/225 (multiple fracture)  Empagliflozin 25 mg + metformin + sulfonylurea: 1/224 (femoral neck)  Sitagliptin 100 mg: 0/223  Placebo: 1/223 (tibia) |
| Philis-Tsimikas 2013^†64^ | Pioglitazone + sitagliptin + insulin degludec vs. non-pioglitazone | SAEs | 1 | 454 | 0 | 0 | Insulin degludec: 1/226 hip  Sitagliptin: 0/228 |
| Nauck 2016^†65^ | Pioglitazone + linagliptin**^¶^** vs. non-pioglitazone | SAEs | 1 | 936 | 0 | 0 | Linagliptin 5 mg/pioglitazone 15 mg/linagliptin 5 mg/pioglitazone 30 mg: 0/126  Linagliptin 5 mg/pioglitazone 30 mg/linagliptin 5 mg/pioglitazone 30 mg: 1/126 (traumatic fracture)  Linagliptin 5 mg/pioglitazone 45 mg/linagliptin 5 mg/pioglitazone 45 mg: 0/126  Pioglitazone 15 mg/pioglitazone 30 mg: 0/131  Pioglitazone 30 mg/pioglitazone 30 mg: 0/140  Pioglitazone 45 mg/pioglitazone 45 mg: 0/138  Linagliptin 5 mg/linagliptin 5 mg: 0/135 |
| Gantz 2017^†66^ | Pioglitazone/omarigliptin + placebo vs. non-pioglitazone | SAEs | 2 | 580 | 0 | 0 | Pioglitazone/Omarigliptin 25 mg + placebo + AHG: 2/191 (hand, fracture displacement)  Omarigliptin 25 mg + TZDs: 0/65  Omarigliptin 25 mg + sulfonylurea: 0/126  Omarigliptin 25 mg + biguanide: 0/66  Omarigliptin 25 mg + glinide: 0/65  Omarigliptin 25 mg + α-glycoside Is: 0/67 |
| Davies 2015^†69^ | TZDs + liraglutide + placebo vs. no-TZDs | SAEs | 1 | 844 | 0 | 0 | Liraglutide 1.8 mg: 1/210 (spinal compression fracture)  Liraglutide 3 mg: 0/422  Liraglutide + placebo: 0/212 |
| Kiyosue 2018^†70^ | TZDs + liraglutide + additional AHG vs. no-TZDs | SAEs | 1 | 360 | 0 | 0 | Liraglutide: 0/240  Additional AHG: 1/120 foot |
| Norwood 2012^†71^ | TZDs + metformin + exenatide vs. no-TZDs | SAEs | 1 | 134 | 0 | 0 | TZDs ± metformin + exenatide: patella |
| Wysham 2014^†72^ | Pioglitazone + exenatide + dulaglutide + placebo vs. non-pioglitazone | SAEs | 2 | 978 | 0 | 0 | Exenatide: 2/278 (humerus)  Dulaglutide 0.75 mg: 0/280  Dulaglutide 1.5 mg: 0/279  Placebo: 0/141 |
| NCT00765817^†^; 2015^73^ | Pioglitazone insulin glargine + exenatide + placebo vs. non-pioglitazone | SAEs | 1 | 259 | 0 | 0 | Exenatide: 0/137  Placebo: 1/122 ankle |
| Bode 2015^†74^ | TZDs + canagliflozin + placebo vs. no-TZDs | SAEs | 5 | 714 | 0 | 0 | Canagliflozin 100 mg: 1/241 (ankle)  Canagliflozin 300 mg: 1/236 (hip)  Placebo: 3/237 (2 cervical vertebrae, 1 hand) |
| Garber 2012^†76^ | ± Pioglitazone + insulin degludec + insulin glargine vs. non-pioglitazone | SAEs | 7 | 1004 | 0 | 0 | Insulin degludec: 3/753 (hip, humerus, ankle)  Insulin glargine: 4/251 (humerus, radius, fibula, ankle) |
| Fulcher 2019^†77^ | ± Pioglitazone + insulin degludec/insulin aspart + biphasic insulin aspart vs. non-pioglitazone | SAEs | 2 | 446 | 0 | 0 | Insulin degludec/insulin aspart: 1/224 radius  Biphasic insulin aspart: 1/222 upper-limb |
| Gross 2016^†78^ | ± Pioglitazone + insulin lispro + insulin glargine vs. non-pioglitazone | SAEs | 1 | 476 | 0 | 0 | Insulin lispr: 1/236 pelvic  Insulin lispro + insulin glargine: 0/240 |

***Abbreviations:*** *DPP4-Is,* Dipeptidyl peptidase-4 inhibitors; *F,* female; *M,* male; *SAEs, serious* adverse events; *TZD,* thiazolidinedione.

***Note:* ^¶^** Novel treatment of fixed-dose combination of 2 different classes of AHG; ^§^Multiple fractures per person; **^†^**these trials report fractures in both groups however all their participants had been exposed to TZDs.

Table S4. Trial outcomes of spine fracture

| Study | Treatment groups | Primary fracture outcomes | TZDs | | Controls | | Fracture per comparator |
| --- | --- | --- | --- | --- | --- | --- | --- |
|  |  |  | **Events Total** | | **Events Total** | |  |
| Kernan and Viscoli 2016^1^ | Pioglitazone vs. placebo | AEs and SAEs  Low-energy, non-pathological | 38  F: 19  M: 19  F: 16  M: 14 | 1939  F: 646  M: 1293  F: 646  M: 1293 | 18  F: 16  M: 2  F: 15  M: 2 | 1937  F: 692  M: 1245  F: 692  M: 1245 | Pioglitazone F: 24 spines (7 lumbar, 12 thoracic, 5 cervical/sacrum)  Pioglitazone M: 30 spines (17 lumbar, 10 thoracic, 3 cervical/sacrum)  Placebo F: 24 spines (12 lumbar, 7 thoracic, 5 cervical/sacrum)  Placebo M: 4 spines (2 lumbar, 2 thoracic, cervical/sacrum)  Overall multiple fractures^§^ 292 (155 F; 137 M) (176 (88F/88M pioglitazone) and (178 (73 F/45M placebo) |
| Dormandy 2005^10^ | Pioglitazone vs. placebo | AEs | 6  F:6  M:0 | 2605  F:870  M:1735 | 0  F:0  M:0 | 2633  F:905  M:1728 | Pioglitazone F: 6 spines  Placebo F: 0 spine  M NR |
| Home 2014^11^ NCT00839527 | Pioglitazone + albiglutide vs. placebo | AEs and SAEs | 0 | 548 | 1 | 115 | Pioglitazone 30-45 mg + metformin ≥1500 mg + glimepiride 4 mg: 0/277  Albiglutide 30 mg + metformin ≥1500 mg + glimepiride 4 mg: 1/271 (SAEs: thoracic vertebra fracture)  Metformin + glimepiride + placebo AEs: 0/115 |
| Chou 2012^12^ | Pioglitazone + rivoglitazone vs. placebo | SAEs | 1 | 1749 | 0 | 137 | Pioglitazone 45 mg: 0/739  Rivoglitazone 1 mg: 0/269  Rivoglitazone 1.5 mg: 1/741 (spinal compression fracture)  Placebo: 0/137 |
| Bilezikian 2013^32^ | Rosiglitazone vs. metformin | SAEs | F:1 | F:114 | F:0 | F:112 | Rosiglitazone 8 mg postmenopausal F (pathological fracture): lumbar spine  Metformin (pathological fracture): 0 |
| Home 2009^41^ | Rosiglitazone vs. metformin + sulfonylurea (active control) | AEs  SAEs | 14  F:8  M:6  49 | 2220  F:1078  M:1142  2220 | 9  F:4  M:5  36 | 2227  F:1075  M:1152  2227 | Rosiglitazone F: 8 spines  Rosiglitazone M: 6 spines  Active control F: 4 spines  Active control M: 5 spines  NR |
| Kahn 2008^42^ | Rosiglitazone vs. metformin + glyburide | AEs | 4  F:1  M:3 | 1456  F:645  M:811 | 3  F:2  M:1 | 2895  F:1195  M:1700 | Rosiglitazone F: 1/645 spine; M: 3/811  Metformin F: 1/590 spine; M: 0/864  Glyburide F: 1/605 spine; M: 1/836 |
| Gomis 2012^47^ | Pioglitazone/linagliptine**^¶^** vs. linagliptine | SAEs | 0 | 589 | 1 | 1532 | Pioglitazone 30 mg + linagliptine: 0  Linagliptine: spinal compression |
| NCT01204294; 2014^48^ | TZDs + linagliptin vs. linagliptin | SAEs | 0 | 74 | 1 | 500 | TZDs + linagliptin: 0/74  Sulfonylurea + metformin: 0/63  Sulfonylurea + linagliptin: 0/143  Biguanide + linagliptin: 1/82 (lumbar vertebra fracture)  Glinide + linagliptin: 0/66  α-glycoside Is + metformin: 0/61  α-glycoside Is + linagliptin: 0/85 |
| Kadowaki 2013^†68^ | Pioglitazone/teneligliptin**^¶^** + placebo vs. non-pioglitazone | AEs | 2 | 204 | 0 | 0 | Pioglitazone + teneligliptin/teneligliptin: 2/103 thoracic vertebra fracture  Pioglitazone + teneligliptin/placebo: 0/101 |
| Davies 2015^†69^ | TZDs + liraglutide + placebo vs. no-TZDs | SAEs | 1 | 844 | 0 | 0 | Liraglutide 1.8 mg: 1/210 (spinal compression fracture)  Liraglutide 3 mg: 0/422  Liraglutide + placebo: 0/212 |
| Bode 2015^†74^ | TZDs + canagliflozin + placebo vs. no-TZDs | SAEs | 2 | 714 | 0 | 0 | Canagliflozin 100 mg: 0/241  Canagliflozin 300 mg: 0/236  Placebo: 2/237 (2 cervical vertebra) |

***Abbreviations:*** *AEs,* adverse event; *F,* female; *M,* male; *NR,* not report*; SAEs, serious* adverse events; *TZD,* thiazolidinedione.

***Note:* ^¶^** Novel treatment of fixed-dose combination of 2 different classes of AHG; ^§^Multiple fractures per person; **^†^**these trials report fractures in both groups however all their participants had been exposed to TZDs.

Table S5. Trial outcomes of hip fracture

| Study | Treatment groups | Fracture outcomes | TZDs | | Controls | | Fracture per comparator |
| --- | --- | --- | --- | --- | --- | --- | --- |
|  |  |  | **Events Total** | | **Events Total** | |  |
| Kernan and Viscoli 2016^1^ | Pioglitazone vs. placebo | AEs and SAEs  Low-energy, non-pathological | 32  F: 15  M: 17  F: 10  M: 14 | 1939  F: 646  M: 1293  F: 646  M: 1293 | 25  F: 16  M: 9  F: 12  M: 6 | 1937  F: 692  M: 1245  F: 692  M: 1245 | Pioglitazone F: (11 hips, 8 pelvis)  Pioglitazone M: (14 hips, 4 pelvis)  Placebo F: (10 hips, 7 pelvis)  Placebo M: (7 hips, 3 pelvis)  Overall multiple hip fractures^§^ 42 (25pioglitazone; 17placebo) (23 (9 F/14M in pioglitazone) and (16 (9F/7M in placebo)  Overall multiple pelvis fractures^§^ 22 (12pioglitazone; 10placebo) (12 (6F/3M pioglitazone) and (10 (7F/2M placebo) |
| Marder 2013^6^ | Pioglitazone vs. placebo | SAEs | 1 | 129 | 0 | 127 | Pioglitazone: hip |
| Dormandy 2005^10^ | Pioglitazone vs. placebo | AEs | 2  F:2  M:0 | 2605  F:870  M:1735 | 0  F:0  M:0 | 2633  F:905  M:1728 | Pioglitazone F: 2 hip  Placebo F: 0 hip  M NR |
| Gold 2010^20^ | Rosiglitazone vs. placebo | AEs and SAEs | 1  F:1  M:0 | 331  129  71 | 1  F:1  M:0 | 165  60  40 | Rosiglitazone 2 mg F AEs: 1/166 (hip)  Rosiglitazone 2 mg AEs: 0/165  Placebo F AEs: hip. SAEs: hip |
| GSK49635/351; 2008^23^ | Rosiglitazone vs. placebo | SAEs | 0 | 30 | 1 | 30 | Placebo: pubic rami |
| Comaschi 2007^40^ | Pioglitazone + sulfonylurea vs. metformin/glibenclamide**^¶^** | AEs | 1 | 340 | 0 | 80 | Pioglitazone: 1/170 hip arthroplasty  Pioglitazone + metformin: 0/103  Pioglitazone + sulfonylurea: 0/67  Metformin + glibenclamide: 0/80 |
| Home 2009^41^ | Rosiglitazone vs. metformin + sulfonylurea (active control) | AEs  SAEs | 0  F:0  M:0  49 | 2220  F:1078  M:1142  2220 | 4  F:1  M:3  36 | 2227  F:1075  M:1152  2227 | Rosiglitazone F: 0 pelvis  Rosiglitazone M: 0 pelvis  Active control F: 1 pelvis  Active control M: 3 pelvises  NR |
| Kahn 2008^42^ | Rosiglitazone vs. metformin + glyburide | AEs | 3  F:2  M:1 | 1456  F:645  M:811 | 5  F:4  M:1 | 2895  F:1195  M:1700 | Rosiglitazone F: 2/645 hip, 0 pelvic; M: 1/811 hip  Metformin F: 2/590 hip, 2 pelvic; M: 0/864 hip  Glyburide F: 0/605 hip, 0 pelvic; M: 1/836 hip |
| Gomis 2012^47^ | Pioglitazone/linagliptine**^¶^** vs. linagliptine | SAEs | 0 | 589 | 1 | 1532 | Pioglitazone 30 mg + linagliptine: 0  Linagliptine: hip |
| NCT01468181; 2015^51^ | TZDs + dulaglutide vs. dulaglutide | AEs and SAEs | 1 | 66 | 0 | 328 | TZDs + dulaglutide 0.75 mg SAEs: 0/66 (pelvic)  Dulaglutide 0.75 mg + sulfonylurea AEs: 0/131  Dulaglutide 0.75 mg + biguanide AEs: 0/61  Dulaglutide 0.75 mg + glinide SAEs: 0/71  Dulaglutide 0.75 mg + α-glycoside Is AEs: 0/65 |
| Araki 2015^52^ | TZDs + empagliflozin vs. empagliflozin | SAEs | 1 | 273 | 1 | 887 | TZDs + empagliflozin 10 mg: 0/137  TZDs + empagliflozin 25 mg: 1/136 (pelvic)  Metformin + sulfonylurea: 0/63  Empagliflozin 10 mg + sulfonylurea: 1/136 (pelvic)  Empagliflozin 25 mg + sulfonylurea: 0/137  Empagliflozin 10 mg + biguanide: 0/68  Empagliflozin 25 mg + biguanide: 0/65  Empagliflozin 10 mg + glinide: 0/70  Empagliflozin 25 mg + glinide: 0/70  Empagliflozin 10 mg + α-glycoside Is: 0/69  Empagliflozin 25 mg + α-glycoside Is: 0/70  Empagliflozin 10 mg + DPP4-Is: 0/68  Empagliflozin 25 mg + DPP4-Is: 0/71 |
| Kovacs 2014^†63^ | Pioglitazone + empagliflozin + placebo + empagliflozin + sitagliptin + placebo vs. non-pioglitazone | SAEs | 1 | 2705 | 0 | 0 | Pioglitazone + empagliflozin 10 mg: 0/165  Pioglitazone + empagliflozin 25 mg: 0/165  Pioglitazone + placebo: 0/168  Metformin + placebo: 0/214  Metformin + sulfonylurea + placebo: 0/217  Empagliflozin 10 mg: 0/229  Empagliflozin 25 mg: 0/224  Empagliflozin 10 mg + metformin: 1/206 (hip)  Empagliflozin 25 mg + metformin: 0/217  Empagliflozin 10 mg + metformin + sulfonylurea: 0/225  Empagliflozin 25 mg + metformin + sulfonylurea: 0/224  Sitagliptin 100 mg: 0/223  Placebo: 0/223 |
| Philis-Tsimikas 2013^†64^ | Pioglitazone + sitagliptin + insulin degludec vs. non-pioglitazone | SAEs | 1 | 454 | 0 | 0 | Insulin degludec: 1/226 hip  Sitagliptin: 0/228 |
| Bode 2015^†74^ | TZDs + canagliflozin + placebo vs. no-TZDs | SAEs | 1 | 714 | 0 | 0 | Canagliflozin 100 mg: 0/241  Canagliflozin 300 mg: 1/236 (hip)  Placebo: 0/237 |
| Garber 2012^†76^ | ± Pioglitazone + insulin degludec + insulin glargine vs. non-pioglitazone | SAEs | 1 | 1004 | 0 | 0 | Insulin degludec: 1/753 hip  Insulin glargine: 0/251 |
| Gross 2016^†78^ | ± Pioglitazone + insulin lispro + insulin glargine vs. non-pioglitazone | SAEs | 1 | 476 | 0 | 0 | Insulin lispro SAEs: 1/236 pelvic  Insulin lispro + insulin glargine: 0/240 |

***Abbreviations:*** *AEs,* adverse event; *F,* female; *M,* male; *NR,* not report*; SAEs, serious* adverse events; *TZD,* thiazolidinedione.

***Note:* ^¶^** Novel treatment of fixed-dose combination of 2 different classes of AHG, ^§^Multiple fractures per person; **^†^**these trials report fractures in both groups however all their participants had been exposed to TZDs.

Table S6. Trial outcomes of femur fracture

| Study | Treatment groups | Fracture outcomes | TZDs | | Controls | | Fracture per comparator |
| --- | --- | --- | --- | --- | --- | --- | --- |
|  |  |  | **Events Total** | | **Events Total** | |  |
| Kernan and Viscoli 2016^1^ | Pioglitazone vs. placebo | AEs and SAEs  Low-energy, non-pathological | 4  F: 3  M: 1  F: 10  M: 14 | 1939  F: 646  M: 1293  F: 646  M: 1293 | 3  F: 3  M: 0  F: 12  M: 6 | 1937  F: 692  M: 1245  F: 692  M: 1245 | Pioglitazone F: 3 femurs  Pioglitazone M: 1 femur  Placebo F: 4 femurs  Placebo M: 0  Overall multiple fractures^§^ 8 (4pioglitazone; 4placebo) (4 (3F/1M pioglitazone) and (3 (3F/0M placebo) |
| GSK49653/334; 2008^22^ | Rosiglitazone vs. placebo | SAEs | 0 | 277 | 1 | 278 | Placebo IR SAEs: femoral neck |
| Wainstein 2012^31^ | Pioglitazone vs. metformin/sitagliptin**^¶^** | SAEs | 0 | 256 | 1 | 261 | Pioglitazone: 0/256  Metformin/sitagliptin: 1/261 femur |
| Home 2009^41^ | Rosiglitazone vs. metformin + sulfonylurea (active control) | AEs  SAEs | 10  F:7  M:3  49 | 2220  F:1078  M:1142  2220 | 8  F:7  M:1  36 | 2227  F:1075  M:1152  2227 | Rosiglitazone F: 7 femur/hip  Rosiglitazone M: 3 femur/hip  Active control F: 7 femur/hip  Active control M: 1 femur/hip  NR |
| Kahn 2008^42^ | Rosiglitazone vs. metformin + glyburide | AEs | 2  F:2  M:0 | 1456  F:645  M:811 | 2  F:0  M:2 | 2895  F:1195  M:1700 | Rosiglitazone F: 2/645femur; M: 0/811  Metformin F: 0/590 femur; M: 2/864  Glyburide F: 0/605 femur; M: 0/836 |
| AVM100264; 2008^44^ | Rosiglitazone + metformin vs. metformin + sulfonylurea | SAEs | 1 | 294 | 1 | 301 | Rosiglitazone + metformin: femur  Metformin + sulfonylurea: femoral neck |
| Gomis 2012^47^ | Pioglitazone/linagliptine**^¶^** vs. linagliptine | SAEs | 0 | 589 | 1 | 1532 | Pioglitazone 30 mg + linagliptine: 0  Linagliptine: femur |
| NCT01777282; 2016^50^ | TZDs + albiglutide vs. albiglutide | SAEs | 0 | 61 | 1 | 313 | TZDs + albiglutide 30 mg: 0/61  Albiglutide 30 mg + sulfonylurea: 0/120  Albiglutide 30 mg + biguanide: 0/67  Albiglutide 30 mg + glinide: 0/65  Albiglutide 30 mg + α-glycoside Is: 1/61 (femur) |
| Araki 2015^52^ | TZDs + empagliflozin vs. empagliflozin | SAEs | 0 | 273 | 2 | 887 | TZDs + empagliflozin 10 mg: 0/137  TZDs + empagliflozin 25 mg: 0/136  Metformin + sulfonylurea: 0/63  Empagliflozin 10 mg + sulfonylurea: 0/136  Empagliflozin 25 mg + sulfonylurea: 0/137  Empagliflozin 10 mg + biguanide: 1/68 (femur)  Empagliflozin 25 mg + biguanide: 0/65  Empagliflozin 10 mg + glinide: 0/70  Empagliflozin 25 mg + glinide: 0/70  Empagliflozin 10 mg + α-glycoside Is: 0/69  Empagliflozin 25 mg + α-glycoside Is: 0/70  Empagliflozin 10 mg + DPP4-Is: 1/68 (femur)  Empagliflozin 25 mg + DPP4-Is: 0/71 |
| Forst 2014^†62^ | Pioglitazone + canagliflozin + sitagliptine/placebo vs. non-pioglitazone | SAEs | 2 | 684 | 0 | 0 | Canagliflozin 100 mg: 2/113 (2 periprosthetic fracture)  Canagliflozin 300 mg: 0/114  Sitagliptine/placebo: 0/230 |
| Kovacs 2014^†63^ | Pioglitazone + empagliflozin + placebo + empagliflozin + sitagliptin + placebo vs. non-pioglitazone | SAEs | 2 | 2705 | 0 | 0 | Pioglitazone + empagliflozin 10 mg: 0/165  Pioglitazone + empagliflozin 25 mg: 0/165  Pioglitazone + placebo: 0/168  Metformin + placebo: 0/214  Metformin + sulfonylurea + placebo: 1/217 (femur)  Empagliflozin 10 mg: 0/229  Empagliflozin 25 mg: 0/224  Empagliflozin 10 mg + metformin: 0/206  Empagliflozin 25 mg + metformin: 0/217  Empagliflozin 10 mg + metformin + sulfonylurea: 0/225  Empagliflozin 25 mg + metformin + sulfonylurea: 1/224 (femoral neck)  Sitagliptin 100 mg: 0/223  Placebo: 0/223 |

***Abbreviations:*** *AEs,* adverse event; *F,* female; *M,* male; *NR,* not report*; SAEs, serious* adverse events; *TZD,* thiazolidinedione.

***Note:* ^¶^** Novel treatment of fixed-dose combination of 2 different classes of AHG, ^§^Multiple fractures per person; **^†^**these trials report fractures in both groups however all their participants had been exposed to TZDs.

Table S7. Trial outcomes of upper-limb fracture

| Study | Treatment groups | Fracture outcomes | TZDs | | Controls | | Fracture per comparator |
| --- | --- | --- | --- | --- | --- | --- | --- |
|  |  |  | **Events Total** | | **Events Total** | |  |
| Kernan and Viscoli 2016^1^ | Pioglitazone vs. placebo | AEs and SAEs  Low-energy, non-pathological | 70  F: 38  M: 32  F: 34  M: 22 | 1939  F: 646  M: 1293  F: 646  M: 1293 | 55  F: 26  M: 29  F: 24  M: 22 | 1937  F: 692  M: 1245  F: 692  M: 1245 | Pioglitazone F: 59 upper-limb (16 humerus, 20 radius, 7 ulna, 4 carpals, 12 metacarpal/phalange)  Pioglitazone M: 34 upper-limb (9 humerus, 10 radius, 1 ulna, 3 carpals, 11 metacarpal/phalange)  Placebo F: 34 upper-limb (7 humerus, 12 radius, 7 ulna, 2 carpals, 6 metacarpal/phalange)  Placebo M: 34 upper-limb (10 humerus, 7 radius, 6 ulna, 2 carpals, 9 metacarpal/phalange)  Overall multiple fractures^§^ 161 (93pioglitazone; 68placebo) (70 (38F/32M pioglitazone) and (55 (26F/29M placebo) |
| Dormandy 2005^10^ | Pioglitazone vs. placebo | AEs | 7  F:7  M:0 | 2605  F:870  M:1735 | 1  F:1  M:0 | 2633  F:905  M:1728 | Pioglitazone F: 7 proximal limbs  Placebo F: 1 proximal limb  M NR |
| NCT00722371; 2015^14^ | Pioglitazone + sitagliptin**^¶^** vs. sitagliptin + placebo | SAEs | 1 | 1384 | 0 | 231 | Pioglitazone: 0/693  Pioglitazone/sitagliptin: 1/691 upper-limb  Sitagliptin: 0/231 |
| Gold 2010^20^ | Rosiglitazone vs. placebo | AEs | 0  F:0  M:0 | 331  129  71 | 1  F:1  M:0 | 165  60  40 | Rosiglitazone 2 mg F AEs: 0/166  Rosiglitazone 2 mg AEs: 0/165  Placebo F AEs: upper limb |
| GSK49653/334; 2008^22^ | Rosiglitazone vs. placebo | SAEs | 0 | 277 | 1 | 278 | Placebo SAEs: upper limb |
| Nissen 2008^37^ | Pioglitazone vs. glimepiride | AEs | 1  F:6  M:2 | 270  F:84  M:186 | 0  F:0  M:0 | 273  F:93  M:180 | Pioglitazone: upper limb  NR by gender |
| Home 2009^41^ | Rosiglitazone vs. metformin + sulfonylurea (active control) | AEs  SAEs | 86  F:63  M:23  49 | 2220  F:1078  M:1142  2220 | 55  F:36  M:19  36 | 2227  F:1075  M:1152  2227 | Rosiglitazone F: 63 upper-limb  Rosiglitazone M: 23 upper-limb  Active control F: 36 upper-limb  Active control M: 19 upper-limb  NR |
| Kahn 2008^42^ | Rosiglitazone vs. metformin + glyburide | AEs | 3  F:3  M:0 | 1456  F:645  M:811 | 5  F:3  M:2 | 2895  F:1195  M:1700 | Rosiglitazone F: 3/645 upper-limb; M: 0/811  Metformin F: 2/590 upper-limb; M: 1/864  Glyburide F: 1/605 upper-limb; M: 1/836 |
| Kovacs 2014^†63^ | Pioglitazone + empagliflozin + placebo vs. empagliflozin + sitagliptin + placebo | SAEs | 2 | 2705 | 0 | 0 | Pioglitazone + empagliflozin 10 mg: 0/165  Pioglitazone + empagliflozin 25 mg: 1/165 (upper limb)  Pioglitazone + placebo: 0/168  Metformin + placebo: 0/214  Metformin + sulfonylurea + placebo: 0/217  Empagliflozin 10 mg: 0/229  Empagliflozin 25 mg: 1/224 (upper limb)  Empagliflozin 10 mg + metformin: 0/206  Empagliflozin 25 mg + metformin: 0/217  Empagliflozin 10 mg + metformin + sulfonylurea: 0/225  Empagliflozin 25 mg + metformin + sulfonylurea: 0/224  Sitagliptin 100 mg: 0/223  Placebo: 0/223 |
| Rosenstock 2012^†75^ | Pioglitazone + dapagliflozin + placebo vs. non-pioglitazone | AEs | 1  F:0  M:1 | 420  F:212  M:208 | 0  F:0  M:0 | 0  F:0  M:0 | Pioglitazone + dapagliflozin: 1/281 limb fractures  F: 0  M: hand  Pioglitazone + placebo: 0/139 |
| Fulcher 2019^†77^ | ± Pioglitazone + insulin degludec/insulin aspart + biphasic insulin aspart vs. non-pioglitazone | SAEs | 1 | 446 | 0 | 0 | Insulin degludec/insulin aspart: 0/224  Biphasic insulin aspart: 1/222 upper-limb |

***Abbreviations:*** *AEs,* adverse event; *F,* female; *M,* male; *NR,* not report*; SAEs, serious* adverse events; *TZD,* thiazolidinedione.

***Note:* ^¶^** Novel treatment of fixed-dose combination of 2 different classes of AHG; ^§^Multiple fractures per person; **^†^**these trials report fractures in both groups however all their participants had been exposed to TZDs.

Table S8. Trial outcomes of wrist fracture

| Study | Treatment groups | Fracture outcomes | TZDs | | Controls | | Fracture per comparator |
| --- | --- | --- | --- | --- | --- | --- | --- |
|  |  |  | **Events Total** | | **Events Total** | |  |
| Bray 2013^4^ | Pioglitazone vs. placebo | AEs^*^ | 3 | 303 | 0 | 299 | Pioglitazone: 3 traumatic fractures (wrist)  Placebo: 0 wrist |
| Gold 2010^20^ | Rosiglitazone vs. placebo | AEs | 0  F:0  M:0 | 331  129  71 | 1  F:1  M:0 | 165  60  40 | Rosiglitazone 2 mg F AEs: 0/166  Rosiglitazone 2 mg AEs: 0/165  Placebo F AEs: wrist. |
| Perez 2009^30^ | Pioglitazone/metformin**^¶^** vs. metformin | AEs | 1  F:1  M:0 | 390  F:234  M:156 | 1  F:0  M:1 | 210  F:112  M:98 | Pioglitazone 15 mg/metformin 850 mg BID F: 1/200 (wrist)  Pioglitazone 15 mg: 0/190  Metformin 850 mg BID M: 1/210 (traumatic wrist) |
| Bilezikian 2013^32^ | Rosiglitazone vs. metformin | SAEs | F:1 | F:114 | F:1 | F:112 | Rosiglitazone 8 mg postmenopausal F (pathological fracture): wrist (fall)  Metformin (pathological fracture): wrist (fall) |
| Nissen 2008^37^ | Pioglitazone vs. glimepiride | AEs | 1  F:6  M:2 | 270  F:84  M:186 | 0  F:0  M:0 | 273  F:93  M:180 | Pioglitazone: wrist  NR by gender |
| Kahn 2008^42^ | Rosiglitazone vs. metformin + glyburide | AEs | 7  F:5  M:2 | 1456  F:645  M:811 | 11  F:7  M:4 | 2895  F:1195  M:1700 | Rosiglitazone F: 5/645; M: 2/811  Metformin F: 3/590; M: 2/864  Glyburide F: 4/605; M: 2/836 |
| NCT00367055; 2010^43^ | Rosiglitazone + metformin**^¶^** vs. gliclazide + metformin**^¶^** | SAEs | 0 | 43 | 1 | 41 | Gliclazide + metformin: wrist |
| Gross 2016^†78^ | ± Pioglitazone + insulin lispro + insulin glargine vs. non-pioglitazone | AEs | 1 | 476 | 0 | 0 | Insulin lispro: 1//236 wrist  Insulin lispro + insulin glargine: 0/240 |

***Abbreviations:*** *AEs,* adverse event; *F,* female; *M,* male; *NR,* not report*; SAEs, serious* adverse events; *TZD,* thiazolidinedione.

***Note:* ^¶^** Novel treatment of fixed-dose combination of 2 different classes of AHG; **^†^**these trials report fractures in both groups however all their participants had been exposed to TZDs.

Table S9. Trial outcomes of lower-limb fracture

| Study | Treatment groups | Fracture outcomes | TZDs | | Controls | | Fracture per comparator |
| --- | --- | --- | --- | --- | --- | --- | --- |
|  |  |  | **Events Total** | | **Events Total** | |  |
| Kernan and Viscoli 2016^1^ | Pioglitazone vs. placebo | AEs and SAEs  Low-energy, non-pathological | 77  F: 38  M: 39  61  F: 35  M: 26 | 1939  F: 646  M: 1293  1939  F: 646  M: 1293 | 43  F: 25  M: 18  35  F: 23  M: 12 | 1937  F: 692  M: 1245  1937  F: 692  M: 1245 | Pioglitazone F: 62 distal-lower-limb (1 patella, 23 fibula, 17 tibia, 4 tarsals, 17 metatarsal/phalange)  Pioglitazone M: 55 distal-lower-limb (2 patella, 14 fibula, 17 tibia, 5 tarsals, 17 metatarsal/phalange)  Placebo F: 33 distal-lower-limb (2 patella, 9 fibula, 7 tibia, 1 tarsal, 14 metatarsal/phalange)  Placebo M: 20 distal-lower-limb (patella, 6 fibula, 4 tibias, tarsal, 10 metatarsal/phalange)  Overall multiple hip fractures^§^ 170 (117pioglitazone; 53placebo) (23 (38F/39M pioglitazone) and (16 (25F/18M placebo) |
| Dormandy 2005^10^ | Pioglitazone vs. placebo | AEs | 22  F:22  M:0 | 2605  F:870  M:1735 | 11  F:11  M:0 | 2633  F:905  M:1728 | Pioglitazone F: 22 distal limbs  Placebo F: 11 distal limbs  M NR |
| Harrison 2012^19^ | Pioglitazone vs. no-TZDs | SAEs | 1 | 77 | 0 | 73 | Pioglitazone: lower limb |
| Bilezikian 2013^32^ | Rosiglitazone vs. metformin | SAEs | F:1 | F:114 | F:0 | F:112 | Rosiglitazone 8 mg postmenopausal F (pathological fracture): lower limb (fall)  Metformin (pathological fracture): 0 |
| Nissen 2008^37^ | Pioglitazone vs. glimepiride | AEs | 2  F:6  M:2 | 270  F:84  M:186 | 0  F:0  M:0 | 273  F:93  M:180 | Pioglitazone: 2 lower-limb  NR by gender |
| Home 2009^41^ | Rosiglitazone vs. metformin + sulfonylurea (active control) | AEs  SAEs | 70  F:47  M:23  49 | 2220  F:1078  M:1142  2220 | 27  F:16  M:11  36 | 2227  F:1075  M:1152  2227 | Rosiglitazone F: 47 distal-lower-limb  Rosiglitazone M: 23 distal-lower-limb  Active control F: 16 distal-lower-limb  Active control M: 11 distal-lower-limb  NR |
| Kahn 2008^42^ | Rosiglitazone vs. metformin + glyburide | AEs | 2  F:1  M:1 | 1456  F:645  M:811 | 2  F:2  M:0 | 2895  F:1195  M:1700 | Rosiglitazone F: 1/645 lower-limb; M: 1/811  Metformin F: 2/590 lower-limb; M: 0/864  Glyburide F: 0/605 lower-limb; M: 0/836 |
| Gomis 2012^47^ | Pioglitazone/linagliptine**^¶^** vs. linagliptine | SAEs | 0 | 589 | 1 | 1532 | Pioglitazone 30 mg + linagliptine: 0  Linagliptine: lower limb |
| Kashiwagi 2011^†57^ | Pioglitazone + sitagliptin + placebo vs. non-pioglitazone | SAEs | 1 | 134 | 0 | 0 | Sitagliptin: 0/133  Sitagliptin/placebo: 1/68 lower-limb |
| Rosenstock 2006^†59^ | Pioglitazone + sitagliptin + placebo vs. non-pioglitazone | SAEs | 1 | 353 | 0 | 0 | Sitagliptin: 0/175  Placebo: 1/178 lower-limb |
| Rosenstock 2012^†75^ | Pioglitazone + dapagliflozin + placebo vs. non-pioglitazone | AEs | 1  F:1  M:0 | 420  F:212  M:208 | 0  F:0  M:0 | 0  F:0  M:0 | Pioglitazone + dapagliflozin: 1/281 limb fracture  F: foot  M: 0  Pioglitazone + placebo: 0/139 |
| Gross 2016^†78^ | ± Pioglitazone + insulin lispro + insulin glargine vs. non-pioglitazone | AEs | 1 | 476 | 0 | 0 | Insulin lispro: 0/236  Insulin lispro + insulin glargine: 1/240 lower-limb |

***Abbreviations:*** *AEs,* adverse event; *F,* female; *M,* male; *NR,* not report*; SAEs, serious* adverse events; *TZD,* thiazolidinedione.

***Note:* ^¶^** Novel treatment of fixed-dose combination of 2 different classes of AHG; ^§^Multiple fractures per person; **^†^**these trials report fractures in both groups however all their participants had been exposed to TZDs.

Table S10. Trial outcomes of ankle fracture

| Study | Treatment groups | Fracture outcomes | TZDs | | Controls | | Fracture per comparator |
| --- | --- | --- | --- | --- | --- | --- | --- |
|  |  |  | **Events Total** | | **Events Total** | |  |
| Simuni 2015^2^ | Pioglitazone vs. placebo | SAEs | 1 | 139 | 0 | 71 | Pioglitazone 15 mg: 1/72 (ankle)  Pioglitazone 45 mg: 0/67 |
| Grey 2014^3^ | Pioglitazone vs. placebo | AEs | 2 | 43 | 0 | 43 | Pioglitazone: 2 ankle |
| Bray 2013^4^ | Pioglitazone vs. placebo | AEs^*^ | 0 | 303 | 3 | 299 | Pioglitazone: 0 ankle  Placebo: 3 traumatic fractures (ankle) |
| Bone 2013^5^ | Pioglitazone vs. placebo | AEs and SAEs | F:0 | F:78 | F:2 | F:78 | Pioglitazone postmenopausal F AEs: 0  Placebo postmenopausal F AEs (pathological fracture): ankle. SAEs (pathological fracture): ankle |
| Home 2014^11^ NCT00839527 | Pioglitazone + albiglutide vs. placebo | SAEs | 1 | 548 | 0 | 115 | Pioglitazone 30-45 mg + metformin ≥1500 mg + glimepiride 4 mg: 1/277 (SAEs: ankle)  Albiglutide 30 mg + metformin ≥1500 mg + glimepiride 4 mg: 0/271  Metformin + glimepiride + placebo AEs: 0/115 |
| Borges 2011^33^ | Rosiglitazone/metformin**^¶^** vs. metformin | AEs and SAEs | 2  F:2  M:0 | 344  F:160  M:184 | 0  F:0  M:0 | 334  F:158  M:176 | Rosiglitazone/metformin AEs: 0  Rosiglitazone/metformin F SAEs: 2 ankles  Metformin AEs: 0 |
| Jain 2006^36^ | Pioglitazone vs. glyburide | SAEs | 0 | 251 | 2 | 251 | Glyburide: ankle |
| Kahn 2008^42^ | Rosiglitazone vs. metformin + glyburide | AEs | 7  F:5  M:2 | 1456  F:645  M:811 | 15  F:9  M:6 | 2895  F:1195  M:1700 | Rosiglitazone F: 5/645; M: 2/811  Metformin F: 6/590; M: 3/864  Glyburide F: 3/605; M: 3/836 |
| NCT01204294; 2014^48^ | TZDs + linagliptin vs. linagliptin | SAEs | 0 | 74 | 1 | 500 | TZDs + linagliptin: 0/74  Sulfonylurea + metformin: 0/63  Sulfonylurea + linagliptin: 1/143 (ankle)  Biguanide + linagliptin: 0/82  Glinide + linagliptin: 0/66  α-glycoside Is + metformin: 0/61  α-glycoside Is + linagliptin: 0/85 |
| Araki 2015^52^ | TZDs + empagliflozin vs. empagliflozin | SAEs | 1 | 273 | 1 | 887 | TZDs + empagliflozin 10 mg: 0/137  TZDs + empagliflozin 25 mg: 1/136 (ankle)  Metformin + sulfonylurea: 0/63  Empagliflozin 10 mg + sulfonylurea: 0/136  Empagliflozin 25 mg + sulfonylurea: 1/137 (ankle)  Empagliflozin 10 mg + biguanide: 0/68  Empagliflozin 25 mg + biguanide: 0/65  Empagliflozin 10 mg + glinide: 0/70  Empagliflozin 25 mg + glinide: 0/70  Empagliflozin 10 mg + α-glycoside Is: 0/69  Empagliflozin 25 mg + α-glycoside Is: 0/70  Empagliflozin 10 mg + DPP4-Is: 0/68  Empagliflozin 25 mg + DPP4-Is: 0/71 |
| Ruilope 2014^†54^ | Pioglitazone + aleglitazar vs. non-pioglitazone | AEs | 1 | 301 | 0 | 0 | Pioglitazone 45 mg: 0/152  Aleglitazar 0.15 mg: 1/149 ankle |
| Kovacs 2014^†63^ | Pioglitazone + empagliflozin + placebo + empagliflozin + sitagliptin + placebo vs. non-pioglitazone | SAEs | 2 | 2705 | 0 | 0 | Pioglitazone + empagliflozin 10 mg: 0/165  Pioglitazone + empagliflozin 25 mg: 1/165 (ankle)  Pioglitazone + placebo: 0/168  Metformin + placebo: 0/214  Metformin + sulfonylurea + placebo: 1/217 (ankle)  Empagliflozin 10 mg: 0/229  Empagliflozin 25 mg: 0/224  Empagliflozin 10 mg + metformin: 0/206  Empagliflozin 25 mg + metformin: 0/217  Empagliflozin 10 mg + metformin + sulfonylurea: 0/225  Empagliflozin 25 mg + metformin + sulfonylurea: 0/224  Sitagliptin 100 mg: 0/223  Placebo: 0/223 |
| NCT00765817^†^; 2015^73^ | Pioglitazone + insulin glargine + exenatide + placebo vs. non-pioglitazone | SAEs | 1 | 259 | 0 | 0 | Exenatide: 0/137  Placebo: 1/122 ankle |
| Gomis 2012^47^ | Pioglitazone/linagliptine**^¶^** vs. linagliptine | SAEs | 0 | 589 | 1 | 1532 | Pioglitazone 30 mg + linagliptine: 0  Linagliptine: ankle |
| NCT01468181; 2015^51^ | TZDs + dulaglutide vs. dulaglutide | AEs  AEs  AEs  SAEs  AEs | 0 | 66 | 1 | 328 | TZDs + dulaglutide 0.75 mg: 0/66  Dulaglutide 0.75 mg + sulfonylurea: 0/131  Dulaglutide 0.75 mg + biguanide: 0/61  Dulaglutide 0.75 mg + glinide: 1/71 (ankle)  Dulaglutide 0.75 mg + α-glycoside Is: 0/65 |
| Bode 2015^†74^ | TZDs + canagliflozin + placebo vs. no-TZDs | SAEs | 1 | 714 | 0 | 0 | Canagliflozin 100 mg: 1/241 (ankle)  Canagliflozin 300 mg: 0/236  Placebo: 0/237 |
| Garber 2012^†76^ | ± Pioglitazone + insulin degludec + insulin glargine vs. non-pioglitazone | SAEs | 2 | 1004 | 0 | 0 | Insulin degludec: 1/753 ankle  Insulin glargine: 1/251 ankle |

***Abbreviations:*** *AEs,* adverse event; *F,* female; *M,* male; *NR,* not report*; SAEs, serious* adverse events; *TZD,* thiazolidinedione.

***Note:* ^¶^** Novel treatment of fixed-dose combination of 2 different classes of AHG; **^†^**these trials report fractures in both groups however all their participants had been exposed to TZDs.

Table S11. Trial outcomes of adverse event fracture by sex difference

| Study | Treatment groups | Fracture outcomes | TZDs | | Controls | | Fracture per comparator |
| --- | --- | --- | --- | --- | --- | --- | --- |
|  |  |  | **Events Total** | | **Events Total** | |  |
| Kernan and Viscoli 2016^1^ | Pioglitazone vs. placebo | AEs  Low energy  High-energy  Non-pathologic  Low-energy, non-pathologic | 133  F: 64  M: 64  F: 88  M: 90  F: 6  M: 29  F: 96  M: 119  F: 88  M: 88 | 1939  F: 646  M: 1293  F: 646  M: 1293  F: 646  M: 1293  F: 646  M: 1293  F: 646  M: 1293 | 94  F: 48  M: 44  F: 73  M: 46  F: 4  M: 18  F: 79  M: 62  F: 73  M: 4 | 1937  F: 692  M: 1245  F: 692  M: 1245  F: 692  M: 1245  F: 692  M: 1245  F: 692  M: 1245 | Pioglitazone F: 59 upper-limb (16 humerus, 20 radius, 7 ulna, 4 carpal, 12 metacarpal/phalange), 14 proximal-lower-limb (11 hip, 8 pelvis, 3 femur, 24 spine (7 lumbar, 12 thoracic, 5 cervical/sacrum)), 6 rib, 62 distal-lower-limb (1 patella, 23 fibula, 17 tibia, 4 tarsal, 17 metatarsal/phalange), 13 other (4 skull/face, 1 scapula, 0 clavicle)  Pioglitazone M: 34 upper-limb (9 humerus, 10 radius, 1 ulna, 3 carpal, 11 metacarpal/phalange), 15 proximal-lower-limbs (14 hip, 4 pelvis, 1 femur, 30 spine (17 lumbar, 10 thoracic, 3 cervical/sacrum)), 43 rib, 55 distal-lower-limb (2 patella, 14 fibula, 17 tibia, 5 tarsal, 17 metatarsal/phalange), 21 other (13 skull/face, 1 scapula, 3 clavicle)  Placebo F: 34 upper-limb (7 humerus, 12 radius, 7 ulna, 2 carpal, 6 metacarpal/phalange), 14 proximal-lower-limb (10 hip, 7 pelvis, 4 femur, 24 spine (12 lumbar, 7 thoracic, 5 cervical/sacrum)), 12 rib, 33 distal-lower-limb (2 patella, 9 fibula, 7 tibia, 1 tarsal, 14 metatarsal/phalange), 11 other (2 skull/face, scapula, 2 clavicle)  Placebo M: 34 upper-limb (10 humerus, 7 radius, 6 ulna, 2 carpal, 9 metacarpal/phalange), 7 proximal-lower-limb (7 hip, 3 pelvis, femur, 4 spine (2 lumbar, 2 thoracic, 0 cervical/sacrum)), 17 rib, 20 distal-lower-limb (patella, 6 fibula, 4 tibia, tarsal, 10 metatarsal/phalange), 15 other (9 skull/face, 0 scapula, 3 clavicle)  Overall multiple fractures^§^ 190 (94 F; 96 M) (128 (64F/64M pioglitazone) and (92 (48F/44M placebo) |
| Bone 2013^5^ | Pioglitazone vs. placebo | AEs | F:1 | F:78 | F:3 | F:78 | Pioglitazone postmenopausal F (pathological fracture): radius, metacarpus  Placebo postmenopausal F (pathological fracture): ankle, lateral malleolus, metatarsal |
| Dormandy 2005^79^ | Pioglitazone vs. placebo | AEs | 74  F:44  M:30 | 2605  F:870  M:1735 | 60  F:23  M:37 | 2633  F:905  M:1728 | Pioglitazone F: 6 spine, 2 hip, 7 proximal-limbs, 22 distal-limbs, 7 undefined limb, 2 osteoporotic/pathological  Placebo F: 0 spine, 0 hip, 1 proximal-limbs, 11 distal-limbs, 4 undefined limb, 2 osteoporotic/pathological  M NR |
| Gold 2010^20^ | Rosiglitazone vs. placebo | AEs | 2  F:2  M:0 | 331  129  71 | 3  F:3  M:0 | 165  60  40 | Rosiglitazone 2 mg F AEs: 2/166 (hip, hand)  Rosiglitazone 2 mg AEs: 0/165  Placebo F AEs: hip, upper arm, wrist. |
| Gruntmanis 2010^21^ | Rosiglitazone vs. placebo | AEs | 2  F:1  M:1 | 56  F:23  M:33 | 0  F:0  M:0 | 55  F:22  M:33 | Rosiglitazone  F: left humerus (fall)  M: rib (fall) |
| Bach 2013^25^ | Rosiglitazone vs. no-TZDs | Bone fracture | 82  50  F:29  M:21 | 1084  680  F:210  M:470 | 74  45  F:18  M:27 | 1076  680  F:210  M:470 | NR |
| Seufert J (b) 2008^28,29^ | Pioglitazone vs. metformin | AEs | 0  F:0  M:0 | 319  F:148  M:171 | 2  F:1  M:1 | 320  F:145  M:175 | NR |
| Perez 2009^30^ | Pioglitazone/metformin**^¶^** vs. metformin | AEs | 1  F:1  M:0 | 390  F:234  M:156 | 1  F:0  M:1 | 210  F:112  M:98 | Pioglitazone 15 mg/metformin 850 mg BID F: 1/200 (wrist)  Pioglitazone 15 mg: 0/190  Metformin 850 mg BID M: 1/210 (traumatic wrist) |
| Borges 2011^33^ | Rosiglitazone/metformin**^¶^** vs. metformin | AEs | 5  F:5  M:0 | 344  F:160  M:184 | 4  F:3  M:1 | 334  F:158  M:176 | Rosiglitazone/metformin AEs: 5  Rosiglitazone/metformin F SAEs: 2 ankle  Metformin AEs: 4 |
| Tolman 2009^35^ | Pioglitazone vs. glibenclamide (glyburide) | AEs | 30  F:16  M:14 | 1051  F:450  M:601 | 27  F:13  M:14 | 1046  F:465  M:581 | NR |
| Nissen 2008^37^ | Pioglitazone vs. glimepiride | AEs | 8  F:6  M:2 | 270  F:84  M:186 | 0  F:0  M:0 | 273  F:93  M:180 | Pioglitazone: upper-limb, 2 lower-limb, hand, facial,2 humerus, wrist, metacarpal, rib, malleolar, 2 foot  NR by gender |
| Seufert J (a) 2008^28,39^ | Pioglitazone vs. gliclazide | AEs | 1  F:1  M:0 | 317  F:156  M:161 | 1  F:1  M:0 | 313  F:159  M:154 | NR |
| Home 2009^41^ | Rosiglitazone vs. metformin + sulfonylurea (active control) | AEs | 185  F:124  M:61 | 2220  F:1078  M:1142 | 118  F:68  M:50 | 2227  F:1075  M:1152 | Rosiglitazone F: 8 spine, 7 femur/hip, 0 pelvis, 63 upper-limb, 47 distal-lower-limb, 11 others  Rosiglitazone M: 6 spine, 3 femur/hip, 0 pelvis, 23 upper-limb, 23 distal-lower-limb, 14 others  Active control F: 4 spine, 7 femur/hip, 1 pelvis, 36 upper-limb, 16 distal-lower-limb, 1 other  Active control M: 5 spine, 1 femur/hip, 3 pelvis, 19 upper-limb, 11 distal-lower-limb, 15 others |
| Kahn 2008^42^ | Rosiglitazone vs. metformin + glyburide | AEs  High or low energy  Pathological/osteoporotic | 92  F:60  M:32  F: 7  M: 0  F: 10  M: 0 | 1456  F:645  M:811  F:645  M:811  F:645  M:811 | 108  F:51  M:57  F: 10  M: 0  F: 7  M: 0 | 2895  F:1195  M:1700  F:1195  M:1700 F:1195  M:1700 | Rosiglitazone F: 1spine, 22 upper-limb (3 upper-limb, 0 forearm, 5 humerus, 1 radius, 8 hand, 5 wrist, 1 clavicle), 36 lower-limb (2 hip, 0 pelvic, 2 femur, 1 lower-limb, 1 tibia, 3 fibula, 5 ankle, 1 patella, 22 foot), 4 thoracic cage, 1 skull and facial, 0 not specific (60/645 total AEs)  Rosiglitazone M: 3 spine, 10 upper-limb (0 upper-limb, 2 humerus, 1 radius, 6 hand, 2 wrist, 0 clavicle), 10 lower-limb (1 hip, 0 femur, 1 lower-limb, 2 tibia, 2 fibula, 2 ankle, 0 patella, 3 foot), 9 thoracic cage, 0 skull and facial, 2 not specific (32/811 total AEs)  Metformin F: 1 spine, 10 upper-limb (2 upper-limb, 0 forearm, 0 humerus, 1 radius, 4 hand, 3 wrist, 0 clavicle), 18 lower-limb (2 hip, 2 pelvic, 0 femur, 2 lower-limb, 0 tibia, 2 fibula, 6 ankle, 1 patella, 7 foot), 2 thoracic cage, 0 skull and facial, 0 not specific (30/590 total AEs)  Metformin M: 0 spine, 7 upper-limb (1 upper-limb, 0 humerus, 2 radius, 3 hand, 2 wrist, 1 clavicle), 13 lower-limb (0 hip, 2 femur, 0 lower-limb, 0 tibia, 1 fibula, 3 ankle, 0 patella, 8 foot), 8 thoracic cage, 1 skull and facial, 1 not specific (29/864 total AEs)  Glyburide F: 1 spine, 9 upper-limb (1 upper-limb, 1 forearm, 0 humerus, 2 radius, 1 hand, 4 wrist, 0 clavicle), 8 lower-limb (0 hip, 0 pelvic, 0 femur, 0 lower-limb, 1 tibia, 0 fibula, 3 ankle, 0 patella, 4 foot), 1 thoracic cage, 1 skull and facial, 2 not specific (21/605 total AEs)  Glyburide M: 1spine, 10 upper-limb (1 upper-limb, 3 humerus, 3 radius, 0 hand, 2 wrist, 2 clavicle), 16 lower-limb (1 hip, 0 femur, 0 lower-limb, 0 tibia, 0 fibula, 3 ankle, 1 patella, 11 foot), 2 thoracic cage, 1 skull and facial,  1 not specific (28/836 total AEs) |
| Rosenstock 2012^†75^ | Pioglitazone + dapagliflozin + placebo vs. non-pioglitazone | AEs | 2  F:1  M:1 | 420  F:212  M:208 | 0  F:0  M:0 | 0  F:0  M:0 | Pioglitazone + dapagliflozin: 2/281 limb fracture  F: foot  M: hand  Pioglitazone + placebo: 0/139 |

***Abbreviations:*** *AEs,* adverse event; *F,* female; *M,* male; *NR,* not report*; TZD,* thiazolidinedione.

***Note:* ^¶^** Novel treatment of fixed-dose combination of 2 different classes of AHG; ^§^Multiple fractures per person; **^†^**this study report fractures in both groups however all their participants had been exposed to TZDs.

Table S12. Trial outcomes of serious adverse event fracture by sex difference

| Study | Treatment groups | Fracture outcomes | TZDs | | Controls | | Fracture per comparator |
| --- | --- | --- | --- | --- | --- | --- | --- |
|  |  |  | **Events Total** | | **Events Total** | |  |
| Kernan and Viscoli 2016^1^ | Pioglitazone vs. placebo | SAEs  Low-energy  High-energy  Non-pathologic  Low-energy, non-pathologic  Serious, low-energy, non-pathologic | F: 41  M: 58  F: 88  M: 90  F: 6  M: 29  F: 96  M 119  F: 88  M 88  F: 35  M: 41 | F: 646  M: 1293  F: 646  M: 1293  F: 646  M: 1293  F: 646  M: 1293  F: 646  M: 1293  F: 646  M: 1293 | F: 38  M: 24  F: 73  M: 46  F: 4  M: 18  F: 79  M: 62  F: 73  M: 45  F: 37  M: 15 | F: 692  M: 1245  F: 692  M: 1245  F: 692  M: 1245  F: 692  M: 1245  F: 692  M: 1245  F: 692  M: 1245 | Pioglitazone F: 59 upper-limb (16 humerus, 20 radius, 7 ulna, 4 carpal, 12 metacarpal/phalange), 14 proximal-lower-limb (11 hip, 8 pelvis, 3 femur, 24 spine (7 lumbar, 12 thoracic, 5 cervical/sacrum)), 6 rib, 62 distal-lower-limb (1 patella, 23 fibula, 17 tibia, 4 tarsal, 17 metatarsal/phalange), 13 other (4 skull/face, 1 scapula, 0 clavicle)  Pioglitazone M: 34 upper-limb (9 humerus, 10 radius, 1 ulna, 3 carpal, 11 metacarpal/phalange), 15 proximal-lower-limb (14 hip, 4 pelvis, 1 femur, 30 spine (17 lumbar, 10 thoracic, 3 cervical/sacrum)), 43 rib, 55 distal-lower-limb (2 patella, 14 fibula, 17 tibia, 5 tarsal, 17 metatarsal/phalange), 21 other (13 skull/face, 1 scapula, 3 clavicle)  Placebo F: 34 upper-limb (7 humerus, 12 radius, 7 ulna, 2 carpal, 6 metacarpal/phalange), 14 proximal-lower-limb (10 hip, 7 pelvis, 4 femur, 24 spine (12 lumbar, 7 thoracic, 5 cervical/sacrum)), 12 rib, 33 distal-lower-limb (2 patella, 9 fibula, 7 tibia, 1 tarsal, 14 metatarsal/phalange), 11 other (2 skull/face, scapula, 2 clavicle)  Placebo M: 34 upper-limb (10 humerus, 7 radius, 6 ulna, 2 carpal, 9 metacarpal/phalange), 7 proximal-lower-limb (7 hip, 3 pelvis, femur, 4 spine (2 lumbar, 2 thoracic, 0 cervical/sacrum)), 17 rib, 20 distal-lower-limb (patella, 6 fibula, 4 tibia, tarsal, 10 metatarsal/phalange), 15 other (9 skull/face, 0 scapula, 3 clavicle)  Overall multiple fractures^§^ 178 (77 F; 101 M) (99 (41F/58M pioglitazone) and (99 (38F/24M placebo) |
| Bone 2013^5^ | Pioglitazone vs. placebo | SAEs | F:0 | F:78 | F:1 | F:78 | Pioglitazone postmenopausal F: 0  Placebo postmenopausal F (pathological fracture): ankle |
| Gold 2010^20^ | Rosiglitazone vs. placebo | SAEs | 0  F:0  M:0 | 331  129  71 | 1  F:1  M:0 | 165  60  40 | Rosiglitazone 2 mg F AEs: 2/166 (hip, hand)  Rosiglitazone 2 mg AEs: 0/165  Placebo F AEs: hip, upper arm, wrist. SAEs: hip |
| Bilezikian 2013^32^ | Rosiglitazone vs. metformin | SAEs | F:5 | F:114 | F:1 | F:112 | Rosiglitazone 8 mg postmenopausal F (pathological fractures): lumbar spine, wrist (fall), fingers (fall), lower leg (fall), toes (trauma)  Metformin (pathological fractures): wrist (fall) |
| Borges 2011^33^ | Rosiglitazone/metformin**^¶^** vs. metformin | SAEs | 2  F:2  M:0 | 344  F:160  M:184 | 0  F:0  M:0 | 334  F:158  M:176 | Rosiglitazone/metformin: 0  Rosiglitazone/metformin F: 2 ankles  Metformin: 0 |

***Abbreviations:*** *F,* female; *M,* male; *NR,* not report*; SAEs, serious* adverse events; *TZD,* thiazolidinedione.

***Note:* ^¶^** Novel treatment of fixed-dose combination of 2 different classes of AHG; ^§^Multiple fracture per person.

Table S13. Trial outcomes of fracture and bone mineral density

| Study | Treatment groups | Population | Mean age*^‡^* | TZDs Dosage*^‡^*  mg/day | Percentage of changes in bone mineral density level, mean (SD) | | | | | | | |
| --- | --- | --- | --- | --- | --- | --- | --- | --- | --- | --- | --- | --- |
|  |  |  |  |  | **Total body BMD** | | **Hip** | | **Lumbar spine** | | **Femoral neck** | |
|  |  |  |  |  | **TZDs** | **Controls** | **TZDs** | **Controls** | **TZDs** | **Controls** | **TZDs** | **Controls** |
| Grey 2014^3^ | Pioglitazone vs. placebo | T2DM or impaired glucose tolerance | 64.0 | 15-30 | *n*=43  −1.0  (NR) | *n*=43  0.1  (NR) | *n*=43  −1.4  (2.7) | *n*=43  −0.2  (1.7) | *n*=43  −1.1  (3.4) | *n*=43  −0.4  (3.1) | NR | NR |
| Bray 2013^4^ | Pioglitazone vs. placebo | Impaired glucose tolerance with one risk factor of T2DM | 53.0 | 30-45 | NR | NR | NR | NR | *n*=213  −1.4  (13.14) | *n*=228  0.5  (15.10) | NR | NR |
| Bone 2013^5^ | Pioglitazone vs. placebo | Postmenopausal with impaired fasting glucose | 59.0 | 30-45 | *n*=59  −00.80  (NR) | *n*=61  −00.70  (NR) | NR | NR | *n*=59  −0.77  (2.84) | *n*=60  0.20  (2.87) | *n*=58  −0.64  (3.35) | *n*=61  0.21  (3.36) |
| Bilezikian 2013^32^ | Rosiglitazone vs. metformin | Postmenopausal T2DM | 64.0 | 8 | NR | NR | *n*=70  −1.62  (3.230) | *n*=78  −0.72  (3.347) | *n*=70  −1.41  (3.481) | *n*=76  −0.04  (3.653) | *n*=70  −1.47  (4.359) | *n*=78  0.22  (4.522) |
| Borges 2011^33^ | Rosiglitazone/Metformin **^¶^** vs. metformin | T2DM naive to oral AHGs | 51.5 | 4-8 | *n*=87  01.1  (NR) | *n*=87  1.1  (NR) | *n*=87  −1.5  (3.27) | *n*=87  0  (3.27) | *n*=87  −2.1  (5.04) | *n*=87  0.1  (5.04) | *n*=87  −1.4  (3.64) | *n*=87  −0.7  (3.64) |
|  | Female | T2DM naive to oral AHGs | 51.5 | 4-8 | *n*=44  01.0  (NR) | *n*=49  2.0  (NR) | *n*=44  −1.8  (4.05) | *n*=49  0  (4.20) | *n*=44  −2.7  (5.11) | *n*=49  0.2  (5.25) | *n*=44  −1.2  (3.78) | *n*=49  −0.3  (3.92) |
|  | Male | T2DM naive to oral AHGs | 51.5 | 4-8 | *n*=43  0.5  (NR) | *n*=38  0.3  (NR) | *n*=43  −1.0  (2.69) | *n*=38  0.3  (2.84) | *n*=43  −0.7  (5.31) | *n*=38  1.0  (5.55) | *n*=43  −2.2  (3.87) | *n*=38  −1.4  (4.07) |

***Abbreviations:*** *AHGs,* anti-hyperglycaemic drugs; *BMD,* bone mineral density; *mean ± SD,* mean and standard deviation; *mg*/day, milligram pear day; *NR,* not report; *T2DM,* type 2 diabetes mellitus; *TZD,* thiazolidinedione; *vs.,* versus.

***Note:* ^‡^** Mean age and dosages present for active TZDs drugs (pioglitazone or rosiglitazone); **^¶^** Novel treatment of fixed-dose combination of 2 different classes of AHG.

## Meta-analysis and GRADE outcomes

Among 70 trials that evaluated the outcomes of fracture, no significant difference was observed in 62 trials. In two trials, no clinically important differences were observed in patients using TZD across the groups.^24,7^ However, the fracture rates increased significantly in six trials on TZD (3 trials with rosiglitazone^42,41,25^ and 3 trials with pioglitazone).^80,79,37^

Generally, the outcome of fracture occurring during the follow-up period were not serious. However, the consequences of these events were not stated in most trials. A majority of non-serious events were not considered to be related to pioglitazone use. However, a causal relationship could not be ruled out in a minority of the events that occurred with TZD use in five trials.^20,16,12,2,6^

### TZDs and fracture risk by severity and mechanism

The rate of high energy, stress, or pathological fracture events did not increase with TZD use compared with that without TZD use. Both non-serious (RR, 1.33; 95% CI, 1.18–1.48; P < 0.00001) and serious fracture (RR, 1.30; 95% CI, 1.05–1.62; P = 0.02) increased with TZD use compared with that without TZD use specifically from low-energy fracture from falls (RR, 1.51; 95% CI, 1.22–1.88; P = 0.0002), using a fixed-effect model (Figure S2). However, only non-serious (RR, 1.22; 95% CI, 1.01–1.47; P = 0.04) and low energy (RR, 1.51; 95% CI, 1.21 to 1.87; P = 0.0002), but not serious, events increased in the random-effect model analysis (Figure S3). The GRADE scores were moderate (Table S14).


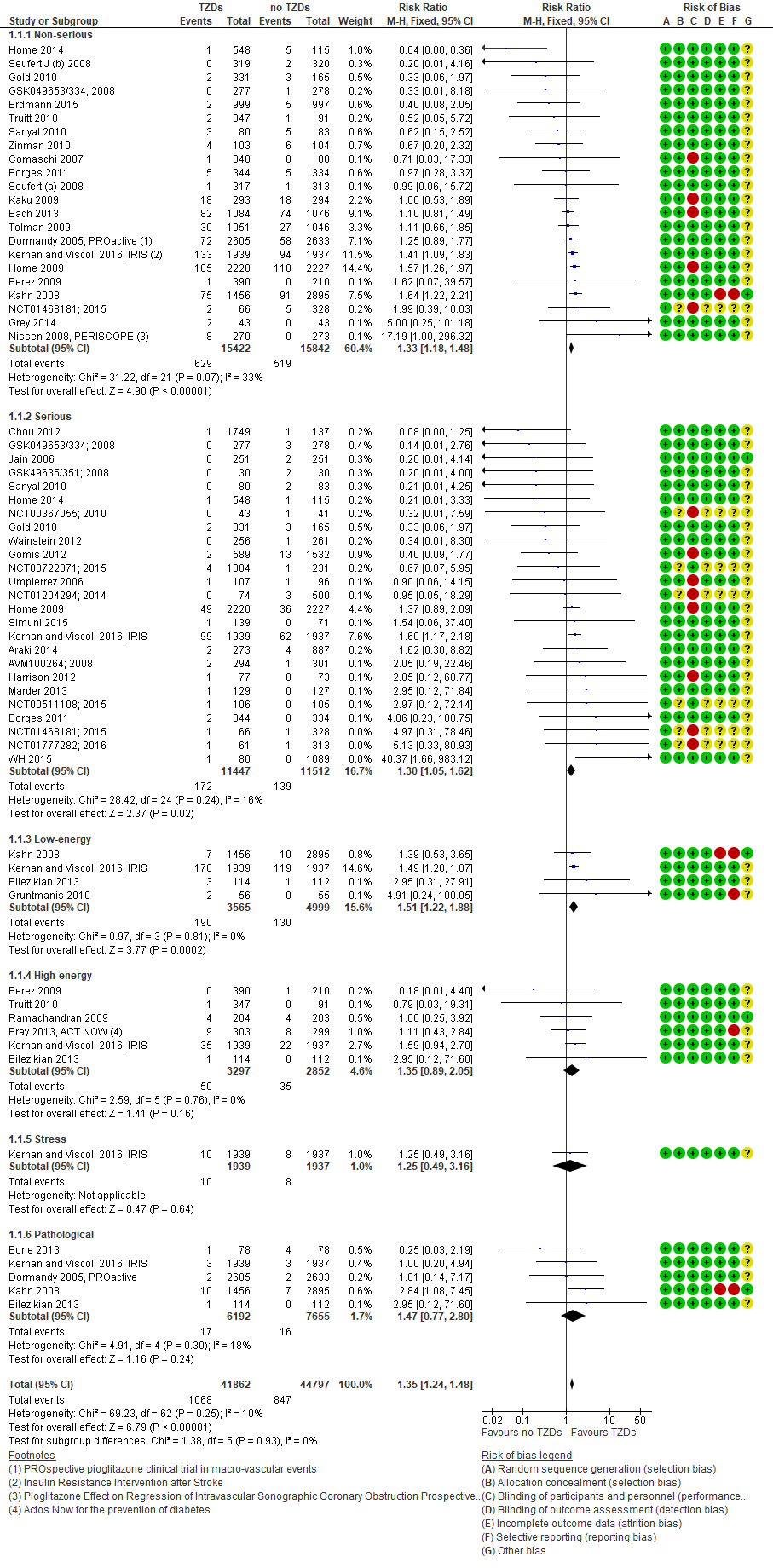


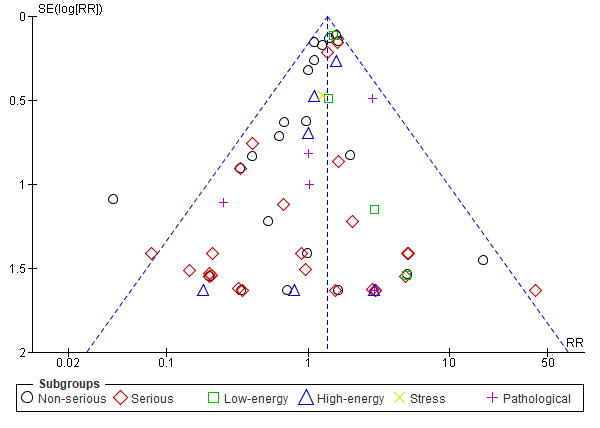


Figure S2. Forest and funnel plot of TZDs and fracture by severity and mechanism, Fixed-effect model.


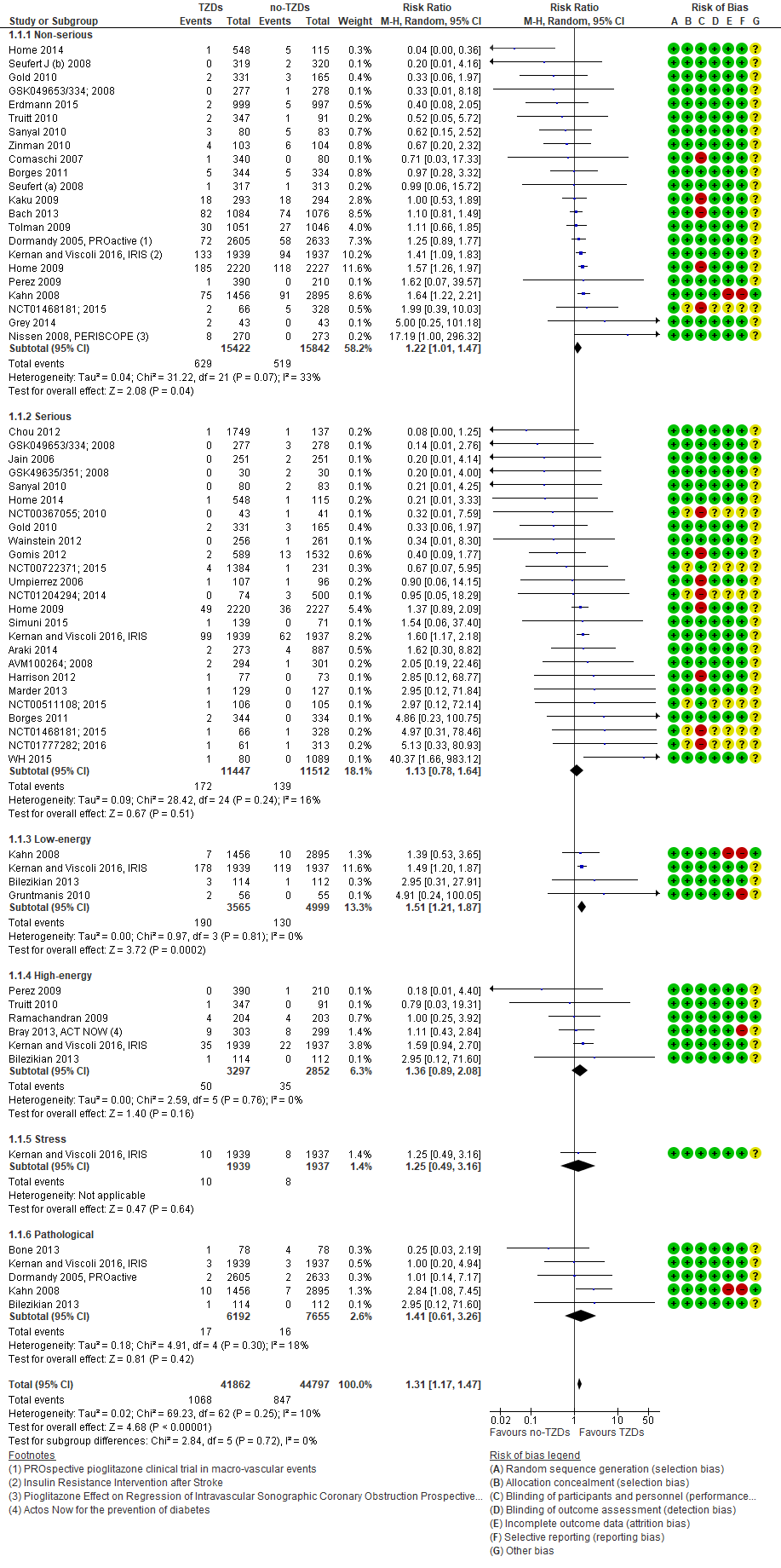


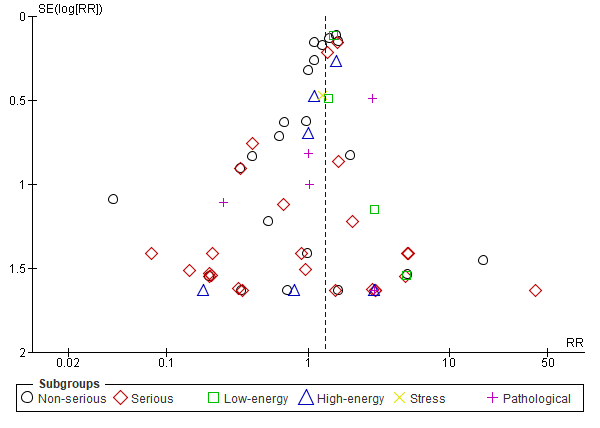


Figure S3. Forest and funnel plot of TZDs and fracture by severity and mechanism, Random-effect model.

Table S14. GRADE evidence profile of TZDs and fracture by severity and mechanism

| **Certainty assessment** | | | | | | | **Summary of findings** | | | | |
| --- | --- | --- | --- | --- | --- | --- | --- | --- | --- | --- | --- |
| **№ of participants (trials)** | **Risk of bias** | **Inconsistency** | **Indirectness** | **Imprecision** | **Publication bias** | **Overall certainty of evidence** | **Study event rates (%)** | | **Relative effect (95% CI)** | **Anticipated absolute effects** | |
|  |  |  |  |  |  |  | **With placebo / controls** | **With TZDs** |  | **Risk with placebo / controls** | **Risk difference with TZDs** |
| **TZDs and fracture by severity and mechanism (follow-up: range 12 to 261 weeks)** | | | | | | | | | | | |
| 86659 (44 RCTs) | serious^a^ | not serious | not serious | not serious | none | ⨁⨁⨁◯ MODERATE | 847/44797 (1.9%) | 1068/41862 (2.6%) | RR 1.36 (1.24 to 1.48) | 2 per 100 | 1 more per 100 (from 0 fewer to 1 more) |
| **Non-serious (follow-up: range 12 to 261 weeks)** | | | | | | | | | | | |
| 31264 (22 RCTs) | serious^a^ | not serious | not serious | very serious^b^ | none | ⨁◯◯◯  VERY LOW | 519/15842 (3.3%) | 629/15422 (4.1%) | RR 1.33 (1.19 to 1.49) | 3 per 100 | 1 more per 100 (1 more to 2 more) |
| **Serious (follow-up: range 24 to 261 weeks)** | | | | | | | | | | | |
| 22959 (25 RCTs) | serious^a^ | not serious | not serious | serious^c^ | none | ⨁⨁◯◯ LOW | 139/11512 (1.2%) | 172/11447 (1.5%) | RR 1.30 (1.05 to 1.62) | 1 per 100 | 0 fewer per 100 (0 fewer to 1 more) |
| **Low energy (follow-up: range 24 to 261 weeks)** | | | | | | | | | | | |
| 8564 (4 RCTs) | not serious | not serious | not serious | serious^d^ | none | ⨁⨁⨁◯ MODERATE | 130/4999 (2.6%) | 190/3565 (5.3%) | RR 1.51 (1.22 to 1.88) | 3 per 100 | 1 more per 100 (1 more to 2 more) |
| **High energy (follow-up: range 24 to 162 weeks)** | | | | | | | | | | | |
| 6149 (6 RCTs) | not serious | not serious | not serious | very serious^e^ | none | ⨁⨁◯◯ LOW | 35/2852 (1.2%) | 50/3297 (1.5%) | RR 1.35 (0.89 to 2.05) | 1 per 100 | 0 fewer per 100 (0 fewer to 1 more) |
| **Stress (follow-up: median 261 weeks)** | | | | | | | | | | | |
| 3876 (1 RCT) | not serious | not serious | not serious | very serious^f^ | none | ⨁⨁◯◯ LOW | 8/1937 (0.4%) | 10/1939 (0.5%) | RR 1.25 (0.49 to 3.16) | 0 per 100 | 0 fewer per 100 (0 fewer to 1 more) |
| **Pathological (follow-up: range 76 to 261 weeks)** | | | | | | | | | | | |
| 13847 (5 RCTs) | not serious | not serious | not serious | very serious^g^ | none | ⨁⨁◯◯ LOW | 16/7655 (0.2%) | 17/6192 (0.3%) | RR 1.47 (0.77 to 2.80) | 0 per 100 | 0 fewer per 100 (0 fewer to 0 fewer) |

***Abbreviations:*** **CI:** Confidence interval; **RCTs:** randomized controlled trials; **RR:** Risk ratio; **TZD:** thiazolidinedione.

***Note:*** The GRADE scores were from the fixed-effect model.

#### GRADE evidence

**a.** Thirteen trials with large (14.4% and 9.1%) and small weights (4.4%, 2.2%, 0.9%, 0.2%, 0.2%, 0.1%, 0.1%, 0.1%, 0.1%, 0.0%, and 0.0%) with the overall (effect estimate) EE rated as a high risk of bias due to a lack of blinding (open-label design) out of 44 trials, five of which were unpublished RCTs.

**b.** The overall imprecision was precise with a significant effect size difference (P < 0.00001). However, all trials reported overlapping CIs, in which five trials reported wide CIs. The 95% CI was consistent with the possibility for a substantial negative effect exceeding the minimal important difference (MID), including only 1,148 fracture events with a large sample size.

**c.** The overall imprecision was precise with a significant effect size difference (P = 0.02). However, all trials reported overlapping CIs, in which 11 trials reported wide CIs. The 95% CI was not consistent with the possibility for a substantial negative effect exceeding the MID, including only 301 fracture events with a large sample size.

**d.** The overall imprecision was precise with a significant effect size difference (P = 0.0002). However, all trials reported overlapping CIs, in which two trials reported wide CIs. The 95% CI was not consistent with the possibility for a substantial negative effect exceeding the MID, including only 320 fracture events with a large sample size.

**e.** The overall imprecision was precise with no significant effect size difference (P = 0.16). However, all trials reported overlapping CIs, in which two trials reported wide CIs. The 95% CI was not consistent with the possibility for a substantial negative effect exceeding the MID, including only 85 fracture events with a large sample size.

**f.** The overall imprecision was precise with no significant effect size difference (P = 0.64). However, all trials reported overlapping and narrow CIs. The 95% CI was not consistent with the possibility for a substantial negative effect exceeding the MID, including only 18 fracture events with a large sample size.

**g.** The overall imprecision was precise with no significant effect size difference (P = 0.24). However, all trials reported overlapping CIs, in which one trial reported wide CIs. The 95% CI was not consistent with the possibility for a substantial negative effect exceeding the MID, including only 33 fracture events with a large sample size.

In Summary, the overall certainty of the pooled EE had a moderate imprecision with a strong significant effect size difference in the level of evidence (P < 0.00001). However, the majority of trials reported overlapping CIs, in which 21 trials reported wide CIs. Six trials (14.6%, 11.5%, 7.5%, 0.6%, 0.1%, and 0.0%) did not cross the line of no difference (1), in which reports a significant association fracture in the direction of TZDs favor versus no-TZDs, and one small trial (1.0%) favor no-TZDs versus TZDs. The 95% CI was consistent with the possibility for a substantial negative effect exceeding the MID, including 1,915 fracture events with a large sample size. There was no evidence of inconsistency, statistically significant heterogeneity (P = 0.25; I^2^ = 10%), or subgroup difference across trials (P = 0.93; I^2^ = 0%), in the fixed-effect model. However, the random-effect model, with TZDs versus on-TZDs, predicts a significant difference across trials (T^2^ = 0.02%). Such a variation indicates an actual difference in the effect of TZDs in each trial as well as sampling variability is due to chance, which could be explained by the differences in trial populations (such as age of patients), interventions received (such as dose of drug), follow-up length, or other factors including stroke-related disability risk as a recurrent fall. Majority of trials had a low risk of bias (68.2%). The overall magnitude of attrition and reporting bias did not affect our results, as these trials were double-blind RCTs. However, the magnitude of effect of some trials suffered from limitations, likely resulting in a biased assessment of the intervention effect due to performance and selection bias (13 trials with open-label design out of 44). The indirectness of the trials indicated a high level of evidence, with individual trial patient, intervention, comparison, outcome, and trial design (PICOS) elements aligning close to the review PICOS. Although our population was restricted to patients with no surgical intervention, our evidence may be regarded as indirect in relation to our broader question of interest. We were uncertain of the negative effects of TZDs in T2DM patients with coronary artery disease, with a two-by-two factorial design trial; the participants were those who underwent a surgical or therapeutic intervention. Finally, there was no evidence of reporting bias. However, three trials reported an outlier outside the pyramid edge due to multiple interventional groups causing wide CIs.

### Pioglitazone and fracture risk by comparator

Use of pioglitazone did not increase the occurrence of fracture (RR, 1.06; 95% CI, 0.76–1.48; P = 0.73) versus placebo or versus controls (RR, 0.98; 95% CI, 0.52–1.81; P = 0.94) using a random-effect model (Figure ‎S4). The GRADE scores were moderate (Table S15).


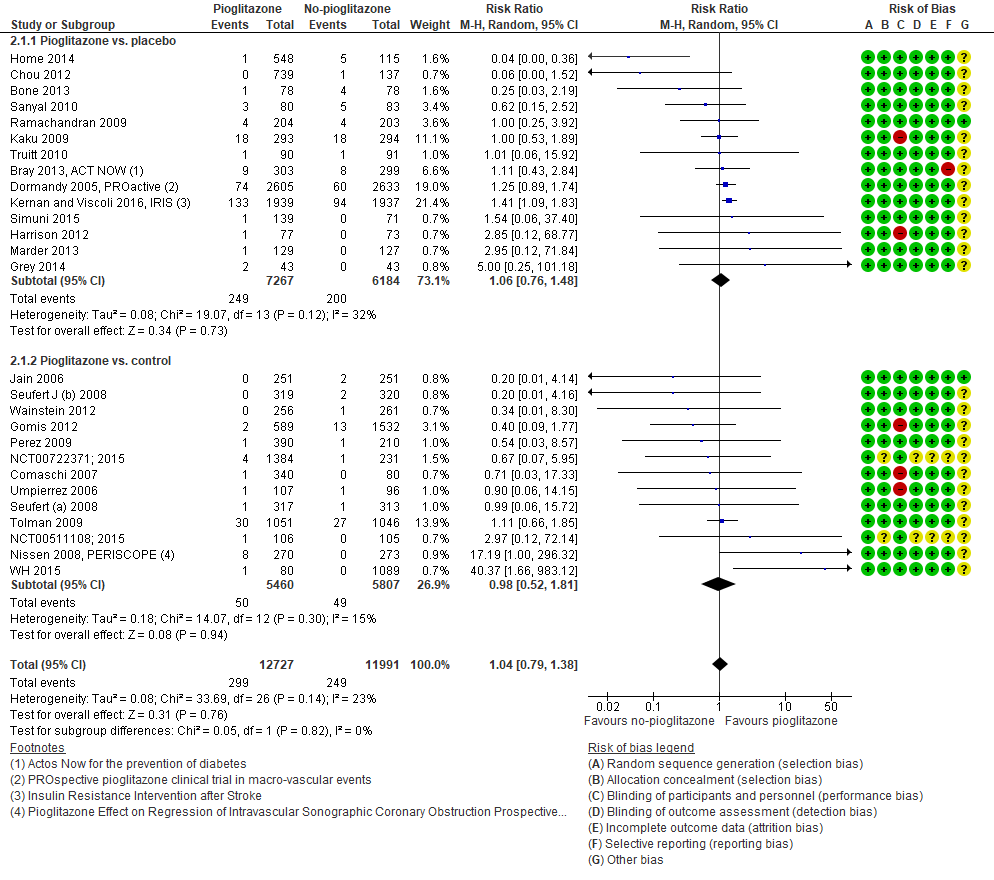


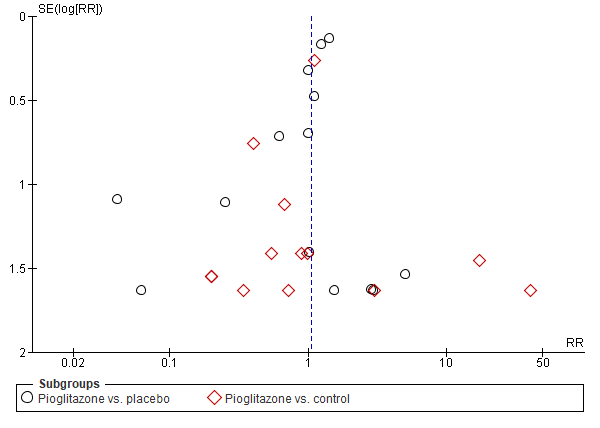


Figure ‎S4. Forest and funnel plot of pioglitazone and fracture by comparator, Random-effect model.

Table S15. GRADE evidence profile of pioglitazone and fracture by comparator

| **Certainty assessment** | | | | | | | **Summary of findings** | | | | |
| --- | --- | --- | --- | --- | --- | --- | --- | --- | --- | --- | --- |
| **№ of participants (trials)** | **Risk of bias** | **Inconsistency** | **Indirectness** | **Imprecision** | **Publication bias** | **Overall certainty of evidence** | **Study event rates (%)** | | **Relative effect (95% CI)** | **Anticipated absolute effects** | |
|  |  |  |  |  |  |  | **With placebo / controls** | **With pioglitazone** |  | **Risk with placebo / controls** | **Risk difference with pioglitazone** |
| **Pioglitazone and fracture by comparator (follow-up: range 24 to 251 weeks)** | | | | | | | | | | | |
| 24718 (27 RCTs) | serious^a^ | not serious | not serious | not serious | none | ⨁⨁⨁◯ MODERATE | 249/11991 (2.1%) | 299/12727 (2.3%) | RR 1.19 (1.01 to 1.40) | 2 per 100 | 0 fewer per 100 (0 fewer to 1 more) |
| **Pioglitazone vs. placebo (follow-up: range 26 to 251 weeks)** | | | | | | | | | | | |
| 13451 (14 RCTs) | serious^a^ | not serious | not serious | serious^b^ | none | ⨁⨁◯◯ LOW | 200/6184 (3.2%) | 249/7267 (3.4%) | RR 1.21 (1.01 to 1.45) | 3 per 100 | 1 more per 100 (0 fewer to 1 more) |
| **Pioglitazone vs. controls (follow-up: range 24 to 251 weeks)** | | | | | | | | | | | |
| 11267 (13 RCTs) | serious^a^ | not serious | not serious | very serious^c^ | none | ⨁◯◯◯  VERY LOW | 49/5807 (0.8%) | 50/5460 (0.9%) | RR 1.08 (0.73 to 1.59) | 1 per 100 | 0 fewer per 100 (0 fewer to 0 fewer) |

***Abbreviations:*** **CI:** Confidence interval; **RCTs:** randomized controlled trials; **RR:** Risk ratio.

***Note:*** The GRADE scores were from the fixed-effect model.

#### GRADE evidence

**a.** Five trials with a small weight (pioglitazone vs. placebo [7.1% and 0.2%] and (pioglitazone vs. control [2.8%, 0.4%, and 0.3%] with the overall EE rated as high risk of bias due to lack of blinding (open-label design) out of 27 trials.

**b.** The overall imprecision was precise with a significant effect size difference (P = 0.04). However, all trials reported overlapping CIs, in which five trials reported wide CIs. The 95% CI was consistent with the possibility for a substantial negative effect exceeding the MID, including only 449 fracture events with a large sample size.

**c.** The overall imprecision was precise with no significant effect size difference (P = 0.70). However, all trials reported overlapping CIs, in which six trials reported wide CIs. The 95% CI was not consistent with the possibility for a substantial negative effect exceeding the MID, including only 99 fracture events with a large sample size.

In summary, the overall certainty of the pooled EE had a moderate imprecision with a significant effect size difference in the level of evidence (P = 0.04). However, the majority of trials reported overlapping CIs, in which 11 trials reported wide CIs. The largest (37%) and smallest trials (0.0%) did not cross the line of no difference (1), in which reports a significant association of fracture in the direction of pioglitazone favor across comparators, and one small trial (3.2%) in the direction of non-pioglitazone favor across pioglitazone. The 95% CI was consistent with the possibility for a substantial negative effect exceeding the MID, including 548 fracture events with a large sample size. There was no evidence of inconsistency, statistically significant heterogeneity (P = 0.14; I^2^ = 23%), or subgroup difference (P = 0.60; I^2^ = 0%). Majority of trials had a low risk of bias (89.2%). The overall magnitude of reporting bias did not affect our results, as these trials were double-blind RCTs. However, the magnitude of effect of some trials that suffered from limitations likely resulted in a biased assessment of the intervention effect due to performance and selection biases (five trials with open-label design out of 27). The indirectness of the trials indicated a high level of evidence, with an individual trial PICOS element aligning close to the review PICOS. Finally, there was no evidence of reporting bias. However, two trials reported an outlier outside the pyramid edge due to multiple interventional groups causing wide CIs.

### Pioglitazone and fracture by severity, mechanism, and comparators

The rate of non-serious, serious, high energy, stress, or pathological fracture events did not increase with pioglitazone use versus placebo. However, the use of pioglitazone increased the occurrence of low-energy fracture from falls (RR, 1.49; 95% CI, 1.20–1.87; P = 0.0004) versus placebo using a random-effect model (Figure S5). The GRADE scores were moderate (Table S16).


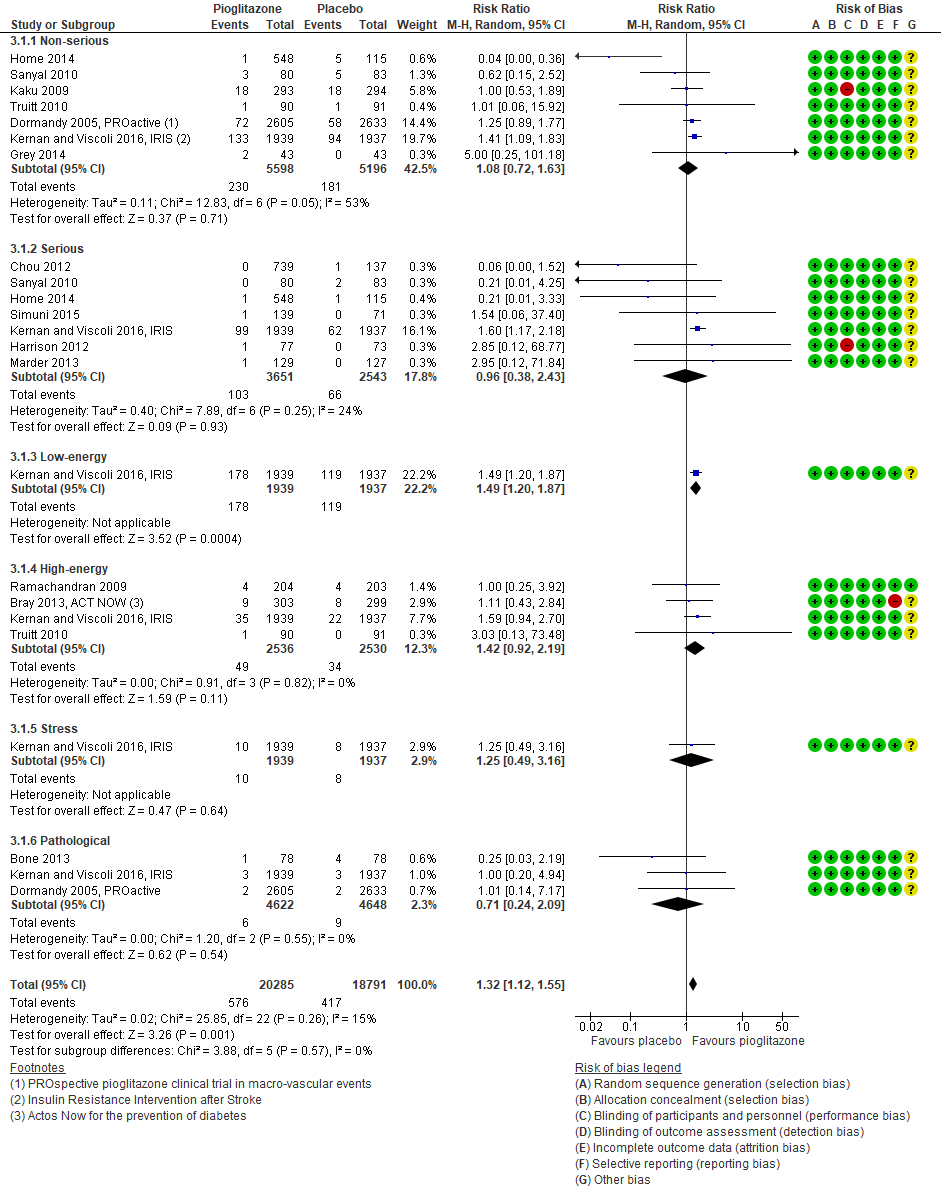


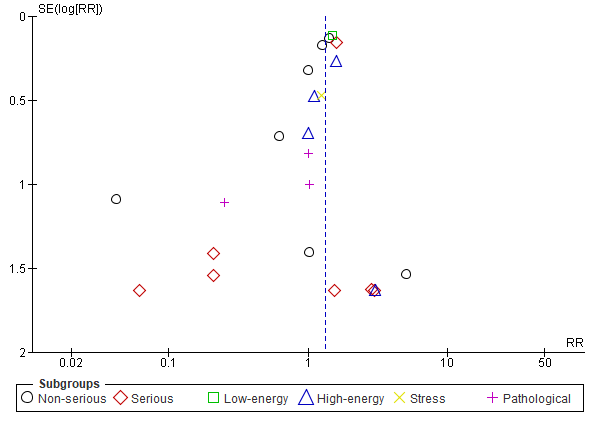


Figure S5. Forest and funnel plot of pioglitazone and fracture by severity and mechanism versus placebo, Random-effect model.

However, use of pioglitazone did not increase the incidence of non-serious (RR, 1.28; 95% CI, 0.81 to 2.01; P = 0.29), serious (RR, 0.71; 95% CI, 0.32 to 1.55; P = 0.38), high energy (RR, 0.18; 95% CI, 0.73 to 1.56; P = 0.75) fracture versus controls using fixed-effect model (Figure S6).


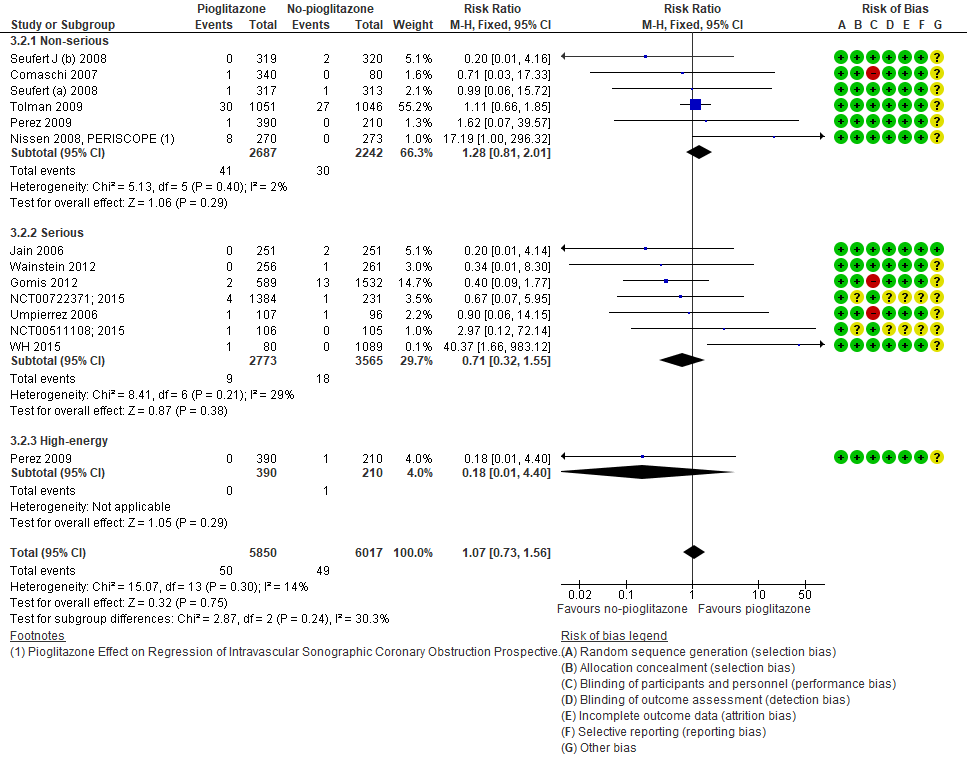


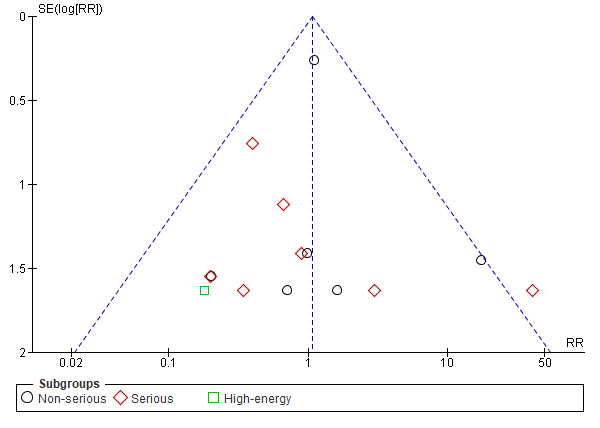


Figure S6. Forest and funnel plot of pioglitazone and fracture by severity and mechanism versus control, Fixed-effect model.

However, use of pioglitazone did not increase the incidence of non-serious (RR, 1.15; 95% CI, 0.06 to 2.02; P = 0.62), serious (RR, 0.87; 95% CI, 0.28 to 2.74; P = 0.82), high energy (RR, 0.18; 95% CI, 0.01 to 4.40; P = 0.29) fracture versus controls using random-effect model (Figure S7).


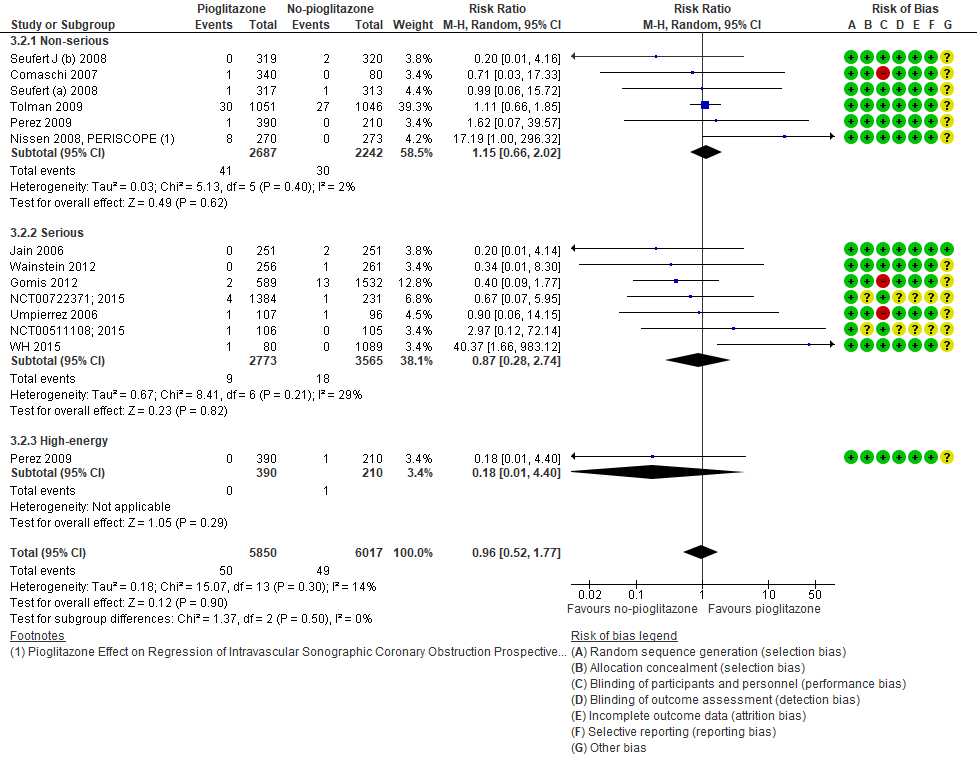


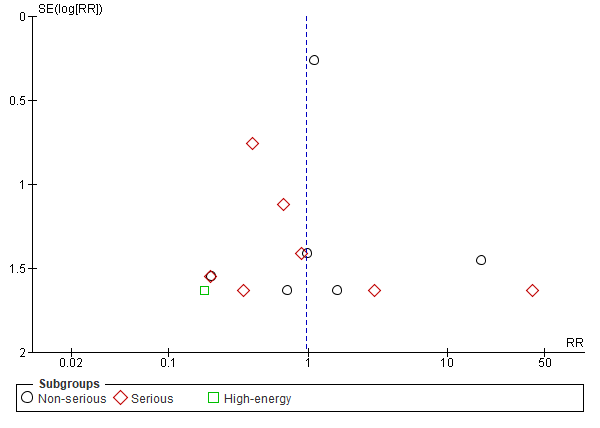


Figure S7. Forest and funnel plot of pioglitazone and fracture by severity and mechanism versus control, Random-effect model.

Table S16. GRADE evidence profile of pioglitazone and fracture by severity, mechanism, and comparators

| **Certainty assessment** | | | | | | | **Summary of findings** | | | | |
| --- | --- | --- | --- | --- | --- | --- | --- | --- | --- | --- | --- |
| **№ of participants (trials)** | **Risk of bias** | **Inconsistency** | **Indirectness** | **Imprecision** | **Publication bias** | **Overall certainty of evidence** | **Study event rates (%)** | | **Relative effect (95% CI)** | **Anticipated absolute effects** | |
|  |  |  |  |  |  |  | **With placebo / controls** | **With Pioglitazone** |  | **Risk with placebo / controls** | **Risk difference with pioglitazone** |
| **Pioglitazone and fracture by severity, mechanism, and comparator (placebo) (follow-up: range 24 to 251 weeks)** | | | | | | | | | | | |
| 39076 (14 RCTs) | serious^a^ | not serious | not serious | not serious | none | ⨁⨁⨁◯ MODERATE | 417/18791 (2.2%) | 576/20285 (2.8%) | RR 1.36 (1.20 to 1.53) | 2 per 100 | 1 more per 100 (from 0 fewer to 1 more) |
| **Non-serious** | | | | | | | | | | | |
| 10794 (7 RCTs) | serious^a^ | not serious | not serious | serious^b^ | none | ⨁⨁◯◯ LOW | 181/5196 (3.5%) | 230/5598 (4.1%) | RR 1.25 (1.03 to 1.51) | 3 per 100 | 1 more per 100 (from 0 fewer to 2 more) |
| **Serious** | | | | | | | | | | | |
| 6194 (7 RCTs) | serious^a^ | not serious | not serious | very serious^c^ | none | ⨁◯◯◯  VERY LOW | 66/2543 (2.6%) | 103/3651 (2.8%) | RR 1.48 (1.10 to 1.98) | 3 per 100 | 1 more per 100 (from 0 fewer to 3 more) |
| **Low energy** | | | | | | | | | | | |
| 3876 (1 RCT) | not serious | not serious | not serious | very serious^d^ | none | ⨁⨁◯◯ LOW | 119/1937 (6.1%) | 178/1939 (9.2%) | RR 1.49 (1.20 to 1.87) | 6 per 100 | 3 more per 100 (from 1 more to 5 more) |
| **High energy** | | | | | | | | | | | |
| 5066 (4 RCTs) | not serious | not serious | not serious | very serious^e^ | none | ⨁⨁◯◯ LOW | 34/2530 (1.3%) | 49/2536 (1.9%) | RR 1.43 (0.93 to 2.20) | 1 per 100 | 1 more per 100 (from 0 fewer to 2 more) |
| **Stress** | | | | | | | | | | | |
| 3876 (1 RCT) | not serious | not serious | not serious | very serious^f^ | none | ⨁⨁◯◯ LOW | 8/1937 (0.4%) | 10/1939 (0.5%) | RR 1.25 (0.49 to 3.16) | 0 per 100 | 0 fewer per 100 (from 0 fewer to 1 more) |
| **Pathological** | | | | | | | | | | | |
| 9270 (3 RCTs) | not serious | not serious | not serious | very serious^g^ | none | ⨁⨁◯◯ LOW | 9/4648 (0.2%) | 6/4622 (0.1%) | RR 0.67 (0.24 to 1.87) | 0 per 100 | 0 fewer per 100 (from 0 fewer to 0 fewer) |
| **Pioglitazone and fracture by severity, mechanism, and comparator (controls) (follow-up: range 24 to 251 weeks)** | | | | | | | | | | | |
| 11867 (13 RCTs) | serious^d^ | not serious | not serious | very serious^h^ | none | ⨁◯◯◯  VERY LOW | 49/6017 (0.8%) | 50/5850 (0.9%) | RR 1.07 (0.73 to 1.56) | 1 per 100 | 0 fewer per 100 (from 0 fewer to 0 fewer) |
| **Non-serious** | | | | | | | | | | | |
| 4929 (6 RCTs) | serious^d^ | not serious | not serious | very serious^i^ | none | ⨁◯◯◯  VERY LOW | 30/2242 (1.3%) | 41/2687 (1.5%) | RR 1.28 (0.81 to 2.01) | 1 per 100 | 0 fewer per 100 (from 0 fewer to 1 more) |
| **Serious** | | | | | | | | | | | |
| 6338 (7 RCTs) | serious^d^ | not serious | not serious | very serious^j^ | none | ⨁◯◯◯  VERY LOW | 18/3565 (0.5%) | 9/2773 (0.3%) | RR 0.71 (0.32 to 1.55) | 1 per 100 | 0 fewer per 100 (from 0 fewer to 0 fewer) |
| **High energy** | | | | | | | | | | | |
| 600 (1 RCT) | not serious | not serious | not serious | very serious^k^ | none | ⨁⨁◯◯ LOW | 1/210 (0.5%) | 0/390 (0.0%) | RR 0.18 (0.01 to 4.40) | 0 per 100 | 0 fewer per 100 (from 0 fewer to 2 more) |

***Abbreviations:*** **CI:** Confidence interval; **RCTs:** randomized controlled trials; **RR:** Risk ratio.

***Note:*** The GRADE scores were from the fixed-effect model.

#### GRADE evidence

**a.** Two trials with a small weight (pioglitazone vs. placebo [4.2% and 0.1%] with the overall EE rated as a high risk of bias due to a lack of blinding (open-label design) out of 27 trials.

**b.** The overall imprecision was precise with a significant effect size difference (P = 0.02). However, all trials reported overlapping CIs, in which two trials reported wide CIs. The 95% CI was consistent with the possibility for a substantial negative effect exceeding the MID, including only 411 fracture events with a large sample size.

**c.** The overall imprecision was precise with a significant effect size difference (P = 0.010). However, all trials reported overlapping CIs, in which three trials reported wide CIs. The 95% CI was not consistent with the possibility for a substantial negative effect exceeding the MID, including only 169 fracture events with a large sample size.

**d.** The overall imprecision was precise with a significant effect size difference (P = 0.0004). However, all trials reported overlapping and narrow CIs. The 95% CI was not consistent with the possibility for a substantial negative effect exceeding the MID, including only 297 fracture events with a large sample size.

**e.** The overall imprecision was precise with no significant effect size difference (P = 0.10). However, all trials reported overlapping CIs, in which one trial reported wide CIs. The 95% CI was not consistent with the possibility for a substantial negative effect exceeding the MID, including only 83 fracture events with a large sample size.

**f.** The overall imprecision was precise with no significant effect size difference (P = 0.64). However, all trials reported overlapping and narrow CIs. The 95% CI was not consistent with the possibility for a substantial negative effect exceeding the MID, including only 18 fracture events with a large sample size.

**g.** The overall imprecision was precise with no significant effect size difference (P = 0.44). However, all trials reported overlapping and narrow CIs. The 95% CI was not consistent with the possibility for a substantial negative effect exceeding the MID, including only 15 fracture events with a large sample size.

**h.** Three trials with a small weight (pioglitazone vs. controls [14.7%, 2.2%, and 1.6%] with the overall EE rated as a high risk of bias due to a lack of blinding (open-label design).

**i.** The overall imprecision was precise with no significant effect size difference (P = 0.29). However, all trials reported overlapping CIs, in which four trials reported wide CIs. The 95% CI was not consistent with the possibility for a substantial negative effect exceeding the MID, including only 71 fracture events with a large sample size.

**j.** The overall imprecision was precise with no significant effect size difference (P = 0.38). However, all trials reported overlapping CIs, in which three trials reported wide CIs. The 95% CI was not consistent with the possibility for a substantial negative effect exceeding the MID, including only 27 fracture events with a large sample size.

**k.** The overall imprecision was precise with no significant effect size difference (P = 0.29). However, all trials reported overlapping and narrow CIs. The 95% CI was not consistent with the possibility for a substantial negative effect exceeding the MID, including only one fracture event with a small sample size (600).

In summary, the overall certainty of the pooled EE had a moderate imprecision with a significant effect size difference in the level of evidence (P < 0.00001) in trials that compared pioglitazone with a placebo, but not with active comparators (P = 0.75). However, the majority of trials reported overlapping CIs, in which 11 trials reported wide CIs. The largest (28%) and small trials (0.1%) did not cross the line of no difference (1), in which reports a significant association of fracture in the direction of pioglitazone favor across comparators, and one small trial (1.9%) in the direction of non-pioglitazone favor across pioglitazone. The 95% CI was consistent with the possibility for a substantial negative effect exceeding the MID, including 1,092 fracture events with a large sample size. There was no evidence of inconsistency, statistically significant heterogeneity (P = 0.26 and I^2^ = 15% across placebo trials vs. P = 0.30 and I^2^ = 14% across controls trials), or subgroup difference (P = 0.59 and I^2^ = 0% across placebo trials; P = 0.24 and I^2^ = 30.3% across controls trials), in the fixed-effect model analysis. However, the random-effect model, with pioglitazone versus placebo, predicted a significant difference across trials (T^2^ = 0.02%). As such a variation indicates an actual difference in pioglitazone effect in each trial as well as sampling variability is due to chance, it could be explained by the differences in trial populations (such as age of patients), interventions received (such as dose of drug), follow-up length, or other factors including stroke-related disability risk as a recurrent fall. Majority of trials had a low risk of bias (95.7%). The overall magnitude of reporting bias did not affect our results, as these trials were double-blind RCTs. However, the magnitude of effect of some trials that suffered from limitations likely resulted in a biased assessment of the intervention effect due to performance and selection biases (five trials with open-label design out of 27). The indirectness of the trials indicated a high level of evidence, with an individual trial PICOS element aligning close to the review PICOS. Finally, there was no evidence of reporting bias. However, one trial reported an outlier outside the pyramid edge due to multiple interventional groups causing wide CIs.

### Pioglitazone and fracture by follow-up duration

The cumulative use of pioglitazone for more than 104 weeks increased the occurrence of fracture (RR, 1.23; 95% CI, 1.03 to 1.46; P = 0.02) compared with no pioglitazone use, as determined using a fixed-effect model (Figure S8). However, the cumulative use of pioglitazone for less than 24 weeks, or between 24 and 52 weeks, or between 52 and 104 weeks did not increase the occurrence of fracture compared with no pioglitazone use. The GRADE scores were moderate (Table S17).


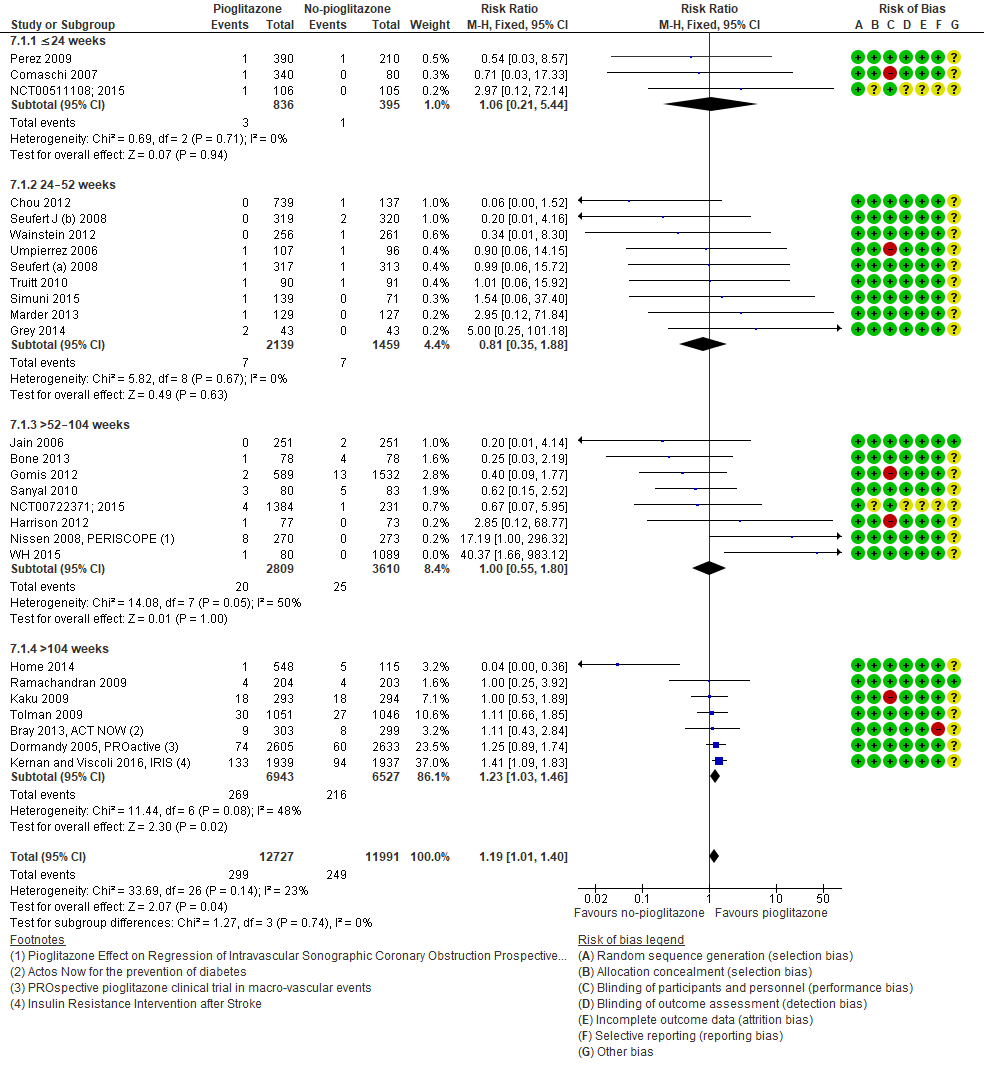


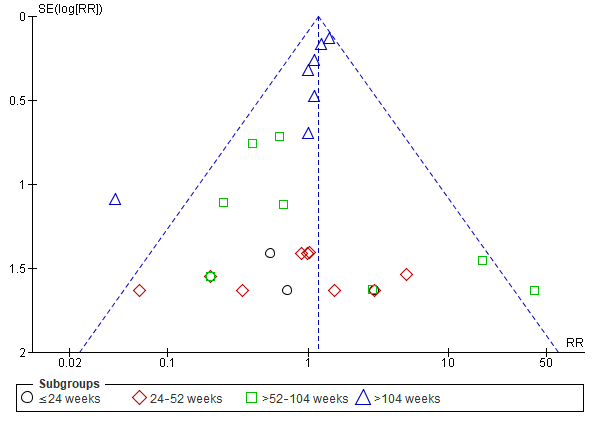


Figure S8. Forest and funnel plot of pioglitazone and fracture by follow-up duration, Fixed-effect model.

However, the cumulative use of pioglitazone for less than 24 weeks or for more than 104 weeks did not increase the occurrence of fracture (RR, 1.04; 95% CI, 1.79 to 1.38; P = 0.76) versus non-pioglitazone using random-effect model (Figure S9).


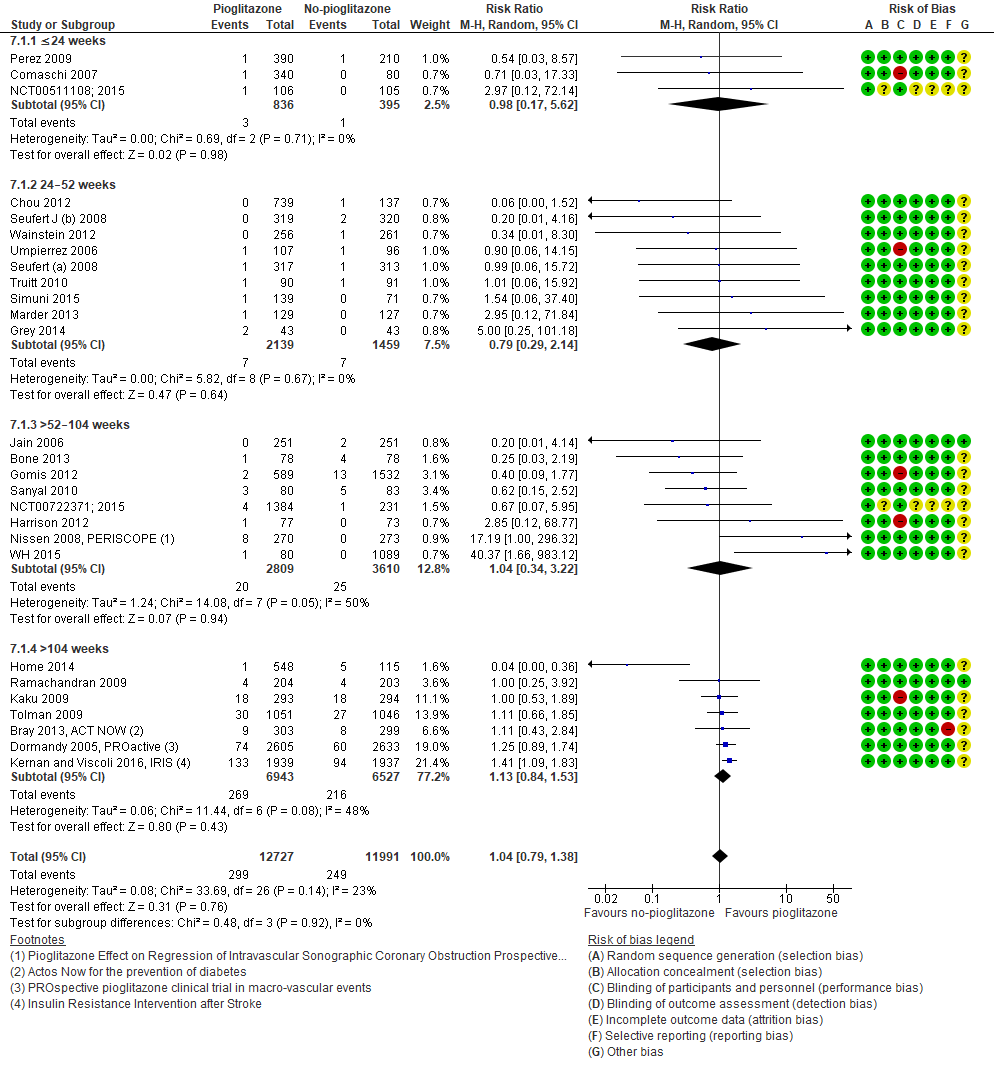


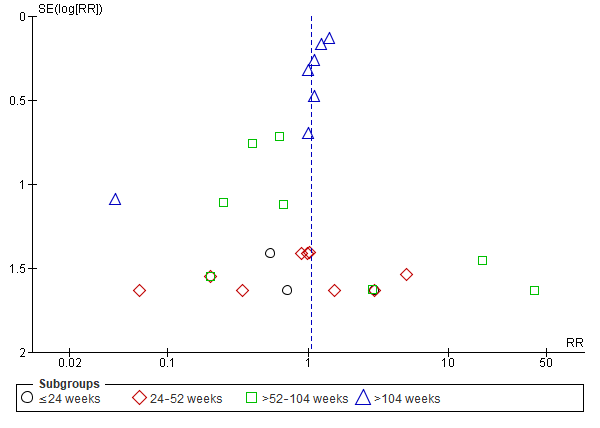


Figure S9. Forest and funnel plot of pioglitazone and fracture by follow-up duration, Random-effect model.

Table S17. GRADE evidence profile of pioglitazone and fracture by follow-up duration

| **Certainty assessment** | | | | | | | **Summary of findings** | | | | |
| --- | --- | --- | --- | --- | --- | --- | --- | --- | --- | --- | --- |
| **№ of participants (trials)** | **Risk of bias** | **Inconsistency** | **Indirectness** | **Imprecision** | **Publication bias** | **Overall certainty of evidence** | **Study event rates (%)** | | **Relative effect (95% CI)** | **Anticipated absolute effects** | |
|  |  |  |  |  |  |  | **With placebo / controls** | **With Pioglitazone** |  | **Risk with placebo / controls** | **Risk difference with pioglitazone** |
| **Pioglitazone and fracture and follow-up duration (follow-up: range 24 to 251 weeks)** | | | | | | | | | | | |
| 24718 (27 RCTs) | serious^a^ | not serious | not serious | not serious | none | ⨁⨁⨁◯ MODERATE | 249/11991 (2.1%) | 299/12727 (2.3%) | RR 1.19 (1.01 to 1.40) | 2 per 100 | 0 fewer per 100 (0 fewer to 1 more) |
| **Follow-up duration (≤24 weeks)** | | | | | | | | | | | |
| 1231 (3 RCTs) | serious^a^ | not serious | not serious | very serious^b^ | none | ⨁◯◯◯  VERY LOW | 1/395 (0.3%) | 3/836 (0.4%) | RR 1.06 (0.21 to 5.44) | 0 per 100 | 0 fewer per 100 (0 fewer to 1 more) |
| **Follow-up duration (24 to 52 weeks)** | | | | | | | | | | | |
| 3598 (9 RCTs) | serious^a^ | not serious | not serious | very serious^c^ | none | ⨁◯◯◯  VERY LOW | 7/1459 (0.5%) | 7/2139 (0.3%) | RR 0.81 (0.35 to 1.88) | 0 per 100 | 0 fewer per 100 (0 fewer to 0 fewer) |
| **Follow-up duration (>52 to 104 weeks)** | | | | | | | | | | | |
| 6419 (8 RCTs) | serious^a^ | not serious | not serious | very serious^d^ | none | ⨁◯◯◯  VERY LOW | 25/3610 (0.7%) | 20/2809 (0.7%) | RR 1.00 (0.55 to 1.80) | 1 per 100 | 0 fewer per 100 (0 fewer to 1 more) |
| **Follow-up duration (>104 weeks)** | | | | | | | | | | | |
| 13470 (7 RCTs) | serious^a^ | not serious | not serious | serious^e^ | none | ⨁⨁◯◯ LOW | 216/6527 (3.3%) | 269/6943 (3.9%) | RR 1.23 (1.03 to 1.46) | 3 per 100 | 1 more per 100 (0 fewer to 2 more) |

***Abbreviations:*** **CI:** Confidence interval; **RCTs:** randomized controlled trials; **RR:** Risk ratio.

***Note:*** The GRADE scores were from the fixed-effect model.

#### GRADE evidence

**a.** Five trials with s small weight ([>104 weeks (7.1%)], [>52 to 104 weeks (2.8% and 0.2%)], [≤24 weeks (0.3%)], and [(24 to 52 weeks (0.4%)]) with the overall EE rated as a high risk of bias due to a lack of blinding (open-label design) out of 27 trials.

**b.** The overall imprecision was precise with no significant effect size difference (P = 0.94). However, all trials reported overlapping CIs, in which two trials reported wide CIs. The 95% CI was not consistent with the possibility for a substantial negative effect exceeding the MID, including only four fracture events with a small sample size (1,231).

**c.** The overall imprecision was precise with no significant effect size difference (P = 0.63). However, all trials reported overlapping CIs, in which six trials reported wide CIs. The 95% CI was not consistent with the possibility for a substantial negative effect exceeding the MID, including only 14 fracture events with a large sample size.

**d.** The overall imprecision was precise with no significant effect size difference (P = 1.00). However, all trials reported overlapping CIs, in which three trials reported wide CIs. The 95% CI was not consistent with the possibility for a substantial negative effect exceeding the MID, including only 45 fracture events with a large sample size.

**e.** The overall imprecision was precise with a significant effect size difference (P = 0.02). However, all trials reported overlapping and narrow CIs. The 95% CI was consistent with the possibility for a substantial negative effect exceeding the MID, including only 485 fracture events with a large sample size.

In summary, the overall certainty of the pooled EE had a moderate imprecision with a significant effect size difference in the level of evidence (P = 0.04). However, the majority of trials reported overlapping CIs, in which 11 trials reported wide CIs. The largest (37%) and smallest trials (0.0%) did not cross the line of no difference (1), in which reports a significant association of fracture in the direction of pioglitazone favor across non-pioglitazone, and one small trial (3.2%) in the direction of non-pioglitazone favor across pioglitazone. The 95% CI was consistent with the possibility for a substantial negative effect exceeding the MID, including 548 fracture events with a large sample size. There was no evidence of inconsistency, statistically significant heterogeneity (P = 0.14; I^2^ = 23%), or subgroup difference (P = 0.74; I^2^ = 0%). Majority of trials had a low risk of bias (89.2%). The overall magnitude of reporting bias did not affect our results, as these trials were double-blind RCTs. However, the magnitude of effect of some trials that suffered from limitations likely resulted in a biased assessment of the intervention effect due to performance and selection biases (five trials with an open-label design out of 27). The indirectness of the trials indicated a high level of evidence, with an individual trial PICOS element aligning close to the review PICOS. Finally, there was no evidence of reporting bias. However, two trials reported an outlier outside the pyramid edge due to multiple interventional groups resulting in wide CIs.

### Pioglitazone and fracture by baseline characteristics

The use of pioglitazone increased the occurrence of fracture in nondiabetic individuals (RR, 1.32; 95% CI, 1.05–1.67; P = 0.02) particularly those with a history of stroke or TIA (RR, 1.41; 95% CI, 1.09–1.83; P = 0.008) versus placebo using a fixed-effect model (Figure S10). The GRADE scores were very low (Table S18).


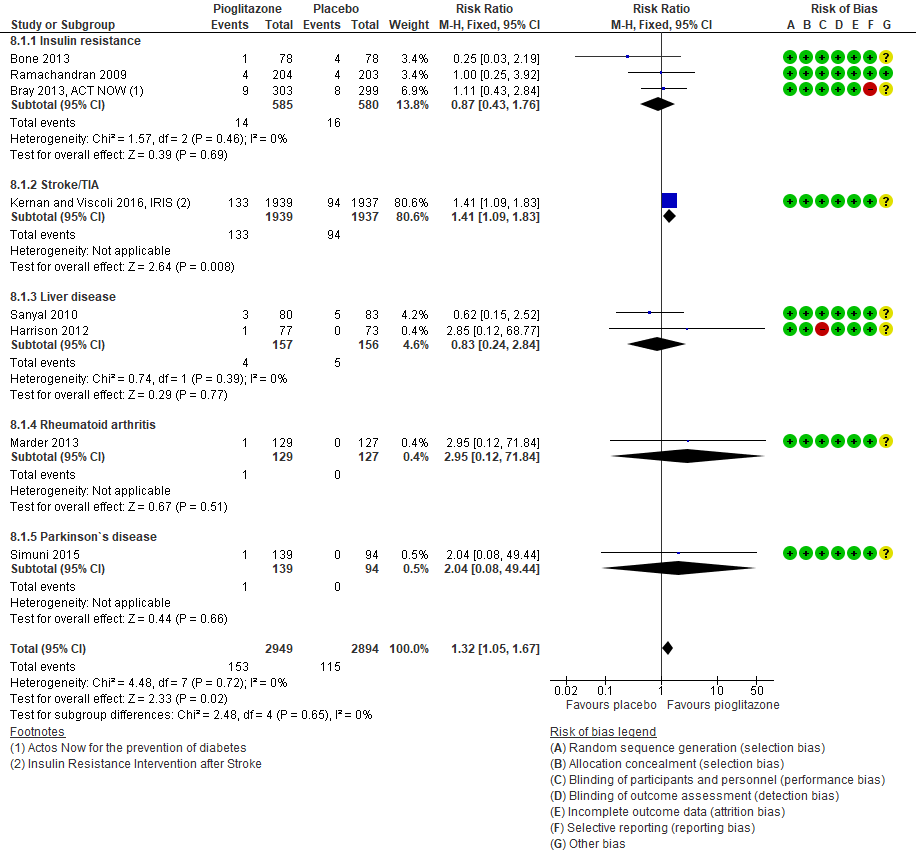


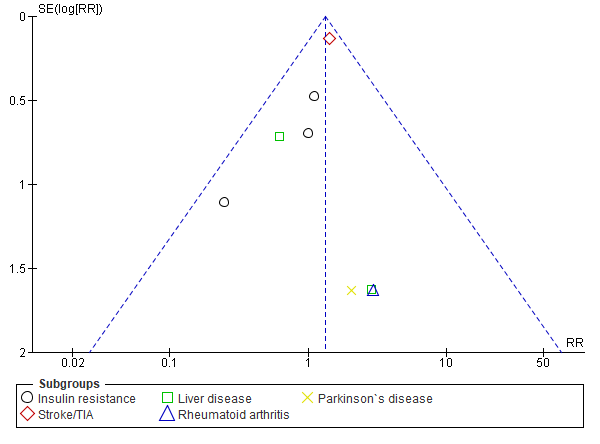


Figure S10. Forest and funnel plot of pioglitazone and fracture in nondiabetic individuals, Fixed-effect model.

The use of pioglitazone increased the occurrence of fracture in nondiabetic individuals (RR, 1.33; 95% CI, 1.05–1.69; P = 0.02) particularly those with a history of stroke or TIA (RR, 1.41; 95% CI, 1.09–1.83; P = 0.008) versus placebo using a random-effect model (Figure S11).


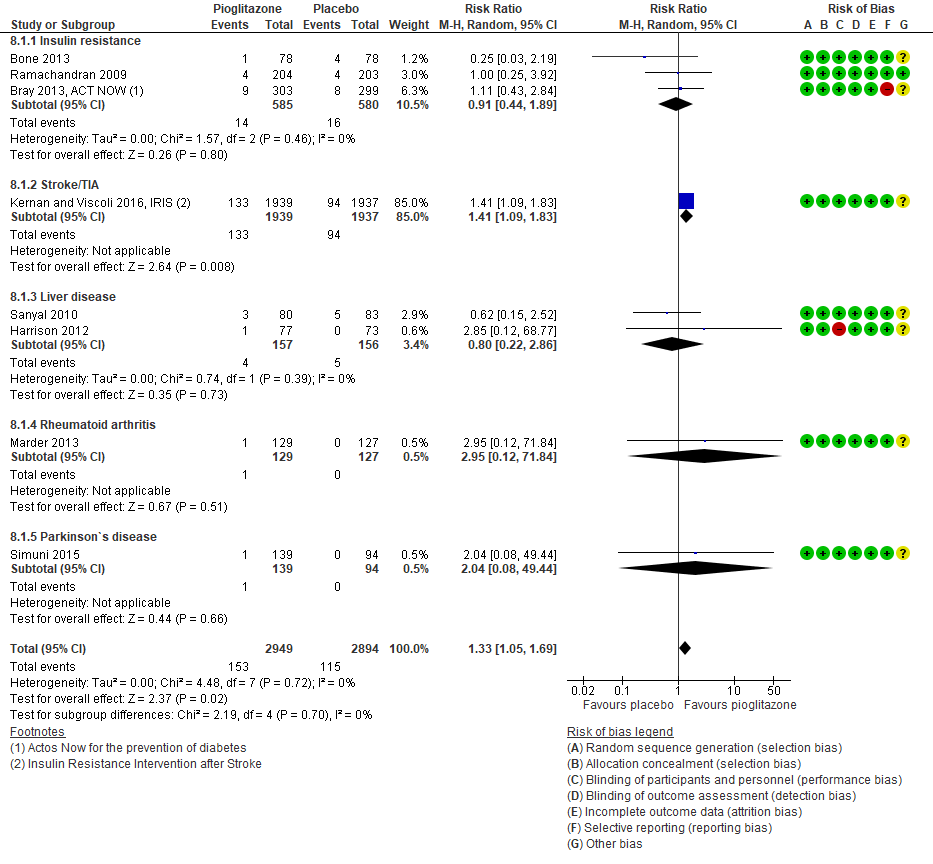


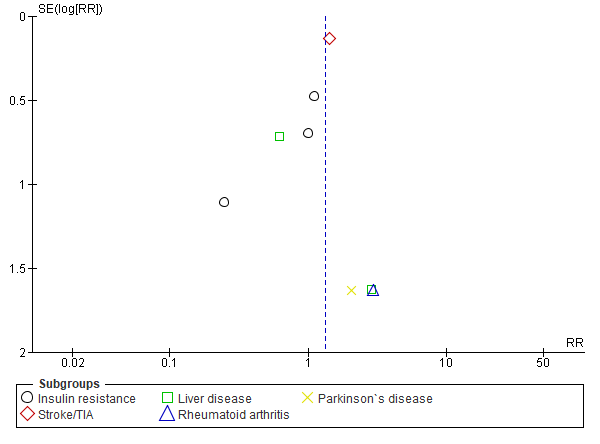


Figure S11. Forest and funnel plot of pioglitazone and fracture in nondiabetic individuals, Random-effect model.

The use of pioglitazone did not increase the occurrence of fracture in T2DM patients at a risk or not at a risk of cardiovascular disease (CVD) (RR, 1.07; 95% CI, 0.86–1.35; P = 0.53) versus non-pioglitazone using a fixed-effect model (Figure S12). The GRADE scores were very low (Table S18).


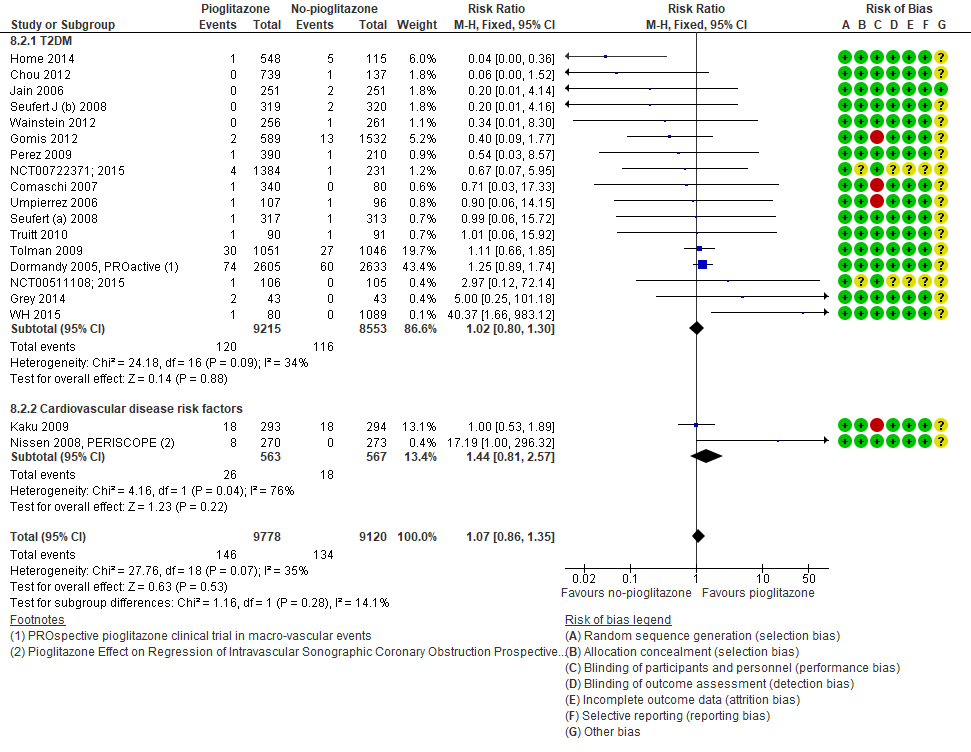


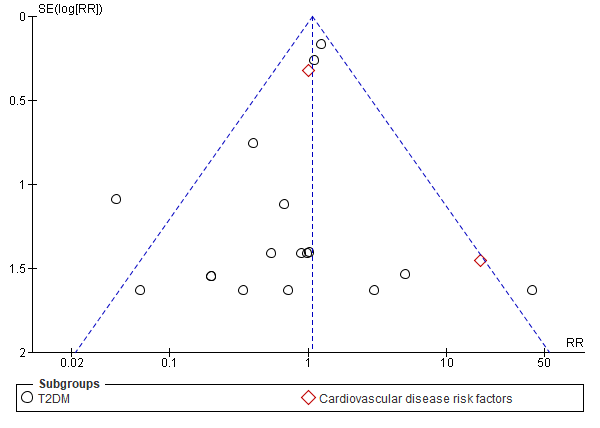


Figure S12. Forest and funnel plot of pioglitazone and fracture in patients with T2DM, Fixed-effect model.

The use of pioglitazone did not increase the occurrence of fracture in T2DM patients at a risk or not at a risk of CVD (RR, 0.90; 95% CI, 0.57–1.42; P = 0.65) versus non-pioglitazone using a fixed-effect model (Figure S13).


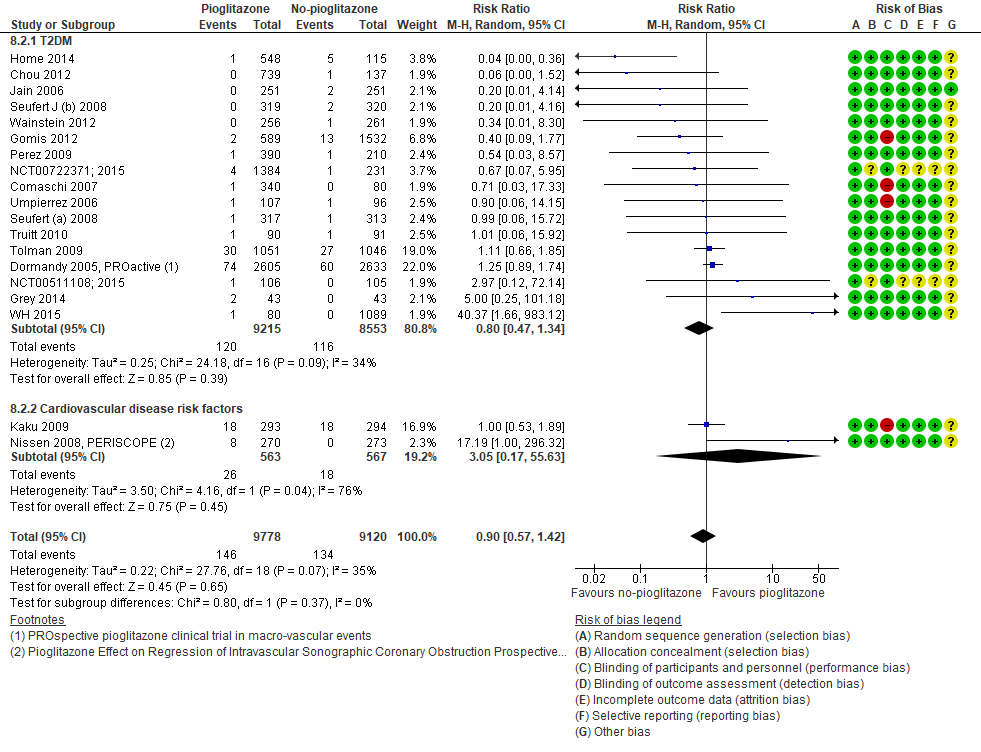


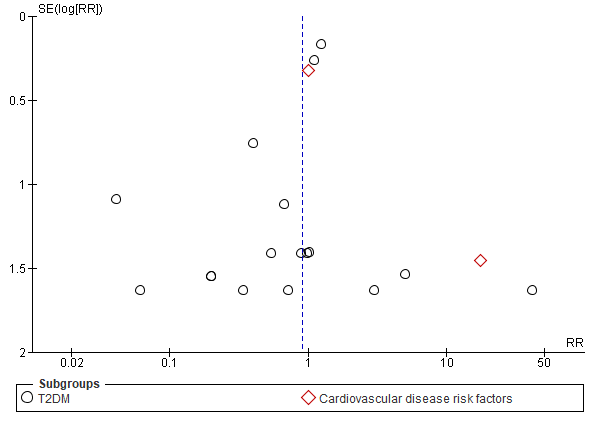


Figure S13. Forest and funnel plot of pioglitazone and fracture in patients with T2DM, Random-effect model.

Table S18. GRADE evidence profile of pioglitazone and fracture by baseline characteristics

| **Certainty assessment** | | | | | | | **Summary of findings** | | | | |
| --- | --- | --- | --- | --- | --- | --- | --- | --- | --- | --- | --- |
| **№ of participants (trials)** | **Risk of bias** | **Inconsistency** | **Indirectness** | **Imprecision** | **Publication bias** | **Overall certainty of evidence** | **Study event rates (%)** | | **Relative effect (95% CI)** | **Anticipated absolute effects** | |
|  |  |  |  |  |  |  | **With placebo / controls** | **With Pioglitazone** |  | **Risk with placebo / controls** | **Risk difference with pioglitazone** |
| **Nondiabetic (follow-up: range 24 to 251 weeks)** | | | | | | | | | | | |
| 5843 (8 RCTs) | serious^a^ | not serious | not serious | very serious^a^ | none | ⨁◯◯◯  VERY LOW | 115/2894 (4.0%) | 153/2949 (5.2%) | RR 1.32 (1.05 to 1.67) | 4 per 100 | 1 more per 100 (from 0 fewer to 3 more) |
| **Insulin resistance (follow-up: range 78 to 157 weeks)** | | | | | | | | | | | |
| 1165 (3 RCTs) | not serious | not serious | not serious | very serious^c^ | none | ⨁⨁◯◯ LOW | 16/580 (2.8%) | 14/585 (2.4%) | RR 0.87 (0.43 to 1.76) | 3 per 100 | 0 fewer per 100 (from 2 fewer to 2 more) |
| **Stroke/TIA (follow-up: median 251 weeks)** | | | | | | | | | | | |
| 3876 (1 RCT) | not serious | not serious | not serious | very serious^d^ | none | ⨁⨁◯◯ LOW | 94/1937 (4.9%) | 133/1939 (6.9%) | RR 1.41 (1.09 to 1.83) | 5 per 100 | 2 more per 100 (from 0 fewer to 4 more) |
| **Liver disease (follow-up: range 88 to 96 weeks)** | | | | | | | | | | | |
| 313 (2 RCTs) | serious^a^ | not serious | not serious | very serious^e^ | none | ⨁◯◯◯  VERY LOW | 5/156 (3.2%) | 4/157 (2.5%) | RR 0.83 (0.24 to 2.84) | 3 per 100 | 1 fewer per 100 (from 2 fewer to 6 more) |
| **Rheumatoid arthritis (follow-up: range 24 to 251 weeks)** | | | | | | | | | | | |
| 256 (1 RCT) | not serious | not serious | not serious | very serious^f^ | none | ⨁⨁◯◯ LOW | 0/127 (0.0%) | 1/129 (0.8%) | RR 2.95 (0.12 to 71.84) | 0 per 100 | 0 fewer per 100 (from 0 fewer to 0 fewer) |
| **Parkinson`s disease (follow-up: mean 44 weeks)** | | | | | | | | | | | |
| 233 (1 RCT) | not serious | not serious | not serious | very serious^g^ | none | ⨁⨁◯◯ LOW | 0/94 (0.0%) | 1/139 (0.7%) | RR 2.04 (0.08 to 49.44) | 0 per 100 | 0 fewer per 100 (from 0 fewer to 0 fewer) |
| **Diabetic (follow-up: range 24 to 192 weeks)** | | | | | | | | | | | |
| 18898 (19 RCTs) | serious^h^ | not serious | not serious | very serious^i^ | none | ⨁◯◯◯  VERY LOW | 134/9120 (1.5%) | 146/9778 (1.5%) | RR 1.07 (0.86 to 1.35) | 1 per 100 | 0 fewer per 100 (from 0 fewer to 1 more) |
| **T2DM (follow-up: range 24 to 156 weeks)** | | | | | | | | | | | |
| 17768 (17 RCTs) | serious^h^ | not serious | not serious | very serious^j^ | none | ⨁◯◯◯  VERY LOW | 116/8553 (1.4%) | 120/9215 (1.3%) | RR 1.02 (0.80 to 1.30) | 1 per 100 | 0 fewer per 100 (from 0 fewer to 0 fewer) |
| **T2DM with cardiovascular disease risk factors (follow-up: range 78 to 192 weeks)** | | | | | | | | | | | |
| 1130 (2 RCTs) | serious^h^ | not serious | not serious | very serious^k^ | none | ⨁◯◯◯  VERY LOW | 18/567 (3.2%) | 26/563 (4.6%) | RR 1.44 (0.81 to 2.57) | 3 per 100 | 1 more per 100 (from 1 fewer to 5 more) |

***Abbreviations:*** **CI:** confidence interval; **RCTs:** randomized controlled trials; **RR:** Risk ratio; **T2DM:** type two diabetes mellitus.

***Note:*** The GRADE scores were from the fixed-effect model.

#### GRADE evidence

**a.** Five trial with a small weight (0.4%) in insulin-resistant individuals and (5.2%, 0.8%, and 0.6%) in patients with T2DM (13.1%) at a risk of CVD with the overall EE rated as a high risk of bias due to a lack of blinding (open-label design) out of eight trials.

**b.** The overall imprecision was precise and narrow CIs with a significant effect size difference (P = 0.02). The 95% CI was not consistent with the possibility for a substantial negative effect exceeding the MID, including only 268 fracture events with a large sample size.

**c.** The overall imprecision was precise with no significant effect size difference (P = 0.69). However, all trials reported overlapping and narrow CIs. The 95% CI was not consistent with the possibility for a substantial negative effect exceeding the MID, including only 30 fracture events with a small sample size (1,165).

**d.** The overall imprecision and narrow CIs was precise with a significant effect size difference (P = 0.008). All trials reported non-overlapping and narrow CIs. The 95% CI was not consistent with the possibility for a substantial negative effect exceeding the MID, including only 227 fracture events with a large sample size.

**e.** The overall imprecision was precise with no significant effect size difference (P = 0.77). However, all trials reported overlapping CIs, in which one trial reported a wide CI. The 95% CI was not consistent with the possibility for a substantial negative effect exceeding the MID, including only nine fracture events with a small sample size (313).

**f.** The overall imprecision was precise with no significant effect size difference (P = 0.51). However, one trial reported overlapping and wide CIs. The 95% CI was not consistent with the possibility for a substantial negative effect exceeding the MID, including only one fracture event with a small sample size (256).

**g.** The overall imprecision was precise with no significant effect size difference (P = 0.66). However, one trial reported overlapping and wide CIs. The 95% CI was not consistent with the possibility for a substantial negative effect exceeding the MID, including only one fracture event with a small sample size (233).

**h.** Four trails with large (13.1%) and small weight (5.2%, 0.8%, and 0.6%) with the overall EE rated as a high risk of bias due to a lack of blinding (open-label design) out of 19 trials.

**i.** The overall imprecision was precise and narrow CIs with no significant effect size difference (P = 0.53). The 95% CI was not consistent with the possibility for a substantial negative effect exceeding the MID, including only 280 fracture events with a large sample size.

**j.** The overall imprecision was precise with no significant effect size difference (P = 0.88). However, all trials reported overlapping CIs, in which seven trials reported wide CIs. The 95% CI was not consistent with the possibility for a substantial negative effect exceeding the MID, including only 236 fracture events with a large sample size.

**k.** The overall imprecision was precise with no significant effect size difference (P = 0.22). However, all trials reported overlapping CIs, in which one trail reported a wide CI. The 95% CI was not consistent with the possibility for a substantial negative effect exceeding the MID, including only 44 fracture events with a small sample size (1,130).

In summary, the overall certainty of the pooled EE had a low imprecision with a significant effect size difference in the level of evidence (P = 0.02) in nondiabetic individuals, but with no significant effect size difference in the level of evidence in patients with diabetes (P = 0.53). However, the majority of trials reported overlapping CIs, in which 11 trials reported wide CIs. Three trials did not cross the line of no difference (1), in which a large trial (80.6% in nondiabetic individuals) and small trial (0.1%, in patients with T2DM) reports a significant association of fracture in the direction of pioglitazone favor across non-pioglitazone, and small trial (6.0%) in the direction of non-pioglitazone favor across pioglitazone. The 95% CI was consistent with the possibility for a substantial negative effect exceeding the MID, including 548 fracture events with a large sample size. There was no evidence of inconsistency, significant heterogeneity (P = 0.72 and I^2^ = 0% vs. nondiabetic; and P = 0.07 and I^2^ = 35% vs. patients with T2DM), or subgroup difference (P = 0.65 and I^2^ = 0% vs. nondiabetic; P = 0.28 and I^2^ = 14.1% vs. patients with T2DM). The overall magnitude of reporting bias did not affect our results, as these trials were double-blind RCTs. However, the magnitude of effect of some trials that suffered from limitations likely resulted in a biased assessment of the intervention effect due to performance and selection biases (five trials with an open-label design out of 27). The indirectness of the trials indicated a high level of evidence, with an individual trial PICOS element aligning close to the review PICOS. Finally, there was no evidence of reporting bias. However, two trials in patients with T2DM reported an outlier outside the pyramid edge due to multiple interventional groups resulting in wide CIs.

### Pioglitazone and fracture by skeletal location and comparators

Most of the fracture outcomes studied according to bone involvement appeared to be non-serious events. Of the 29 trials that studied fracture outcomes by anatomical site, no difference in fracture rates was observed across the groups in 26 trials but fracture rates significantly increased in three trials: one trial on TZD rosiglitazone,^41^ involving the spine, upper limbs and lower limbs, and two trials on the TZD pioglitazone, involving the upper limbs and lower limbs^79^ and the spine, upper limbs, and lower limbs.^1^

Pioglitazone use increases the number of fractures in the spine (RR, 2.13; 95% CI, 1.28–3.55; P = 0.004) and the lower extremities (RR, 1.85; 95% CI, 1.33–2.56; P = 0.0002) versus placebo. However, fracture did not increase the number of fractures in the hip (RR, 1.38; 95% CI, 0.84–2.28; P = 0.20), femur (RR, 8.99; 95% CI, 0.48–166.88; P = 0.14), upper-extremities (RR, 1.37; 95% CI, 0.98–1.93; P = 0.07), wrist (RR, 6.91; 95% CI, 0.36–133.16; P = 0.20), or in the ankle (RR, 0.63; 95% CI, 0.21–1.90; P = 0.41) versus placebo or controls, using a fixed-effect model (Figure S14). However, only the lower-limb (RR, 1.84; 95% CI, 1.33–2.55; P = 0.0002) fracture in patients taking pioglitazone versus placebo were increased in the random-effect model (Figure S15). The GRADE scores were moderate (Table S19).


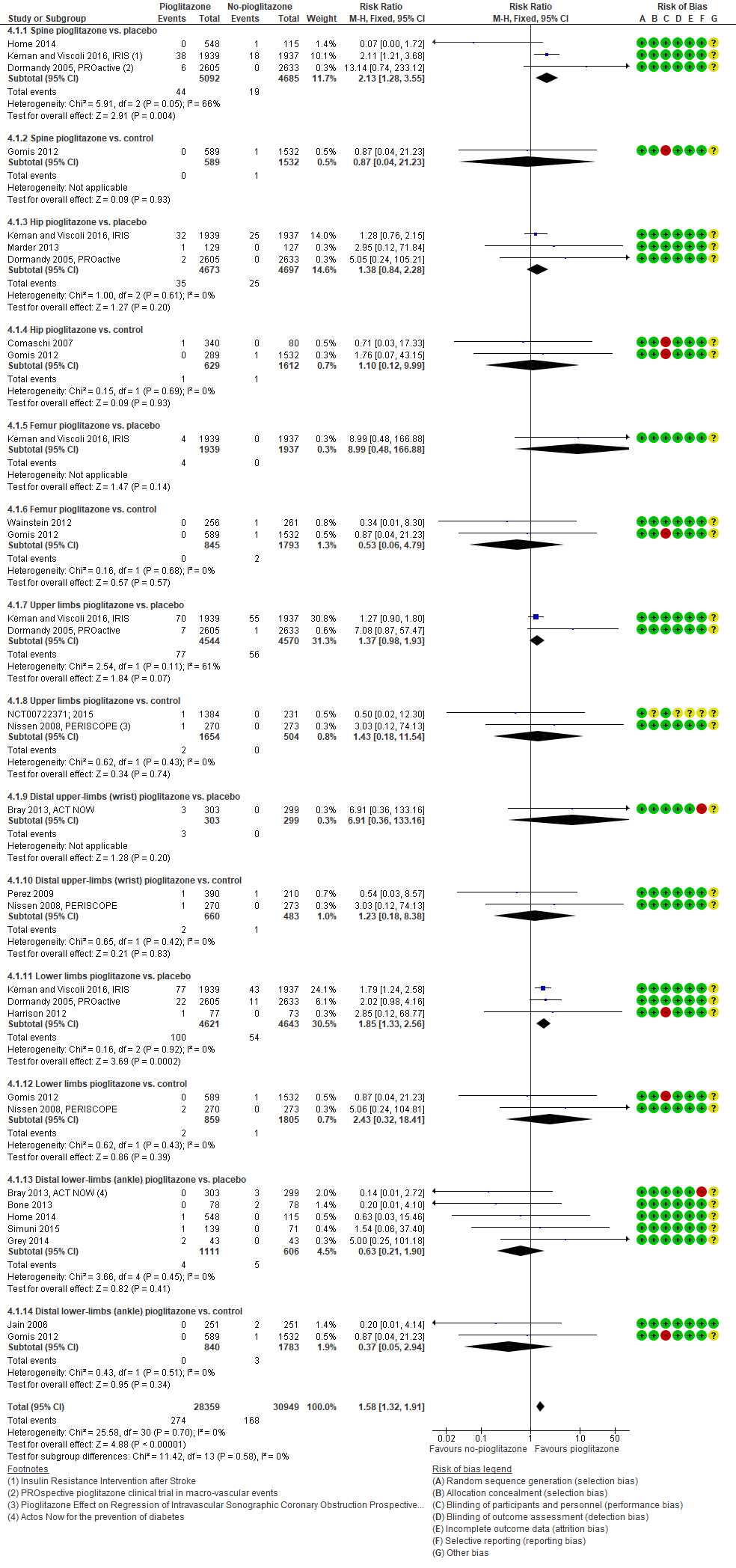


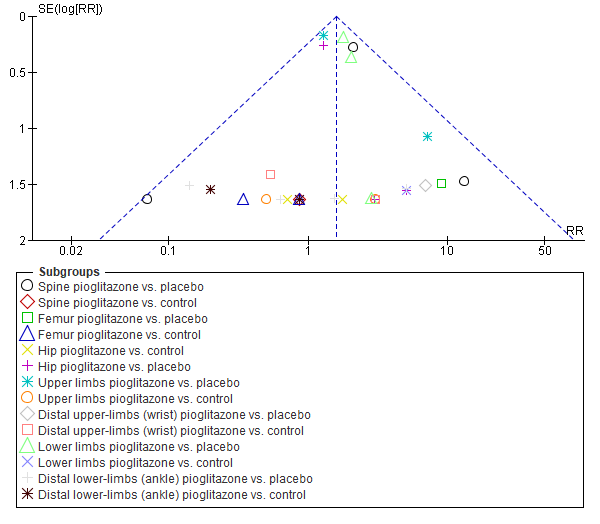


Figure S14. Forest and funnel plot of pioglitazone and fracture by skeletal location and comparators, Fixed-effect model.


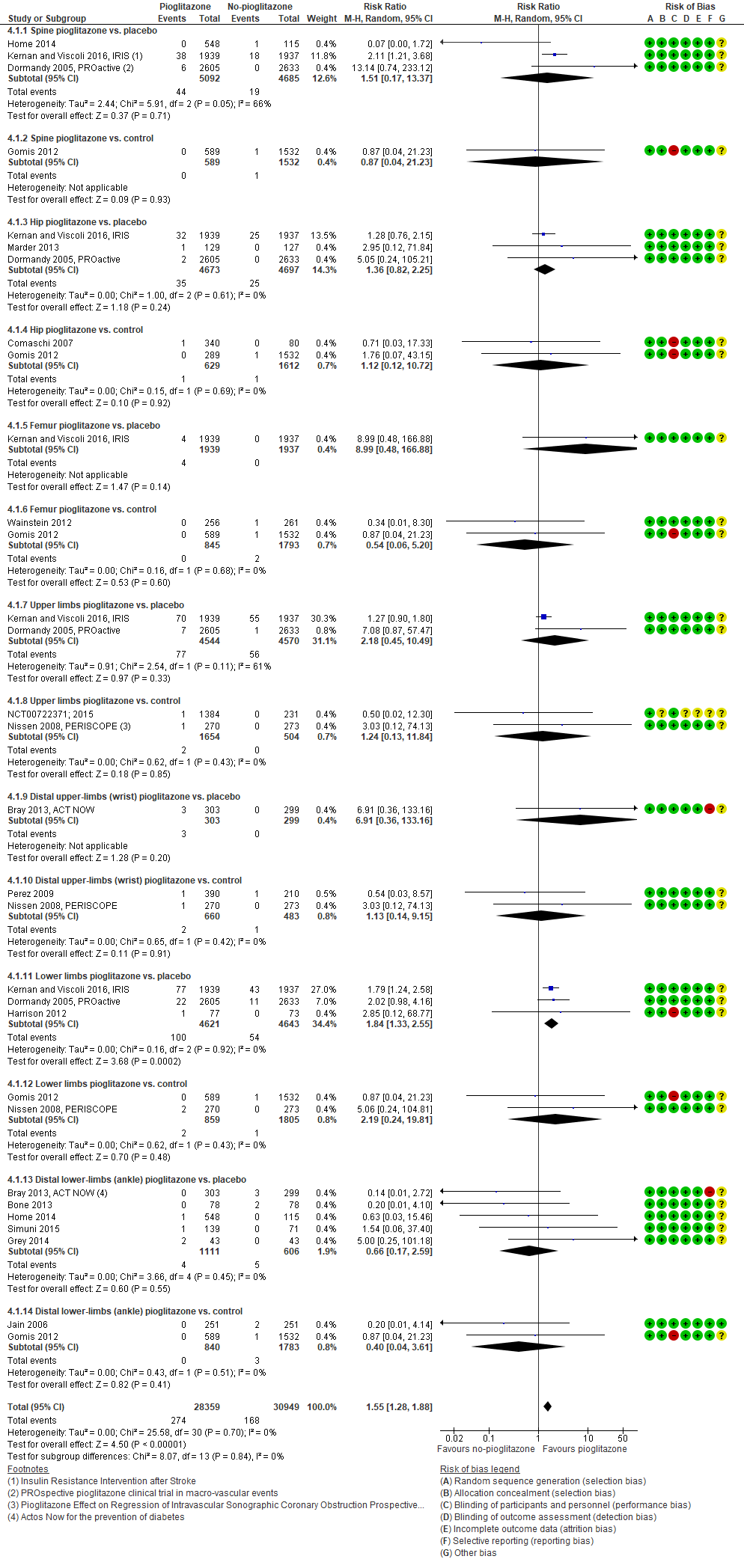


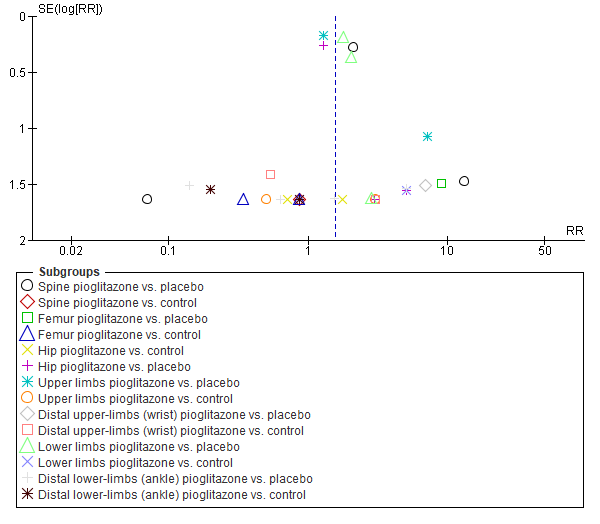


Figure S15. Forest and funnel plot of pioglitazone and fracture by skeletal location and comparators, Random-effect model.

Table S19. GRADE evidence profile of pioglitazone fracture by skeletal location and comparators

| **Certainty assessment** | | | | | | | **Summary of findings** | | | | |
| --- | --- | --- | --- | --- | --- | --- | --- | --- | --- | --- | --- |
| **№ of participants (trials)** | **Risk of bias** | **Inconsistency** | **Indirectness** | **Imprecision** | **Publication bias** | **Overall certainty of evidence** | **Study event rates (%)** | | **Relative effect (95% CI)** | **Anticipated absolute effects** | |
|  |  |  |  |  |  |  | **With placebo / controls** | **With Pioglitazone** |  | **Risk with placebo / controls** | **Risk difference with pioglitazone** |
| **Pioglitazone and fracture by skeletal location and comparators ((follow-up: range 24 to 251 weeks)** | | | | | | | | | | | |
| 59308 (16 RCTs) | serious^a^ | not serious | not serious | not serious | none | ⨁⨁⨁◯ MODERATE | 168/30949 (0.5%) | 274/28359 (1.0%) | RR 1.58 (1.32 to 1.91) | 1 per 100 | 0 fewer per 100 (from 0 fewer to 0 fewer) |
| **Spine pioglitazone vs. placebo (follow-up: range 150 to 251 weeks)** | | | | | | | | | | | |
| 9777 (3 RCTs) | not serious | not serious | not serious | very serious^b^ | none | ⨁⨁◯◯ LOW | 19/4685 (0.4%) | 44/5092 (0.9%) | RR 2.13 (1.28 to 3.55) | 0 per 100 | 0 fewer per 100 (from 0 fewer to 1 more) |
| **Spine pioglitazone vs. controls (follow-up: mean 78 weeks)** | | | | | | | | | | | |
| 2121 (1 RCT) | serious^a^ | not serious | not serious | very serious^c^ | none | ⨁◯◯◯  VERY LOW | 1/1532 (0.1%) | 0/589 (0.0%) | RR 0.87 (0.04 to 21.23) | 0 per 100 | 0 fewer per 100 (from 0 fewer to 1 more) |
| **Hip pioglitazone vs. placebo (follow-up: range 24 to 78 weeks)** | | | | | | | | | | | |
| 9370 (3 RCTs) | not serious | not serious | not serious | very serious^d^ | none | ⨁⨁◯◯ LOW | 25/4697 (0.5%) | 35/4673 (0.7%) | RR 1.38 (0.84 to 2.28) | 1 per 100 | 0 fewer per 100 (from 0 fewer to 1 more) |
| **Hip pioglitazone vs. controls (follow-up: range 32 to 251 weeks)** | | | | | | | | | | | |
| 2241 (2 RCTs) | serious^a^ | not serious | not serious | very serious^e^ | none | ⨁◯◯◯  VERY LOW | 1/1612 (0.1%) | 1/629 (0.2%) | RR 1.10 (0.12 to 9.99) | 0 per 100 | 0 fewer per 100 (from 0 fewer to 1 more) |
| **Femur pioglitazone vs. placebo (follow-up: median 251 weeks)** | | | | | | | | | | | |
| 3876 (1 RCT) | not serious | not serious | not serious | very serious^f^ | none | ⨁⨁◯◯ LOW | 0/1937 (0.0%) | 4/1939 (0.2%) | RR 8.99 (0.48 to 166.88) | 0 per 100 | 0 fewer per 100 (from 0 fewer to 0 fewer) |
| **Femur pioglitazone vs. controls (follow-up: range 32 to 78 weeks)** | | | | | | | | | | | |
| 2638 (2 RCTs) | serious^a^ | not serious | not serious | very serious^g^ | none | ⨁◯◯◯  VERY LOW | 2/1793 (0.1%) | 0/845 (0.0%) | RR 0.53 (0.06 to 4.79) | 0 per 100 | 0 fewer per 100 (from 0 fewer to 0 fewer) |
| **Upper-limbs pioglitazone vs. placebo (follow-up: range 150 to 251 weeks)** | | | | | | | | | | | |
| 9114 (2 RCTs) | not serious | not serious | not serious | very serious^h^ | none | ⨁⨁◯◯ LOW | 56/4570 (1.2%) | 77/4544 (1.7%) | RR 1.37 (0.98 to 1.93) | 1 per 100 | 0 fewer per 100 (from 0 fewer to 1 more) |
| **Upper-limbs pioglitazone vs. controls (follow-up: range 54 to 78 weeks)** | | | | | | | | | | | |
| 2158 (2 RCTs) | not serious | not serious | not serious | very serious^i^ | none | ⨁⨁◯◯ LOW | 0/504 (0.0%) | 2/1654 (0.1%) | RR 1.43 (0.18 to 11.54) | 0 per 100 | 0 fewer per 100 (from 0 fewer to 0 fewer) |
| **Distal upper-limbs (wrist) pioglitazone vs. placebo (follow-up: mean 126 weeks)** | | | | | | | | | | | |
| 602 (1 RCT) | not serious | not serious | not serious | very serious^j^ | none | ⨁⨁◯◯ LOW | 0/299 (0.0%) | 3/303 (1.0%) | RR 6.91 (0.36 to 133.16) | 0 per 100 | 0 fewer per 100 (from 0 fewer to 0 fewer) |
| **Distal upper-limbs (wrist) pioglitazone vs. controls (follow-up: range 24 to 78 weeks)** | | | | | | | | | | | |
| 1143 (2 RCTs) | not serious | not serious | not serious | very serious^k^ | none | ⨁⨁◯◯ LOW | 1/483 (0.2%) | 2/660 (0.3%) | RR 1.23 (0.18 to 8.38) | 0 per 100 | 0 fewer per 100 (from 0 fewer to 2 more) |
| **Lower limbs pioglitazone vs. placebo (follow-up: range 88 to 251 weeks)** | | | | | | | | | | | |
| 9264 (3 RCTs) | serious^a^ | not serious | not serious | very serious^l^ | none | ⨁◯◯◯  VERY LOW | 54/4643 (1.2%) | 100/4621 (2.2%) | RR 1.85 (1.33 to 2.56) | 1 per 100 | 1 more per 100 (from 0 fewer to 2 more) |
| **Lower limbs pioglitazone vs. controls (follow-up: mean 78 weeks)** | | | | | | | | | | | |
| 2664 (2 RCTs) | serious^a^ | not serious | not serious | very serious^m^ | none | ⨁◯◯◯  VERY LOW | 1/1805 (0.1%) | 2/859 (0.2%) | RR 2.43 (0.32 to 18.41) | 0 per 100 | 0 fewer per 100 (from 0 fewer to 1 more) |
| **Distal lower-limbs (ankle) pioglitazone vs. placebo (follow-up: range 44 to 156 weeks)** | | | | | | | | | | | |
| 1717 (5 RCTs) | not serious | not serious | not serious | very serious^n^ | none | ⨁⨁◯◯ LOW | 5/606 (0.8%) | 4/1111 (0.4%) | RR 0.63 (0.21 to 1.90) | 1 per 100 | 0 fewer per 100 (from 1 fewer to 1 more) |
| **Distal lower-limbs (ankle) pioglitazone vs. controls (follow-up: range 56 to 78 weeks)** | | | | | | | | | | | |
| 2623 (2 RCTs) | serious^a^ | not serious | not serious | very serious^o^ | none | ⨁◯◯◯  VERY LOW | 3/1783 (0.2%) | 0/840 (0.0%) | RR 0.37 (0.05 to 2.94) | 0 per 100 | 0 fewer per 100 (from 0 fewer to 0 fewer) |

***Abbreviations:*** **CI:** Confidence interval; **RCTs:** randomized controlled trials; **RR:** Risk ratio.

***Note:*** The GRADE scores were from the fixed-effect model.

#### GRADE evidence

**a.** Seven trials with a small weight (spine fracture [0.5%], hip fracture [0.5% and 0.3%], femur fracture [0.5%], lower-limb fracture [0.5% and 0.3%], and ankle fracture [0.5%]) with the overall EE rated as a high risk of bias due to a lack of blinding (open-label design) out of 16 trials.

**b.** The overall imprecision was precise with a significant effect size difference (P = 0.004). However, all trials reported overlapping CIs, in which one trial reported wide CIs. The 95% CI was not consistent with the possibility for a substantial negative effect exceeding the MID, including only 63 fracture events with a large sample size.

**c.** The overall imprecision was precise with no significant effect size difference (P = 0.93). However, one trial reported overlapping and wide CIs. The 95% CI was not consistent with the possibility for a substantial negative effect exceeding the MID, including only one fracture event with a large sample size.

**d.** The overall imprecision was precise with no significant effect size difference (P = 0.20). However, all trials reported overlapping CIs, in which two trials reported wide CIs. The 95% CI was not consistent with the possibility for a substantial negative effect exceeding the MID, including only 60 fracture events with a large sample size.

**e.** The overall imprecision was precise with no significant effect size difference (P = 0.93). However, all trials reported overlapping and wide CIs. The 95% CI was not consistent with the possibility for a substantial negative effect exceeding the MID, including only two fracture events with a large sample size.

**f.** The overall imprecision was precise with no significant effect size difference (P = 0.14). However, one trial reported overlapping and wide CIs. The 95% CI was not consistent with the possibility for a substantial negative effect exceeding the MID, including only four fracture events with a large sample size.

**g.** The overall imprecision was precise with no significant effect size difference (P = 0.57). However, all trials reported overlapping CIs, in which one trial reported wide CIs. The 95% CI was not consistent with the possibility for a substantial negative effect exceeding the MID, including only two fracture events with a large sample size.

**h.** The overall imprecision was precise with no significant effect size difference (P = 0.07). However, all trials reported overlapping CIs, in which one trial reported wide CIs. The 95% CI was not consistent with the possibility for a substantial negative effect exceeding the MID, including only 133 fracture events with a large sample size.

**i.** The overall imprecision was precise with no significant effect size difference (P = 0.74). However, all trials reported overlapping and wide CIs. The 95% CI was not consistent with the possibility for a substantial negative effect exceeding the MID, including only two fracture events with a large sample size.

**g.** The overall imprecision was precise with no significant effect size difference (P = 0.20). However, one trial reported overlapping and wide CIs. The 95% CI was not consistent with the possibility for a substantial negative effect exceeding the MID, including only three fracture events with a small sample size (602).

**k.** The overall imprecision was precise with no significant effect size difference (P = 0.83). However, all trials reported overlapping CIs, in which one trial reported wide CIs. The 95% CI was not consistent with the possibility for a substantial negative effect exceeding the MID, including only three fracture events with a small sample size (1,143).

**l.** The overall imprecision was precise with a significant effect size difference (P = 0.0002). However, all trials reported overlapping CIs, in which one trial reported wide CIs. The 95% CI was not consistent with the possibility for a substantial negative effect exceeding the MID, including only 154 fracture events with a large sample size.

**m.** The overall imprecision was precise with no significant effect size difference (P = 0.39). However, all trials reported overlapping and wide CIs. The 95% CI was not consistent with the possibility for a substantial negative effect exceeding the MID, including only three fracture events with a large sample size.

**n.** The overall imprecision was precise with no significant effect size difference (P = 0.41). However, all trials reported overlapping CIs, in which three trials reported wide CIs. The 95% CI was not consistent with the possibility for a substantial negative effect exceeding the MID, including only nine fracture events with a small sample size (1,717).

**o.** The overall imprecision was precise with no significant effect size difference (P = 0.34). However, all trials reported overlapping CIs, in which one trial reported wide CIs. The 95% CI was not consistent with the possibility for a substantial negative effect exceeding the MID, including only three fracture events with a large sample size.

In summary, the overall certainty of the pooled EE had a moderate imprecision with a significant effect size difference in the level of evidence (P < 0.00001). However, the majority of trials reported overlapping CIs, in which 11 trials reported wide CIs. Two trials (24.1% and 10.1%) did not cross the line of no difference (1), in which reports a significant association of spine and lower-limbs fracture in the direction of pioglitazone favor across placebo. The 95% CI was consistent with the possibility for a substantial negative effect exceeding the MID, including 442 skeletal fracture events with a large sample size. There was no evidence of inconsistency or significant heterogeneity (P = 0.70; I^2^ = 0%). However, in a subgroup analysis, spinal fracture in pioglitazone versus placebo groups, there was a moderate borderline nonsignificant evidence of heterogeneity across trials (I^2^ = 66%; P = 0.05). There was no evidence of a subgroup difference across trials (P = 0.58; I^2^ = 0%), using a fixed-effect model. However, the random-effect model predicted a significant difference across trials (T^2^ = 0.00%). As a such variation indicates that the actual difference in pioglitazone effect in each trial as well as sampling variability is due to chance, which could be explained by the differences in trial populations (such as age of patients), interventions received (such as dose of drug), follow-up length, or other factors including stroke-related disability as a recurrent fall. The majority of trials had a low risk of bias (96.4%). The overall magnitude of reporting bias did not affect our results, as these trials were double-blind RCTs. However, the magnitude of effect of some trials that suffered from limitations likely resulted in a biased assessment of the intervention effect due to performance and selection biases (three trials with an open-label design out of 16). The indirectness of the trials indicated a high level of evidence, with an individual trial PICOS element aligning close to the review PICOS. Finally, there was no evidence of reporting bias; all included trials were within the two boundaries of the pyramid.

### Pioglitazone and fracture by six difference and comparators

Among 16 trials that studied fracture outcomes by sex, the fracture rates were low and comparable between females and males in 10 of the trials. However, in six trials, the fracture rate increased with TZDs compared with that with placebo use; two trials considered the use of the TZD rosiglitazone in women^41^ and in men and women,^42^ and three trials used the TZD pioglitazone in post-menopausal women,^10^ in both sexes,^1,37^ and in men (in the latter two trials, the fracture rates increased with placebo compared with that with pioglitazone).^10,25^

The use of pioglitazone increased the incidence rate of fracture in females (RR, 1.56; 95% CI, 1.17–2.07; P = 0.002) versus placebo, but not with active comparators (RR, 1.55; 95% CI, 0.84–2.86; P = 0.16), using fixed-effect and random-effects models (Figures S16 and S17). However, the incidence rate of fracture did not increase in males (RR, 1.13; 95% CI, 0.84–1.52; P = 0.41) versus placebo or versus active comparators (RR, 0.95; 95% CI, 0.49–1.82; P = 0.87), using fixed-effect and random-effects models (Figures S18 and S19). The GRADE scores were high (Table S20).


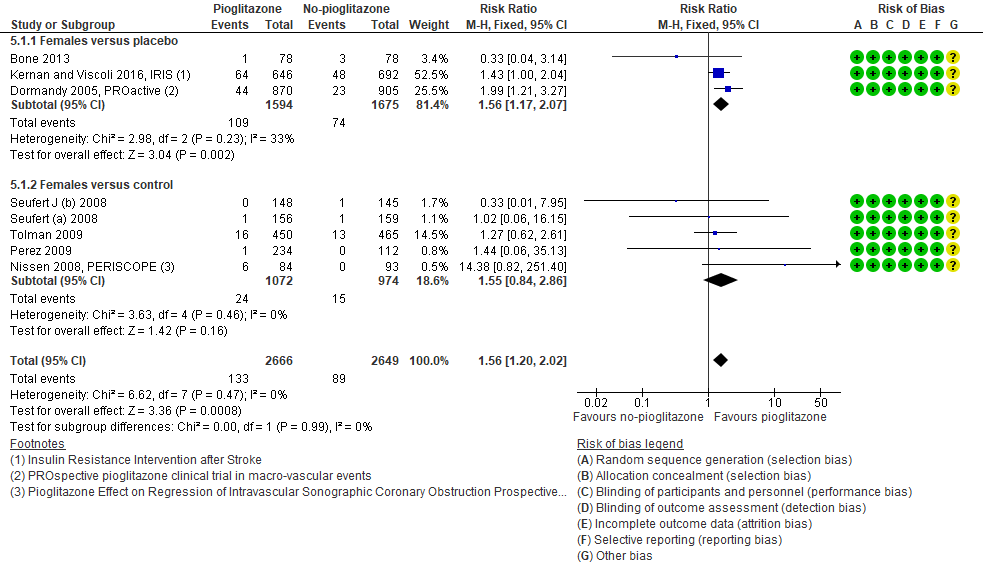


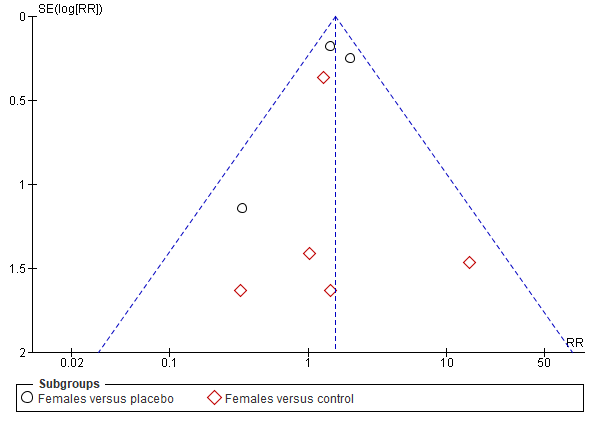


Figure S16. Forest and funnel plot of pioglitazone and fracture in females versus comparator, Fixed-effect model.


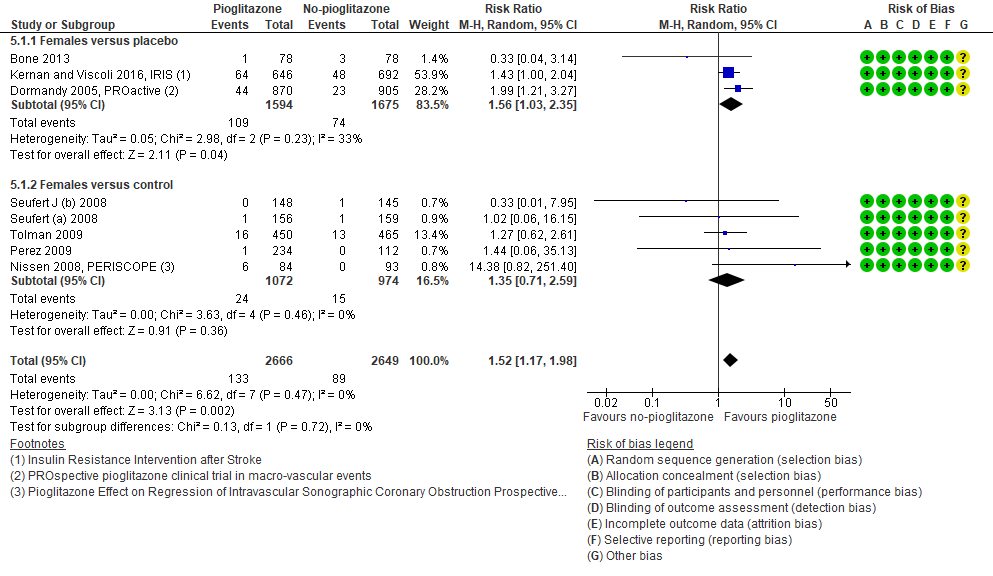


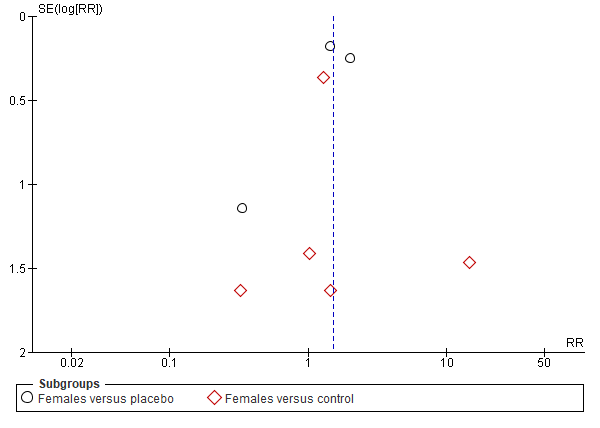


Figure S17. Forest and funnel plot of pioglitazone and fracture in females versus comparator, Random-effect model.


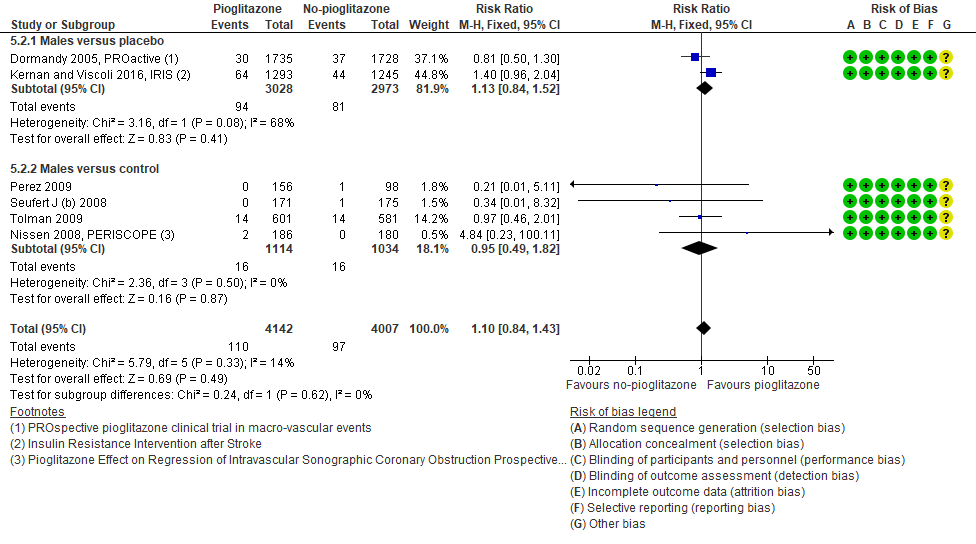


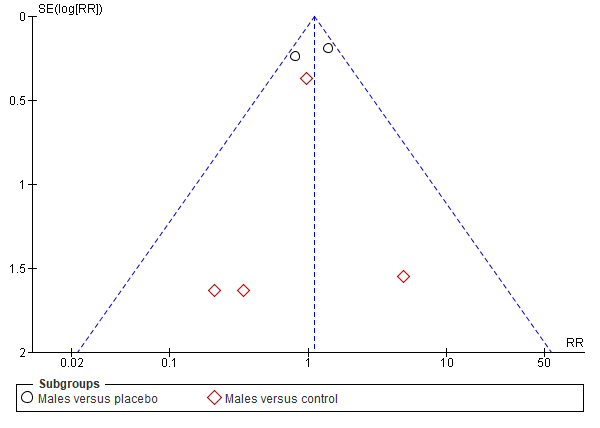


Figure S18. Forest and funnel plot of pioglitazone and fracture in males versus comparator, Fixed-effect model.


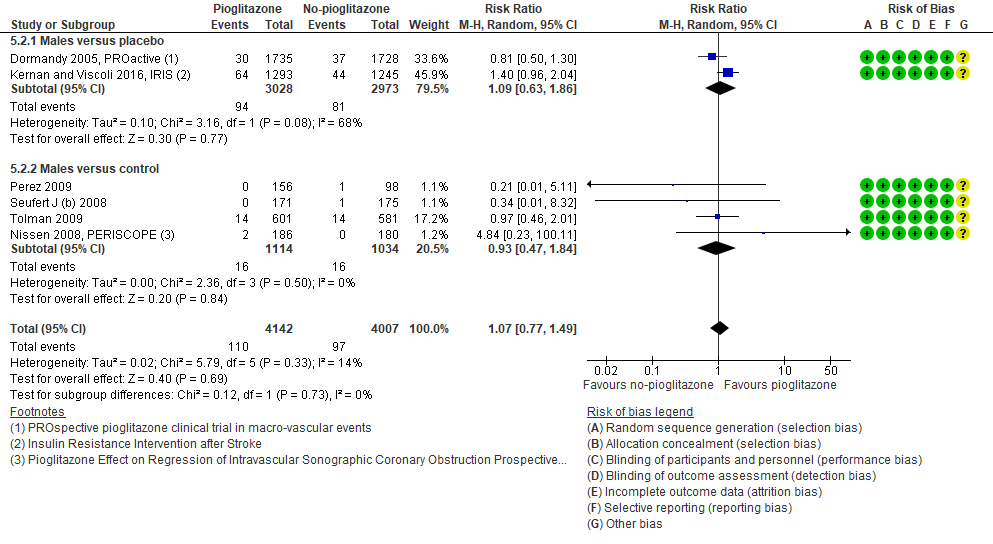


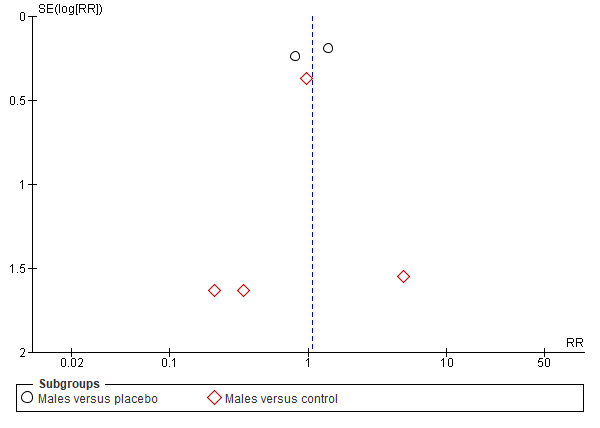


Figure S19. Forest and funnel plot of pioglitazone and fracture in males versus comparator, Random-effect model.

Table S20. GRADE evidence profile of pioglitazone and fracture by six difference and comparators

| **Certainty assessment** | | | | | | | **Summary of findings** | | | | |
| --- | --- | --- | --- | --- | --- | --- | --- | --- | --- | --- | --- |
| **№ of participants (trials)** | **Risk of bias** | **Inconsistency** | **Indirectness** | **Imprecision** | **Publication bias** | **Overall certainty of evidence** | **Study event rates (%)** | | **Relative effect (95% CI)** | **Anticipated absolute effects** | |
|  |  |  |  |  |  |  | **With placebo / controls** | **With Pioglitazone** |  | **Risk with placebo / controls** | **Risk difference with pioglitazone** |
| **Pioglitazone and fracture by six difference (follow-up: range 24 to 251 weeks)** | | | | | | | | | | | |
| 13464 (8 RCTs) | not serious | not serious | not serious | not serious | none | ⨁⨁⨁⨁ HIGH | 186/6656 (2.8%) | 243/6808 (3.6%) | RR 1.31 (1.09 to 1.58) | 3 per 100 | 1 more per 100 (0 fewer to 2 more) |
| **Females (follow-up: range 24 to 251 weeks)** | | | | | | | | | | | |
| 5315 (8 RCTs) | not serious | not serious | not serious | very serious^a^ | none | ⨁⨁◯◯ LOW | 89/2649 (3.4%) | 133/2666 (5.0%) | RR 1.56 (1.20 to 2.02) | 3 per 100 | 2 more per 100 (from 1 more to 3 more) |
| **Females – pioglitazone vs. placebo (follow-up: range 78 to 251 weeks)** | | | | | | | | | | | |
| 3269 (3 RCTs) | not serious | not serious | not serious | very serious^b^ | none | ⨁⨁◯◯ LOW | 74/1675 (4.4%) | 109/1594 (6.8%) | RR 1.56 (1.17 to 2.07) | 4 per 100 | 2 more per 100 (from 1 more to 5 more) |
| **Females – pioglitazone vs. controls (follow-up: range 24 to 144 weeks)** | | | | | | | | | | | |
| 2046 (5 RCTs) | not serious | not serious | not serious | very serious^c^ | none | ⨁⨁◯◯ LOW | 15/974 (1.5%) | 24/1072 (2.2%) | RR 1.55 (0.84 to 2.86) | 2 per 100 | 1 more per 100 (from 0 fewer to 3 more) |
| **Males (follow-up: range 24 to 251 weeks)** | | | | | | | | | | | |
| 8149 (6 RCTs) | not serious | not serious | not serious | very serious^d^ | none | ⨁⨁◯◯ LOW | 97/4007 (2.4%) | 110/4142 (2.7%) | RR 1.10 (0.84 to 1.43) | 2 per 100 | 0 fewer per 100 (from 0 fewer to 1 more) |
| **Males – pioglitazone vs. placebo (follow-up: range 150 to 251 weeks)** | | | | | | | | | | | |
| 6001 (2 RCTs) | not serious | not serious | not serious | very serious^e^ | none | ⨁⨁◯◯ LOW | 81/2973 (2.7%) | 94/3028 (3.1%) | RR 1.13 (0.84 to 1.52) | 3 per 100 | 0 fewer per 100 (from 0 fewer to 1 more) |
| **Males – pioglitazone vs. controls (follow-up: range 24 to 144 weeks)** | | | | | | | | | | | |
| 2148 (4 RCTs) | not serious | not serious | not serious | very serious^f^ | none | ⨁⨁◯◯ LOW | 16/1034 (1.5%) | 16/1114 (1.4%) | RR 0.95 (0.49 to 1.82) | 2 per 100 | 0 fewer per 100 (from 1 fewer to 1 more) |

***Abbreviations:*** **CI:** Confidence interval; **RCTs:** randomized controlled trials; **RR:** Risk ratio.

***Note:*** The GRADE scores were from the fixed-effect model.

#### GRADE evidence

**a.** The overall imprecision was precise and narrow CIs with a significant effect size difference (P = 0.0008). The 95% CI was not consistent with the possibility for a substantial negative effect exceeding the MID, including only 222 fracture events with a large sample size.

**b.** The overall imprecision was precise with a significant effect size difference (P = 0.002). However, all trials reported overlapping and narrow CIs. The 95% CI was not consistent with the possibility for a substantial negative effect exceeding the MID, including only 183 fracture events with a large sample size.

**c.** The overall imprecision was precise with no significant effect size difference (P = 0.16). However, all trials reported overlapping CIs, in which three trials reported wide CIs. The 95% CI was not consistent with the possibility for a substantial negative effect exceeding the MID, including only 39 fracture events with a large sample size.

**d.** The overall imprecision was precise and narrow CIs with no significant effect size difference (P = 0.49). The 95% CI was not consistent with the possibility for a substantial negative effect exceeding the MID, including only 207 fracture events with a large sample size.

**e.** The overall imprecision was precise with no significant effect size difference (P = 0.41). However, all trials reported overlapping and narrow CIs. The 95% CI was not consistent with the possibility for a substantial negative effect exceeding the MID, including only 175 fracture events with a large sample size.

**f.** The overall imprecision was precise with no significant effect size difference (P = 0.87). However, all trials reported overlapping CIs, in which one trial reported wide CIs. The 95% CI was not consistent with the possibility for a substantial negative effect exceeding the MID, including only 32 fracture events with a large sample size.

In summary, the overall certainty of the pooled EE had a high imprecision with a significant effect size difference in the level of evidence (P = 0.0008) in the trials that compared fracture risk with pioglitazone use versus placebo and active comparators in females, but not in males (P = 0.49). However, the majority of trials reported overlapping CIs, in which four trials reported wide CIs. The largest (52.5% and 25.5%) trials did not cross the line of no difference (1), in which reports a significant association of fracture in the direction of pioglitazone favor across placebo in females. However, the 95% CI was not consistent with the possibility for a substantial negative effect exceeding the MID, including 222 fracture events in females versus 207 fractures in males with a large sample size. There was no evidence of inconsistency, statistically significant heterogeneity (P = 0.47 and I^2^ = 0% in females vs. P = 0.33 and I^2^ = 14% in males), or subgroup difference (P = 0.99 and I^2^ = 0% in females vs. P = 0.62 and I^2^ = 0% in males). All trials had a low risk of bias (100%). The indirectness of the trials indicated a high level of evidence, with an individual trial PICOS element aligning close to the review PICOS. Finally, there was no evidence of reporting bias; all included trials were within the two boundaries of the pyramid.

### Pioglitazone and fracture by six, comparator, severity, and mechanism

The use of pioglitazone increased the incidence rate of non-serious fracture in females (RR, 1.57; 95% CI, 1.18–2.10; P = 0.002) versus placebo, but not with active comparators (RR, 1.55; 95% CI, 0.84–2.86; P = 0.16), using fixed-effect and random-effects models (Figure S20 and Figure S21). However, the use of pioglitazone increased the incidence rate of serious fracture in males (RR, 2.33; 95% CI, 1.46–3.72; P = 0.0004), specifically from low energy fractures (RR, 1.88; 95% CI, 1.33–2.66; P = 0.0003) versus placebo, fixed-effect and random-effects models (Figure S22 and Figure S23). The GRADE scores were high (Table S21).


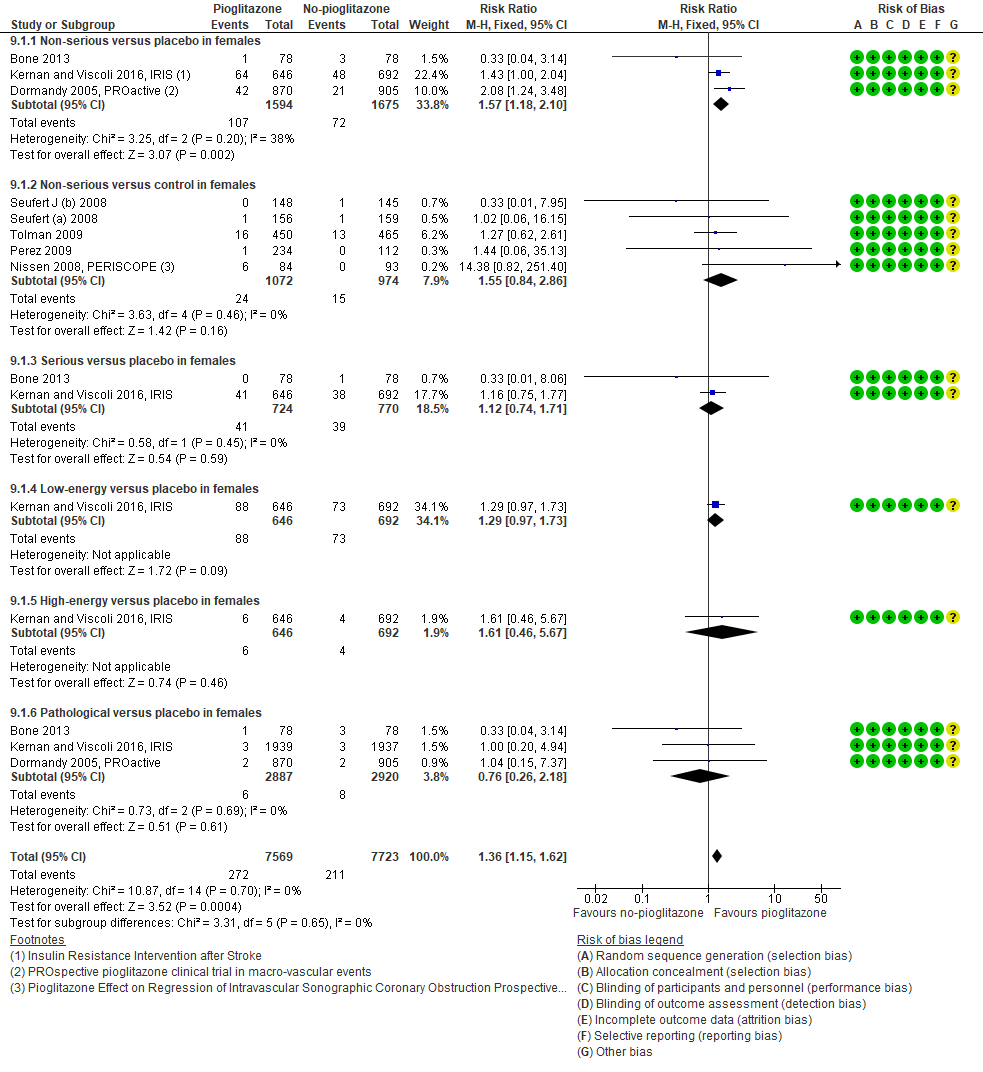


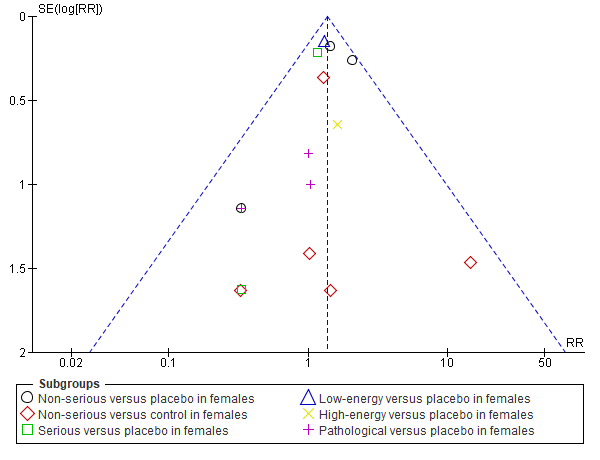


Figure S20. Forest and funnel plot of pioglitazone and fracture in females by comparator, severity, and mechanism, Fixed-effect model.


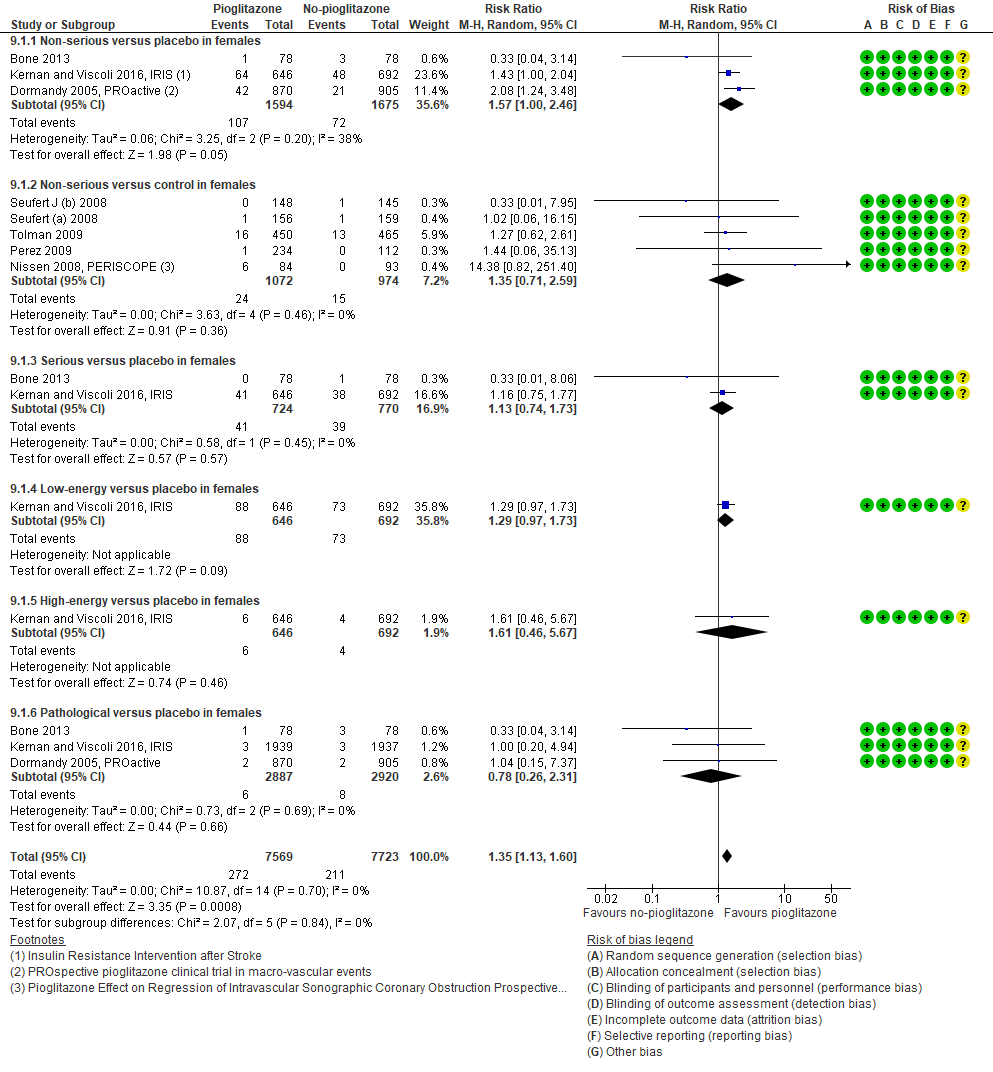


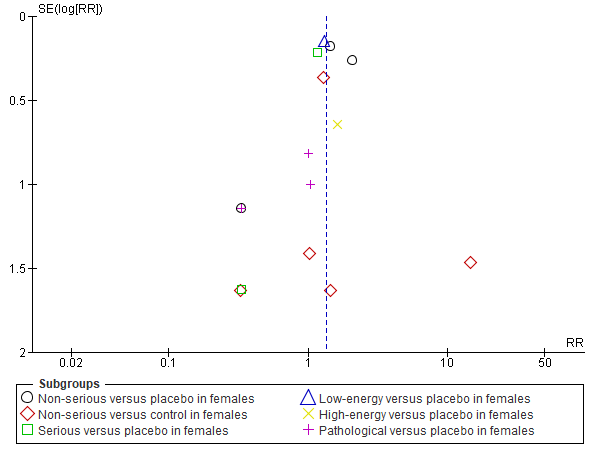


Figure S21. Forest and funnel plot of pioglitazone and fracture in females by comparator, severity, and mechanism, Random-effect model.


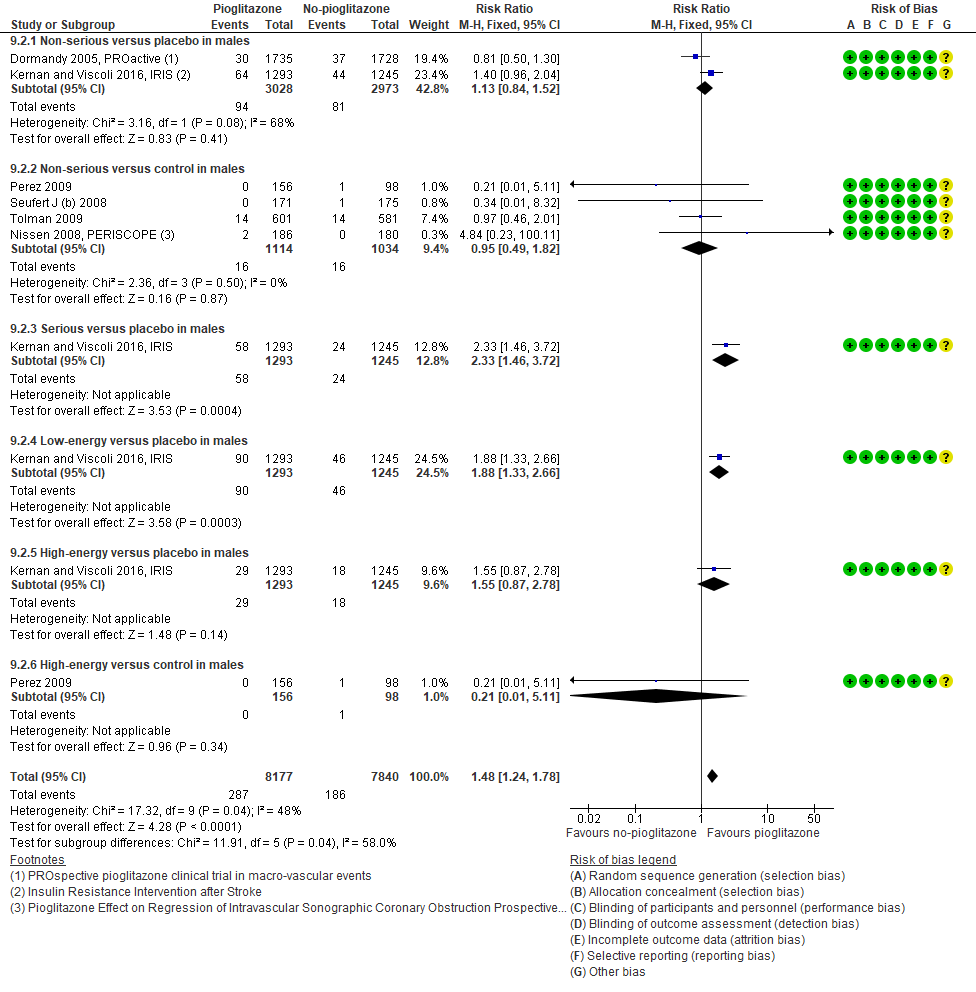


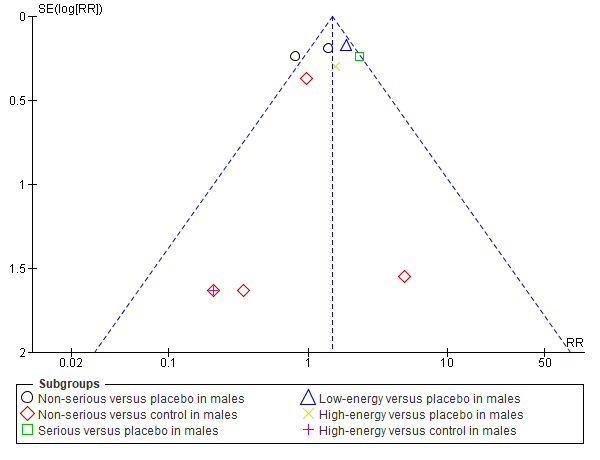


Figure S22. Forest and funnel plot of pioglitazone and fracture in males by comparator, severity, and mechanism, Fixed-effect model.


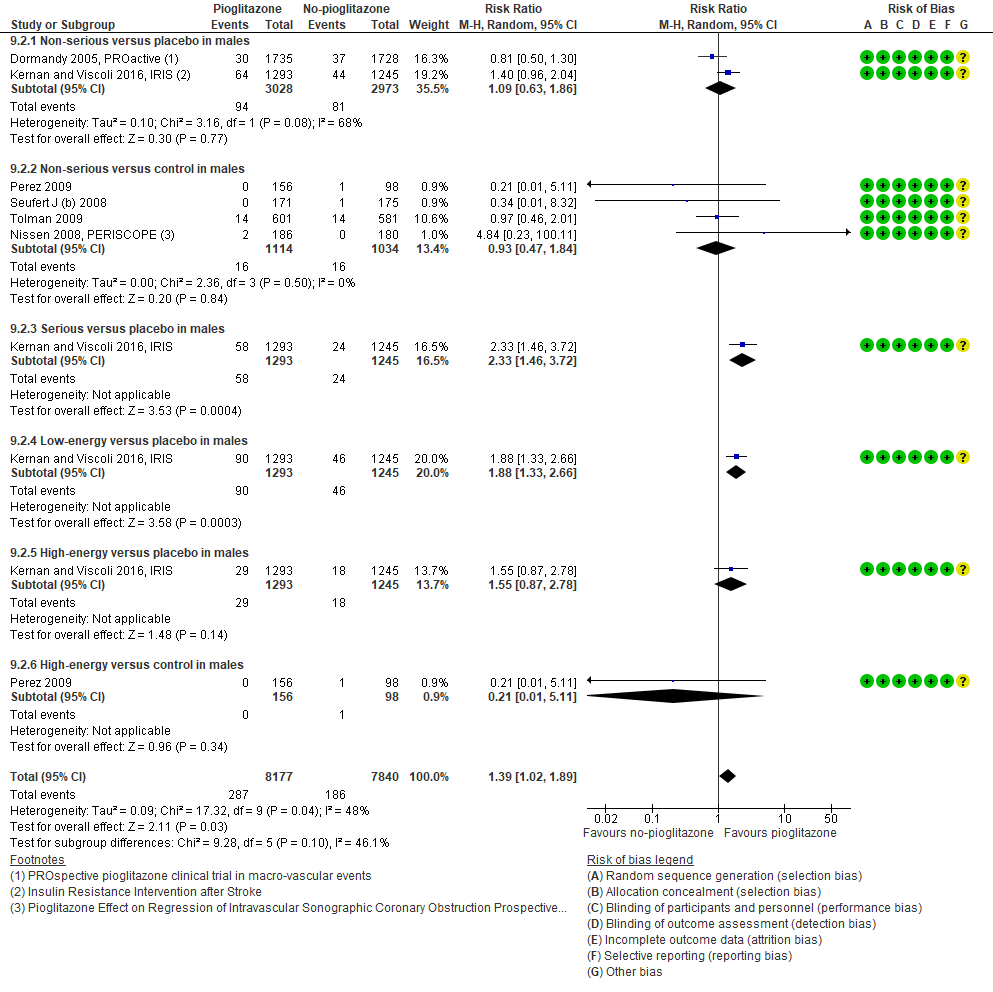


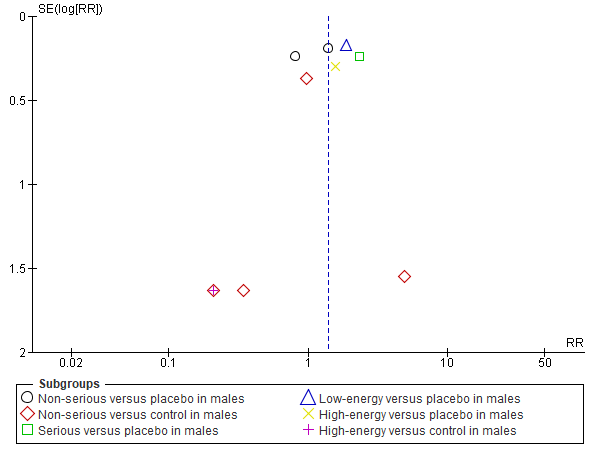


Figure S23. Forest and funnel plot of pioglitazone and fracture in males by comparator, severity, and mechanism, Random-effect model.

Table S21. GRADE evidence profile of pioglitazone and fracture by six, comparator, severity, and mechanism

| **Certainty assessment** | | | | | | | **Summary of findings** | | | | |
| --- | --- | --- | --- | --- | --- | --- | --- | --- | --- | --- | --- |
| **№ of participants (trials)** | **Risk of bias** | **Inconsistency** | **Indirectness** | **Imprecision** | **Publication bias** | **Overall certainty of evidence** | **Study event rates (%)** | | **Relative effect (95% CI)** | **Anticipated absolute effects** | |
|  |  |  |  |  |  |  | **With placebo / controls** | **With Pioglitazone** |  | **Risk with placebo / controls** | **Risk difference with pioglitazone** |
| **Pioglitazone and fracture by severity, mechanism, and comparators in females (follow-up: range 24 to 251 weeks)** | | | | | | | | | | | |
| 21099 (8 RCTs) | not serious | not serious | not serious | not serious | none | ⨁⨁⨁⨁ HIGH | 219/10643 (2.1%) | 278/10456 (2.7%) | RR 1.34 (1.13 to 1.59) | 2 per 100 | 1 more per 100 (from 0 fewer to 1 more) |
| **Non-serious fracture (pioglitazone vs. placebo) follow-up: range 87 to 251 weeks** | | | | | | | | | | | |
| 3269 (3 RCTs) | not serious | not serious | not serious | very serious^a^ | none | ⨁⨁◯◯ LOW | 72/1675 (4.3%) | 107/1594 (6.7%) | RR 1.57 (1.18 to 2.10) | 4 per 100 | 2 more per 100 (from 1 more to 5 more) |
| **Non-serious fracture (pioglitazone vs. controls) follow-up: range 24 to 144 weeks** | | | | | | | | | | | |
| 2046 (5 RCTs) | not serious | not serious | not serious | very serious^b^ | none | ⨁⨁◯◯ LOW | 15/974 (1.5%) | 24/1072 (2.2%) | RR 1.55 (0.84 to 2.86) | 2 per 100 | 1 more per 100 (from 0 fewer to 3 more) |
| **Serious fracture (pioglitazone vs. placebo) follow-up: range 78 to 251 weeks** | | | | | | | | | | | |
| 1494 (2 RCTs) | not serious | not serious | not serious | very serious^c^ | none | ⨁⨁◯◯ LOW | 39/770 (5.1%) | 41/724 (5.7%) | RR 1.12 (0.74 to 1.71) | 5 per 100 | 1 more per 100 (from 1 fewer to 4 more) |
| **Low energy fracture (pioglitazone vs. placebo) follow-up: median 251 weeks** | | | | | | | | | | | |
| 1338 (1 RCT) | not serious | not serious | not serious | very serious^d^ | none | ⨁⨁◯◯ LOW | 73/692 (10.5%) | 88/646 (13.6%) | RR 1.29 (0.97 to 1.73) | 11 per 100 | 3 more per 100 (from 0 fewer to 8 more) |
| **High energy fracture (pioglitazone vs. placebo) follow-up: median 251 weeks** | | | | | | | | | | | |
| 1338 (1 RCT) | not serious | not serious | not serious | very serious^e^ | none | ⨁⨁◯◯ LOW | 4/692 (0.6%) | 6/646 (0.9%) | RR 1.61 (0.46 to 5.67) | 1 per 100 | 0 fewer per 100 (from 0 fewer to 3 more) |
| **Pathological fracture (pioglitazone vs. placebo) follow-up: range 78 to 251 weeks** | | | | | | | | | | | |
| 5807 (3 RCTs) | not serious | not serious | not serious | very serious^f^ | none | ⨁⨁◯◯ LOW | 8/2920 (0.3%) | 6/2887 (0.2%) | RR 0.76 (0.26 to 2.18) | 0 per 100 | 0 fewer per 100 (from 0 fewer to 0 fewer) |
| **Pioglitazone fracture by severity, mechanism, and comparators in males (follow-up: range 24 to 251 weeks)** | | | | | | | | | | | |
| 16017 (6 RCTs) | not serious | not serious | not serious | not serious | none | ⨁⨁⨁⨁ HIGH | 186/7840 (2.4%) | 287/8177 (3.5%) | RR 1.48 (1.24 to 1.78) | 2 per 100 | 1 more per 100 (from 1 more to 2 more) |
| **Non-serious fracture (pioglitazone vs. placebo)** | | | | | | | | | | | |
| 6001 (2 RCTs) | not serious | not serious | not serious | very serious^g^ | none | ⨁⨁◯◯ LOW | 81/2973 (2.7%) | 94/3028 (3.1%) | RR 1.13 (0.84 to 1.52) | 3 per 100 | 0 fewer per 100 (from 0 fewer to 1 more) |
| **Non-serious fracture (pioglitazone vs. controls)** | | | | | | | | | | | |
| 2148 (4 RCTs) | not serious | not serious | not serious | very serious^h^ | none | ⨁⨁◯◯ LOW | 16/1034 (1.5%) | 16/1114 (1.4%) | RR 0.95 (0.49 to 1.82) | 2 per 100 | 0 fewer per 100 (from 1 fewer to 1 more) |
| **Serious fracture (pioglitazone vs. placebo)** | | | | | | | | | | | |
| 2538 (1 RCT) | not serious | not serious | not serious | very serious^i^ | none | ⨁⨁◯◯ LOW | 24/1245 (1.9%) | 58/1293 (4.5%) | RR 2.33 (1.46 to 3.72) | 2 per 100 | 3 more per 100 (from 1 more to 5 more) |
| **Low energy fracture (pioglitazone vs. placebo)** | | | | | | | | | | | |
| 2538 (1 RCT) | not serious | not serious | not serious | very serious^j^ | none | ⨁⨁◯◯ LOW | 46/1245 (3.7%) | 90/1293 (7.0%) | RR 1.88 (1.33 to 2.66) | 4 per 100 | 3 more per 100 (from 1 more to 6 more) |
| **High energy fracture (pioglitazone vs. placebo)** | | | | | | | | | | | |
| 2538 (1 RCT) | not serious | not serious | not serious | very serious^k^ | none | ⨁⨁◯◯ LOW | 18/1245 (1.4%) | 29/1293 (2.2%) | RR 1.55 (0.87 to 2.78) | 1 per 100 | 1 more per 100 (from 0 fewer to 3 more) |
| **High energy fracture (pioglitazone vs. controls)** | | | | | | | | | | | |
| 254 (1 RCT) | not serious | not serious | not serious | very serious^l^ | none | ⨁⨁◯◯ LOW | 1/98 (1.0%) | 0/156 (0.0%) | RR 0.21 (0.01 to 5.11) | 1 per 100 | 1 fewer per 100 (from 1 fewer to 4 more) |

***Abbreviations:*** **CI:** Confidence interval; **RCTs:** randomized controlled trials; **RR:** Risk ratio.

***Note:*** The GRADE scores were from the fixed-effect model.

#### GRADE evidence

**a.** The overall imprecision was precise with a significant effect size difference (P = 0.002). However, all trials reported overlapping and narrow CIs. The 95% CI was not consistent with the possibility for a substantial negative effect exceeding the MID, including only 179 fracture events with a large sample size.

**b.** The overall imprecision was precise with no significant effect size difference (P = 0.16). However, all trials reported overlapping CIs, in which three trials reported wide CIs. The 95% CI was not consistent with the possibility a substantial negative effect exceeding the MID, including only 39 fracture events with a large sample size.

**c.** The overall imprecision was precise with no significant effect size difference (P = 0.59). However, all trials reported overlapping and narrow CIs. The 95% CI was not consistent with the possibility for a substantial negative effect exceeding the MID, including only 80 fracture events with a small sample size (1,494).

**d.** The overall imprecision was precise with no significant effect size difference (P = 0.09). However, all trials reported overlapping and narrow CIs. The 95% CI was not consistent with the possibility for a substantial negative effect exceeding the MID, including only 161 fracture events with a small sample size (1,338).

**e.** The overall imprecision was precise with no significant effect size difference (P = 0.46). However, all trials reported overlapping and narrow CIs. The 95% CI was not consistent with the possibility for a substantial negative effect exceeding the MID, including only 10 fracture events with a small sample size (1,338).

**f.** The overall imprecision was precise with no significant effect difference (P = 0.61). However, all trials reported overlapping and narrow CIs. The 95% CI was not consistent with the possibility for a substantial negative effect exceeding the MID, including only 14 fracture events with a large sample size.

**g.** The overall imprecision was precise with no significant effect size difference (P = 0.41). However, all trials reported overlapping and narrow CIs. The 95% CI was not consistent with the possibility for a substantial negative effect exceeding the MID, including only 175 fracture events with a large sample size.

**h.** The overall imprecision was precise with no significant effect size difference (P = 0.87). However, all trials reported overlapping CIs, in which one trial reported wide CIs. The 95% CI was not consistent with the possibility for a substantial negative effect exceeding the MID, including only 32 fracture events with a large sample size.

**i.** The overall imprecision was precise with a significant effect size difference (P = 0.0004). However, all trials reported a non-overlapping and narrow CIs. The 95% CI was not consistent with the possibility for a substantial negative effect exceeding the MID, including only 82 fracture events with a large sample size.

**j.** The overall imprecision was precise with a significant effect size difference (P = 0.0003). However, all trials reported a non-overlapping and narrow CIs. The 95% CI was not consistent with the possibility for a substantial negative effect exceeding the MID, including only 106 fracture events with a large sample size.

**k.** The overall imprecision was precise with no significant effect size difference (P = 0.14). However, all trials reported overlapping and narrow CIs. The 95% CI was not consistent with the possibility for a substantial negative effect exceeding the MID, including only 47 fracture events with a large sample size.

**l.** The overall imprecision was precise with no significant effect size difference (P = 0.34). However, all trials reported overlapping and narrow CIs. The 95% CI was not consistent with the possibility for a substantial negative effect exceeding the MID, including only one fracture event with a small sample size (254).

In summary, the overall certainty of the pooled EE had a high imprecision with a significant effect size difference in the level of evidence (P = 0.0004) in trials that compared pioglitazone versus placebo and active comparators in females and males (P < 0.0001). However, the majority of trials reported overlapping CIs, in which four trials reported wide CIs. The largest trial in males (24.5%) and large (22.4%) and small (10.0%) trials in females did not cross the line of no difference (1), in which reports a significant association of fracture in the direction of pioglitazone favor across placebo in both sexes. However, the 95% CI was consistent with the possibility for a substantial negative effect exceeding the MID, including 483 fractures in females versus 473 fractures in males with a large sample size. There was no evidence of inconsistency, statistically significant heterogeneity (P = 0.70 and I^2^ = 0%), or subgroup difference (P = 0.65 and I^2^ = 0%) in females. However, in males, although the I^2^ for the statistical heterogeneity (48%) or subgroup difference (58%) were not scored high, there was evidence of significant inconsistency and subgroup difference across trials (P = 0.04), using a fixed-effect model. However, the random-effect model did not predict a significant difference across trials (T^2^ = 0.09%) without trial subgroup differences. As a such variation indicates that an actual difference in pioglitazone effect in each trial as well as sampling variability is due to chance, which could be explained by the differences in trial populations (such as age of patients), interventions received (such as dose of drug), follow-up length, or other factors including stroke-related disability as a recurrent fall (the IRIS trials dominator). All trials at had a low risk of bias (100%). The indirectness of the trials indicated a high level of evidence, with an individual trial PICOS element aligning close to the review PICOS. Finally, there was no evidence of reporting bias. However, one trial in males reports an outlier outside the pyramid edge due to multiple interventional groups resulting in wide CIs.

### Pioglitazone and fracture by bone mineral density and skeletal location

The data on bone mineral density (BMD) and the risk of fracture were minimal. Of the five trials that examined the effects of BMD on fracture risk, the fracture rates were low and comparable across groups. In two trials that evaluated the use of the TZD rosiglitazone in postmenopausal women, the BMD of the hip and femoral neck (FN)^32^ after 12 months of treatment and the BMD of the lumber spine (LS) and hip^33^ after 18 months of treatment decreased significantly. However, no significant effect was observed on BMD or bone remodeling markers in the LS, FN, or proximal femur in postmenopausal women after 12 to 18 months of pioglitazone use; in this trial, 1 pioglitazone-treated and 3 placebo-treated women experienced fracture after treatment.^5^ In another trial, significant bone loss was evident in the proximal femur (hip) without any significant changes in the LS, in the BMD levels, or in the biochemical markers of the bone after 12 months of pioglitazone use.^3^ However, the mean BMD was reduced in both sexes in the pelvis and in a higher males in the LS in patients using pioglitazone; no fracture was observed in these areas.^4^

In trials comparing pioglitazone with placebo, standardized mean difference (SMD) levels were decreased by pioglitazone and by the highest amount in the LS (SMD, -0.18; 95% CI, -0.34 to -0.03; P = 0.02) and hip (SMD, -0.53; 95% CI, -0.96 to -0.10; P = 0.02) but not in the FN (SMD, -0.25; 95% CI, -0.61 to 0.11; P = 0.17) using fixed-effect and random-effect (Figures S24 and S25) models. The GRADER scores were low (Table S22).


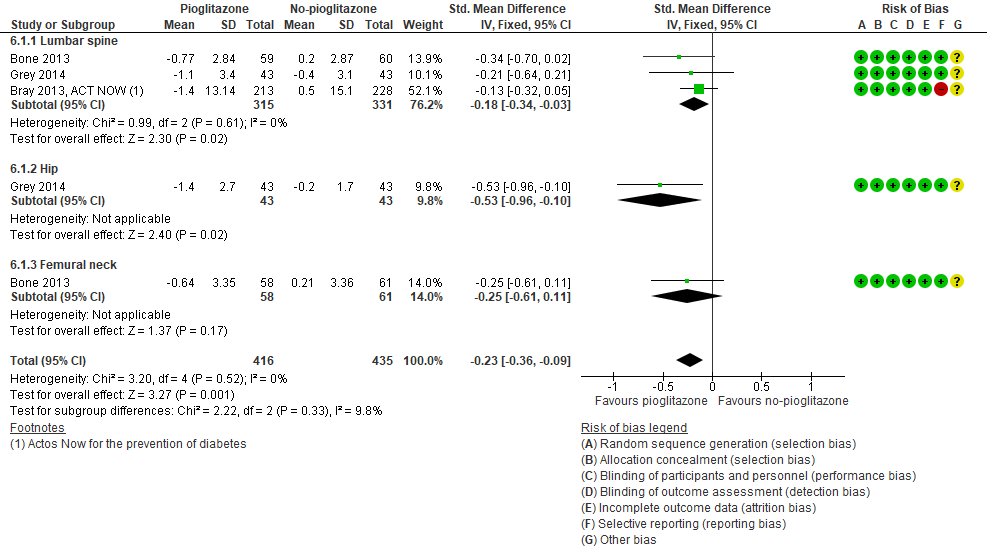


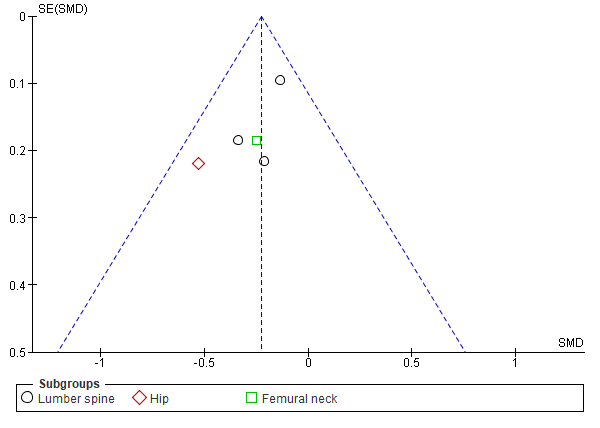


Figure S24. Forest and funnel plot of pioglitazone and fracture by bone mineral density versus comparator, Fixed-effect model.


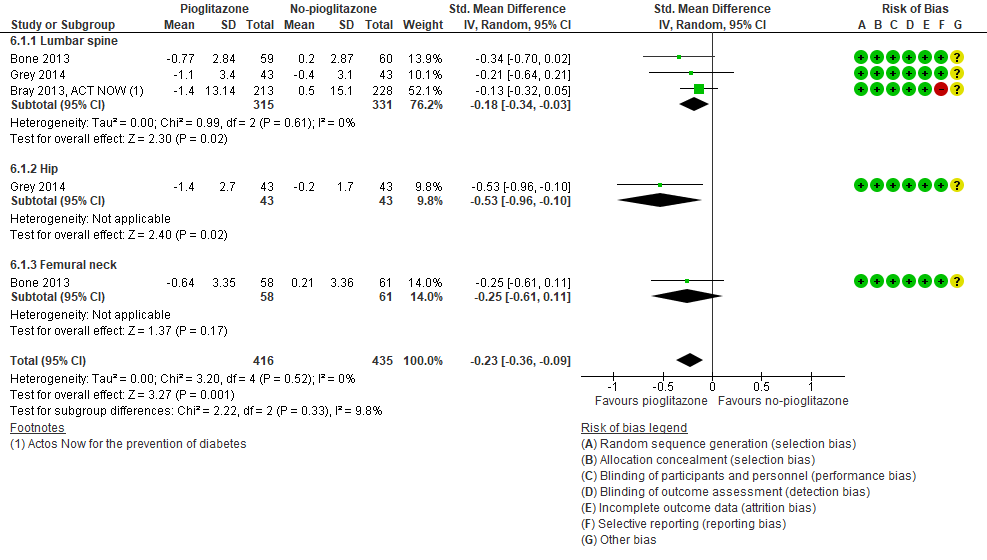


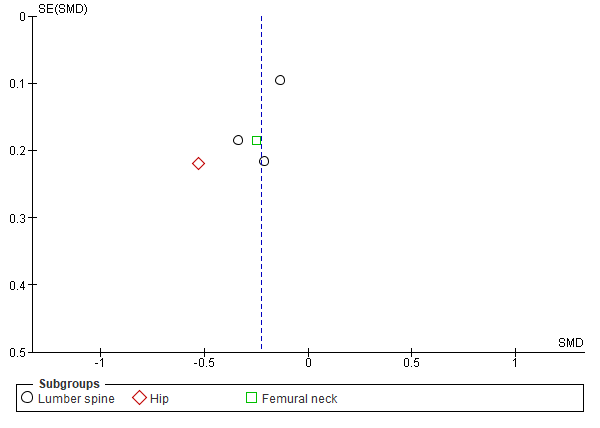


Figure S25. Forest and funnel plot of pioglitazone and fracture by bone mineral density versus comparator, Random-effect model.

Table S22. GRADE evidence profile of pioglitazone and fracture by bone mineral density and skeletal allocation

| **Certainty assessment** | | | | | | | **Summary of findings** | | | | |
| --- | --- | --- | --- | --- | --- | --- | --- | --- | --- | --- | --- |
| **№ of participants (trials)** | **Risk of bias** | **Inconsistency** | **Indirectness** | **Imprecision** | **Publication bias** | **Overall certainty of evidence** | **Study event rates (%)** | | **Relative effect (95% CI)** | **Anticipated absolute effects** | |
|  |  |  |  |  |  |  | **With placebo / controls** | **With Pioglitazone** |  | **Risk with placebo / controls** | **Risk difference with pioglitazone** |
| **Pioglitazone and bone mineral density by skeletal location (follow-up: range 52 to 126 weeks)** | | | | | | | | | | | |
| 851 (3 RCTs) | not serious | not serious | not serious | very serious^a^ | none | ⨁⨁◯◯ LOW | 435 | 416 | - | - | SMD 0.23 lower (0.36 lower to 0.09 lower) |
| **Lumber spine (follow-up: range 52 to 126 weeks)** | | | | | | | | | | | |
| 646 (3 RCTs) | not serious | not serious | not serious | very serious^b^ | none | ⨁⨁◯◯ LOW | 331 | 315 | - | - | SMD 0.18 lower (0.34 lower to 0.03 lower) |
| **Hip (follow-up means 52 weeks)** | | | | | | | | | | | |
| 86 (1 RCT) | not serious | not serious | not serious | very serious^f^ | none | ⨁⨁◯◯ LOW | 43 | 43 | - | - | SMD 0.53 lower (0.96 lower to 0.1 lower) |
| **Femoral neck (follow-up means 78 weeks)** | | | | | | | | | | | |
| 119 (1 RCT) | not serious | not serious | not serious | very serious^d^ | none | ⨁⨁◯◯ LOW | 61 | 58 | - | - | SMD 0.25 lower (0.61 lower to 0.11 higher) |

***Abbreviations:*** **CI:** Confidence interval; **RCTs:** randomized controlled trials; **RR:** Risk ratio.

***Note:*** The GRADE scores were from the fixed-effect model.

#### GRADE evidence

**a.** The overall imprecision was precise and narrow with a significant effect size difference (P = 0.001). The 95% CI was not consistent with the possibility for a substantial negative effect exceeding the MID, including only a few fracture events with a small sample size (851).

**b.** The overall imprecision was precise with a significant effect size difference (P = 0.02). However, all trials reported overlapping and narrow CIs. The 95% CI was not consistent with the possibility for a substantial negative effect exceeding the MID, including only a few fracture events with a small sample size (646).

**c.** The overall imprecision was precise with a significant effect difference (P = 0.02). However, only one trial with overlapping and narrow CIs reported a significant reduction in the BMD level at the hip. The 95% CI was not consistent with the possibility for a substantial negative effect exceeding the MID, including a few fracture events with a small sample size (86).

**d.** The overall imprecision was precise with no significant effect size difference (P = 0.17). However, included only one trial with overlapping and narrow CIs, in which reported a nonsignificant reduction in the BMD levels at the femoral neck. The 95% CI was not consistent with the possibility for a substantial negative effect exceeding the MID, including a few fracture events with a small sample size (119).

In summary, the overall certainty of EE had a low imprecision with a significant effect size difference in the level of evidence (P = 0.001). However, the majority of trials reported a small and overlapping CIs. A small trial (9.8%) did not cross the line of no difference (1) with a significant association of BMD loss in the direction of pioglitazone favor across non-pioglitazone. The 95% CI was not consistent with the possibility for a substantial negative effect exceeding the MID with a small sample size. There was no evidence of inconsistency, significant heterogeneity (P = 0.52 and I^2^ = 0%), or subgroup difference across trials (I^2^ = 9.8%; P = 0.33), using a fixed-effect model. However, the random-effect model predicted a significant difference across trials (T^2^ = 0.00%). As such a variation indicates that the actual difference in pioglitazone effect in each trial as well as sampling variability is due to chance, which could be explained by the differences in the trial populations (such as age of patients), interventions received (such as dose of drug), follow-up length, or other factors including stroke-related disability as a recurrent fall. All trials were at a low risk of bias (100%). The overall magnitude of reporting bias did not affect our results, as these trials were double-blind RCTs. The indirectness of the trials indicated a high level of evidence, with an individual trial PICOS element aligning close to the review PICOS. Finally, there was no evidence of reporting bias; all included trials were within the two boundaries of the pyramid.

### Pioglitazone and fracture by AHGs subclass

The use of pioglitazone subclasses did not increase fracture risk compared with the AHG subclasses metformin (RR, 0.32; 95% CI, 0.06–1.77; P = 0.19), sulfonylurea (RR, 1.27; 95% CI, 0.81–2.01; P = 0.30), or the fixed combinations of metformin and sulfonylurea (RR, 0.71; 95% CI, 0.03–17.33; P = 0.84) versus non-pioglitazone, using fixed-effect and random-effect (Figures S26 and S27) models. The GRADE scores were very low (Table S23).


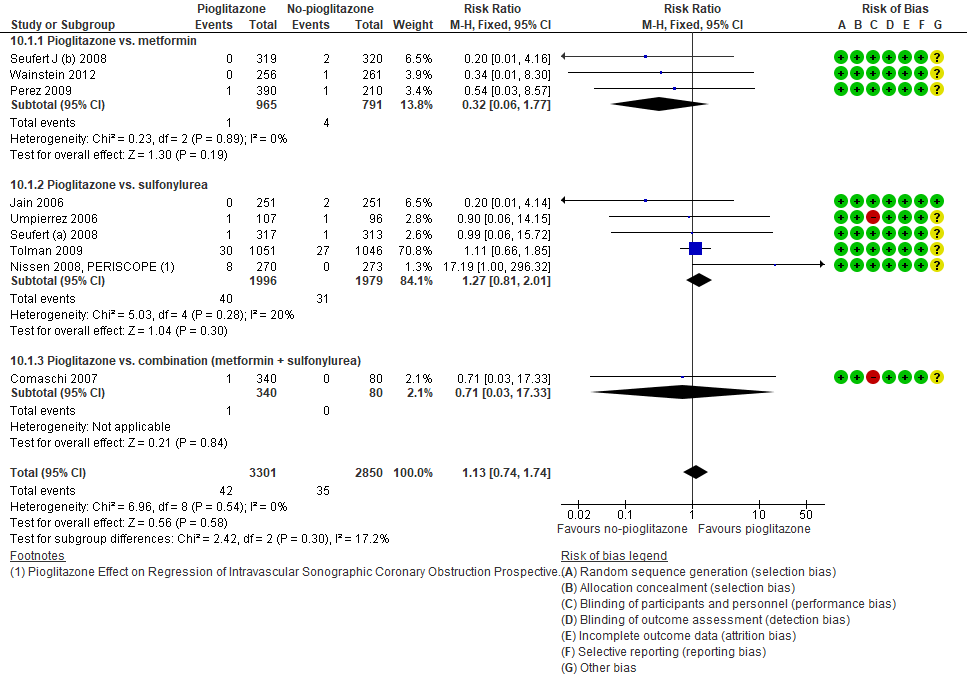


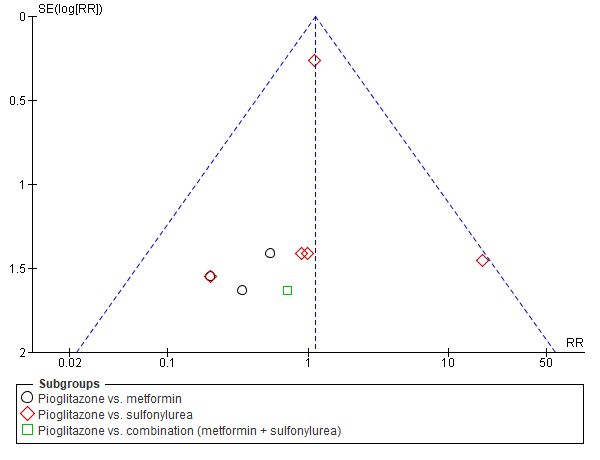


Figure S26. Forest and funnel plot of pioglitazone and fracture by AHGs subclass, Fixed-effect model.


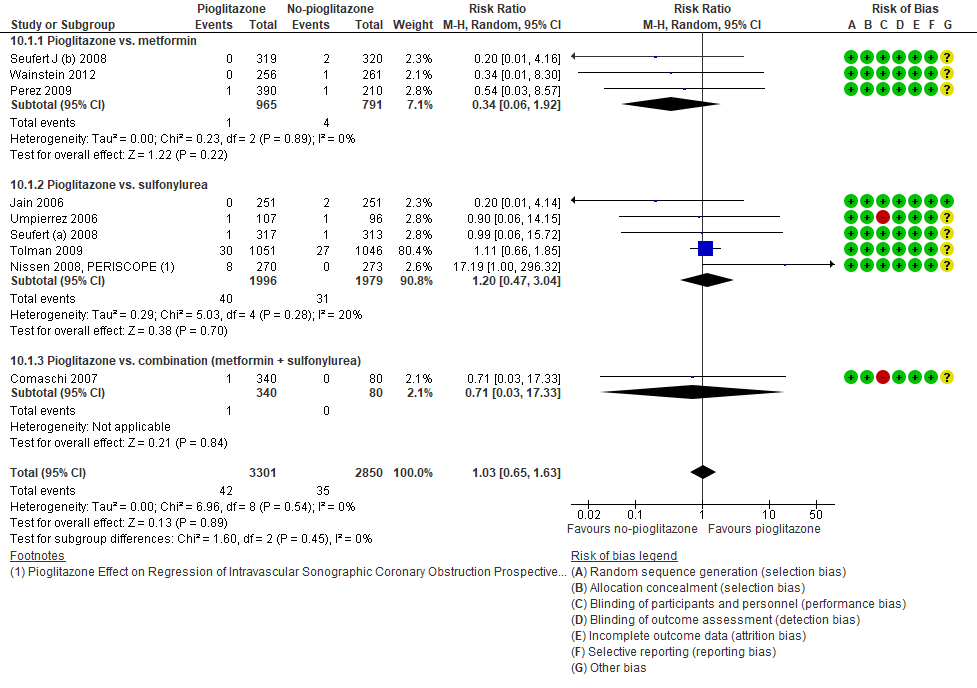


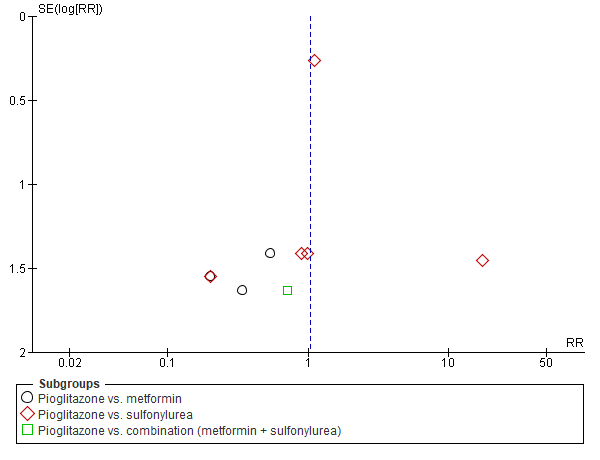


Figure S27. Forest and funnel plot of pioglitazone and fracture by AHGs subclass, Random-effect model.

Table S23. GRADE evidence profile of pioglitazone and fracture by AHGs subclass

| **Certainty assessment** | | | | | | | **Summary of findings** | | | | |
| --- | --- | --- | --- | --- | --- | --- | --- | --- | --- | --- | --- |
| **№ of participants (trials)** | **Risk of bias** | **Inconsistency** | **Indirectness** | **Imprecision** | **Publication bias** | **Overall certainty of evidence** | **Study event rates (%)** | | **Relative effect (95% CI)** | **Anticipated absolute effects** | |
|  |  |  |  |  |  |  | **With AHGs agents** | **With Pioglitazone** |  | **Risk with placebo** | **Risk difference with pioglitazone** |
| **Pioglitazone vs. AHGs subclass** **(follow-up: range 24 to 144 weeks)** | | | | | | | | | | | |
| 6151 (9 RCTs) | serious^a^ | not serious | not serious | very serious^b^ | none | ⨁◯◯◯  VERY LOW | 35/2850 (1.2%) | 42/3301 (1.3%) | RR 1.13 (0.74 to 1.74) | 1 per 100 | 0 fewer per 100 (from 0 fewer to 1 more) |
| **Pioglitazone vs. metformin (follow-up: range 24 to 52 weeks)** | | | | | | | | | | | |
| 1756 (3 RCTs) | not serious | not serious | not serious | very serious^c^ | none | ⨁⨁◯◯ LOW | 4/791 (0.5%) | 1/965 (0.1%) | RR 0.32 (0.06 to 1.77) | 1 per 100 | 0 fewer per 100 (from 0 fewer to 0 fewer) |
| **Pioglitazone vs. sulfonylurea** **(follow-up: range 28 to 144 weeks)** | | | | | | | | | | | |
| 3975 (5 RCTs) | serious^a^ | not serious | not serious | very serious^d^ | none | ⨁◯◯◯  VERY LOW | 31/1979 (1.6%) | 40/1996 (2.0%) | RR 1.27 (0.81 to 2.01) | 2 per 100 | 0 fewer per 100 (from 0 fewer to 2 more) |
| **Pioglitazone vs. combination treatment of AHGs (metformin + sulfonylurea)** **follow-up: mean 24 weeks** | | | | | | | | | | | |
| 420 (1 RCT) | serious^a^ | not serious | not serious | very serious^e^ | none | ⨁◯◯◯  VERY LOW | 0/80 (0.0%) | 1/340 (0.3%) | RR 0.71 (0.03 to 17.33) | 0 per 100 | 0 fewer per 100 (from 0 fewer to 0 fewer) |

***Abbreviations:*** **CI:** Confidence interval; **RCTs:** randomized controlled trials; **RR:** Risk ratio.

***Note:*** The GRADE scores were from the fixed-effect model.

#### GRADE evidence

**a.** Two trials with a small weight (pioglitazone versus sulfonylurea [2.8%]) and (pioglitazone versus metformin and sulfonylurea [2.1%]) with the overall EE rated as a high risk of bias due to a lack of blinding (open-label design) out of nine trials.

**b.** The overall imprecision was precise and narrow CIs with no significant effect size difference (P = 0.58). The 95% CI was not consistent with the possibility for a substantial negative effect exceeding the MID, including only 77 fracture events with a large sample size.

**c.** The overall imprecision was precise with no significant effect size difference (P = 0.19). However, all trials reported overlapping and narrow CIs. The 95% CI was not consistent with the possibility for a substantial negative effect exceeding the MID, including five fracture events with a small sample size (1,756).

**d.** The overall imprecision was precise with no significant effect size difference (P = 0.30). However, all trials reported overlapping CIs, in which three trials reported wide CIs. The 95% CI was not consistent with the possibility for a substantial negative effect exceeding the MID, including 71 fracture events with a large sample size.

**e.** The overall imprecision was precise without significant effect size difference (P = 0.84). However, one trial reported overlapping and wide CIs. The 95% CI was not consistent with the possibility for a substantial negative effect exceeding the MID, including one fracture event with a small sample size (420).

In summary, the overall certainty of the pooled EE had a low imprecision without a significant effect size difference in the level of evidence (P = 0.58). However, the majority of trials reported overlapping CIs, in which four trials reported wide CIs. One trial (1.3%) did not cross the line of no difference (1), in which reports a significant association of fracture in the direction of pioglitazone favor across sulfonylurea. The 95% CI was not consistent with the possibility for a substantial negative effect exceeding the MID, including a total of 77 fracture events with a large sample size. There was no evidence of inconsistency, significant heterogeneity (P = 0.54; I^2^ = 0%), or subgroup difference (P = 0.30; I^2^ = 17.2%). Majority of trials had low risk of bias (95.1%). However, the magnitude of effect of some trials that suffered from limitations likely resulted in a biased assessment of the intervention effect due to performance and selection biases (two trials with an open-label design out of nine). The indirectness of the trials indicated a high level of evidence, with an individual trial PICOS element aligning close to the review PICOS. Finally, there was no evidence of reporting bias; all included trials were within the two boundaries of the pyramid.

### Pioglitazone and fracture by risk of bias and comparators

The use of pioglitazone increased the occurrence of fracture in low risk of bias trials (RR, 1.23; 95% CI, 1.02–1.48; P = 0.03) versus placebo compared with trials with an unclear or high risk of bias using a fixed-effect model only (Figure S28). The GRADE scores were moderate (Table S24).


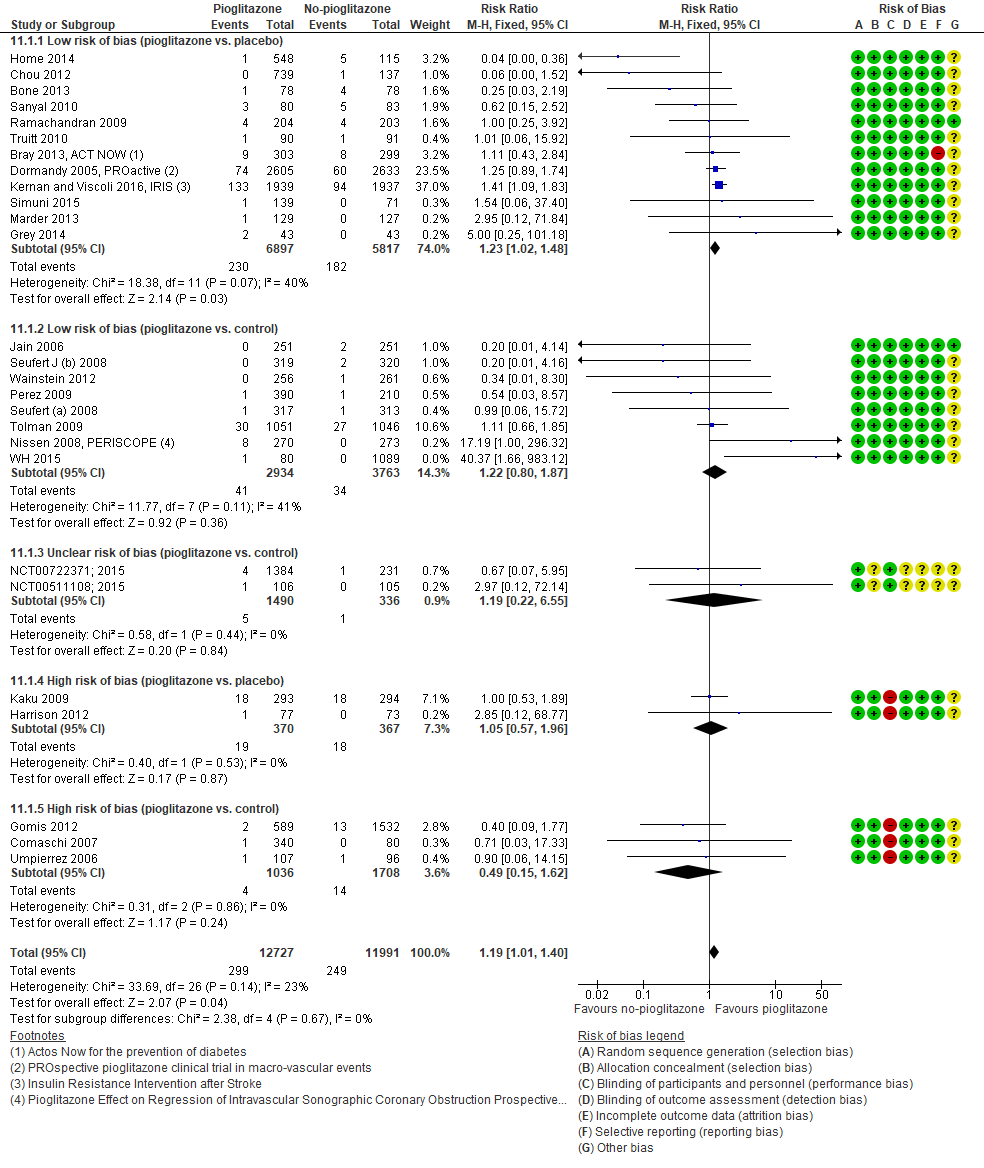


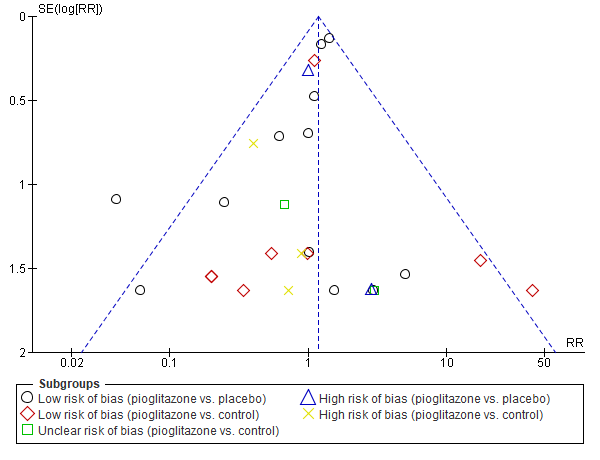


Figure S28. Forest and funnel plot of pioglitazone and fracture by risk of bias and comparators, Fixed-effect model.

The use of pioglitazone did not increase the occurrence of fracture in trials with a low, unclear, or high risk of bias (RR, 1.04; 95% CI, 0.79–1.38; P = 0.76) versus non-pioglitazone using a random-effect model (Figure S29).


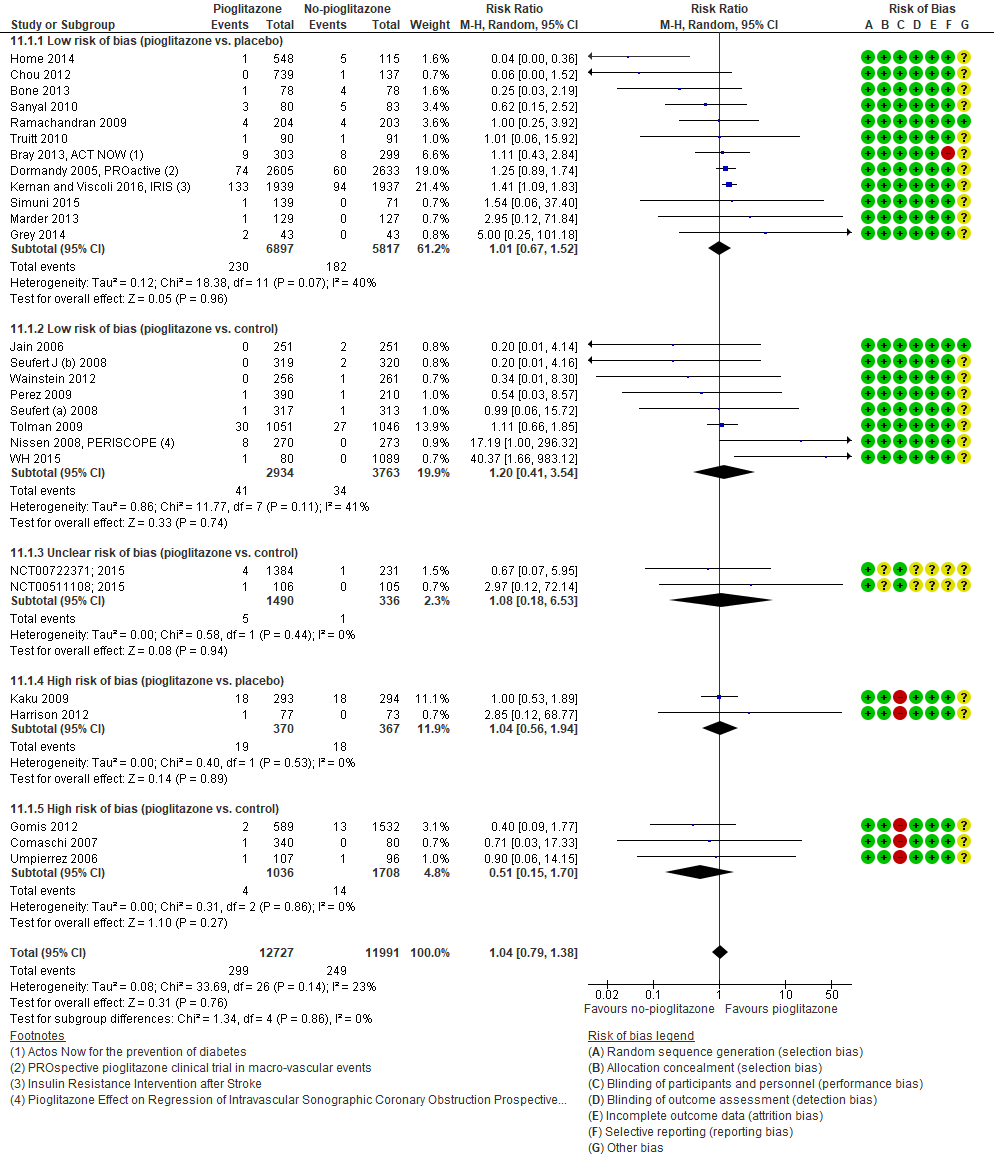


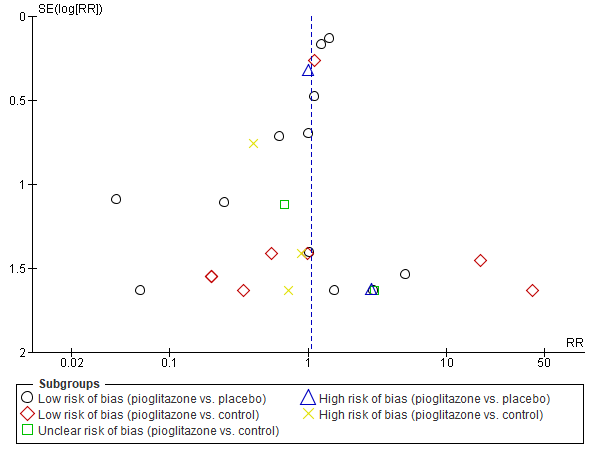


Figure S29. Forest and funnel plot of pioglitazone and fracture by risk of bias and comparators, Random-effect model.

Table S24. GRADE evidence profile of pioglitazone and fracture by risk of bias and comparators

| **Certainty assessment** | | | | | | | **Summary of findings** | | | | |
| --- | --- | --- | --- | --- | --- | --- | --- | --- | --- | --- | --- |
| **№ of participants (trials)** | **Risk of bias** | **Inconsistency** | **Indirectness** | **Imprecision** | **Publication bias** | **Overall certainty of evidence** | **Study event rates (%)** | | **Relative effect (95% CI)** | **Anticipated absolute effects** | |
|  |  |  |  |  |  |  | **With placebo / controls** | **With Pioglitazone** |  | **Risk with placebo / controls** | **Risk difference with pioglitazone** |
| **Pioglitazone and fracture by risk of bias and comparators (follow-up: range 24 to 251 weeks)** | | | | | | | | | | | |
| 24718 (27 RCTs) | serious^a^ | not serious | not serious | not serious | none | ⨁⨁⨁◯ MODERATE | 249/11991 (2.1%) | 299/12727 (2.4%) | RR 1.18 (1.00 to 1.39) | 2 per 100 | 0 fewer per 100 (0 fewer to 1 more) |
| **Low risk of bias (pioglitazone vs. placebo) follow-up: range 26 to 251 weeks** | | | | | | | | | | | |
| 12714 (12 RCTs) | not serious | not serious | not serious | serious^b^ | none | ⨁⨁⨁◯ MODERATE | 182/5817 (3.1%) | 230/6897 (3.3%) | RR 1.22 (1.01 to 1.47) | 3 per 100 | 1 more per 100 (0 fewer to 1 more) |
| **Low risk of bias (pioglitazone vs. control) follow-up: range 24 to 144 weeks** | | | | | | | | | | | |
| 6697 (6 RCTs) | not serious | not serious | not serious | very serious^c^ | none | ⨁⨁◯◯ LOW | 34/3763 (1.4%) | 41/2934 (1.5%) | RR 1.18 (0.76 to 1.84) | 1 per 100 | 0 fewer per 100 (0 fewer to 1 more) |
| **Unclear risk of bias (pioglitazone vs. control) follow-up: range 24 to 78 weeks** | | | | | | | | | | | |
| 1826 (4 RCTs) | not serious | not serious | not serious | very serious^d^ | none | ⨁⨁◯◯ LOW | 1/336 (0.1%) | 5/1490 (0.3%) | RR 1.57 (0.53 to 4.69) | 0 per 100 | 0 fewer per 100 (0 fewer to 0 fewer) |
| **High risk of bias (pioglitazone vs. placebo) follow-up: range 88 to 192 weeks** | | | | | | | | | | | |
| 737 (2 RCTs) | serious^a^ | not serious | not serious | very serious^e^ | none | ⨁◯◯◯  VERY LOW | 18/367 (4.9%) | 19/370 (5.1%) | RR 1.05 (0.57 to 1.96) | 5 per 100 | 0 fewer per 100 (2 fewer to 5 more) |
| **High risk of bias (pioglitazone vs. control) follow-up: range 24 to 78 weeks** | | | | | | | | | | | |
| 2744 (3 RCTs) | serious^a^ | not serious | not serious | very serious^f^ | none | ⨁◯◯◯  VERY LOW | 14/1708 (0.8%) | 4/1036 (0.4%) | RR 0.49 (0.15 to 1.62) | 1 per 100 | 0 fewer per 100 (1 fewer to 1 more) |

***Abbreviations:*** **CI:** Confidence interval; **RCTs:** randomized controlled trials; **RR:** Risk ratio.

***Note:*** The GRADE scores were from the fixed-effect model.

#### GRADE evidence

**a.** Five trials with a small weight in high risk of bias trials (pioglitazone vs. placebo [7.1% and 0.2%] and (pioglitazone vs. control [2.8%, 0.4%, and 0.3%] with the overall EE rated as a high risk of bias due to a lack of blinding (open-label design) out of 27 trials.

**b.** The overall imprecision was precise with a significant effect size difference (P = 0.03). However, all trials reported overlapping CIs, in which four trials reported wide CIs. The 95% CI was consistent with the possibility for a substantial negative effect exceeding the MID, including only 412 fracture events with a large sample size.

**c.** The overall imprecision was precise with no significant effect size difference (P = 0.36). However, all trials reported overlapping CIs, in which three trials reported wide CIs. The 95% CI was not consistent with the possibility for a substantial negative effect exceeding the MID, including only 75 fracture events with a large sample size.

**d.** The overall imprecision was precise with no significant effect size difference (P = 0.84). However, all trials reported overlapping CIs, in which one trial reported wide CIs. The 95% CI was not consistent with the possibility for a substantial negative effect exceeding the MID, including only six fractures event with a small sample size (1,826).

**e.** The overall imprecision was precise with no significant effect size difference (P = 0.87). However, all trials reported overlapping CIs, in which one trial reported wide CIs. The 95% CI was not consistent with the possibility for a substantial negative effect exceeding the MID, including only 37 fracture events with a small sample size (737).

**f.** The overall imprecision was precise with no significant effect size difference (P = 0.24). However, all trials reported overlapping CIs, in which two trials reported wide CIs. The 95% CI was not consistent with the possibility for a substantial negative effect exceeding the MID, including only 18 fracture events with a large sample size.

In summary, the overall certainty of the pooled EE had a moderate imprecision with a significant effect size difference in the level of evidence (P = 0.04). However, the majority of trials reported overlapping CIs, in which 11 trials reported wide CIs. The largest (37%) and smallest trials (0.0%) did not cross the line of no difference (1), in which reports a significant association of fracture in the direction of pioglitazone favor across non-pioglitazone, and one small trial (3.2%) in the direction of non-pioglitazone favor across pioglitazone. The 95% CI was consistent with the possibility for a substantial negative effect exceeding the MID, including 548 fracture events with a large sample size. There was no evidence of inconsistency, significant heterogeneity (P = 0.14; I^2^ = 23%), or subgroup difference across trials (P = 0.67; I^2^ = 0%). The majority of trials had a low risk of bias (89.2%). The overall magnitude of reporting bias did not affect our results, as these trials were double-blind RCTs. However, the magnitude of effect of some trials that suffered from limitations likely resulted in a biased assessment of the intervention effect due to performance and selection biases (five trials with open-label design out of 27). The indirectness of the trials indicated a high level of evidence, with an individual trial PICOS element aligning close to the review PICOS. Finally, there was no evidence of reporting bias. However, two trials reported an outlier outside the pyramid edge due to multiple interventional groups resulting in wide CIs.

### Pioglitazone and fracture by treatment dose

The cumulative use of pioglitazone 15–45 mg/day increased the occurrence of fracture (RR, 1.29; 95% CI, 1.08–1.54; P = 0.005) versus non-pioglitazone using a fixed-effect model (Figure S30). However, the cumulative use of pioglitazone 30 mg/day, 45 mg/day, or combinations doses of 15–30 mg/day or 30–45 mg/day did not increase the occurrence of fracture versus non-pioglitazone. The GRADE scores were moderate (Table S25).


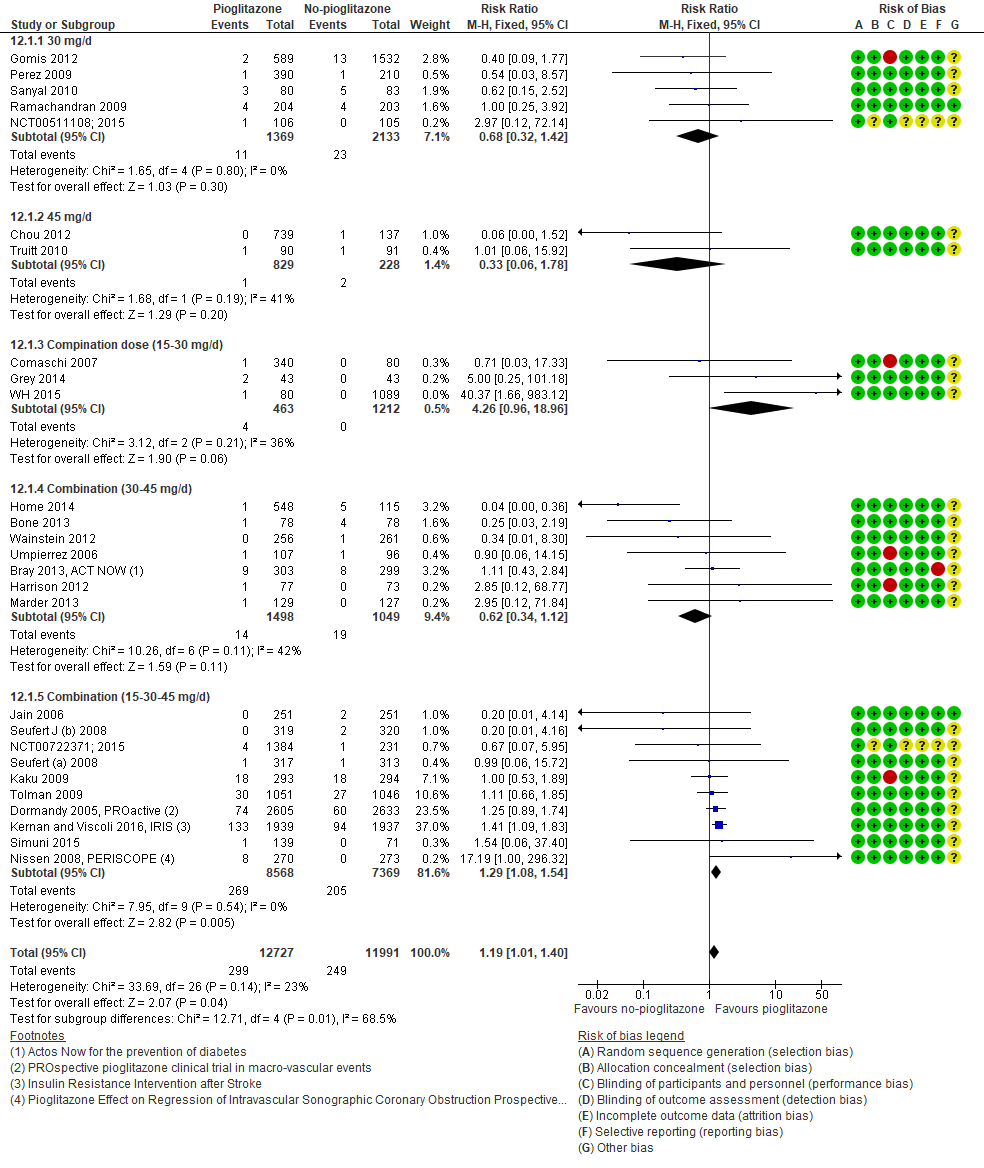


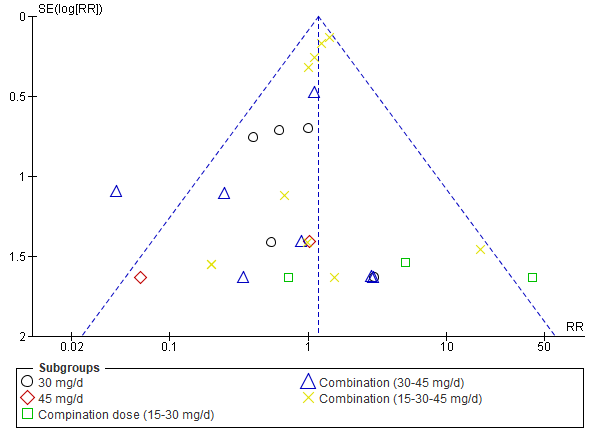


Figure S30. Forest and funnel plot of pioglitazone and fracture by treatment dose, Fixed-effect model.

The cumulative use of pioglitazone 15–45 mg/day increased the occurrence of fracture (RR, 1.27; 95% CI, 1.07–1.53; P = 0.008) versus non-pioglitazone, using a random-effect model (Figure S31). However, the cumulative use of pioglitazone 30 mg/day, 45 mg/day, or combinations doses of 15–30 mg/day or 30–45 mg/day did not increase the occurrence of fracture versus non-pioglitazone.


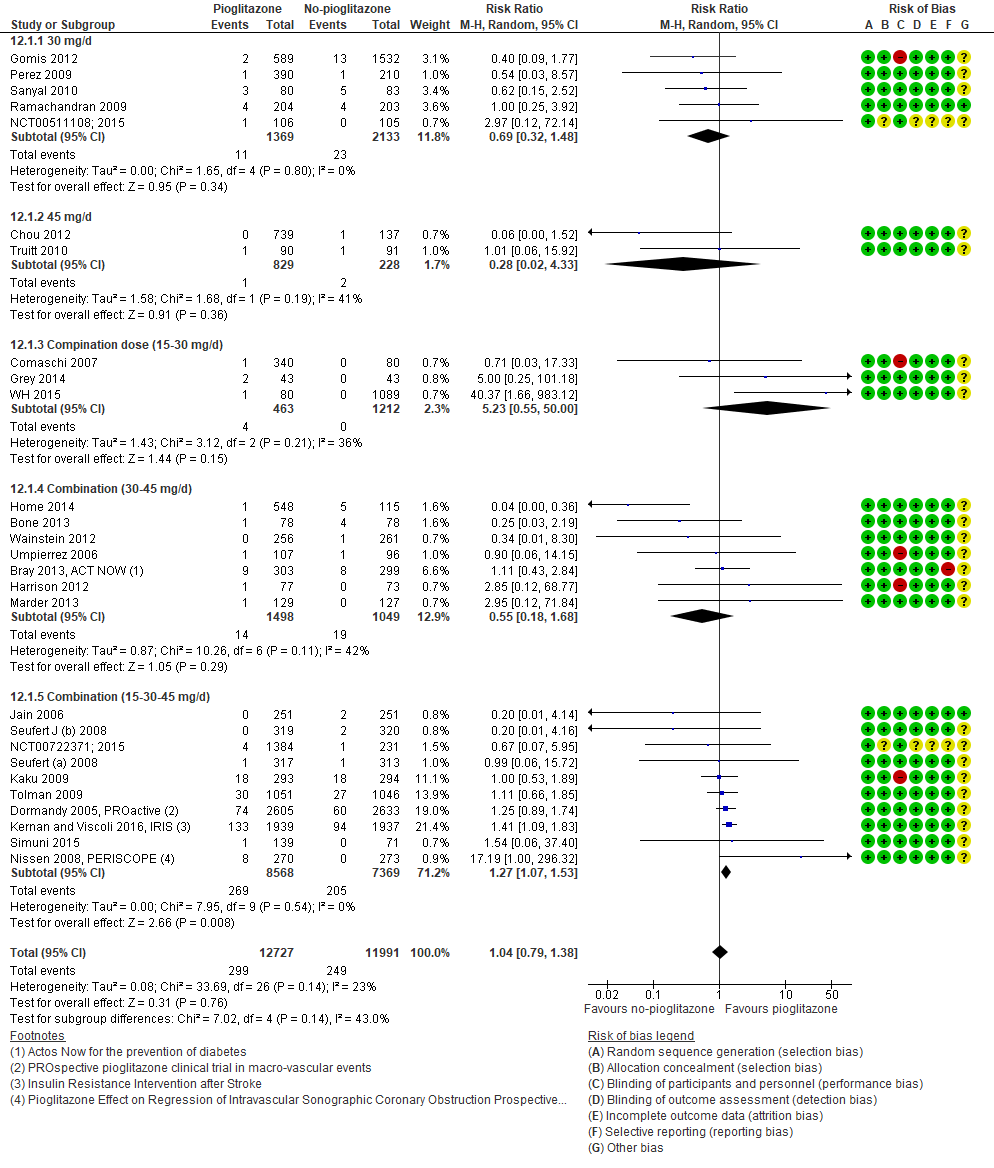


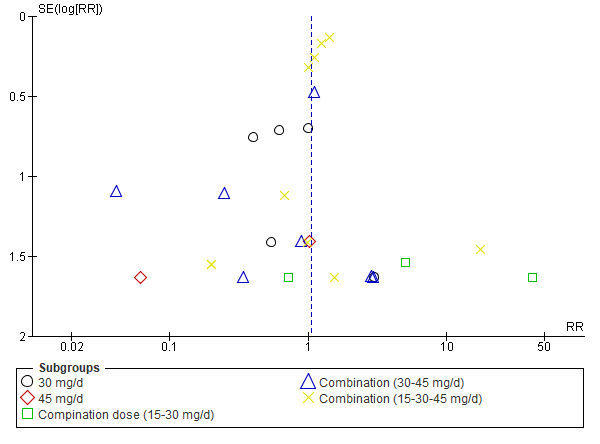


Figure S31. Forest and funnel plot of pioglitazone and fracture by treatment dose, Random-effect model.

Table S25. GRADE evidence profile of pioglitazone and fracture by treatment dose

| **Certainty assessment** | | | | | | | **Summary of findings** | | | | |
| --- | --- | --- | --- | --- | --- | --- | --- | --- | --- | --- | --- |
| **№ of participants (trials)** | **Risk of bias** | **Inconsistency** | **Indirectness** | **Imprecision** | **Publication bias** | **Overall certainty of evidence** | **Study event rates (%)** | | **Relative effect (95% CI)** | **Anticipated absolute effects** | |
|  |  |  |  |  |  |  | **With placebo / controls** | **With Pioglitazone** |  | **Risk with placebo / controls** | **Risk difference with pioglitazone** |
| **Pioglitazone and fracture by treatment dose (follow-up: range 24 to 251 weeks)** | | | | | | | | | | | |
| 24718 (27 RCTs) | serious^a^ | not serious | not serious | not serious | none | ⨁⨁⨁◯ MODERATE | 249/11991 (2.1%) | 299/12727 (2.3%) | RR 1.19 (1.01 to 1.39) | 2 per 100 | 0 fewer per 100 (0 fewer to 1 more) |
| **30 mg/day (follow-up: range 24 to 157 weeks)** | | | | | | | | | | | |
| 3502 (5 RCTs) | serious^a^ | not serious | not serious | very serious^b^ | none | ⨁◯◯◯  VERY LOW | 23/2133 (1.1%) | 11/1369 (0.8%) | RR 0.68 (0.32 to 1.42) | 1 per 100 | 0 fewer per 100 (1 fewer to 0 fewer) |
| **45 mg/day (follow-up: mean 26 weeks)** | | | | | | | | | | | |
| 1057 (2 RCTs) | not serious | not serious | not serious | very serious^c^ | none | ⨁⨁◯◯ LOW | 2/228 (0.9%) | 1/829 (0.1%) | RR 0.33 (0.06 to 1.78) | 1 per 100 | 1 fewer per 100 (1 fewer to 1 more) |
| **Combination dose of 15 to 30 mg/day (follow-up: range 24 to 78 weeks)** | | | | | | | | | | | |
| 1675 (3 RCTs) | serious^a^ | not serious | not serious | very serious^d^ | none | ⨁◯◯◯  VERY LOW | 0/1212 (0.0%) | 4/463 (0.9%) | RR 4.26 (0.96 to 18.96) | 0 per 100 | 0 fewer per 100 (0 fewer to 0 fewer) |
| **Combination dose of 30 to 45 mg/day (follow-up: range 28 to 156 weeks)** | | | | | | | | | | | |
| 2547 (7 RCTs) | serious^a^ | not serious | not serious | very serious^e^ | none | ⨁◯◯◯  VERY LOW | 19/1049 (1.8%) | 14/1498 (0.9%) | RR 0.62 (0.34 to 1.12) | 2 per 100 | 1 fewer per 100 (1 fewer to 0 fewer) |
| **Combination dose of 15, 30 and 45 mg/day (follow-up: range 44 to 251 weeks)** | | | | | | | | | | | |
| 15937 (10 RCTs) | serious^a^ | not serious | not serious | serious^f^ | none | ⨁⨁◯◯ LOW | 205/7369 (2.8%) | 269/8568 (3.1%) | RR 1.29 (1.08 to 1.54) | 3 per 100 | 1 more per 100 (0 fewer to 2 more) |

***Abbreviations:*** **CI:** Confidence interval; **mg/day:** milligram pear day; **RCTs:** randomized controlled trials; **RR:** Risk ratio.

***Note:*** The GRADE scores were from the fixed-effect model.

#### GRADE evidence

**a.** Five trials with a small weight ([15, 30, 45 mg/day (7.1%)], [30 mg/day (2.8%)], [15 to 30 mg/day (0.3%)], and [30 to 45 mg/day (0.4% and 0.2%)]) with the overall EE rated as a high risk of bias due to a lack of blinding (open-label design) out of 27 trials.

**b.** The overall imprecision was precise with no significant effect size difference (P = 0.30). However, all trials reported overlapping CIs, in which one trial reported wide CIs. The 95% CI was not consistent with the possibility for a substantial negative effect exceeding the MID, including only 34 fracture events with a large sample size.

**c.** The overall imprecision was precise with no significant effect size difference (P = 0.20). However, all trials reported overlapping CIs, in which one trial reported wide CIs. The 95% CI was not consistent with the possibility for a substantial negative effect exceeding the MID, including only three fracture events with a small sample size (1,057).

**d.** The overall imprecision was precise with no significant effect size difference (P = 0.06). However, all trials reported overlapping and wide CIs. The 95% CI was not consistent with the possibility for a substantial negative effect exceeding the MID, including only four fracture events with a small sample size (1,675).

**e.** The overall imprecision was precise with no significant effect size difference (P = 0.11). However, all trials reported overlapping CIs, in which three trials reported wide CIs. The 95% CI was not consistent with the possibility for a substantial negative effect exceeding the MID, including only 33 fracture events with a large sample size.

**f.** The overall imprecision was precise with a significant effect size difference (P = 0.005). However, all trials reported overlapping CIs, in which three trials reported wide CIs. The 95% CI was consistent with the possibility a substantial negative effect exceeding the MID, including only 474 fracture events with a large sample size.

In summary, the overall certainty of the pooled EE had a moderate imprecision with a significant effect size difference in the level of evidence (P = 0.04). However, the majority of trials reported overlapping CIs, in which 11 trials reported wide CIs. The largest (37%) and smallest trials (0.0%) did not cross the line of no difference (1), in which two trials reports a significant association of fracture in the direction of pioglitazone favor across non-pioglitazone, and one small trial (3.2%) in the direction of non-pioglitazone favor across pioglitazone. The 95% CI was consistent with the possibility for a substantial negative effect exceeding the MID, including 548 fracture events with a large sample size. There was no evidence of inconsistency and significant heterogeneity (P = 0.14; I^2^ = 23%), but with subgroup difference across overlapping trials (P = 0.01; I^2^ = 68.5%). The majority of trials had a low risk of bias (89.2%). The overall magnitude of reporting bias did not affect our results, as these trials were double-blind RCTs. However, the magnitude of effect of some trials that suffered from limitations likely resulted in a biased assessment of the intervention effect due to performance and selection biased (five trials with an open-label design out of 27). The indirectness of the trials indicated a high level of evidence, with an individual trial PICOS element aligning close to the review PICOS. Finally, there was no evidence of reporting bias. However, two trials report an outlier outside the pyramid edge due to multiple interventional groups resulting in wide CIs.

## References

1. Viscoli M, Inzucchi S, Young L, et al. Pioglitazone and Risk for Bone Fracture: Safety Data From a Randomized Clinical Trial. J Clin Endocrinol Metab 2017;102:914-22.

2. Simuni T, NINDS exploratory trials in parkinson disease (NET-PD) groups. Pioglitazone in early Parkinson's disease: a phase 2, multicentre, double-blind, randomised trial. Lancet 2015;14:795-803.

3. Grey A, Bolland M, Fenwick S, et al. The skeletal effects of pioglitazone in type 2 diabetes or impaired glucose tolerance: a randomized controlled trial. Eur J Endocrinol 2013;170:255-62.

4. Bray G, Smith S, Banerji M, et al. Effect of pioglitazone on body composition and bone density in subjects with prediabetes in the ACT NOW trial. Diabetes Obes Metabo 2013;15:931-7.

5. Bone H, Lindsay R, McClung M, Perez A, Raanan M, Spanheimer R. Effects of pioglitazone on bone in postmenopausal women with impaired fasting glucose or impaired glucose tolerance: a randomized, double-blind, placebo-controlled study. J Clin Endocrinol Metab 2013;98:4691-701.

6. Marder W, Khalatbari S, Myles J, et al. The peroxisome proliferator activated receptor-γ pioglitazone improves vascular function and decreases disease activity in patients with rheumatoid arthritis. J Am Heart Assoc 2013;2:e00044.

7. Sanyal A, Chalasani N, Kowdley K, et al. Pioglitazone, vitamin E, or placebo for nonalcoholic steatohepatitis. NEJM 2010;362:1675-85.

8. Ramachandran A, Snehalatha C, Mary S, et al. Pioglitazone does not enhance the effectiveness of lifestyle modification in preventing conversion of impaired glucose tolerance to diabetes in Asian Indians: results of the Indian diabetes prevention programme-2 (IDPP-2). Diabetologia 2009;52:1019-26.

9. Aithal G, Thomas J, Kaye P, et al. Randomized, placebo-controlled trial of pioglitazone in nondiabetic subjects with nonalcoholic steatohepatitis. Gastroenterology 2008;135:1176-84.

10. Dormandy J, Bhattacharya M, van Troostenburg de Bruyn A. Safety and tolerability of pioglitazone in high-risk patients with type 2 diabetes: an overview of data from PROactive. Drug Safety 2009;32:187-202.

11. Home P, Shamanna P, Stewart M, et al. Efficacy and tolerability of albiglutide versus placebo or pioglitazone over 1 year in people with type 2 diabetes currently taking metformin and glimepiride: HARMONY 5. Diabetes Obes Metab 2014;17:179-87.

12. Chou H, Truitt K, Moberly J, et al. A 26-week, placebo- and pioglitazone-controlled monotherapy study of rivoglitazone in subjects with type 2 diabetes mellitus. Diabetes Obes Metab 2012;14:1000-9.

13. Truitt K, Goldberg R, Rosenstock J, et al. A 26-week, placebo- and pioglitazone-controlled, dose-ranging study of rivoglitazone, a novel thiazolidinedione for the treatment of type 2 diabetes. Curr Med Res Opin 2010;26:1321-31.

14. ClinicalTrials.gov. MK0431 and pioglitazone co-administration factorial study in patients with type 2 diabetes mellitus (0431-102 AM2). (<https://clinicaltrials.gov/ct2/show/NCT00722371>).

15. ClinicalTrials.gov. Sitagliptin and pioglitazone mechanism of action study in type 2 diabetes mellitus (0431-061). (<https://clinicaltrials.gov/ct2/show/NCT00511108>).

16. Kaku K, Daida H, Kashiwagi A, et al. Long-term effects of pioglitazone in Japanese patients with type 2 diabetes without a recent history of macrovascular morbidity. Curr Med Res Opin 2009;25:2925-32.

17. Abe M, Okada K, Maruyama T, Maruyama N, Soma M, Matsumoto K. Clinical effectiveness and safety evaluation of long-term pioglitazone treatment for erythropoietin responsiveness and insulin resistance in type 2 diabetic patients on hemodialysis. Expert Opin Pharmacother 2010;11:1611-1620.

18. Yan H, Xia M, Wang Y, et al. Efficacy of berberine in patients with non-alcoholic fatty liver disease. PLoS One 2015;10:e0134172.

19. Harrison S, Hamzeh F, Han J, Pandya P, Sheikh M, Vierling J. Chronic hepatitis C genotype 1 patients with insulin resistance treated with pioglitazone and peginterferon alpha‐2a plus ribavirin. Hepatology 2012;56:464-73.

20. Gold M, Alderton C, Zvartau-Hind M, et al. Rosiglitazone monotherapy in mild-to-moderate Alzheimer's disease: results from a randomized, double-blind, placebo-controlled phase III study. Dement Geriatr Cogn Disord 2010;30:131-46.

21. Gruntmanis U, Fordan S, Ghayee H, et al. The peroxisome proliferator-activated receptor-gamma agonist rosiglitazone increases bone resorption in women with type 2 diabetes: a randomized, controlled trial. Calcif Tissue Int 2010;86:343-9.

22. ClinicalTrials.gov. RAS rosiglitazone and atherosclerosis study: a 1 year randomised, double-blind, parallel group, placebo controlled study to evaluate the efficacy of rosiglitazone on the progression of intima-media thickness in the carotid artery in subjects with insulin resistance syndrome and/or type 2 diabetes mellitus [study no 049653/334]. GlaxoSmithKline, Brentford (UK) (2008). (<http://ctr.gsk.co.uk/Summary/rosiglitazone/studylist.asp>).

23. ClinicalTrials.gov. Rosiglitazone and plaque study: a 12 month randomised, double-blind, placebocontrolled, magnetic resonance imaging study to evaluate the effect of rosiglitazone on the structure and composition of carotid atherosclerotic plaques in subjects with type 2 diabetes mellitus and coexisting vascular disease or hypertension [study no 049653/351] GlaxoSmithKline, Brentford (UK) (2008). (<http://ctr.gsk.co.uk/Summary/rosiglitazone/studylist.asp>).

24. Zinman B, Harris S, Neuman J, et al. Low-dose combination therapy with rosiglitazone and metformin to prevent type 2 diabetes mellitus (CANOE trial): a double-blind randomised controlled study. Lancet 2010;376:103-11.

25. Frye R, August P, Brooks M, et al. A randomized trial of therapies for type 2 diabetes and coronary artery disease. NEJM 2009;360:2503-15.

26. Erdmann E, Califf R, Gerstein H, et al. Effects of the dual peroxisome proliferator-activated receptor activator aleglitazar in patients with type 2 diabetes mellitus or prediabetes. Am Heart J 2015;170:117-22.

27. Esposito K, Maiorino M, Di Palo C, et al. Effects of pioglitazone versus metformin on circulating endothelial microparticles and progenitor cells in patients with newly diagnosed type 2 diabetes--a randomized controlled trial. Diabetes Obes Metab 2011;13:439-45.

28. Seufert J, Urquhart R. 2-year effects of pioglitazone add-on to sulfonylurea or metformin on oral glucose tolerance in patients with type 2 diabetes. Diabetes Res Clin Pract 2008;79:453-60.

29. Hanefeld M, Brunetti P, Schernthaner G, Matthews D, B; C, QUARTET Study Group. One-year glycemic control with a sulfonylurea plus pioglitazone versus a sulfonylurea plus metformin in patients with type 2 diabetes. Diabetes Care 2004;27:141-7.

30. Perez A, Zhao Z, Jacks R, Spanheimer R. Efficacy and safety of pioglitazone/metformin fixed-dose combination therapy compared with pioglitazone and metformin monotherapy in treating patients with T2DM. Curr Med Res Opin 2009;25:2915-23.

31. Wainstein J, Katz L, Engel S, et al. Initial therapy with the fixed-dose combination of sitagliptin and metformin results in greater improvement in glycaemic control compared with pioglitazone monotherapy in patients with type 2 diabetes. Diabetes Obes Metab 2012;14:409-18.

32. Bilezikian J, Josse R, Eastell R, et al. Rosiglitazone decreases bone mineral density and increases bone turnover in postmenopausal women with type 2 diabetes mellitus. J Clin Endocrinol Metab 2013;98:1519-28.

33. Borges J, Bilezikian J, Jones-Leone A, et al. A randomized, parallel group, double-blind, multicentre study comparing the efficacy and safety of Avandamet (rosiglitazone/metformin) and metformin on long-term glycaemic control and bone mineral density after 80 weeks of treatment in drug-naïve type 2 diabetes mellitus patients. Diabetes Obes Metabolism 2011;13:1036-46.

34. Sheu WH, Gantz I, Chen M, et al. Safety and efficacy of omarigliptin (MK-3102), a novel once-weekly DPP-4 inhibitor for the treatment of patients with type 2 diabetes. Diabetes Care 2015;38:2106-14.

35. Tolman K, Freston J, Kupfer S, Perez A. Liver safety in patients with type 2 diabetes treated with pioglitazone: results from a 3-year, randomized, comparator-controlled study in the US. Drug Safety 2009;32:787-800.

36. Jain R, Osei K, Kupfer S, Perez A, Zhang J. Long-term safety of pioglitazone versus glyburide in patients with recently diagnosed type 2 diabetes mellitus. Pharmacotherapy 2006;26:1388-95.

37. Nissen E, Nicholls J, Wolski K, et al. Comparison of pioglitazone vs glimepiride on progression of coronary atherosclerosis in patients with type 2 diabetes: the PERISCOPE randomized controlled trial. JAMA 2008:1561-73.

38. Umpierrez G, Issa M, Vlajnic A. Glimepiride versus pioglitazone combination therapy in subjects with type 2 diabetes inadequately controlled on metformin monotherapy: results of a randomized clinical trial. Curr Med Res Opin 2006;22:751-59.

39. Matthews D, Charbonnel B, Hanefeld M, Brunetti P, Schernthaner G. Long-term therapy with addition of pioglitazone to metformin compared with the addition of gliclazide to metformin in patients with type 2 diabetes: a randomized, comparative study. Diabetes Metab Res Rev 2005;21:167-74.

40. Comaschi M, Demicheli A, Di Pietro C, Bellatreccia A, Mariz S; COM06 Study Investigators. Effects of pioglitazone in combination with metformin or a sulfonylurea compared to a fixed-dose combination of metformin and glibenclamide in patients with type 2 diabetes. Diabetes Technol Ther 2007;9:387-98.

41. Home P, Pocock S, Beck-Nielsen H, et al. Rosiglitazone evaluated for cardiovascular outcomes in oral agent combination therapy for type 2 diabetes (RECORD): a multicentre, randomised, open-label trial. Lancet 2009;373:2125-35.

42. Kahn S, Zinman B, Lachin J, et al. Rosiglitazone-associated fractures in type 2 diabetes - an analysis from a diabetes outcome progression trial (ADOPT). Diabetes Care 2008;31:845-51.

43. ClinicalTrials.gov. Rosiglitazone-metformin combination versus metformin-sulfonylurea combination on beta-cell function in type 2 diabetes. (<https://clinicaltrials.gov/ct2/show/NCT00367055>).

44. ClinicalTrials.gov. A randomised, multi-centre, phase IV, double-blind, parallel group study comparing the effects of 52 weeks' administration of AVANDAMET and metformin plus sulphonylurea on change in HbA1c from baseline in overweight type 2 diabetics poorly controlled on metformin [study no AVM100264]. Glaxo-SmithKline, Brentford (UK) (2008). (<http://ctr.gsk.co.uk/Summary/rosiglitazone/studylist.asp>).

45. DeFronzo R, Burant C, Fleck P, Wilson C, Mekki Q, Pratley R. Efficacy and tolerability of the DPP-4 inhibitor alogliptin combined with pioglitazone, in metformin-treated patients with type 2 diabetes. J Clin Endocrinol Metab 2012;97:1615-22.

46. Rosenstock J, Inzucchi S, Seufert J, Fleck P, Wilson C, Mekki Q. Initial combination therapy with alogliptin and pioglitazone in drug-naïve patients with type 2 diabetes. Diabetes Care 2010;33:2406-8.

47. Gomis R, Owens D, Taskinen M, et al. Long‐term safety and efficacy of linagliptin as monotherapy or in combination with other oral glucose‐lowering agents in 2121 subjects with type 2 diabetes: up to 2 years exposure in 24‐week phase III trials followed by a 78‐week open‐label extension. Int J Clin Pract 2012;66:731-40.

48. ClinicalTrials.gov. The safety and efficacy of linagliptin (5mg / once daily) given for 52 weeks as add-on therapy to patients with type 2 diabetes mellitus and insufficient glycaemic control despite diet, exercise, and treatment with one approved antidiabetic drug. (<https://clinicaltrials.gov/ct2/show/NCT01204294>).

49. Takihata M, Nakamura A, Tajima K, et al. Comparative study of sitagliptin with pioglitazone in Japanese type 2 diabetic patients: the COMPASS randomized controlled trial. Diabetes Obes Metab 2013;15:455-62.

50. ClinicalTrials.gov. A study to determine the long term safety and efficacy of albiglutide in combination with oral monotherapy antihyperglycemic medications in Japanese patients with type 2 diabetes mellitus. (<https://clinicaltrials.gov/ct2/show/NCT01777282>).

51. ClinicalTrials.gov. A study of LY2189265 in Japanese participants with type 2 diabetes mellitus. (<https://clinicaltrials.gov/ct2/show/NCT01468181>).

52. Araki E, Tanizawa Y, Tanaka Y, et al. Long-term treatment with empagliflozin as add-on to oral antidiabetes therapy in Japanese patients with type 2 diabetes mellitus. Diabetes Obes Metab 2015;17.

53. Abdul-Ghani M, Puckett C, Triplitt C, et al. Initial combination therapy with metformin, pioglitazone and exenatide is more effective than sequential add-on therapy in subjects with new-onset diabetes. Results from the efficacy and durability of initial combination therapy for type 2 diabetes (EDICT): a randomized trial. Diabetes Obes Metab 2015;17:268-75.

54. Ruilope L, Hanefeld M, Lincoff A, et al. Effects of the dual peroxisome proliferator-activated receptor-α/γ agonist aleglitazar on renal function in patients with stage 3 chronic kidney disease and type 2 diabetes: a Phase IIb, randomized study. BMC Nephrology 2014;15:180.

55. Bosi E, Ellis G, Wilson C, Fleck P. Alogliptin as a third oral antidiabetic drug in patients with type 2 diabetes and inadequate glycaemic control on metformin and pioglitazone: a 52-week, randomized, double-blind, active-controlled, parallel-group study. Diabetes Obes Metab 2011;13:1088-96.

56. Yoon K, Steinberg H, Teng R, et al. Efficacy and safety of initial combination therapy with sitagliptin and pioglitazone in patients with type 2 diabetes: a 54-week study. Diabetes Obes Metab 2012;14.

57. Kashiwagi A, Kadowaki T, Tajima N, et al. Sitagliptin added to treatment with ongoing pioglitazone for up to 52 weeks improves glycemic control in Japanese patients with type 2 diabetes. J Diabetes Investig 2011;2:381-90.

58. ClinicalTrials.gov. A study to evaluate the safety and efficacy of sitagliptin 100 mg in participants with type 2 diabetes mellitus who have inadequate glycemic control (MK-0431-229). (<https://clinicaltrials.gov/ct2/show/NCT01076075>).

59. Rosenstock J, Brazg R, Andryuk P, Lu K, P; S, Group* ftSS. Efficacy and safety of the dipeptidyl peptidase-4 inhibitor sitagliptin added to ongoing pioglitazone therapy in patients with type 2 diabetes: a 24-week, multicenter, randomized, double-blind, placebo-controlled, parallel-group study. Clinical Therapeutics 2006;28.

60. Fonseca V, Staels B, Morgan J, et al. Efficacy and safety of sitagliptin added to ongoing metformin and pioglitazone combination therapy in a randomized, placebo-controlled, 26-week trial in patients with type 2 diabetes. J Diabetes Complicat 2013;27:177-83.

61. Charbonnel B, Karasik A, Liu J, Wu M, Meininger G, Sitagliptin Study 020 Group. Efficacy and safety of the dipeptidyl peptidase-4 inhibitor sitagliptin added to ongoing metformin therapy in patients with type 2 diabetes inadequately controlled with metformin alone. Diabetes Care 2006;29:2638-43.

62. Forst T, Guthrie R, Goldenberg R, et al. Efficacy and safety of canagliflozin over 52 weeks in patients with type 2 diabetes on background metformin and pioglitazone. Diabetes Obes Metab 2014;16:467-77.

63. Kovacs C, Seshiah V, Swallow R, et al. Empagliflozin improves glycaemic and weight control as add-on therapy to pioglitazone or pioglitazone plus metformin in patients with type 2 diabetes: a 24-week, randomized, placebo-controlled trial. Diabetes Obes Metab 2014;16:147-58.

64. Philis-Tsimikas A, Prato D, Satman I, et al. Effect of insulin degludec versus sitagliptin in patients with type 2 diabetes uncontrolled on oral antidiabetic agents. Diabetes Obes Metab 2013;15:760-6.

65. Nauck M, di Domenico M, Patel S, Kobe M, Toorawa R, Woerle H. Linagliptin and pioglitazone combination therapy versus monotherapy with linagliptin or pioglitazone: A randomised, double-blind, parallel-group, multinational clinical trial. Diabetes Vasc Dis Res 2016;13:286-98.

66. Gantz I, Okamoto T, Ito Y, et al. A randomized, placebo-controlled trial evaluating the safety and efficacy of adding omarigliptin to antihyperglycemic therapies in Japanese patients with type 2 diabetes and inadequate glycemic control. Diabetes Therapy 2017;8:793-810.

67. Hollander P, Li J, Frederich R, Allen E, R; C, CV181013 Investigators. Safety and efficacy of saxagliptin added to thiazolidinedione over 76 weeks in patients with type 2 diabetes mellitus. Diabetes Vasc Dis Res 2011;8:125-35.

68. Kadowaki T, K. K. Efficacy and safety of teneligliptin in combination with pioglitazone in Japanese patients with type 2 diabetes mellitus. J Diabetes Investig 2013;4:576-84.

69. Davies M, Bergenstal R, Bode B, et al. Efficacy of liraglutide for weight loss among patients with type 2 diabetes: the SCALE diabetes randomized clinical trial. JAMA 2015;314:687-99.

70. Kiyosue A, Seino Y, Nishijima K, Bosch‐Traberg H, Kaku K. Safety and efficacy of the combination of the glucagon‐like peptide‐1 receptor agonist liraglutide with an oral antidiabetic drug in Japanese patients with type 2 diabetes: post‐hoc analysis of a randomized, 52‐week, open‐label, parallel‐group trial. J Diabetes Investig 2018;9:831-9.

71. Norwood P, Liutkus J, Haber H, Pintilei E, Boardman M, Trautmann M. Safety of exenatide once weekly in patients with type 2 diabetes mellitus treated with a thiazolidinedione alone or in combination with metformin for 2 years. Clinical Therapeutics 2012;34:2082-90.

72. Wysham C, Blevins T, Arakaki R, et al. Efficacy and safety of dulaglutide added onto pioglitazone and metformin versus exenatide in type 2 diabetes in a randomized controlled trial (AWARD-1). Diabetes Care 2014;37:2159-67.

73. ClinicalTrials.gov. Addition of exenatide to insulin glargine In type 2 diabetes mellitus. (<https://clinicaltrials.gov/ct2/show/NCT00765817>).

74. Bode B, Stenlöf K, Sullivan D, Fung A, Usiskin K. Efficacy and safety of canagliflozin treatment in older subjects with type 2 diabetes mellitus: a randomized trial. Hospital Practice 2015;41: 72-84.

75. Rosenstock J, Vico M, Wei L, Salsali A, List J. Effects of dapagliflozin, an SGLT2 inhibitor, on HbA1c, body weight, and hypoglycemia risk in patients With type 2 diabetes inadequately controlled on pioglitazone monotherapy. Diabetes Care 2012;35:1473-8.

76. Garber A, King A, Del Prato S, et al. Insulin degludec, an ultra-longacting basal insulin, versus insulin glargine in basal-bolus treatment with mealtime insulin aspart in type 2 diabetes (BEGIN basal-bolus type 2): a phase 3, randomised, open-label, treat-to-target non-inferiority trial. Lancet 2012;379:1498-507.

77. Fulcher G, Mehta R, Fita E, Ekelund M, Bain S. Efficacy and safety of IDegAsp versus BIAsp 30, both twice daily, in elderly patients with type 2 diabetes: post hoc analysis of two phase 3 randomized controlled BOOST trials. Diabetes Therapy 2019;10:107-18.

78. Gross J, Rojas A, Shah S, Tinahones F, Cleall S, Rodríguez A. Efficacy and safety of a premixed versus a basal-plus insulin regimen as intensification for type 2 diabetes by timing of the main meal. Curr Med Res Opin 2016;32:1109-16.

79. Dormandy A, Charbonnel B, Eckland J, et al. Secondary prevention of macrovascular events in patients with type 2 diabetes in the PROactive Study (PROspective pioglitAzone Clinical Trial In macroVascular Events): a randomised controlled trial. Lancet 2005;366:1279-89.

80. Kernan W, Viscoli M, Furie L, et al. Pioglitazone after ischemic stroke or transient ischemic attack. NEJM 2016;374:1321-31.
